# Absolute Configurations of 14,15-Hydroxylated Prenylxanthones from a Marine-Derived *Aspergillus* sp. Fungus by Chiroptical Methods

**DOI:** 10.1038/s41598-018-28996-5

**Published:** 2018-07-13

**Authors:** Ao Zhu, Meng-Yue Yang, Ya-Hui Zhang, Chang-Lun Shao, Chang-Yun Wang, Lian-Dong Hu, Fei Cao, Hua-Jie Zhu

**Affiliations:** 1 College of Pharmaceutical Sciences, Key Laboratory of Pharmaceutical Quality Control of Hebei Province, Key Laboratory of Medicinal Chemistry and Molecular Diagnostics of Education Ministry of China, Hebei University, Baoding, 071002 People’s Republic of China; 2 Key Laboratory of Marine Drugs, The Ministry of Education of China, School of Medicine and Pharmacy, Ocean University of China, Qingdao, 266003 People’s Republic of China

## Abstract

Determination of the absolute configrations for natural products is one of the most important and challenging tasks, especially when the molecules display high conformational flexibility. In this paper, eight new prenylxanthones, aspergixanthones A-H (**1**–**8**), and one known analogue (**9**), were isolated from the marine-derived fungus *Aspergillus* sp. ZA-01. The absolute configurations of C-14 and C-15 in **1**–**8** were difficult to be assigned due to the high conformational flexibility of the chains. To solve this problem, the experimental ECD, ORD, and VCD spectra of **1** were combined for analysis with the corresponding theoretical predictions for its different diastereomers. This study suggested that a concerted application of more than one chiroptical methods could be used as a preferable approach for the stereochemical characterizations of flexible molecules. Compounds **1**–**9** were evaluated for their cytotoxic and antibacterial activities. Among them, **6** showed cytotoxicity against the A-549 cell line with the IC_50_ value of 1.1 *μ*M, and **7** exhibited antibacterial activity against *Micrococcus lysodeikticus* with the MIC value of 0.78 *μ*g/mL.

## Introduction

**Figure 1:**
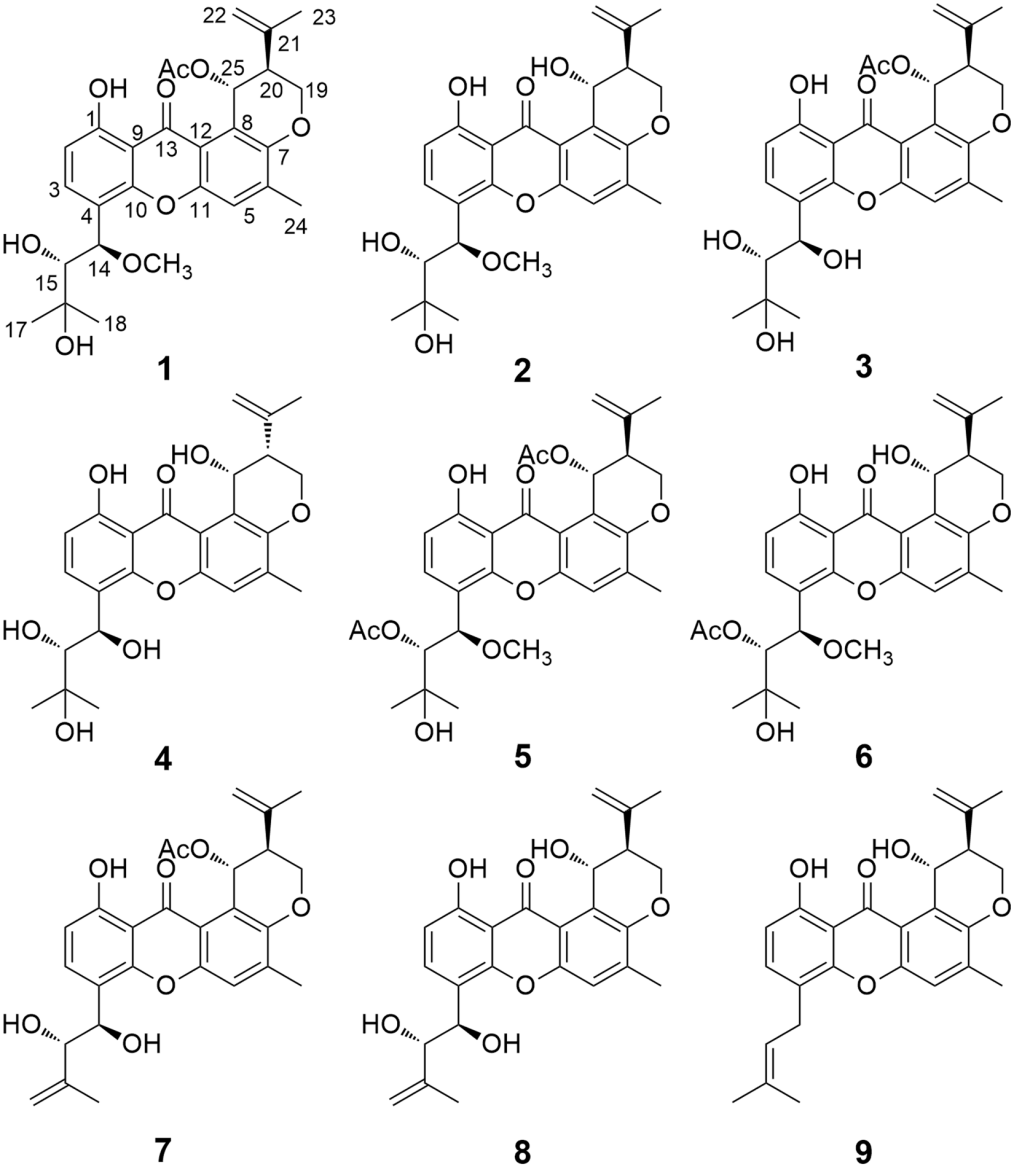
Chemical structures of 1–9.

Prenylated derivatives, including prenylated polyketides^1–7^, prenylated alkaloids^8,9^, prenylated flavones^10^, and so on, which contained conformationally flexible terpenoid-derived carbon chains, made such a task extremely challenging to assign their absolute configurations. Specially, when the terpenoid-derived carbons in prenylated derivatives were hydroxylated, it was more difficult to solve this problem by common means as the high free rotation of the stereogenic centers in chains^3–7^. Fortunately, the concerted application of more than one chiroptical methods has recently emerged as a hopeful approach for the stereochemical characterizations of prenylated derivatives^11–13^. In the course of our ongoing works to discover bioactive secondary metabolites from fungal sources^14–16^, the marine-derived fungus *Aspergillus* sp. ZA-01 was chosen for research as its TLC and HPLC-UV profiles of the secondary metabolites. As a result, eight new prenylated xanthones (prenylxanthones), aspergixanthones A–H (**1**–**8**) and one known shamixanthone (**9**)^5^, were obtained from the fungus *Aspergillus* sp., which was grown on rice in solid culture. It was hard to assign the absolute configurations of C-14 and C-15 in chains of **1**–**8** as the free rotation of the stereogenic centers. To address this problem, a combined analysis of ECD, ORD, and VCD was performed for **1**, its conclusion was further confirmed by using the Snatzke′s method. The cytotoxic and antibacterial activities of **1**–**9** were also evaluated. Herein, we report the isolation, absolute configurations, and biological activities of **1**–**9** (Fig. 1).

## Results and Discussion

**Figure 2:**
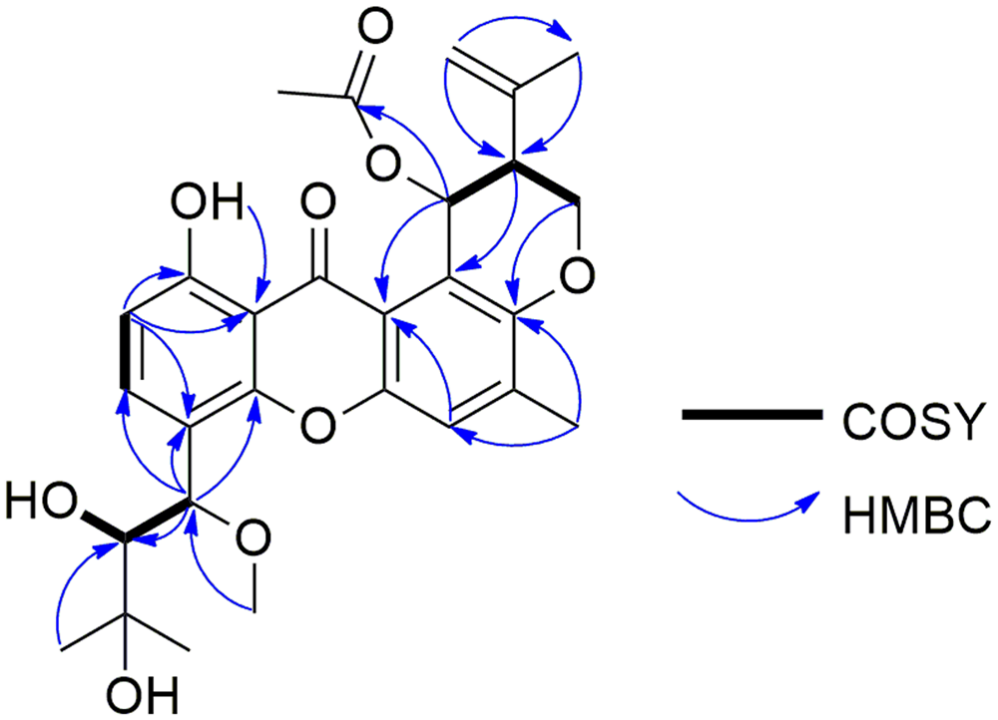
COSY and key HMBC correlations of 1.

Aspergixanthone A (**1**) was isolated as a yellow powder, with the molecular formula of C_28_H_32_O_9_ (12 degrees of unsaturation) established by positive HRESIMS (*m/z* 535.1935 [M + Na]^+^, calcd. for 535.1939). In the NMR data of **1** (Tables 1 and 2), one keto carbonyl [*δ*_C_ 183.0 (C-13)], three aromatic signals [*δ*_H_ 7.66 (1 H, d, *J* = 8.4 Hz, H-3), 7.24 (1 H, s, H-5), and 6.82 (1 H, d, *J* = 8.4 Hz, H-2); *δ*_C_ 135.3 (C-3), 120.2 (C-5), and 110.4 (C-2)], one disubstituted double bond [*δ*_H_ 4.80 (1 H, s, H-22a), 4.75 (1 H, s, H-22b); *δ*_C_ 141.5 (C-21) and 112.7 (C-22)], and four methyls [*δ*_H_ 2.35 (3 H, s, H-24), 1.88 (3 H, s, H-23), 1.40 (3 H, s, H-18), and 1.25 (3 H, s, H-17); *δ*_C_ 26.5 (C-18), 26.4 (C-17), 22.3 (C-23) and 17.2 (C-24)] were present. Along with the IR absorptions of **1**, it was found that **1** contains the presence of hydroxy (3445 cm^−1^), aromatic ring (1635 cm^−1^), and aromatic ketone (1591 cm^−1^) groups. The above characteristic NMR and IR data indicated that **1** belongs to the family of prenylxanthones skeleton^3–6^. Careful comparison of the 1D and 2D NMR spectra of **1** with those of the known compound tajixanthone hydrate, previously isolated from the fungus *Emericella variecolor*^5^, indicated that **1** and tajixanthone hydrate share the same prenylxanthone nucleus structure. The detailed comparison of 1D NMR data between **1** and tajixanthone hydrate suggested the presence of an additional acetoxy [*δ*_H_ 2.07 (3 H, s, 25-OCOCH_3_); *δ*_C_ 170.0 (25-O*C*OCH_3_) and 21.2 (25-OCO*C*H_3_)] and an additional methoxyl [*δ*_H_ 3.28 (3 H, s, 14-OCH_3_); *δ*_C_ 56.6 (14-OCH_3_)] in **1**. The observed key HMBC correlations (Fig. 2) from H-14 to C-3, C-4 and C-15, and from 14-OCH_3_ to C-14 revealed the methoxyl connected to position C-14 in **1**, while the key HMBC correlations from H-25 to 25-O*C*OCH_3_ implied that the additional acetoxy was attached to C-25 in **1**. Therefore, **1** was identified as the 14-methoxyl-25-acetyl derivative of tajixanthone hydrate.

**Table 1:** ^1^H NMR Data (*δ*) of **1**–**4** (600 MHz, *δ* in ppm, *J* in Hz).

No.	1^*a*^	2^*a*^	3^*b*^	4^*b*^
2	6.82, d (8.4)	6.86, d (8.4)	6.77, d (8.4)	6.78, d (8.4)
3	7.66, d (8.4)	7.72, d (8.4)	7.82, d (8.4)	7.83, d (8.4)
5	7.24, s	7.21, s	7.50, s	7.40, s
14	5.10, brs	5.13, d (2.4)	5.47, d (2.8)	5.48, brs
15	3.45, brs	3.46, brs	3.27, brd (7.2)	3.27, brd (6.6)
17	1.25, s	1.25, s	1.21, s	1.22, s
18	1.40, s	1.42, s	1.28, s	1.29, s
19	4.54, brd (10.8)	4.43, brd (10.8)	4.56, brd (11.4)	4.46, brd (11.4)
	4.31, dd (10.8, 2.4)	4.35, dd (10.8, 2.4)	4.20, dd (11.4, 2.4)	4.34, dd (11.4, 2.4)
20	2.71, brs	2.72, brs	2.68, brs	2.51, brs
22	4.80, s	4.78, s	4.79, s	4.74, s
	4.75, s	4.56, s	4.61, s	4.55, s
23	1.88, s	1.84, s	1.81, s	1.78, s
24	2.35, s	2.35, s	2.31, s	2.29, s
25	6.89, brs	5.40, brs	6.81, brs	5.81, brs
1-OH	12.97, brs	12.71, brs	12.78, brs	12.84, brs
14-OH/OCH_3_	3.28, s	3.29, s	5.19, d (5.4)	5.17, d (3.6)
15-OH	2.73, brs	2.95, brs	4.44, brd (7.2)	4.46, brd (6.6)
16-OH	—	—	4.55, brs	4.54, brs
25-OH/OAc	2.07, s	4.94, d (2.4)	1.99, s	5.26, d (3.6)

^*a*^Recorded in CDCl_3_. ^b^Recorded in DMSO-*d*_6_.

**Table 2:** ^13^C NMR Data (*δ*) of **1**–**8** (150 MHz, *δ* in ppm).

No.	1^*a*^	2^*a*^	3^*b*^	4^*b*^	5^*a*^	6^*a*^	7^*a*^	8^*a*^
1	161.7, C	161.5, C	159.6, C	159.6, C	162.0, C	161.8, C	161.7, C	161.4, C
2	110.4, CH	110.5, CH	109.2, CH	109.0, CH	110.0, CH	110.1, CH	110.3, CH	110.3, CH
3	135.3, CH	135.9, CH	135.8, CH	135.6, CH	134.1, CH	134.6, CH	134.5, CH	135.0, CH
4	115.0, C	115.5, C	122.3, C	122.2, C	113.4, C	113.8, C	117.7, C	118.2, C
5	120.2, CH	119.0, C	120.7, CH	119.1, CH	120.3, CH	119.1, CH	120.2, CH	118.9, CH
6	137.6, C	138.6, C	137.6, C	137.2, C	137.6, C	138.6, C	137.7, C	138.7, C
7	150.2, C	149.6, C	149.9, C	148.7, C	150.3, C	149.7, C	150.3, C	149.8, C
8	115.2, C	121.2, C	114.7, C	121.0, C	115.1, C	121.2, C	115.0, C	121.4, C
9	109.0, C	108.9, C	108.1, C	108.1, C	109.1, C	109.0, C	108.2, C	108.7, C
10	152.2, C	152.4, C	151.4, C	151.1, C	152.2, C	152.4, C	152.0, C	152.1, C
11	151.5, C	151.7, C	150.5, C	150.5, C	151.5, C	151.7, C	151.6, C	151.8, C
12	116.3, C	116.9, C	115.5, C	115.8, C	116.5, C	117.0, C	116.3, C	116.8, C
13	183.0, C	184.2, C	183.1, C	183.4, C	183.0, C	184.2, C	183.1, C	184.3, C
14	76.2, CH	76.2, CH	65.3, CH	65.2, CH	76.4, CH	76.5, CH	68.8, CH	68.8, CH
15	78.3, CH	78.2, CH	78.0, CH	78.0, CH	77.0, CH	77.0, CH	79.1, CH	79.1, CH
16	72.9, C	72.9, C	72.7, C	72.7, C	72.7, C	72.7, C	143.4, C	143.4, C
17	26.4, CH_3_	26.5, CH_3_	26.2, CH_3_	26.2, CH_3_	26.9, CH_3_	26.9, CH_3_	113.7, CH_2_	113.7, CH_2_
18	26.5, CH_3_	26.5, CH_3_	27.5, CH_3_	27.5, CH_3_	27.9, CH_3_	27.9, CH_3_	18.6, CH_3_	18.6, CH_3_
19	63.8, CH_2_	64.5, CH_2_	63.5, CH_2_	63.5, CH_2_	63.8, CH_2_	64.4, CH_2_	63.8, CH_2_	64.8, CH_2_
20	42.5, CH	44.8, CH	41.7, CH	44.4, CH	42.5, CH	44.8, CH	42.5, CH	45.0, CH
21	141.5, C	142.5, C	141.8, C	142.8, C	141.6, C	142.6, C	141.4, C	142.5, C
22	112.7, CH_2_	112.2, CH_2_	112.6, CH_2_	111.9, CH_2_	112.7, CH_2_	112.2, CH_2_	112.8, CH_2_	112.3, CH_2_
23	22.3, CH_3_	22.5, CH_3_	22.1, CH_3_	22.4, CH_3_	22.4, CH_3_	22.5, CH_3_	22.4, CH_3_	22.5, CH_3_
24	17.2, CH_3_	17.3, CH_3_	16.9, CH_3_	16.9, CH_3_	17.3, CH_3_	17.4, CH_3_	17.3, CH_3_	17.4, CH_3_
25	65.4, CH	63.1, CH	64.8, CH	60.9, CH	65.4, CH	63.0, CH	65.5, CH	63.3, CH
14-OCH_3_	56.6, CH_3_	56.6, CH_3_	—	—	57.1, CH_3_	57.1, CH_3_	—	—
15-OAc	—	—	—	—	170.2, C	170.1, C	—	—
	—	—	—	—	20.5, CH_3_	20.4, CH_3_	—	—
25-OAc	170.0, C	—	169.3, C	—	170.1, C	—	170.0, C	—
	21.2, CH_3_	—	21.0, CH_3_	—	21.3, CH_3_	—	21.2, CH_3_	—

^*a*^Recorded in CDCl_3_. ^b^Recorded in DMSO-*d*_6_.

Aspergixanthone B (**2**) was also obtained as a yellow powder. Its molecular formula was detetermined as C_26_H_30_O_8_ by positive HRESIMS, revealing the loss of a -COCH_3_ group compared with that of **1**. The NMR data (Tables 1 and 2) revealed that **2** had the same structural features as those of **1**, except for the absence of an acetoxy at C-25 in **2**. This observation was further demonstrated by the ^1^H-^1^H COSY cross-peaks of OH-25/H-25/H-20. Compound **2** was thus identified as the 25-deacetylation derivative of **1**.

Aspergixanthones C and D (**3** and **4**) were also isolated as yellow powder with the molecular formulas of C_27_H_30_O_9_ and C_25_H_28_O_8_ by positive HRESIMS, respectively. The 1D and 2D NMR data of **3** and **4** (Tables 1 and 2) revealed their similar prenylxanthone nucleus to those of **1** and **2**. Particularly, the NMR data showed the presence of a C-25 acetoxy group in **3** and a C-25 hydroxy group in **4**. Detailed analysis and comparison of the NMR and MS data of **3** and **4** with those of **1** and **2** revealed that the methoxy at C-14 in **1** and **2** was absent in **3** and **4**, which was verified by the each HMBC correlations from H-14 to C-3, C-4 and C-15 in **3** and **4**.

Aspergixanthones E and F (**5** and **6**) displayed quasi-molecular ions at *m/z* 577.2048 and 535.1539 [M + Na]^+^ in the positive HRESIMS, corresponding to the molecular formulas of C_30_H_34_O_10_ and C_28_H_32_O_9_, respectively. Compounds **5** and **6** were also prenylxanthone analogues by the comparison of the strikingly similar NMR data of **5** and **6** (Tables 2 and 3) with those of **1** and **2**, with the appearance of the additional acetoxy groups in **5** and **6**. The additional acetoxys laid within their respective side chains at C-15, demonstrated by the detailed analysis of ^1^H-^1^H COSY and HMBC spectra of **5** and **6**, respectively.

**Table 3:** ^1^H NMR Data (*δ*) of **5**–**8** (600 MHz, *δ* in ppm, CDCl_3_, *J* in Hz).

No.	5	6	7	8
2	6.77, d (8.4)	6.82, d (8.4)	6.79, d (8.4)	6.79, d (8.4)
3	7.59, d (8.4)	7.65, d (8.4)	7.73, d (8.4)	7.76, d (8.4)
5	7.24, s	7.22, s	7.24, s	7.20, s
14	5.41, d (1.8)	5.42, d (1.2)	5.28, brs	5.28, brs
15	5.00, d (2.4)	5.02, d (2.4)	4.27, d (6.0)	4.26, d (5.4)
17	1.22, s	1.22, s	4.82, s	4.82, s
18	1.56, s	1.56, s	1.82, s	1.83, s
19	4.54, brd (10.8)	4.43, brd (10.8)	4.55, brd (10.8)	4.41, brd (10.8)
	4.32, dd (10.8, 3.0)	4.36, dd (10.8, 3.0)	4.27, dd (10.8, 2.4)	4.33, dd (10.8, 2.4)
20	2.72, brs	2.73, brs	2.72, brs	2.73, d (2.4)
22	4.80, s	4.78, s	4.85, s	4.83, s
	4.74, s	4.55, s	4.77, s	4.60, s
23	1.88, s	1.84, s	1.89, s	1.86, s
24	2.36, s	2.37, s	2.35, s	2.35, s
25	6.88, brs	5.40, brs	6.90, brs	5.41, brs
1-OH	12.97, brs	12.74, brs	13.00, brs	12.70, brs
14-OH/OCH_3_	3.34, s	3.34, s	2.76, brs	2.94, brs
15-OH/OAc	1.92, s	1.91, s	2.52, brs	2.59, brs
25-OH/OAc	2.10, s	4.86, d (3.0)	2.08, s	4.99, d (4.2)

Aspergixanthones G and H (**7** and **8**) had the molecular formulas of C_27_H_28_O_8_ and C_25_H_26_O_7_, respectively. The NMR data (Tables 2 and 3) of **7** and **8** were similar to those of **3** and **4**, except that the presence of disubstituted double bond signals in **7** and **8** instead of the methyls in **3** and **4**. Comprehensive analysis of 2D NMR data of **7** and **8** supported that the C-17 methyls in **3** and **4** were oxidized to form the corresponding olefinic bonds in **7** and **8**, respectively.

The relative configurations of C-20 and C-25 of **1**–**8** were assigned by NOESY experiments and comparison of the NMR data with those of reported prenylxanthone derivatives^3–6^. In the NOESY spectra of **1**–**3** and **5**–**8**, the correlation between H-25 and CH_2_-22/CH_3_-23 was observed, suggesting these protons should be on the same face of the molecule. And, the ^1^H NMR data about *δ*_H_ 2.71 for H-20 suggested a trans-diaxal relationship of hydroxy and isopropenyl in **1**–**3** and **5**–**8**, which was consistent with the corresponding configuration of tajixanthone hydrate^4–6^. Whereas, the NOESY correlation between OH-25 and CH_3_-23 was unobserved and the ^1^H NMR data about *δ*_H_ 2.51 for H-20 indicated the cis-related relationship of H-20 and H-25 (twist-chair conformation) in **4**, which was close to literature values for known compound epitajixanthone hydrate (*δ*_H_ 2.55)^3^. The relative configurations of C-20 and C-25 in **4** were further confirmed by comparison of the positive specific rotation value [[*α*]_D_^20^ = +39.0 (*c* 0.1, MeOH)] of **4** with those of compound epitajixanthone hydrate [[*α*]_D_^24^ = +62.0 (*c* 0.1, CHCl_3_)]^3^ and compounds **1**–**3** and **5**–**8** (all of them were negative values, see Methods Section). However, the relative configurations of C-14 and C-15 in **1**–**8** was difficult to be determined due to the high conformational flexibility of the terpenoid-derived chains. Thus, eight possible absolute configurations [(14*R*, 15*R*, 20*S*, 25*R*)-**1**, [(14*R*, 15*S*, 20*S*, 25*R*)-**1**, (14*S*, 15*R*, 20*S*, 25*R*)-**1** and (14*S*, 15*S*, 20*S*, 25*R*)-**1**, and their enantiomers] were present for **1**.

In recent years, three most frequently used chiroptical methods, namely electronic circular dichroism (ECD), optical rotatory dispersion (ORD), and vibrational circular dichroism (VCD) have proven to be useful means for the stereochemical characterizations of natural products^17–19^. However, none of these chiroptical techniques was capable of dominating stereochemical characterizations, as they each had their respective limitations for the different structures^20^. ECD had one or two orders of magnitude higher sensitivity, but the stereogenic centers should be close to UV-Vis chromophores^21^. ORD has been popularly used in recent research, yet, it was hard to explain the spectra^21^. VCD, which require no chromophores in the UV-Vis region and have a larger scope than ECD, was limited by the sample quantity^21^. Thus, the use of more than one chiroptical techniques could provide more reliable results for complex products, especially for some flexible compounds^20,21^. In this paper, a combined analysis of ECD, ORD, and VCD properties was applied to elucidate the absolute configuration of conformationally flexible **1**.

**Figure 3:**
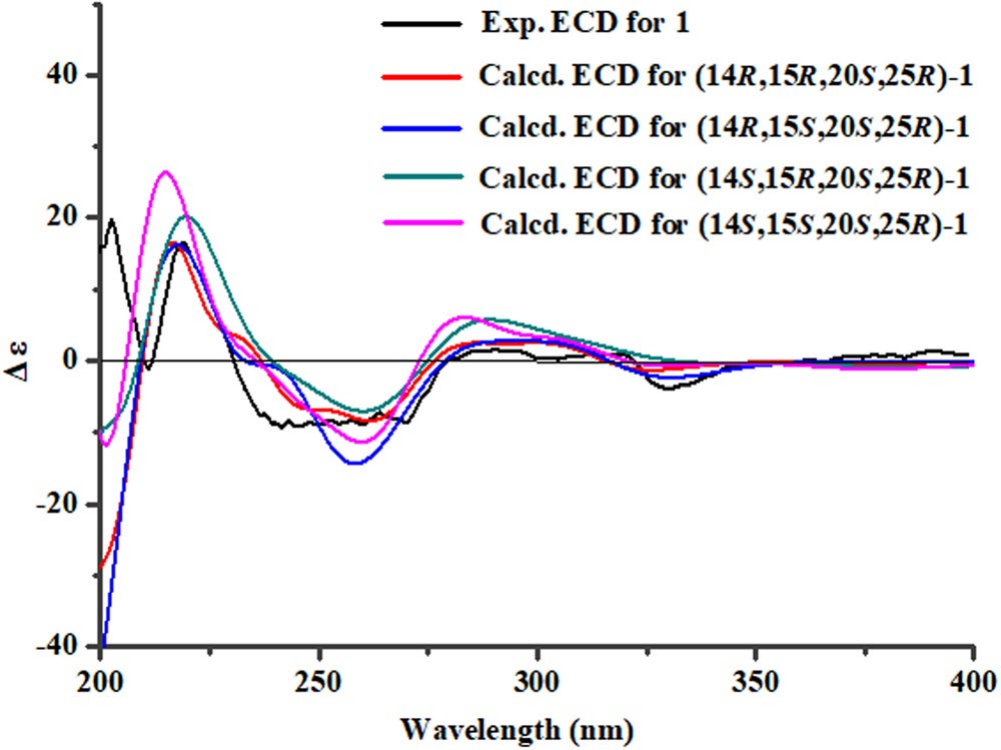
Experimental ECD spectrum of 1 and the calculated ECD spectra for its four diastereomers.

The half possibly structures of **1** [(14*R*, 15*R*, 20*S*, 25*R*)-**1**, (14*R*, 15*S*, 20*S*, 25*R*)-**1**, (14*S*, 15*R*, 20*S*, 25*R*)-**1** and (14*S*, 15*S*, 20*S*, 25*R*)-**1**] were firstly performed for quantum chemical time-dependent (TD)-DFT calculations of their ECD spectra. After the conformational optimizations at the gas-phase B3LYP/6-311 ++ G(d) level, the TD-DFT ECD calculations of all the conformers of (14*R*, 15*R*, 20*S*, 25*R*)-**1**, (14*R*, 15*S*, 20*S*, 25*R*)-**1**, (14*S*, 15*R*, 20*S*, 25*R*)-**1** and (14*S*, 15*S*, 20*S*, 25*R*)-**1** were calculated using the gas-phase B3LYP/6–311 ++ G(2d,p) level. The Boltzmann-weighted ECD spectra of the four diastereomers were generated using the SpecDis 1.6 soft with a standard deviation of σ 0.2 eV. As shown in Fig. 3, all of the predicted ECD curves for the four diastereomers matched well with the measured ECD spectrum of **1**, suggesting that the absolute configurations of C-20 and C-25 in **1** were assigned as 20*S*, 25*R*.

**Figure 4:**
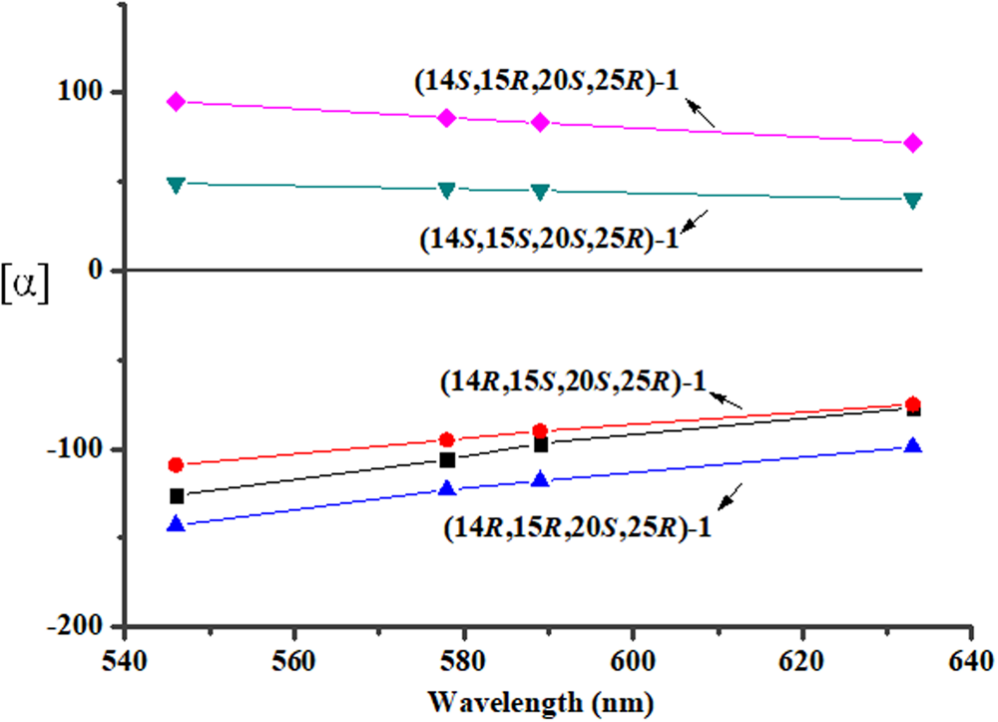
Experimental ORD values of 1 (black) measured at 4 points (549, 578, 589, and 633 nm) compared with the computed ORD values of its four diastereomers.

Then, the ORD spectra of **1** was recorded at four wavelengths (549, 578, 589, and 633 nm) in CH_3_OH to acquire the ORD curve of **1**, which was compared with ORD curves calculated for the remaining four diastereoisomers of (14*R*, 15*R*, 20*S*, 25*R*)-**1**, (14*R*, 15*S*, 20*S*, 25*R*)-**1**, (14*S*, 15*R*, 20*S*, 25*R*)-**1** and (14*S*, 15*S*, 20*S*, 25*R*)-**1**. The ORD calculations for the four diastereomers were performed at the B3LYP/6-311 + G(2d,p) level in a CH_3_OH implicit CPCM solvation model^19–21^. As shown in Fig. 4, the calculated ORD curve for (14*R*, 15*R*, 20*S*, 25*R*)-**1** and (14*R*, 15*S*, 20*S*, 25*R*)-**1** fitted well with experimental ORD data, which in accordance with the expectation, showed negative signals increasing with the increasing wavelength. While, the ORD calculation for (14*S*, 15*R*, 20*S*, 25*R*)-**1** and (14*S*, 15*S*, 20*S*, 25*R*)-**1** provided positive values, which were opposite to experimental ORD data. The above results suggested that these ORD data could be used to exclude (14*S*, 15*R*, 20*S*, 25*R*)-**1** and (14*S*, 15*S*, 20*S*, 25*R*)-**1** diastereomers.

**Figure 5:**
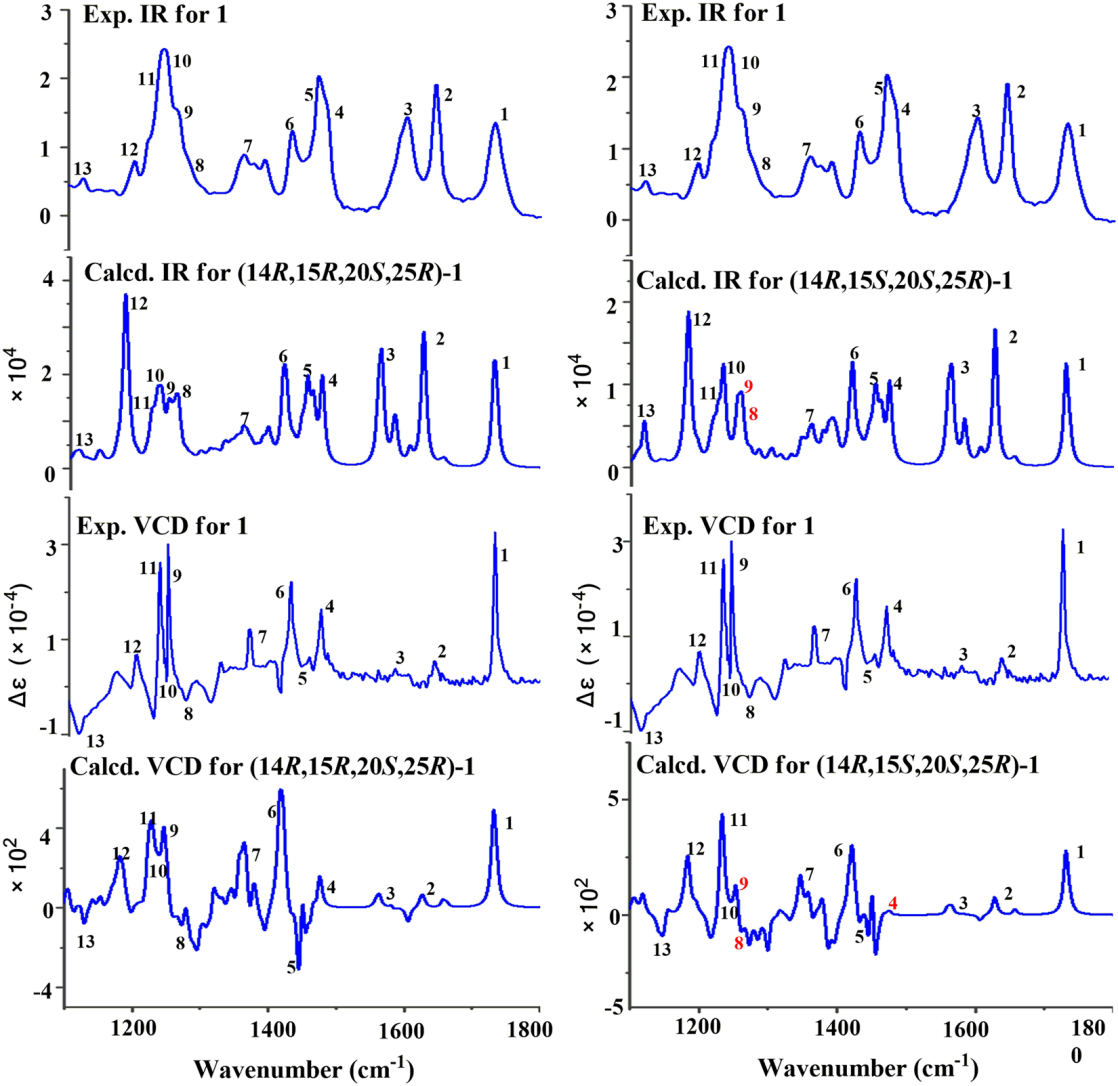
Comparison of the calculated VCD/IR spectra of (14*R*, 15*R*, 20*S*, 25*R*)-**1** and (14*R*, 15*S*, 20*S*, 25*R*)-**1** and the experimental VCD/IR spectra of 1.

**Figure 6:**
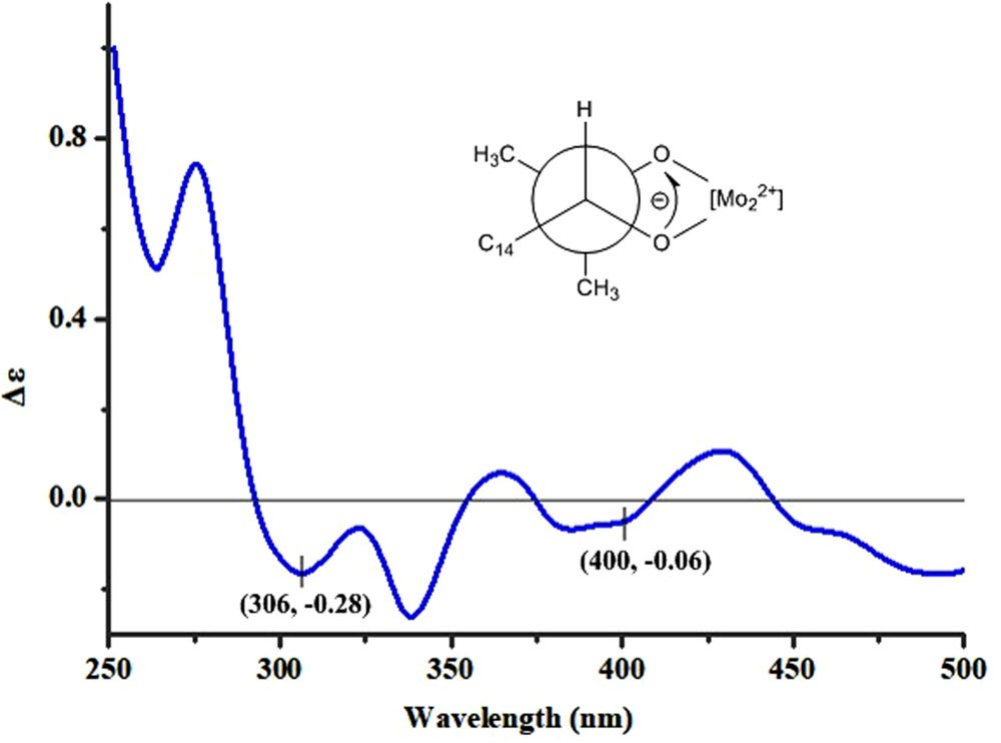
ICD spectrum of Mo-complexes of 1 recorded in DMSO.

Recently, VCD approach has become robust and reliable alternative for the stereochemical characterizations of natural products, especially in conditions not accessible to the other methods^22–24^. The experimental IR and VCD spectra of **1** (12.0 mg) were measured in 120 *μ*L of DMSO-*d*_6_ using a BioTools dual PEM Chiral*IR*-2X spectrophotometer. The IR and VCD frequencies of (14*R*, 15*R*, 20*S*, 25*R*)-**1** and (14*R*, 15*S*, 20*S*, 25*R*)-**1** were calculated at the gas-phase PBEPBE/6-311 + G(d)//PBEPBE/6-311 + G(d) level to compare the experimental IR and VCD data of **1**. All most of the calculated VCD signals of (14*R*, 15*R*, 20*S*, 25*R*)-**1** had agreements with the experimental VCD signals of **1**, while the signals of 4, 8 and 9 in the calculated VCD spectrum of (14*R*, 15*S*, 20*S*, 25*R*)-**1** had disagreements with the corresponding signals in the experimental VCD spectrum of **1** (Fig. 5), suggesting that the structure of (14*R*, 15*R*,20*S*,25*R*)-**1** was closer to the real structure of **1**. In order to further verify the absolute configuration of **1**, the dimolybdenum tetraacetate [Mo_2_(AcO)_4_] induced circular dichroism (ICD) procedure (Snatzke′s method) was used. The negative ICD Cotton effects around 300 and 400 nm of **1** (Fig. 6) gave the newman form of Mo-complexes of **1**. It was found that a counterclockwise rotation, suggesting the *R* configuration for C-15 in **1**. Therefore, on the basis of the above ECD, ORD, VCD, and Snatzke′s results, the absolute configuration of **1** could be defined as 14*R*, 15*R*, 20*S*, 25*R*, unambiguously.

It was showed that the ECD spectra of **2**–**8** closely matched that of **1** (Figures S1 and S2), suggesting the 20*S*, 25*R* absolute configuration for **2**–**3** and **5**–**8**, and 20*R*, 25*R* for **4**. Due to the sample quantity limitation of **2**–**8**, it was difficult to elucidate the absolute configurations of C-14 and C-15 in **2**–**8** by VCD method, directly. Their absolute configurations could be tentatively assigned on the basis of a shared biogenesis with the co-isolated **1**, whose absolute configuration had been unambiguously established firstly. The absolute configurations could be proposed as 14*R*, 15*S*, 20*S*, 25*R* for **2**, **3**, **5** and **6**, 14*R*, 15*S*, 20*R*, 25*R* for **4**, and 14*R*, 15*R*, 20*S*, 25*R* for **7** and **8**.

Xanthones, which were isolated from many different species within fungi, bacteria, and higher plants are widespread classes of typically polysubstituted dibenzo-*γ*-pyrone derivatives^25,26^. Among them, prenylxanthones (**1**–**9**) represent the family of naturally occurring xanthones with C-4 terpenoid-derived side chain^25^. In the previous literature, twenty prenylxanthones have been mainly obtained from the genus *Aspergillus*/*Emericella* fungi, such as 14-hydroxyltajixanthone hydrate^3^, emerixanthones A–D^4^, ruguloxanthones A–C^27^, and so on. The configurations of C-14 and C-15 in prenylxanthone derivatives were challenging to be assigned due to the high conformational flexibility of the terpenoid-derived side chains. It was un-accommodated to determine the relative configuration of C-14 and C-15 by comparison of the coupling constants (*J*_14,15_) as the free rotation of the flexible side chain^5^. Also, it was problem to define the absolute configuration of C-15 by comparison of the optical rotatory^27^. This work demonstrated that using multiple chiroptical methods in combination with DFT calculations allowed one to determine absolute configurations with high confidence for chiral natural products, which possessed rotatable bonds.

Compounds **1**–**9** were subjected to test their cytotoxic activities by MTT method against human breast cancer (MDA-MB-231 and MCF-7), human gastric cancer (MGC-803), cervical cancer (HeLa), and human lung epithelial carcinoma (A-549) cell lines. Compound **1** displayed selective cytotoxicity against the A-549 cell line with the IC_50_ value of 1.8 *μ*M. Compounds **3** and **6** showed broad-spectrum cytotoxicities against five tumor cell lines with the IC_50_ values ranging from 1.1 to 9.8 *μ*M (Table 4). However, the other compounds (**2**, **4**, **5**, **7**, **8**, **9**) exhibited very low cytotoxicity to any of the above cell lines (IC_50_ > 10.0 *μ*M).

**Table 4:** Cytotoxicity of compounds **1–9**.

Compounds	Cell lines IC_50_ (*μ*M)				
	MDA-MB-231	MCF-7	MGC-803	HeLa	A-549
**1**	>10.0	>10.0	>10.0	>10.0	1.8
**3**	3.3	2.8	3.6	2.9	3.2
**6**	9.8	2.7	3.6	1.7	1.1
**2, 4, 5, 7, 8, 9**	>10.0	>10.0	>10.0	>10.0	>10.0
**Cisplatin**	1.3	0.97	1.1	0.82	0.74

Compounds **1**–**9** were further tested their antibacterial activities against a panel of pathogenic bacteria, including *Micrococcus lysodeikticus*, *Bacillus anthraci*, *Salmonella typhi*, and *Enterobacter aerogenes*. Only **7** and **8** showed antibacterial activity against *M*. *lysodeikticus*, *B*. *anthraci*, *S*. *typhi*, and *E*. *aerogenes*, with MIC values of 0.78, 12.5, 6.13 and 6.13 *μ*g/mL for **7**, and 6.13, 12.5, 6.13 and 6.13 *μ*g/mL for **8**, respectively. These data indicated that the antibacterial activities may be due to the double bonds between C-16 and C-17 in **7** and **8**. In addition, ciprofloxacin showed antibacterial activity against *M*. *lysodeikticus*, *B*. *anthraci*, *S*. *typhi*, and *E*. *aerogenes*, with MIC values of 0.19, 1.56, 3.13, 1.56 *μ*g/mL, respectively.

In summary, three chiroptical methods, including ECD, ORD and VCD, combined with quantum theory calculation were carried out to elucidate the absolute configuration of the prenylxanthone **1**, which was difficult to be determined by single method due to high flexibility of the molecule. The interesting chemical structures and potent biological activities of these prenylxanthones (**1**–**9**) may encourage further investigations on this cluster of metabolites for drug discovery.

## Methods

### General Experimental Procedures

Optical rotatory dispersions were acquired using a JASCO P-2000 spectrometer. Optical rotations were obtained on an Optical Activity AA-55 series polarimeter. UV data were performed on a Perkin-Elmer model 241 spectrophotometer in MeOH. Electronic circular dichroism spectra were measured using a JASCO J-715 circular dichroism spectrometer. IR spectra were determined using KBr pellets with a Nicolet NEXUS 470 spectrophotometer. Vibrational circular dichroism spectra were taken on a BioTools Chiral*IR*-2X spectrophotometer. 1D and 2D NMR data (600 MHz for ^1^H and 150 MHz for ^13^C) were acquired on Bruker Avance-III 600 MHz NMR spectrometer with TMS as an internal standard. High-resolution mass data were obtained from a Thermo Scientific LTQ Orbitrap XL spectrometer. HPLC analysis and semi-preparation was performed on a Shimadzu LC-20AT system with a SPD-M20A photodiode array detector, using a Waters RP-18 (XBridge OBD, 5 *μ*m, 10 × 250 mm) and a Waters normal phase (Viridis^TM^ Silica 2-Ethylpyridine, 5 *μ*m, 10 × 250 mm) columns. Column chromatography was performed on Silica gel 200–300 mesh (Qingdao Marine Chemical Factory), and Sephadex LH-20 (18–110 *μ*m, Pharmacia Fine Chemical Co., Ltd., Sweden).

### Fungal Material

The fungus *Aspergillus* sp. ZA-01 was obtained from sediment, collected from the Bohai Sea of Huanghuagang, Hebei Province of China, in June 2016. The strain was identified according to its 16*S* rRNA amplification and sequencing of the ITS region. The strain was deposited in College of Pharmaceutical Sciences, Key Laboratory of Medicinal Chemistry and Molecular Diagnostics of Education Ministry of China, Hebei University, Baoding, China.

### Extraction and Purification

The fermentation of the fungus Aspergillus sp. ZA-01 was carried out using solid culture in eighty erlenmeyer flasks (each erlenmeyer flask containing rice 100 g, water 100 mL, NaNO_3_ 0.3 g, KH_2_PO_4_ 0.1 g, MgSO_4_·7H_2_O 0.05 g, NaCl 0.05 g, FeSO_4_ 0.01 g, sucrose 3.0 g, pH adjusted to 7.3) at room temperature. After 45 days, the fermented solid medium was repeatedly extracted with a CH2Cl2/MeOH (1:1) mixture (eight times) afforded the crude extract (150.0 g). Then, the extract was partitioned between EtOAc and H_2_O to give the EtOAc extract (60.0 g). The EtOAc extract was then subjected to silica gel column chromatography (CC) [10 × 20 cm, stepwise gradient, petroleum ether (PE)-EtOAc to offer six fractions: Fr.1-Fr.6. Fr.2 (7.5 g) was applied to silica gel CC (PE:EtOAc = 3:1), followed by Sephadex LH-20 (CH2Cl2:MeOH = 1:1) to obtain three subfractions: Fr.3-1-Fr.3-3. Fr.3-3 was further purified by preparative HPLC by a Waters RP-18 column at a flow rate of 2.0 mL/min (MeOH/H2O, 75:25) and a Waters normal phase column at a flow rate of 2.0 mL/min (PE/EtOH, 85:15) to give 1 (15.0 mg), 2 (4.5 mg), 3 (5.0 mg), 4 (5.2 mg), 5 (5.0 mg), 6 (4.5 mg), 7 (5.0 mg), 8 (4.7 mg) and 9 (2.5 mg).

*Aspergixanthone A (****1****):* yellow, amorphous powder; [*α*]_D_^20^ = −97 (*c* 0.1, MeOH); UV (MeOH) λ_max_ (log *ε*) 231 (4.7), 241 (4.3), 265 (4.9), 286 (2.0), 383 (1.7) nm; CD (MeOH) λ_max_ (Δ*ε*) 219 (16.3), 241 (−9.4), 271 (−8.1), 289 (1.4), 319 (1.0), 332 (−3.2) nm; IR (KBr) *ν*_max_ 3445, 2929, 2355, 1635, 1591, 1475, 1246, 1065, 895 cm^−1^; NMR data, see Tables 1 and 2; HRESIMS *m/z* 535.1935 [M + Na]^+^, (calcd. for C_28_H_32_O_9_Na, 535.1939).

*Aspergixanthone B (****2****):* yellow, amorphous powder; [*α*]_D_^20^ = −120 (*c* 0.1, MeOH); UV (MeOH) λ_max_ (log *ε*) 232 (4.6), 245 (4.3), 267 (4.8), 284 (2.0), 385 (1.8) nm; CD (MeOH) λ_max_ (Δ*ε*) 219 (3.3), 242 (−15.1), 267 (−10.5), 285 (0.2), 318 (−1.8), 330 (−6.6) nm; IR (KBr) *ν*_max_ 3442, 2929, 2358, 1639, 1593, 1471, 1242, 1072, 894 cm^−1^; NMR data, see Tables 1 and 2; HRESIMS *m/z* 493.1839 [M + Na]^+^, (calcd. for C_26_H_30_O_9_Na, 493.1833).

*Aspergixanthone C (****3****):* yellow, amorphous powder; [*α*]_D_^20^ = −107 (*c* 0.1, MeOH); UV (MeOH) λ_max_ (log *ε*) 235 (4.7), 242 (4.5), 269 (4.9), 286 (1.9), 384 (1.7) nm; CD (MeOH) λ_max_ (Δ*ε*) 220 (16.9), 238 (−12.7), 271 (−12.7), 289 (0.8), 318 (0.6), 332 (−4.6) nm; IR (KBr) *ν*_max_ 3419, 2966, 2358, 1737, 1733, 1473, 1238, 1020, 829 cm^−1^; NMR data, see Tables 1 and 2; HRESIMS *m/z* 521.1784 [M + Na]^+^, (calcd. for C_27_H_30_O_9_Na, 521.1782).

*Aspergixanthone D (****4****):* yellow, amorphous powder; [*α*]_D_^20^ = +39.0 (*c* 0.1, MeOH); UV (MeOH) λ_max_ (log *ε*) 231 (4.3), 249 (4.1), 262 (4.3), 288 (1.8), 389 (1.6) nm; CD (MeOH) λ_max_ (Δ*ε*) 217 (−3.7), 243 (−19.2), 270 (−9.2), 286 (0.1), 316 (−3.6), 332 (−7.9) nm; IR (KBr) *ν*_max_ 3421, 2964, 2356, 1726, 1718, 1475, 1236, 1016, 835 cm^−1^; NMR data, see Tables 1 and 2; HRESIMS *m/z* 457.1857 [M + H]^+^, (calcd. for C_25_H_29_O_8_, 457.1857).

*Aspergixanthone E (****5****):* yellow, amorphous powder; [*α*]_D_^20^ = −92 (*c* 0.1, MeOH); UV (MeOH) λ_max_ (log *ε*) 230 (4.7), 245 (4.5), 266 (4.7), 285 (1.9), 384 (1.8) nm; CD (MeOH) λ_max_ (Δ*ε*) 221 (7.1), 240 (−18.5), 270 (−13.5), 286 (−1.0), 314 (−1.1), 331 (−1.8) nm; IR (KBr) *ν*_max_ 3432, 2926, 2357, 1662, 1587, 1465, 1236, 1064, 847 cm^−1^; NMR data, see Tables 2 and 3; HRESIMS *m/z* 577.2048 [M + Na]^+^, (calcd. for C_30_H_34_O_10_Na, 577.2044).

*Aspergixanthone F (****6****):* yellow, amorphous powder; [*α*]_D_^20^ = −73 (*c* 0.1, MeOH); UV (MeOH) λ_max_ (log *ε*) 232 (4.7), 246 (4.5), 269 (4.7), 285 (1.8), 386 (1.8) nm; CD (MeOH) λ_max_ (Δ*ε*) 223 (−10.5), 240 (−33.1), 267 (−19.1), 280 (−2.1), 315 (−2.0), 331 (−4.1) nm; IR (KBr) *ν*_max_ 3434, 2929, 2354, 1665, 1587, 1466, 1232, 1069, 852 cm^−1^; NMR data, see Tables 2 and 3; HRESIMS *m/z* 535.1939 [M + Na]^+^, (calcd. for C_28_H_32_O_9_Na, 535.1939).

*Aspergixanthone G (****7****):* yellow, amorphous powder; [*α*]_D_^20^ = −56 (*c* 0.1, MeOH); UV (MeOH) λ_max_ (log *ε*) 237 (4.6), 242 (4.4), 271 (4.6), 285 (1.8), 385 (1.8) nm; CD (MeOH) λ_max_ (Δ*ε*) 222 (13.1), 235 (−11.7), 271 (−6.2), 283 (2.9), 314 (0.1), 332 (−4.0) nm; IR (KBr) *ν*_max_ 3446, 2972, 2368, 1733, 1645, 1471, 1249, 1029, 831 cm^−1^; NMR data, see Tables 2 and 3; HRESIMS *m/z* 503.1667 [M + Na]^+^, (calcd. for C_27_H_28_O_8_Na, 503.1676).

*Aspergixanthone H (****8****):* yellow, amorphous powder; [*α*]_D_^20^ = −113 (*c* 0.1, MeOH); UV (MeOH) λ_max_ (log *ε*) 234 (4.8), 246 (4.5), 271 (4.7), 286 (1.8), 389 (1.8) nm; CD (MeOH) λ_max_ (Δ*ε*) 222 (−3.1), 240 (−10.7), 275 (1.2), 283 (1.5), 316 (−2.6), 331 (−4.7) nm; IR (KBr) *ν*_max_ 3431, 2972, 2352, 1733, 1637, 1471, 1249, 1054, 817 cm^−1^; NMR data, see Tables 2 and 3; HRESIMS *m/z* 461.1569 [M + Na]^+^, (calcd. for C_25_H_26_O_7_Na, 461.1571).

### Computational section

The four possibly diastereomers of **1** [(14*R*, 15*R*, 20*S*, 25*R*)-**1**, (14*R*, 15*S*, 20*S*, 25*R*)-**1**, (14*S*, 15*R*, 20*S*, 25*R*)-**1** and (14*S*, 15*S*, 20*S*, 25*R*)-**1**] were constructed and used for conformational searches using MMFF94S force field by the BARISTA software (CONFLEX Corporation). Totally 63 stable conformers for (14*R*, 15*R*, 20*S*, 25*R*)-**1** with relative energy within a 10.0 kcal/mol energy window, 56 conformers for (14*R*, 15*S*, 20*S*, 25*R*)-**1**, 70 conformers for (14*S*, 15*R*, 20*S*, 25*R*)-**1** and 75 conformers for (14*S*, 15*S*, 20*S*, 25*R*)-**1**, were recorded, respectively. These corresponding minimum geometries were optimized at the gas-phase B3LYP/6-31 G(d) level using Gaussian 09 package. The re-optimizations were performed at the gas-phase B3LYP/6-311 + G(d) level for four possibly diastereomers of **1** with relative energy less than 4.6 kcal/mol (ten conformers for (14*R*, 15*R*, 20*S*, 25*R*)-**1**, two conformers for (14*R*, 15*S*, 20*S*, 25*R*)-**1**, three conformers for (14*S*, 15*R*, 20*S*, 25*R*)-**1**, and eight conformers for (14*S*, 15*S*, 20*S*, 25*R*)-**1**], respectively). Time-dependent density functional theory (TD-DFT) at the set of gas-phase B3LYP/6-311 ++ G(2d,p)//B3LYP/6-311 + G(d) level was used for ECD calculations with total of 60 excited states for the four diastereomers. ORD calculations were performed at the B3LYP/6-311 ++ G(2d,p)//B3LYP/6-311 + G(d) level. VCD predictions were carried out at the gas-phase B3LYP/6-311 + G(d)//B3LYP/6-311 + G(d) level. Boltzmann statistics were used for all simulations of ECD, ORD and VCD.

### Snatzke′s method

The ICD spectrum of **1** by adding Mo_2_(OAc)_4_ was measured according to the referenced procedure^28^.

### Cytotoxicity Assays

The cytotoxicities against human breast cancer (MDA-MB-231 and MCF-7), human gastric cancer (MGC-803), cervical cancer (HeLa), and human lung epithelial carcinoma (A-549) cell lines were evaluated using the MTT method^29^. Cisplatin was used as a positive control.

### Antibacterial Assays

Antibacterial activity was evaluated by the conventional broth dilution assay^30^. Four pathogenic bacterial strains, *Micrococcus lysodeikticus*, *Bacillus anthraci*, *Salmonella typhi*, and *Enterobacter aerogenes* were used, and ciprofloxacin was used as a positive control.

## Electronic supplementary material

Supporting Information
