# Supplementary material for: Absolute Configurations of 14,15-Hydroxylated Prenylxanthones from a Marine-Derived Aspergillus sp. Fungus by Chiroptical Methods
Source: Sci Rep. 2018 Jul 13;8:10621. doi: 10.1038/s41598-018-28996-5 (PMC6045578; doi:10.1038/s41598-018-28996-5)
Supplement: Supplementary file 1 — Supporting Information [file 41598_2018_28996_MOESM1_ESM.doc]

**Absolute Configurations of 14,15-Hydroxylated Prenylxanthones from a Marine-Derived *Aspergillus* sp. Fungus by Chiroptical Methods**

Ao Zhu,^1^ Meng-Yue Yang,^1^ Ya-Hui Zhang,^2^ Chang-Lun Shao,^2^ Chang-Yun Wang,^2^ Lian-Dong Hu,^1^ Fei Cao,^1*^ and Hua-Jie Zhu^1*^

^1^College of Pharmaceutical Sciences, Key Laboratory of Pharmaceutical Quality Control of Hebei Province, Key Laboratory of Medicinal Chemistry and Molecular Diagnostics of Education Ministry of China, Hebei University, Baoding 071002, People’s Republic of China.

^2^Key Laboratory of Marine Drugs, The Ministry of Education of China, School of Medicine and Pharmacy, Ocean University of China, Qingdao 266003, People’s Republic of China

*Correspondence and requests for materials should be addressed to F. C. (caofei542927001@163.com); H. -J. Z. (hjzhu2017@163.com)

**List of Supporting Information**

**Table S1.** 1D NMR and 2D NMR correlations data for compound **1**

**Table S2.** 1D NMR and 2D NMR correlations data for compound **2**

**Table S3.** 1D NMR and 2D NMR correlations data for compound **3**

**Table S4.** 1D NMR and 2D NMR correlations data for compound **4**

**Table S5.** 1D NMR and 2D NMR correlations data for compound **5**

**Table S6.** 1D NMR and 2D NMR correlations data for compound **6**

**Table S7.** 1D NMR and 2D NMR correlations data for compound **7**

**Table S8.** 1D NMR and 2D NMR correlations data for compound **8**

**Figure S1.** Experimental ECD spectra of **1**−**4**

**Figure S2.** Experimental ECD spectra of **5**−**8**

**Figure S3.** ^1^H NMR (600 MHz, CDCl_3_) spectrum of compound **1**

**Figure S4.** ^13^C NMR (150 MHz, CDCl_3_) spectrum of compound **1**

**Figure S5.** HSQC (CDCl_3_) spectrum of compound **1**

**Figure S6.** ^1^H-^1^H COSY (CDCl_3_) spectrum of compound **1**

**Figure S7.** HMBC (CDCl_3_) spectrum of compound **1**

**Figure S8.** NOESY (CDCl_3_) spectrum of compound **1**

**Figure S9.** HRESIMS spectrum of compound **1**

**Figure S10.** ^1^H NMR (600 MHz, CDCl_3_) spectrum of compound **2**

**Figure S11.** ^13^C NMR (150 MHz, CDCl_3_) spectrum of compound **2**

**Figure S12.** HSQC (CDCl_3_) spectrum of compound **2**

**Figure S13.** ^1^H-^1^H COSY (CDCl_3_) spectrum of compound **2**

**Figure S13.** ^1^H-^1^H COSY (CDCl_3_) spectrum of compound **2**

**Figure S15.** NOESY (CDCl_3_) spectrum of compound **2**

**Figure S16.** HRESIMS spectrum of compound **2**

**Figure S17.** ^1^H NMR (600 MHz, DMSO-*d*_6_) spectrum of compound **3**

**Figure S18.** ^13^C NMR (150 MHz, DMSO-*d*_6_) spectrum of compound **3**

**Figure S19.** HSQC (DMSO-*d*_6_) spectrum of compound **3**

**Figure S20.** ^1^H-^1^H COSY (DMSO-*d*_6_) spectrum of compound **3**

**Figure S21.** HMBC (DMSO-*d*_6_) spectrum of compound **3**

**Figure S22.** NOESY (DMSO-*d*_6_) spectrum of compound **3**

**Figure S23.** HRESIMS spectrum of compound **3**

**Figure S24.** ^1^H NMR (600 MHz, DMSO-*d*_6_) spectrum of compound **4**

**Figure S25.** ^13^C NMR (150 MHz, DMSO-*d*_6_) spectrum of compound **4**

**Figure S26.** HSQC (DMSO-*d*_6_) spectrum of compound **4**

**Figure S27.** ^1^H-^1^H COSY (DMSO-*d*_6_) spectrum of compound **4**

**Figure S28.** HMBC (DMSO-*d*_6_) spectrum of compound **4**

**Figure S29.** NOESY (DMSO-*d*_6_) spectrum of compound **4**

**Figure S30.** HRESIMS spectrum of compound **4**

**Figure S31.** ^1^H NMR (600 MHz, CDCl_3_) spectrum of compound **5**

**Figure S32.** ^13^C NMR (150 MHz, CDCl_3_) spectrum of compound **5**

**Figure S33.** HSQC (CDCl_3_) spectrum of compound **5**

**Figure S34.** ^1^H-^1^H COSY (CDCl_3_) spectrum of compound **5**

**Figure S35.** HMBC (CDCl_3_) spectrum of compound **5**

**Figure S36.** NOESY (CDCl_3_) spectrum of compound **5**

**Figure S37.** HRESIMS spectrum of compound **5**

**Figure S38.** ^1^H NMR (600 MHz, CDCl_3_) spectrum of compound **6**

**Figure S39.** ^13^C NMR (150 MHz, CDCl_3_) spectrum of compound **6**

**Figure S40.** HSQC (CDCl_3_) spectrum of compound **6**

**Figure S41.** ^1^H-^1^H COSY (CDCl_3_) spectrum of compound **6**

**Figure S42.** HMBC (CDCl_3_) spectrum of compound **6**

**Figure S43.** NOESY (CDCl_3_) spectrum of compound **6**

**Figure S44.** HRESIMS spectrum of compound **6**

**Figure S45.** ^1^H NMR (600 MHz, CDCl_3_) spectrum of compound **7**

**Figure S46.** ^13^C NMR (150 MHz, CDCl_3_) spectrum of compound **7**

**Figure S47.** HSQC (CDCl_3_) spectrum of compound **7**

**Figure S48.** ^1^H-^1^H COSY (CDCl_3_) spectrum of compound **7**

**Figure S49.** HMBC (CDCl_3_) spectrum of compound **7**

**Figure S50.** NOESY (CDCl_3_) spectrum of compound **7**

**Figure S51.** HRESIMS spectrum of compound **7**

**Figure S52.** ^1^H NMR (600 MHz, CDCl_3_) spectrum of compound **8**

**Figure S53.** ^13^C NMR (150 MHz, CDCl_3_) spectrum of compound **8**

**Figure S54.** HSQC (CDCl_3_) spectrum of compound **8**

**Figure S55.** ^1^H-^1^H COSY (CDCl_3_) spectrum of compound **8**

**Figure S56.** HMBC (CDCl_3_) spectrum of compound **8**

**Figure S57.** NOESY (CDCl_3_) spectrum of compound **8**

**Figure S58.** HRESIMS spectrum of compound **8**

Lowest energy conformers for ECD, ORD and VCD calculation

**Table S1. 1D NMR and 2D NMR correlations data for compound 1**

| **No.** | **δ_H_ (J in Hz)** | **δ_C_, type** | **Key HMBC** | **^1^H-^1^H COSY** |
| --- | --- | --- | --- | --- |
| 1 |  | 161.7, C |  |  |
| 2 | 6.82, d (8.4) | 110.4, CH | C-1, C-4, C-9, | H-3 |
| 3 | 7.66, d (8.4) | 135.3, CH | C-2, C-4 | H-2 |
| 4 |  | 115.0, C |  |  |
| 5 | 7.24, s | 120.2, CH | C-11, C-12, C-6, C-7 |  |
| 6 |  | 137.6, C |  |  |
| 7 |  | 150.2, C |  |  |
| 8 |  | 115.2, C |  |  |
| 9 |  | 109.0, C |  |  |
| 10 |  | 152.2, C |  |  |
| 11 |  | 151.5, C |  |  |
| 12 |  | 116.3, C |  |  |
| 13 |  | 183.0, C |  |  |
| 14 | 5.10, brs | 76.2, CH | C-3, C-4, C-10, C-15 | H-15, 15-OH |
| 15 | 3.45, brs | 78.3, CH |  | H-14, 15-OH |
| 16 |  | 72.9, C |  |  |
| 17 | 1.25, s | 26.4, CH_3_ | C-15, C-16, C-18 |  |
| 18 | 1.40, s | 26.5, CH_3_ | C-15, C-16, C-17 |  |
| 19 | 4.54, brd (10.8)  4.31, dd (10.8,2.4) | 63.8, CH_2_ | C-7, C-21 | H-20, H-25 |
| 20 | 2.71, brs | 42.5, CH | C-8, C-23 | H-19, H-25 |
| 21 |  | 141.5, C |  |  |
| 22 | 4.80, s  4.75, s | 112.7, CH_2_ | C-20, C-23 |  |
| 23 | 1.88, s | 22.3, CH_3_ | C-20, C-22 |  |
| 24 | 2.35, s | 17.2, CH_3_ | C-5, C-7 |  |
| 25 | 6.89, brs | 65.4, CH | C-12, 25-OAc | H-20 |
| 1-OH | 12.97, brs |  | C-9 |  |
| 15-OH | 2.73, brs |  |  | H-14 |
| 14-OCH_3_ | 3.28, s | 56.6, CH_3_ | C-14 |  |
| 25-OAc | 2.07, s | 170.0, C  21.2, CH_3_ |  |  |

**Table S2. 1D NMR and 2D NMR correlations data for compound 2**

| **No.** | **δ_H_ (J in Hz)** | **δ_C_, type** | **Key HMBC** | **^1^H-^1^H COSY** |
| --- | --- | --- | --- | --- |
| 1 |  | 161.5, C |  |  |
| 2 | 6.86, d (8.4) | 110.5, CH | C-1, C-4, C-9 | H-3 |
| 3 | 7.72, d (8.4) | 135.9, CH | C-1, C-4, C-10 | H-2 |
| 4 |  | 115.5, C |  |  |
| 5 | 7.21, s | 119.0, C | C-11, C-7, C-12 |  |
| 6 |  | 138.6, C |  |  |
| 7 |  | 149.6, C |  |  |
| 8 |  | 121.2, C |  |  |
| 9 |  | 108.9, C |  |  |
| 10 |  | 152.4, C |  |  |
| 11 |  | 151.7, C |  |  |
| 12 |  | 116.9, C |  |  |
| 13 |  | 184.2, C |  |  |
| 14 | 5.13, d (2.4) | 76.2, CH | C-3, C-4, C-10, C-15 | H-15 |
| 15 | 3.46, brs | 78.2, CH |  | H-14 |
| 16 |  | 72.9, C |  |  |
| 17 | 1.25, s | 26.5, CH_3_ | C-15, C-16, C-18 |  |
| 18 | 1.42, s | 26.5, CH_3_ | C-15, C-16, C-17 |  |
| 19 | 4.43, brd (10.8)  4.35, dd (10.8, 2.4) | 64.5, CH_2_ | C-7, C-21 | H-20 |
| 20 | 2.72, brs | 44.8, CH | C-8, C-22, C-23 | H-19 |
| 21 |  | 142.5, C |  |  |
| 22 | 4.78, s  4.56, s | 112.2, CH_2_ | C-20, C-23 |  |
| 23 | 1.84, s | 22.5, CH_3_ | C-20, C-22 |  |
| 24 | 2.35, s | 17.3, CH_3_ | C-5, C-7 |  |
| 25 | 5.40, brs | 63.1, CH | C-7, C-12 | H-20 |
| 1-OH | 12.71, brs |  | C-9 |  |
| 14-OCH_3_ | 3.29, s | 56.6, CH_3_ | C-14 |  |
| 15-OH | 2.95, brs |  |  |  |
| 25-OH | 4.94, d (2.4) |  |  |  |

**Table S3. 1D NMR and 2D NMR correlations data for compound 3**

| **No.** | **δ_H_ (J in Hz)** | **δ_C_, type** | **Key HMBC** | **^1^H-^1^H COSY** |
| --- | --- | --- | --- | --- |
| 1 |  | 159.6, C |  |  |
| 2 | 6.77, d (8.4) | 109.2, CH | C-1, C-4, C-9 | H-3 |
| 3 | 7.82, d (8.4) | 135.8, CH | C-1, C-4, C-10 | H-2 |
| 4 |  | 122.3, C |  |  |
| 5 | 7.50, s | 120.7, CH | C-11, C-7, C-12 |  |
| 6 |  | 137.6, C |  |  |
| 7 |  | 149.9, C |  |  |
| 8 |  | 114.7, C |  |  |
| 9 |  | 108.1, C |  |  |
| 10 |  | 151.4, C |  |  |
| 11 |  | 150.5, C |  |  |
| 12 |  | 115.5, C |  |  |
| 13 |  | 183.1, C |  |  |
| 14 | 5.47, d (2.8) | 65.3, CH | C-3, C-4, C-10, C-15 | H-15 |
| 15 | 3.27, brd (7.2) | 78.0, CH |  | H-14 |
| 16 |  | 72.7, C |  |  |
| 17 | 1.21, s | 26.2, CH_3_ | C-15, C-16, C-18 |  |
| 18 | 1.28, s | 27.5, CH_3_ | C-15, C-16, C-17 |  |
| 19 | 4.56, brd (11.4) | 63.5, CH_2_ | C-7, C-21 | H-20 |
|  | 4.20, dd (11.4, 2.4) |
| 20 | 2.68, brs | 41.7, CH | C-8, C-22 | H-19 |
| 21 |  | 141.8, C |  |  |
| 22 | 4.79, s  4.61, s | 112.6, CH_2_ | C-20, C-23 |  |
| 23 | 1.81, s | 22.1, CH_3_ | C-20, C-22 |  |
| 24 | 2.31, s | 16.9, CH_3_ | C-5, C-7 |  |
| 25 | 6.81, brs | 64.8, CH | C-12, 25-OAc | H-20 |
| 1-OH | 12.78, brs |  |  |  |
| 14-OH | 5.19, d (5.4) |  |  | H-15 |
| 15-OH | 4.44, brd (7.2) |  |  | H-14 |
| 16-OH | 4.55, brs |  |  |  |
| 25-OAc | 1.99, s | 169.3, C  21.0, CH_3_ |  |  |

**Table S4. 1D NMR and 2D NMR correlations data for compound 4**

| **No.** | **δ_H_ (J in Hz)** | **δ_C_, type** | **Key HMBC** | **^1^H-^1^H COSY** |
| --- | --- | --- | --- | --- |
| 1 |  | 159.6, C |  |  |
| 2 | 6.78, d (8.4) | 109.0, CH | C-1, C-4, C-9 | H-3 |
| 3 | 7.83, d (8.4) | 135.6, CH | C-1, C-4, C-10 | H-2 |
| 4 |  | 122.2, C |  |  |
| 5 | 7.40, s | 119.1, CH | C-11, C-7, C-12 |  |
| 6 |  | 137.2, C |  |  |
| 7 |  | 148.7, C |  |  |
| 8 |  | 121.0, C |  |  |
| 9 |  | 108.1, C |  |  |
| 10 |  | 151.1, C |  |  |
| 11 |  | 150.5, C |  |  |
| 12 |  | 115.8, C |  |  |
| 13 |  | 183.4, C |  |  |
| 14 | 5.48, brs | 65.2, CH | C-3, C-4, C-10, C-15 | H-15 |
| 15 | 3.27, brd (6.6) | 78.0, CH |  | H-14 |
| 16 |  | 72.7, C |  |  |
| 17 | 1.22, s | 26.2, CH_3_ | C-15, C-16, C-18 |  |
| 18 | 1.29, s | 27.5, CH_3_ | C-15, C-16, C-17 |  |
| 19 | 4.46, brd (11.4) | 63.5, CH_2_ | C-7, C-21 | H-20 |
|  | 4.34, dd (11.4, 2.4) |
| 20 | 2.51, brs | 44.4, CH | C-8, C-22 | H-19 |
| 21 |  | 142.8, C |  |  |
| 22 | 4.74, s | 111.9, CH_2_ | C-20, C-23 |  |
|  | 4.55, s |  |  |  |
| 23 | 1.78, s | 22.4, CH_3_ | C-20, C-22 |  |
| 24 | 2.29, s | 16.9, CH_3_ | C-5, C-7 |  |
| 25 | 5.81, brs | 60.9, CH | C-7, C-12 | H-20 |
| 1-OH | 12.84, brs |  | C-1, C-9 |  |
| 14-OH | 5.17, d (3.6) |  |  |  |
| 15-OH | 4.46, brd (6.6) |  |  |  |
| 16-OH | 4.54, brs |  |  |  |
| 25-OH | 5.26, d (3.6) |  |  |  |

**Table S5. 1D NMR and 2D NMR correlations data for compound 5**

| **No.** | **δ_H_ (J in Hz)** | **δ_C_, type** | **Key HMBC** | **^1^H-^1^H COSY** |
| --- | --- | --- | --- | --- |
| 1 |  | 162.0, C |  |  |
| 2 | 6.77, d (8.4) | 110.0, CH | C-1, C-4, C-9 | H-3 |
| 3 | 7.59, d (8.4) | 134.1, CH | C-1, C-4, C-10 | H-2 |
| 4 |  | 113.4, C |  |  |
| 5 | 7.24, s | 120.3, CH | C-11, C-7, C-12 |  |
| 6 |  | 137.6, C |  |  |
| 7 |  | 150.3, C |  |  |
| 8 |  | 115.1, C |  |  |
| 9 |  | 109.1, C |  |  |
| 10 |  | 152.2, C |  |  |
| 11 |  | 151.5, C |  |  |
| 12 |  | 116.5, C |  |  |
| 13 |  | 183.0, C |  |  |
| 14 | 5.41, d (1.8) | 76.4, CH | C-3, C-4, C-10, C-15, 14-OAc | H-15 |
| 15 | 5.00, d (2.4) | 77.0, CH | 15-OAc | H-14 |
| 16 |  | 72.7, C |  |  |
| 17 | 1.22, s | 26.9, CH_3_ | C-15, C-16, C-18 |  |
| 18 | 1.56, s | 27.9, CH_3_ | C-15, C-16, C-17 |  |
| 19 | 4.54, brd (10.8)  4.32, dd (10.8, 3.0) | 63.8, CH | C-7, C-21 | H-20 |
| 20 | 2.72, brs | 42.5, CH | C-8, C-22 | H-19 |
| 21 |  | 141.6, C |  |  |
| 22 | 4.80, s  4.74, s | 112.7, CH_2_ | C-20, C-23 |  |
| 23 | 1.88, s | 22.4, CH_3_ | C-20, C-22 |  |
| 24 | 2.36, s | 17.3, CH_3_ | C-5, C-7 |  |
| 25 | 6.88, brs | 65.4, CH | C-12, 25-OAc | H-20 |
| 1-OH | 12.97, brs |  |  |  |
| 14-OCH_3_ | 3.34, s | 57.1, CH_3_ | C-14 |  |
| 15-OAc | 1.92, s | 170.2, C  20.5, CH_3_ | C-15 |  |
| 25-OAc | 2.10, s | 170.1, C |  |  |
|  |  | 21.3, CH_3_ |  |  |

**Table S6. 1D NMR and 2D NMR correlations data for compound 6**

| **No.** | **δ_H_ (J in Hz)** | **δ_C_, type** | **Key HMBC** | **^1^H-^1^H COSY** |
| --- | --- | --- | --- | --- |
| 1 |  | 161.8, C |  |  |
| 2 | 6.82, d (8.4) | 110.1, CH | C-1, C-4, C-9 | H-3 |
| 3 | 7.65, d (8.4) | 134.6, CH | C-1, C-4, C-10 | H-2 |
| 4 |  | 113.8, C |  |  |
| 5 | 7.22, s | 119.1, CH | C-11, C-7, C-12 |  |
| 6 |  | 138.6, C |  |  |
| 7 |  | 149.7, C |  |  |
| 8 |  | 121.2, C |  |  |
| 9 |  | 109.0, C |  |  |
| 10 |  | 152.4, C |  |  |
| 11 |  | 151.7, C |  |  |
| 12 |  | 117.0, C |  |  |
| 13 |  | 184.2, C |  |  |
| 14 | 5.42, d (1.2) | 76.5, CH | C-3, C-4, C-10, C-15, 14-OAc | H-15 |
| 15 | 5.02, d (2.4) | 77.0, CH |  | H-14 |
| 16 |  | 72.7, C |  |  |
| 17 | 1.22, s | 26.9, CH_3_ | C-15, C-16, C-18 |  |
| 18 | 1.56, s | 27.9, CH_3_ | C-15, C-16, C-17 |  |
| 19 | 4.43, dd (10.8,1.8)  4.36, dd (10.8,3.0) | 64.4, CH_2_ | C-7, C-21 | H-20 |
| 20 | 2.73, brs | 44.8, CH | C-8, C-22 | H-19 |
| 21 |  | 142.6, C |  |  |
| 22 | 4.78, s  4.55, s | 112.2, CH_2_ | C-20, C-23 |  |
| 23 | 1.84, s | 22.5, CH_3_ | C-20, C-22 |  |
| 24 | 2.37, s | 17.4, CH_3_ | C-5, C-7 |  |
| 25 | 5.40, brs | 63.0, CH | C-12 | H-20 |
| 1-OH | 12.74, brs |  | C-1, C-9 |  |
| 14-OCH_3_ | 3.34, s | 57.1, CH_3_ | C-14 |  |
| 15-OAc | 1.91, s | 170.1, C  20.4, CH_3_ | C-15 |  |
| 25-OH | 4.86, d (3.0) |  |  |  |

**Table S7. 1D NMR and 2D NMR correlations data for compound 7**

| **No.** | **δ_H_ (J in Hz)** | **δ_C_, type** | **Key HMBC** | **^1^H-^1^H COSY** |
| --- | --- | --- | --- | --- |
| 1 |  | 161.7, C |  |  |
| 2 | 6.79, d (8.4) | 110.3, CH | C-1, C-4, C-9 | H-3 |
| 3 | 7.73, d (8.4) | 134.5, CH | C-1, C-4, C-10 | H-2 |
| 4 |  | 117.7, C |  |  |
| 5 | 7.24, s | 120.2, CH | C-11, C-7, C-12 |  |
| 6 |  | 137.7, C |  |  |
| 7 |  | 150.3, C |  |  |
| 8 |  | 115.0, C |  |  |
| 9 |  | 108.2, C |  |  |
| 10 |  | 152.0, C |  |  |
| 11 |  | 151.6, C |  |  |
| 12 |  | 116.3, C |  |  |
| 13 |  | 183.1, C |  |  |
| 14 | 5.28, brs | 68.8, CH | C-3, C-4, C-10, C-15 | H-15 |
| 15 | 4.27, d (6.0) | 79.1, CH |  | H-14 |
| 16 |  | 143.4, C |  |  |
| 17 | 4.82, s | 113.7, CH_2_ | C-15, C-16, C-18 |  |
| 18 | 1.82, s | 18.6, CH_3_ | C-15, C-16, C-17 |  |
| 19 | 4.55, brd (10.8)  4.27, dd (10.8,2.4) | 63.8, CH_2_ | C-7 | H-20 |
| 20 | 2.72, brs | 42.5, CH | C-8 | H-19 |
| 21 |  | 141.4, C |  |  |
| 22 | 4.85, s  4.77, s | 112.8, CH_2_ | C-20, C-23 |  |
| 23 | 1.89, s | 22.4, CH_3_ | C-20, C-22 |  |
| 24 | 2.35, s | 17.3, CH_3_ | C-5, C-7 |  |
| 25 | 6.90, brs | 65.5, CH | C-12, 25-OAc | H-20 |
| 1-OH | 13.00, brs |  | C-9, C-1 |  |
| 14-OH | 2.76, brs |  |  |  |
| 15-OH | 2.52, brs |  |  |  |
| 25-OAc | 2.08, s | 170.0, C  21.2, CH_3_ | C-25 |  |

**Table S8. 1D NMR and 2D NMR correlations data for compound 8**

| **No.** | **δ_H_ (J in Hz)** | **δ_C_, type** | **Key HMBC** | **^1^H-^1^H COSY** |
| --- | --- | --- | --- | --- |
| 1 |  | 161.4, C |  |  |
| 2 | 6.79, d (8.4) | 110.3, CH | C-1, C-4, C-9 | H-3 |
| 3 | 7.76, d (8.4) | 135.0, CH | C-1, C-4, C-10 | H-2 |
| 4 |  | 118.2, C |  |  |
| 5 | 7.20, s | 118.9, CH | C-11, C-7, C-12 |  |
| 6 |  | 138.7, C |  |  |
| 7 |  | 149.8, C |  |  |
| 8 |  | 121.4, C |  |  |
| 9 |  | 108.7, C |  |  |
| 10 |  | 152.1, C |  |  |
| 11 |  | 151.8, C |  |  |
| 12 |  | 116.8, C |  |  |
| 13 |  | 184.3, C |  |  |
| 14 | 5.28, brs | 68.8, CH | C-3, C-4, C-10, C-15 | H-15 |
| 15 | 4.26, d (5.4) | 79.1, CH |  | H-14 |
| 16 |  | 143.4, C |  |  |
| 17 | 4.82, s | 113.7, CH_2_ | C-15, C-16, C-18 |  |
| 18 | 1.83, s | 18.6, CH_3_ | C-15, C-16, C-17 |  |
| 19 |  | 64.8, CH_2_ | C-7 | H-20 |
| 20 |  | 45.0, CH | C-8 | H-19 |
| 21 |  | 142.5, C |  |  |
| 22 | 4.41, dd (10.8,2.4)  4.33, dd (10.8,2.4) | 112.3, CH_2_ | C-20, C-23 |  |
| 23 | 2.73, d (2.4) | 22.5, CH_3_ | C-20, C-22 |  |
| 24 | 2.35, s | 17.4, CH_3_ | C-5, C-7, |  |
| 25 | 5.41, brs | 63.3, CH | C-12 | H-20 |
| 1-OH | 12.70,brs |  | C-9, C-1 |  |
| 14-OH | 2.94, brs |  |  |  |
| 15-OH | 2.59, brs |  |  |  |
| 25-OH | 4.99, d (4.2) |  |  |  |


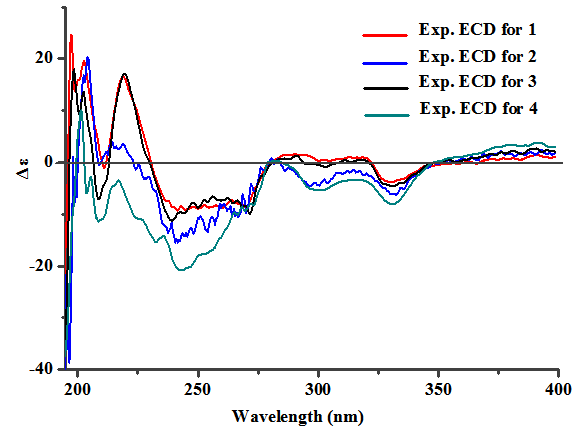


**Figure S1.** Experimental ECD spectra of **1**−**4**


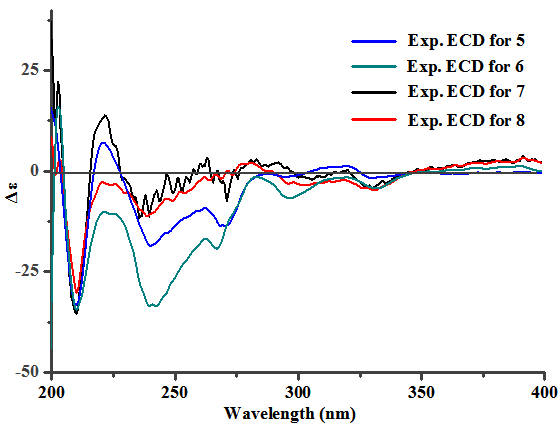


**Figure S2.** Experimental ECD spectra of **5**−**8**


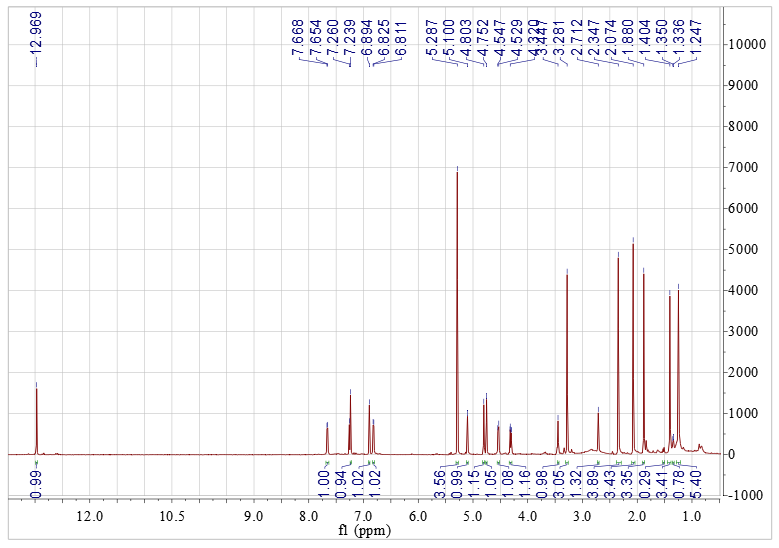


**Figure S3.** ^1^H NMR (600 MHz, CDCl_3_) spectrum of compound **1**


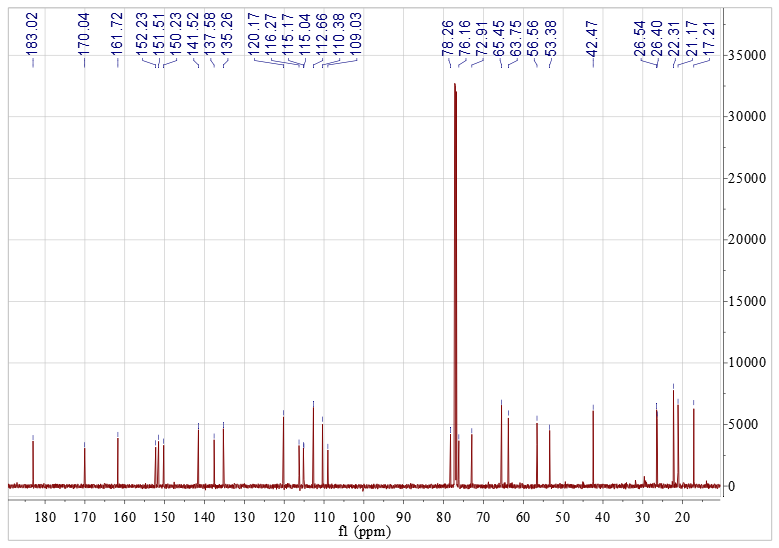


**Figure S4.** ^13^C NMR (150 MHz, CDCl_3_) spectrum of compound **1**


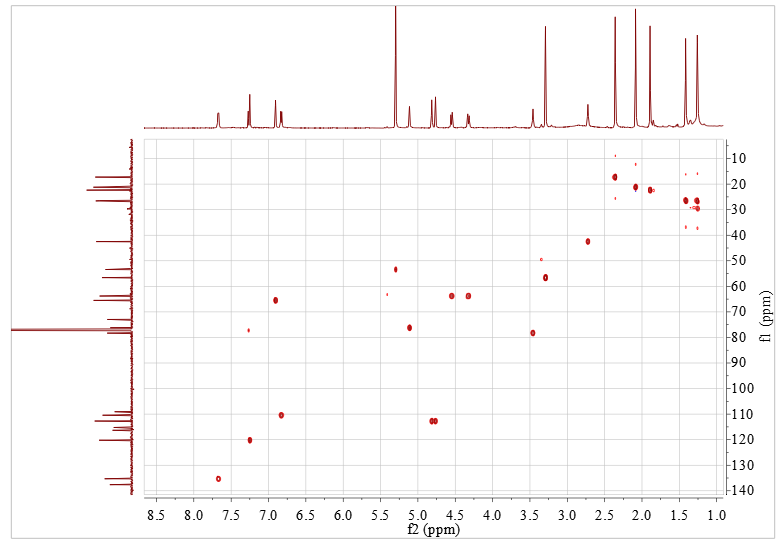


**Figure S5.** HSQC (CDCl_3_) spectrum of compound **1**


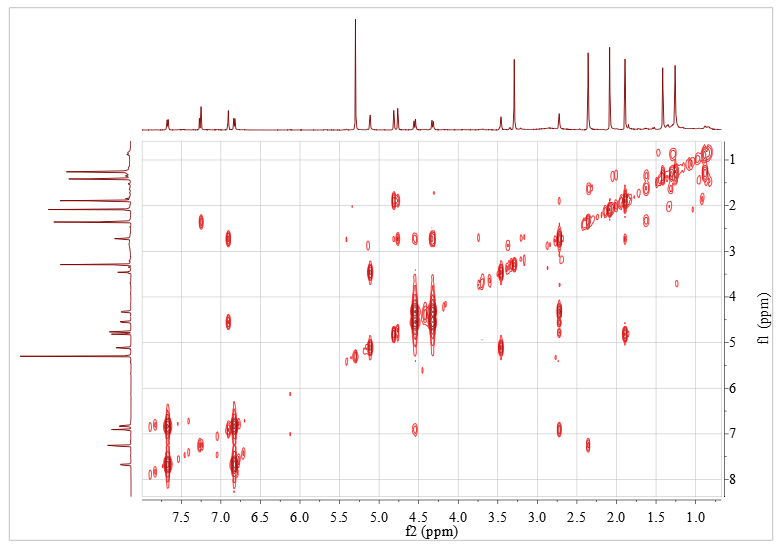


**Figure S6.** ^1^H-^1^H COSY (CDCl_3_) spectrum of compound **1**


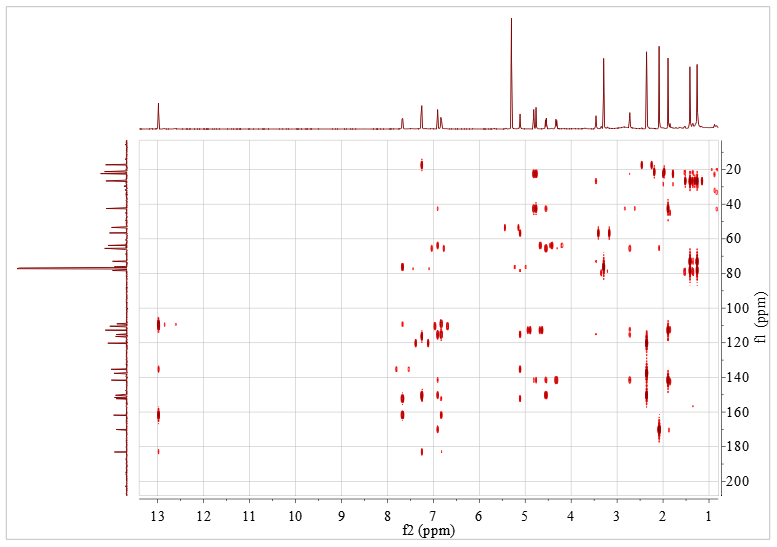


**Figure S7.** HMBC (CDCl_3_) spectrum of compound **1**


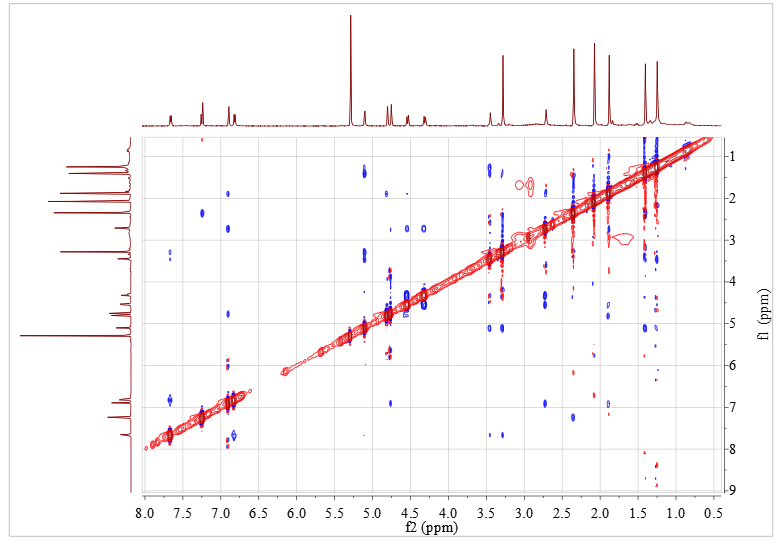


**Figure S8.** NOESY (CDCl_3_) spectrum of compound **1**

**Figure S9.** HRESIMS spectrum of compound **1**


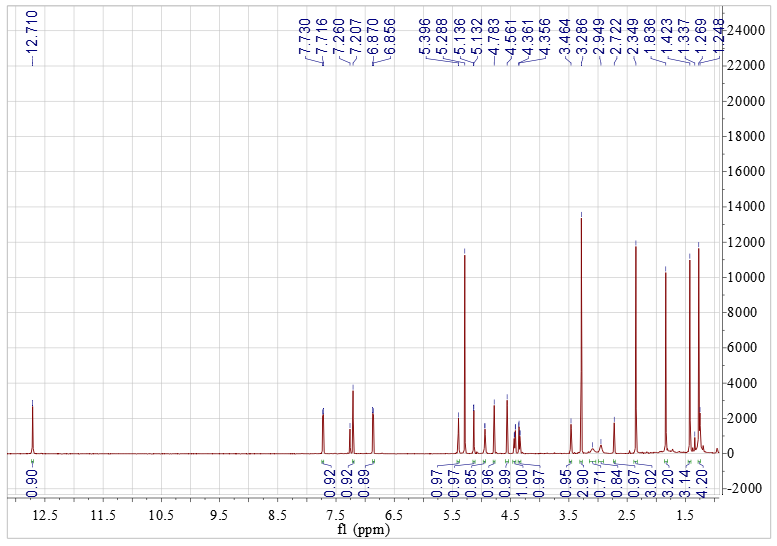


**Figure S10.** ^1^H NMR (600 MHz, CDCl_3_) spectrum of compound **2**


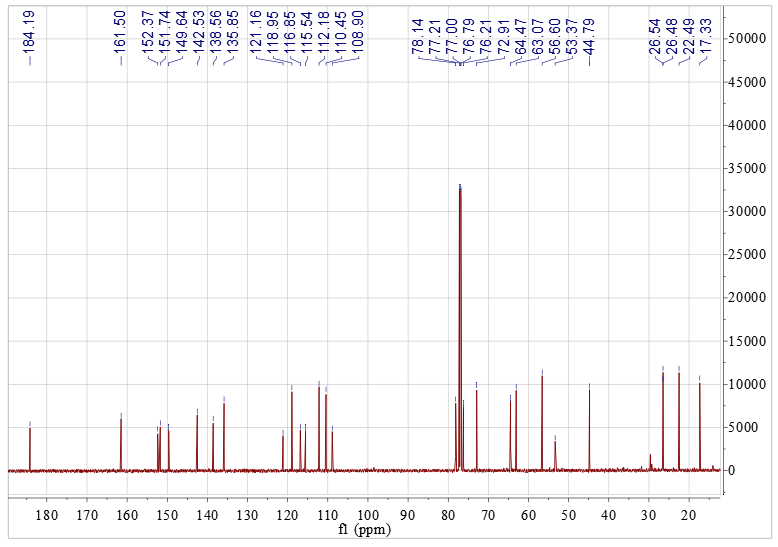


**Figure S11.** ^13^C NMR (150 MHz, CDCl_3_) spectrum of compound **2**


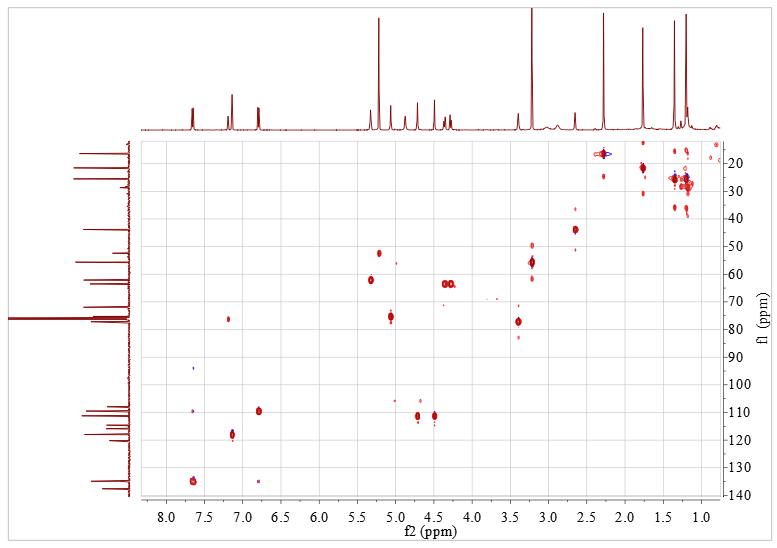


**Figure S12.** HSQC (CDCl_3_) spectrum of compound **2**


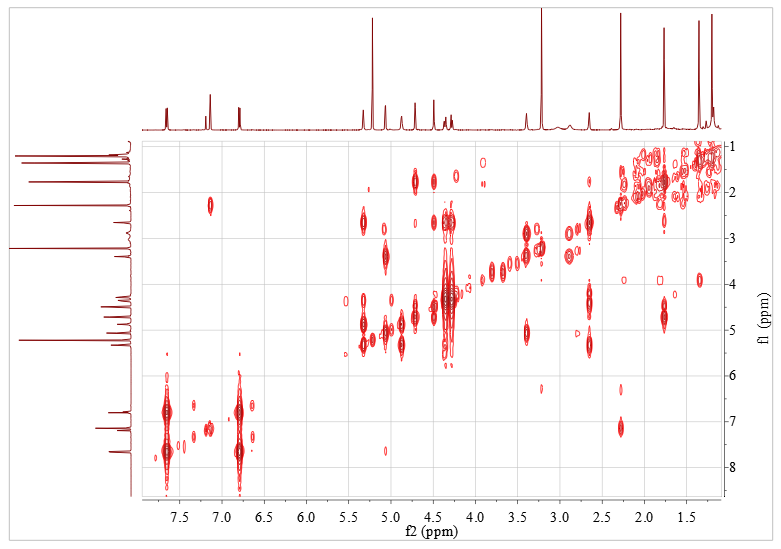


**Figure S13.** ^1^H-^1^H COSY (CDCl_3_) spectrum of compound **2**


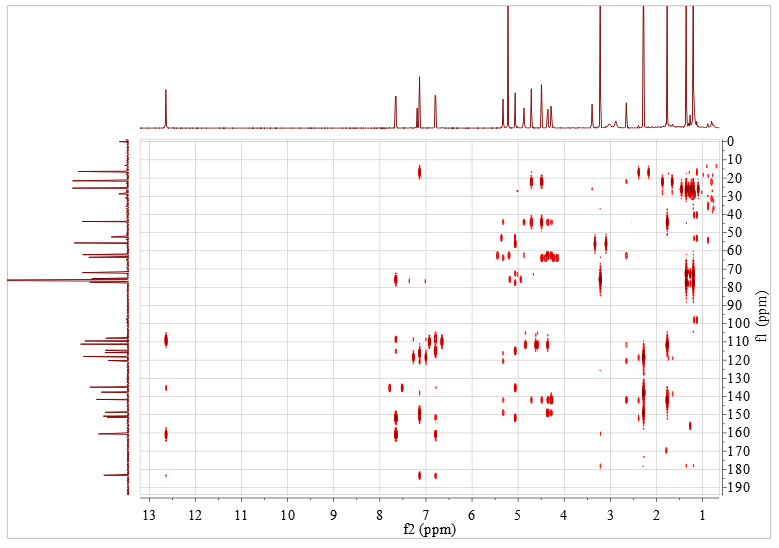


**Figure S14.** HMBC (CDCl_3_) spectrum of compound **2**


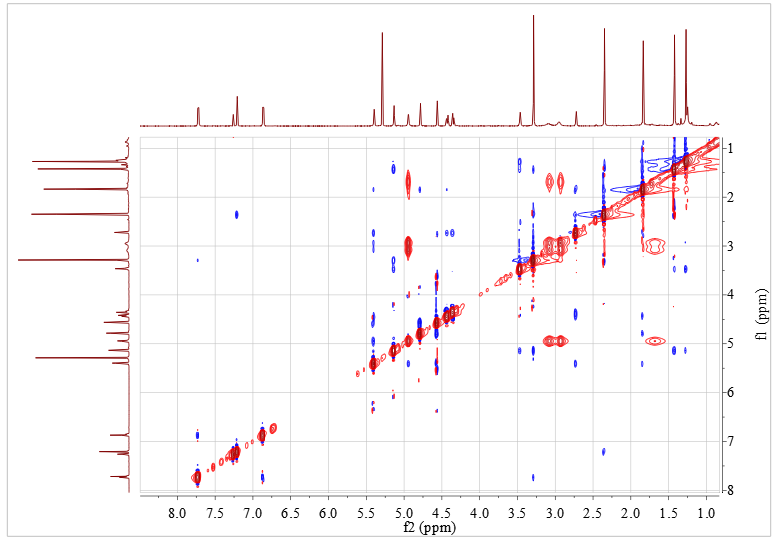


**Figure S15.** NOESY (CDCl_3_) spectrum of compound **2**

**Figure S16.** HRESIMS spectrum of compound **2**


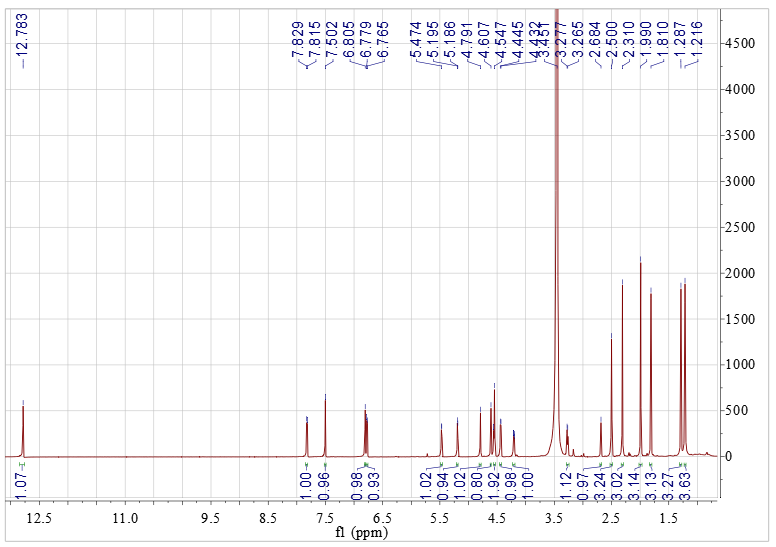


**Figure S17.** ^1^H NMR (600 MHz, DMSO-*d*_6_) spectrum of compound **3**


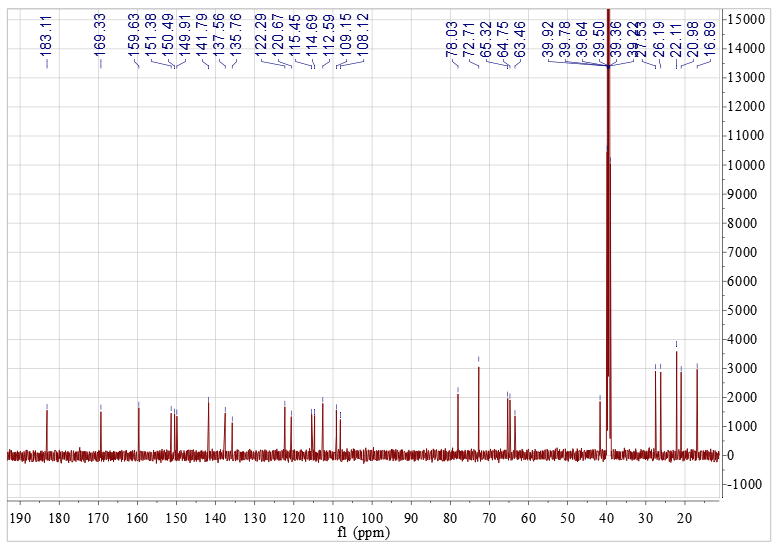


**Figure S18.** ^13^C NMR (150 MHz, DMSO-*d*_6_) spectrum of compound **3**


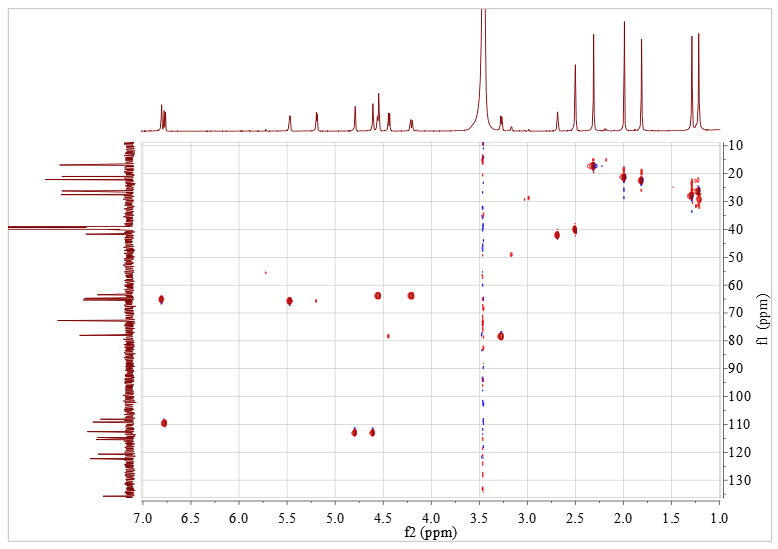


**Figure S19.** HSQC (DMSO-*d*_6_) spectrum of compound **3**


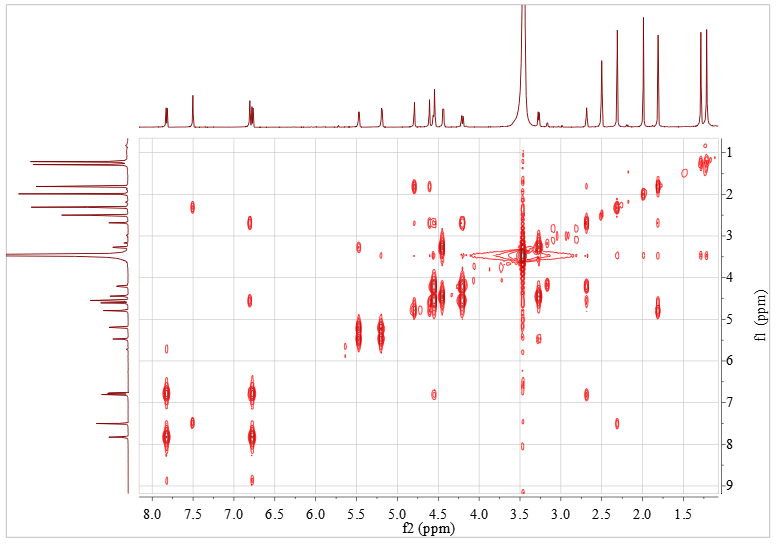


**Figure S20.** ^1^H-^1^H COSY (DMSO-*d*_6_) spectrum of compound **3**


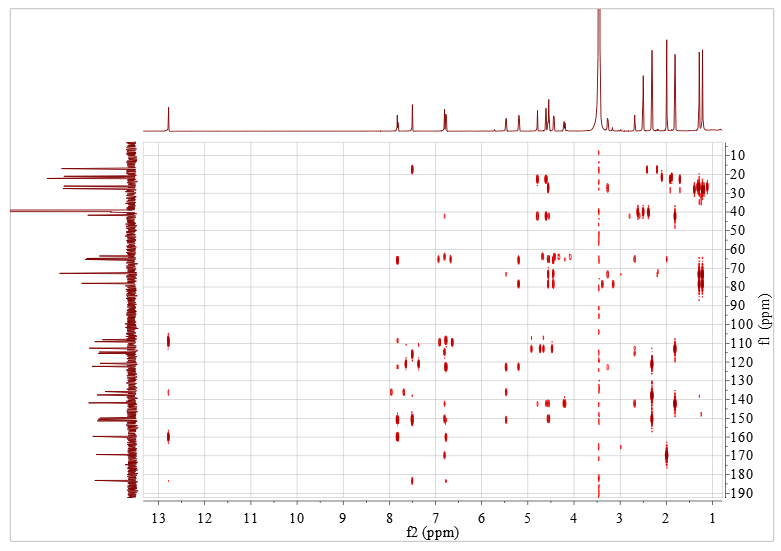


**Figure S21.** HMBC (DMSO-*d*_6_) spectrum of compound **3**


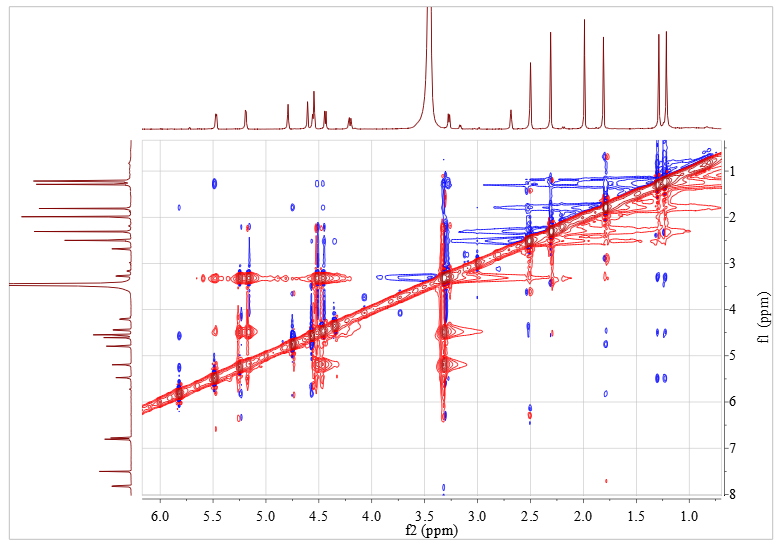


**Figure S22.** NOESY (DMSO-*d*_6_) spectrum of compound **3**

**Figure S23.** HRESIMS spectrum of compound **3**


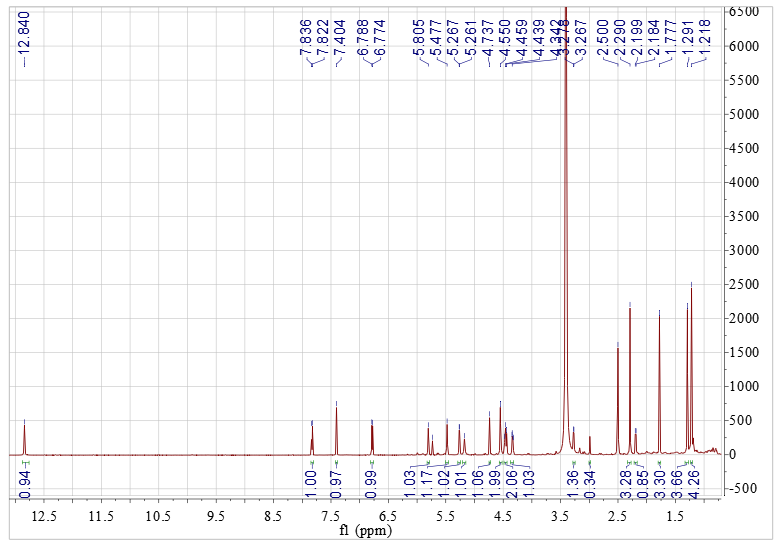


**Figure S24.** ^1^H NMR (600 MHz, DMSO-*d*_6_) spectrum of compound **4**


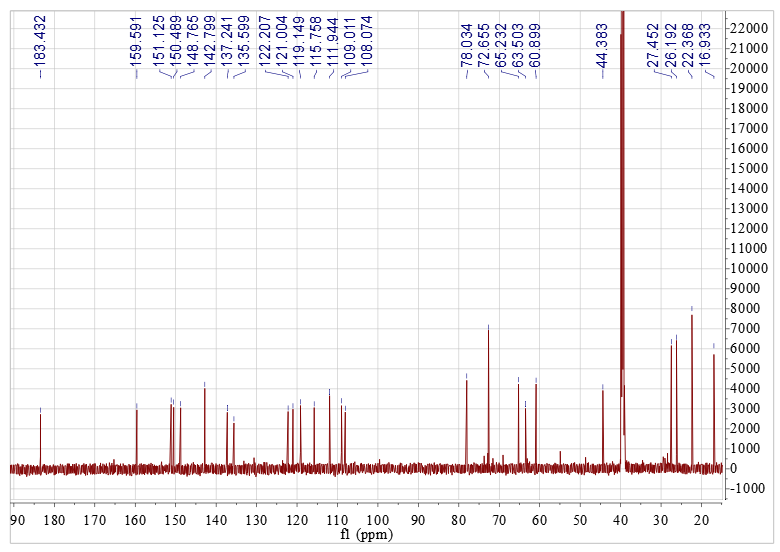


**Figure S25.** ^13^C NMR (150 MHz, DMSO-*d*_6_) spectrum of compound **4**


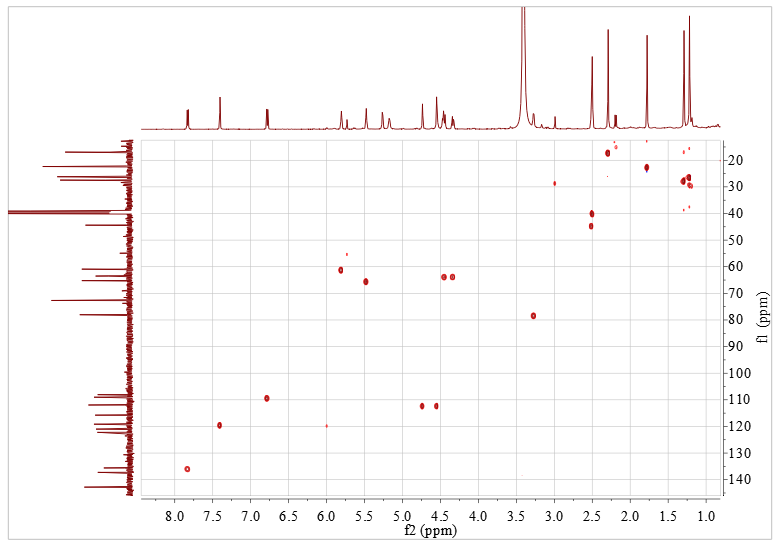


**Figure S26.** HSQC (DMSO-*d*_6_) spectrum of compound **4**


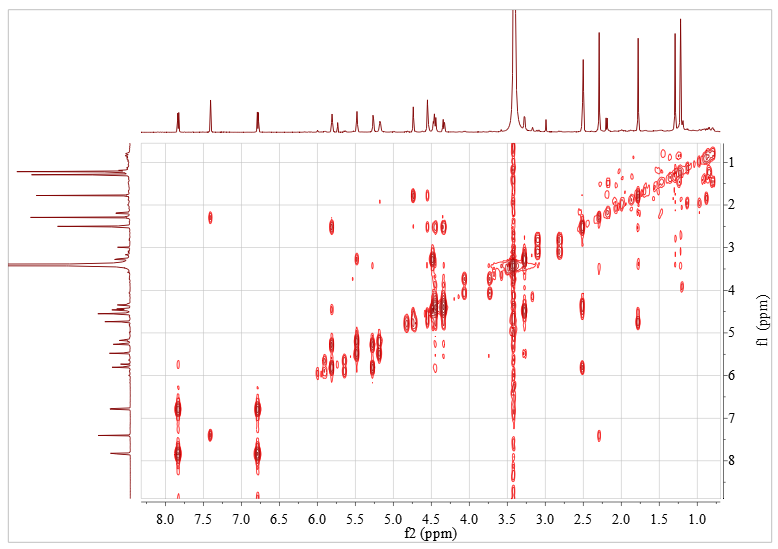


**Figure S27.** ^1^H-^1^H COSY (DMSO-*d*_6_) spectrum of compound **4**


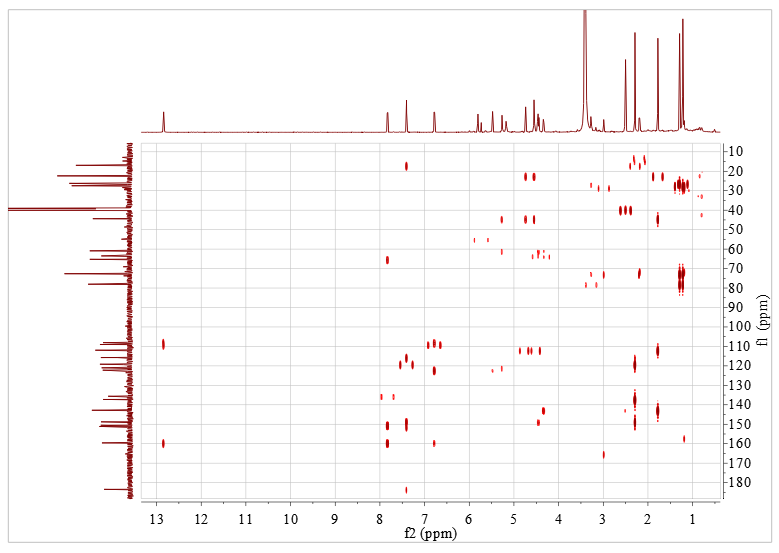


**Figure S28.** HMBC (DMSO-*d*_6_) spectrum of compound **4**


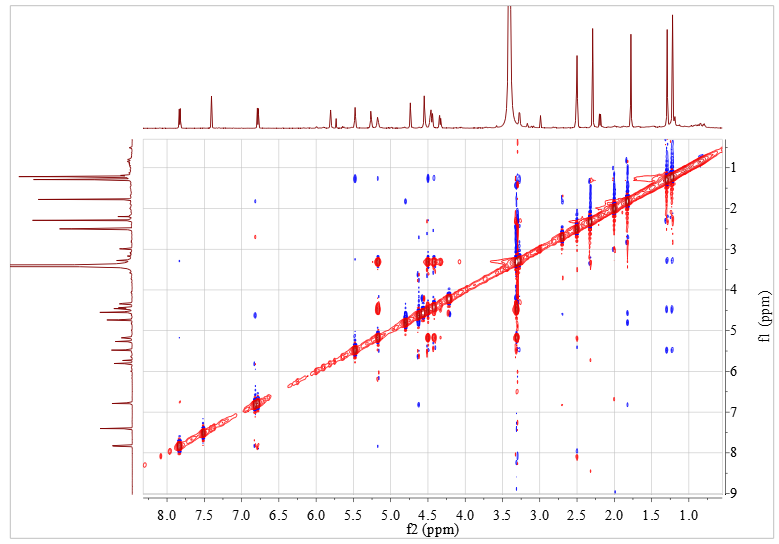


**Figure S29.** NOESY (DMSO-*d*_6_) spectrum of compound **4**

**Figure S30.** HRESIMS spectrum of compound **4**


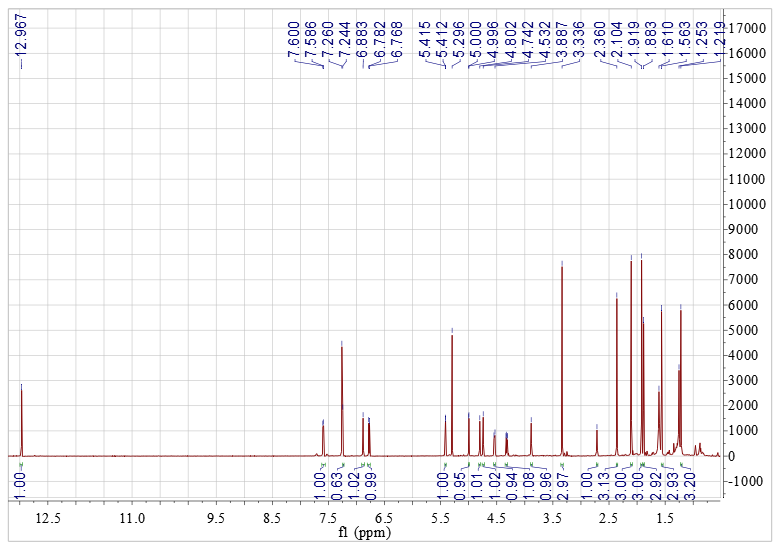


**Figure S31.** ^1^H NMR (600 MHz, CDCl_3_) spectrum of compound **5**


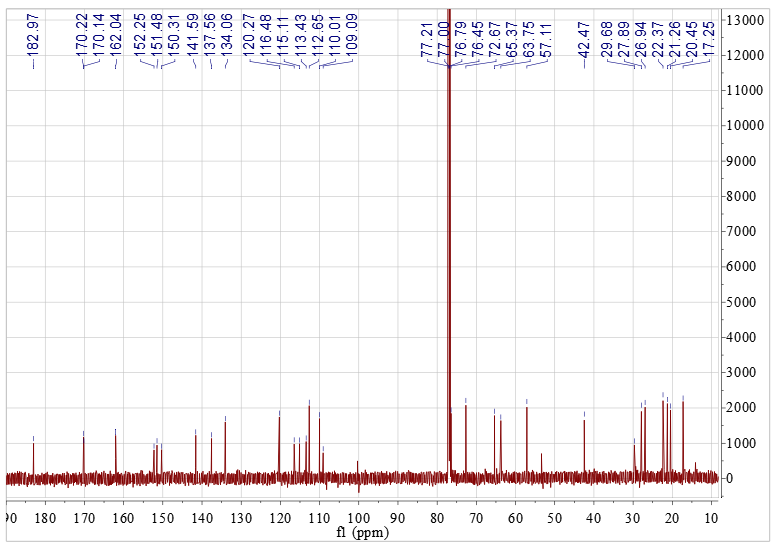


**Figure S32.** ^13^C NMR (150 MHz, CDCl_3_) spectrum of compound **5**


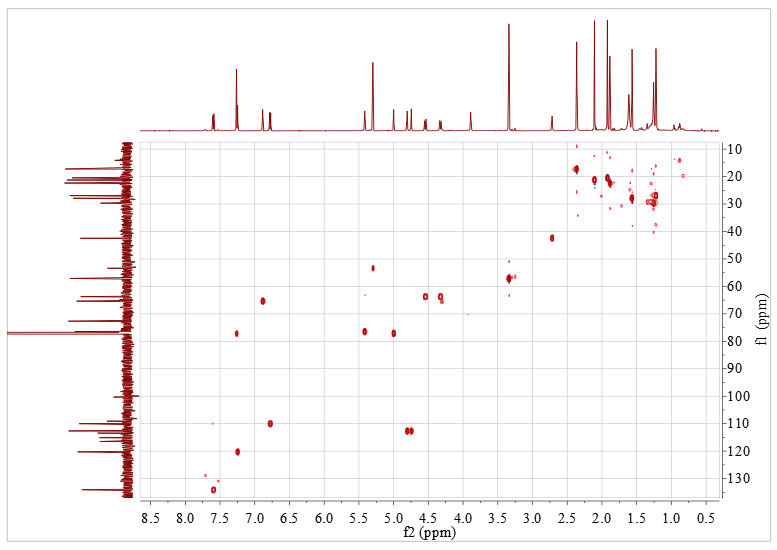


**Figure S33.** HSQC (CDCl_3_) spectrum of compound **5**


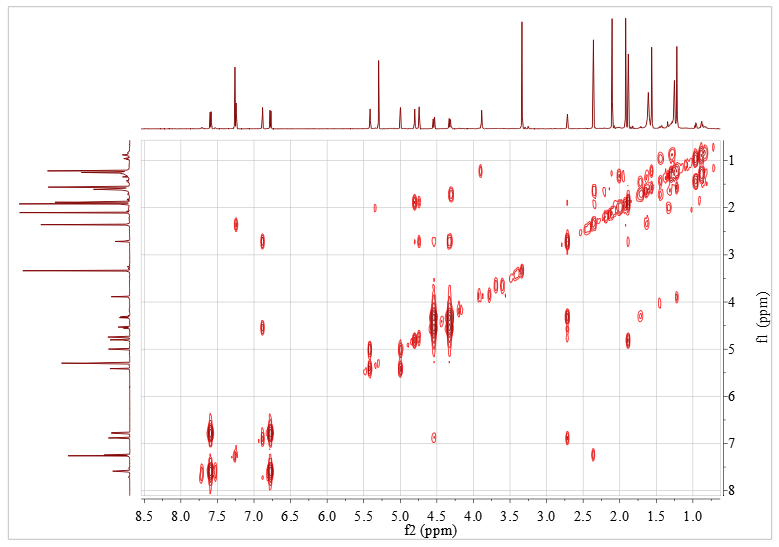


**Figure S34.** ^1^H-^1^H COSY (CDCl_3_) spectrum of compound **5**


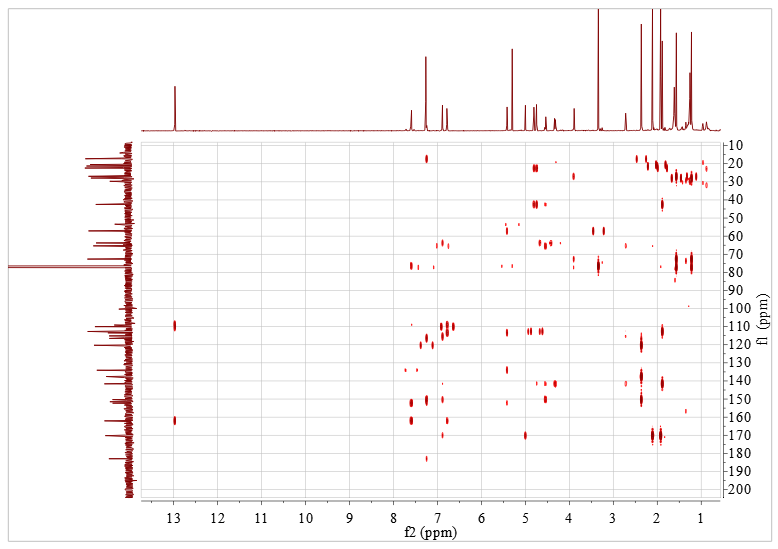


**Figure S35.** HMBC (CDCl_3_) spectrum of compound **5**


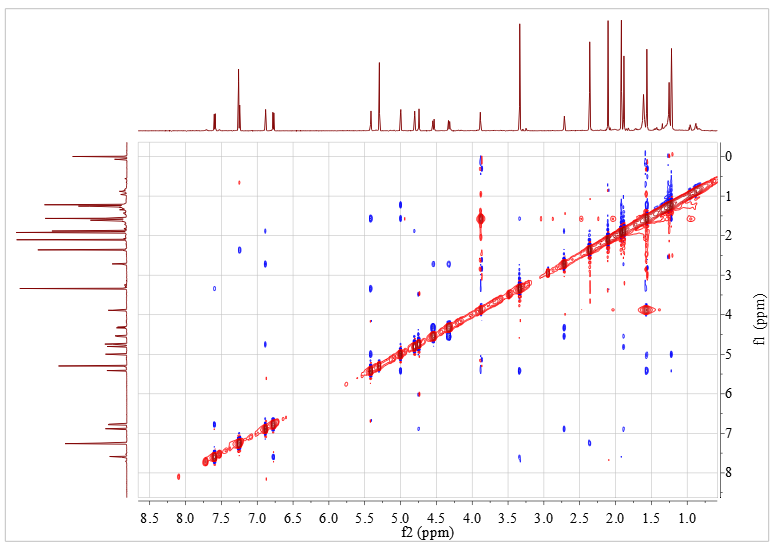


**Figure S36.** NOESY (CDCl_3_) spectrum of compound **5**

**Figure S37.** HRESIMS spectrum of compound **5**


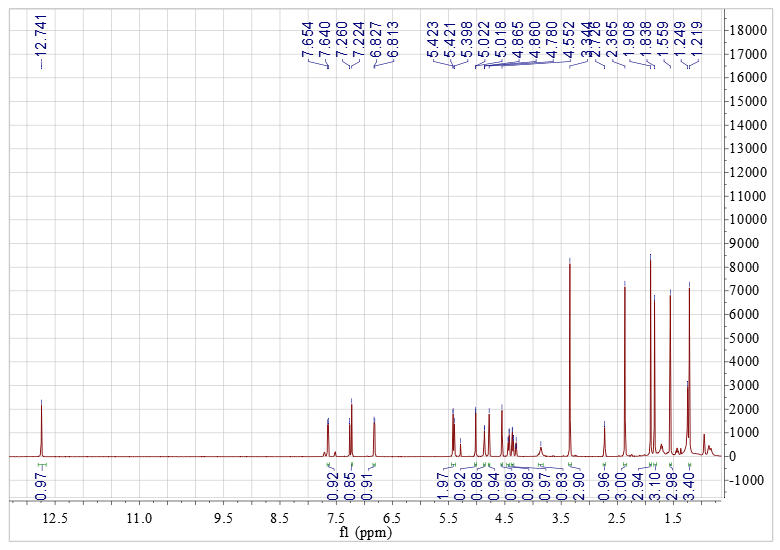


**Figure S38.** ^1^H NMR (600 MHz, CDCl_3_) spectrum of compound **6**


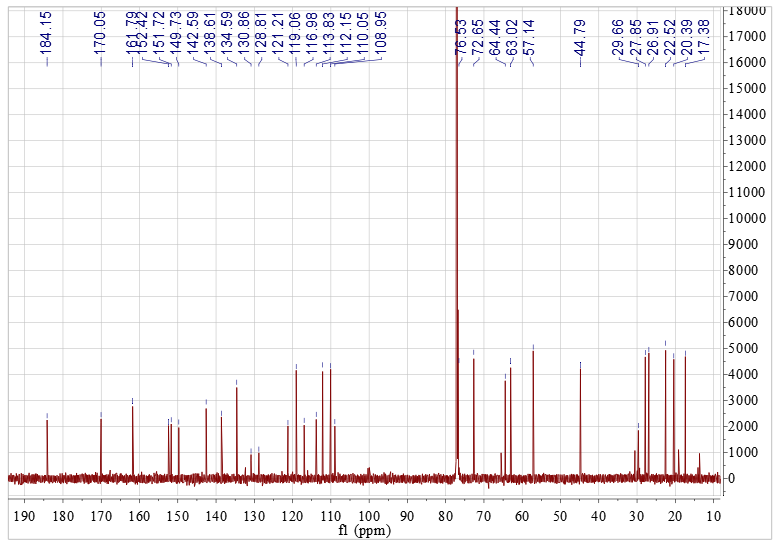


**Figure S39.** ^13^C NMR (150 MHz, CDCl_3_) spectrum of compound **6**


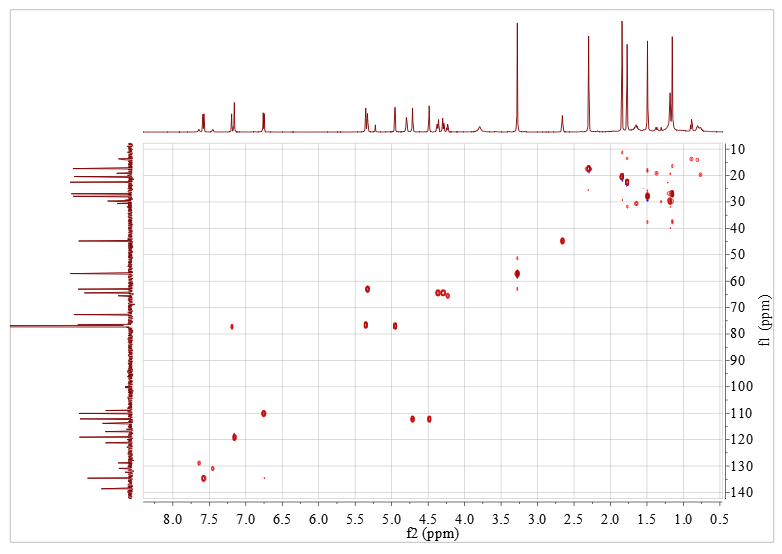


**Figure S40.** HSQC (CDCl_3_) spectrum of compound **6**


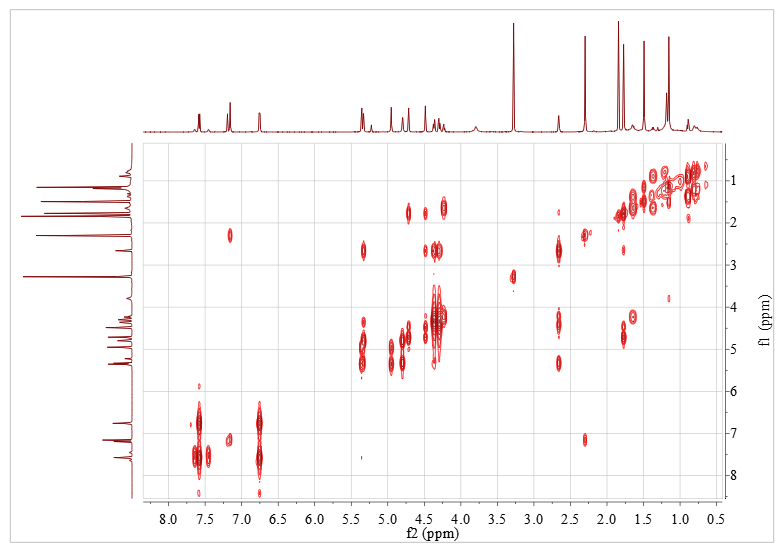


**Figure S41.** ^1^H-^1^H COSY (CDCl_3_) spectrum of compound **6**


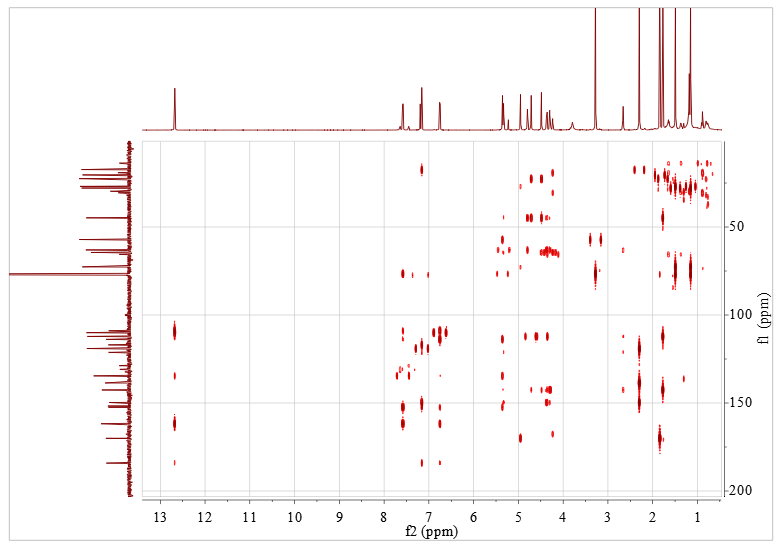


**Figure S42.** HMBC (CDCl_3_) spectrum of compound **6**


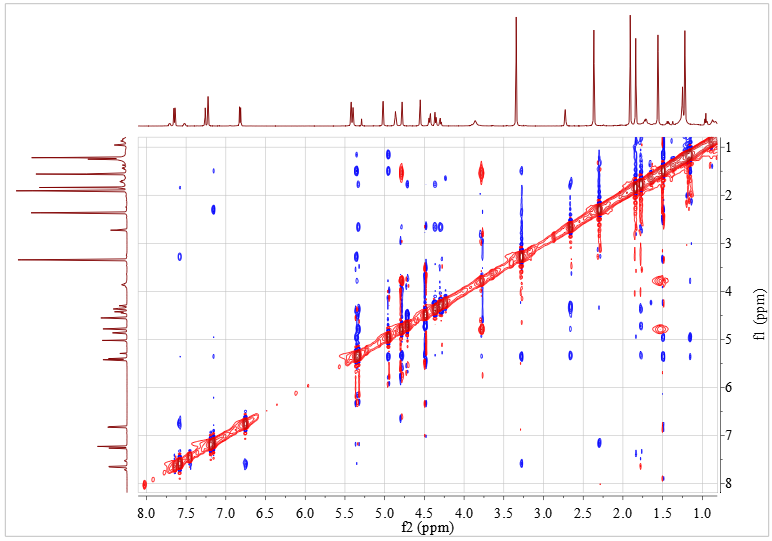


**Figure S43.** NOESY (CDCl_3_) spectrum of compound **6**

**Figure S44.** HRESIMS spectrum of compound **6**


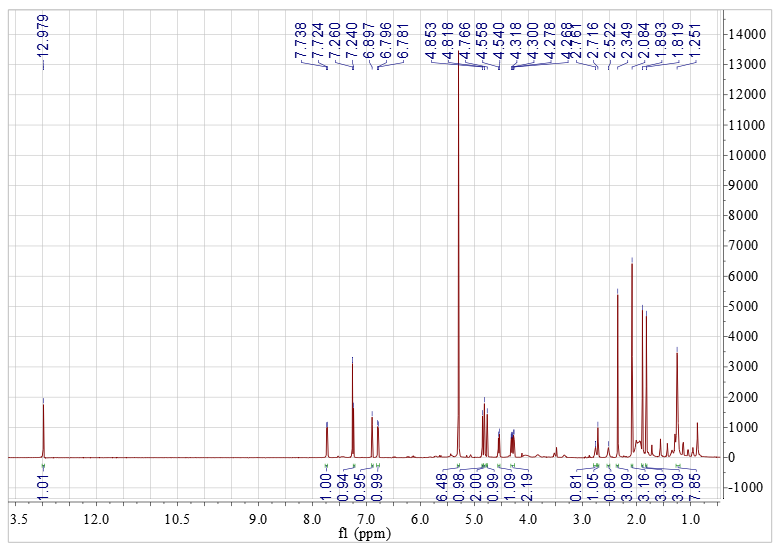


**Figure S45.** ^1^H NMR (600 MHz, CDCl_3_) spectrum of compound **7**


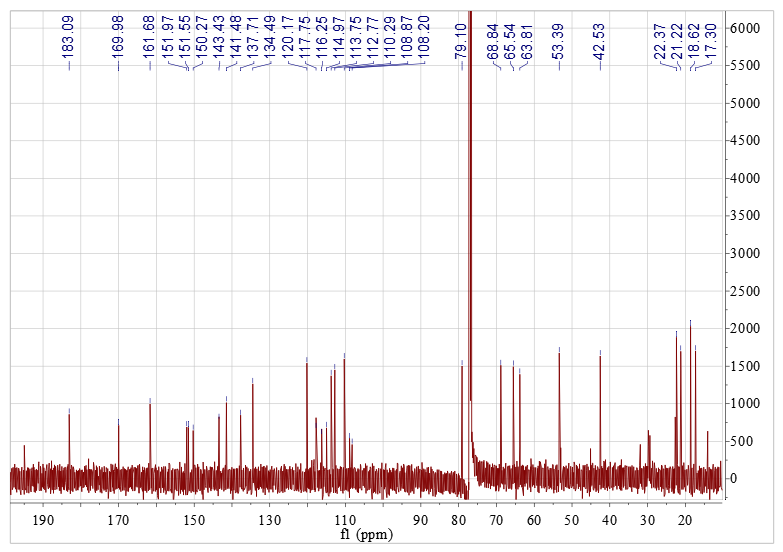


**Figure S46.** ^13^C NMR (150 MHz, CDCl_3_) spectrum of compound **7**


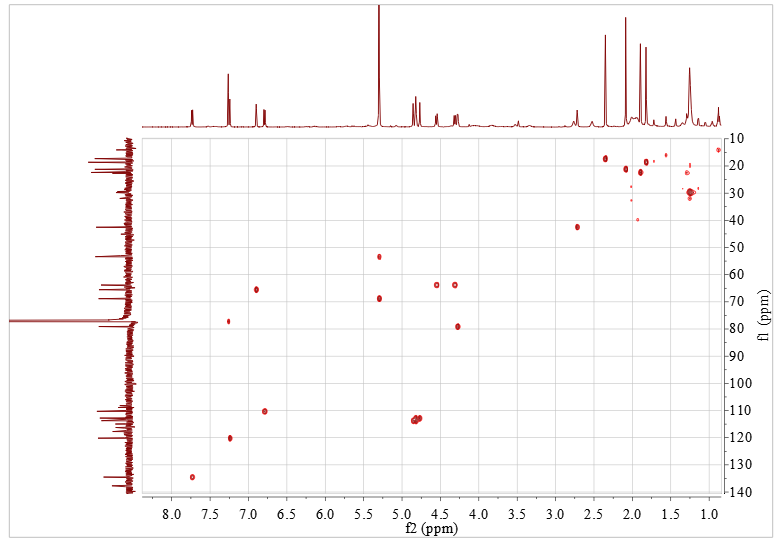


**Figure S47.** HSQC (CDCl_3_) spectrum of compound **7**


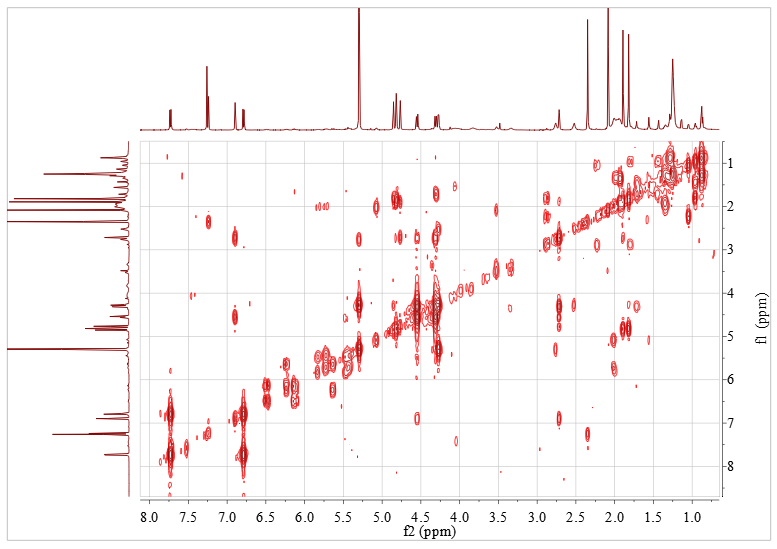


**Figure S48.** ^1^H-^1^H COSY (CDCl_3_) spectrum of compound **7**


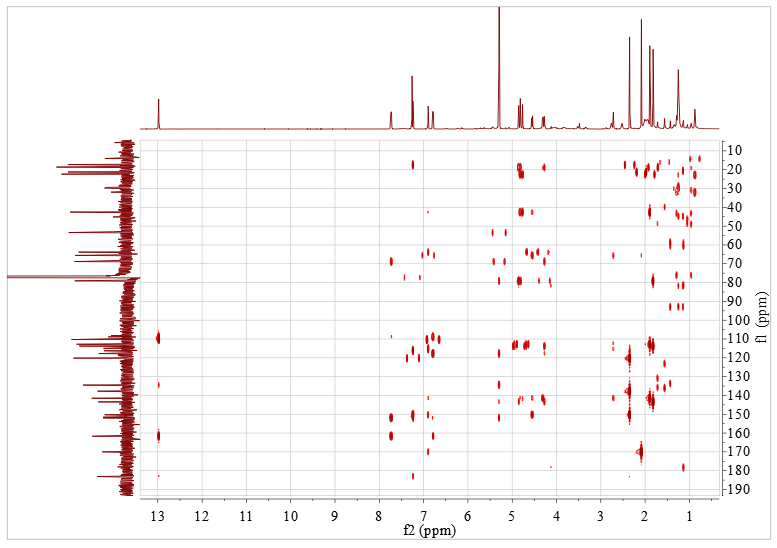


**Figure S49.** HMBC (CDCl_3_) spectrum of compound **7**


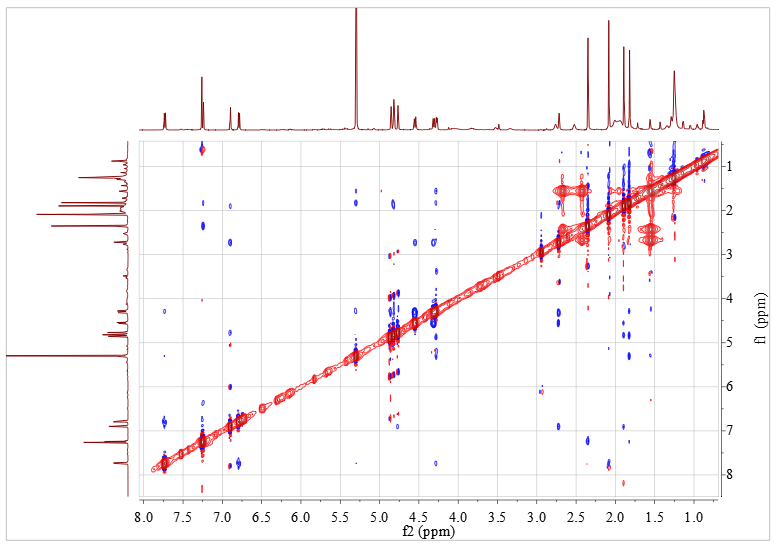


**Figure S50.** NOESY (CDCl_3_) spectrum of compound **7**

**Figure S51.** HRESIMS spectrum of compound **7**


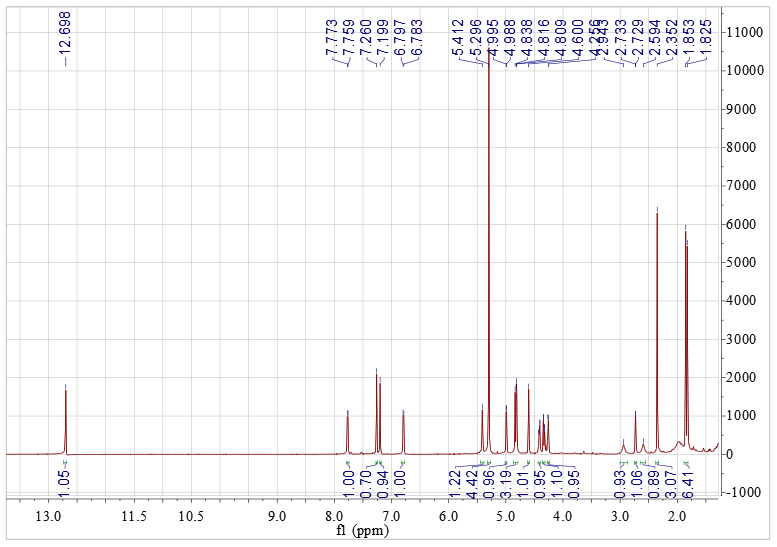


**Figure S52.** ^1^H NMR (600 MHz, CDCl_3_) spectrum of compound **8**


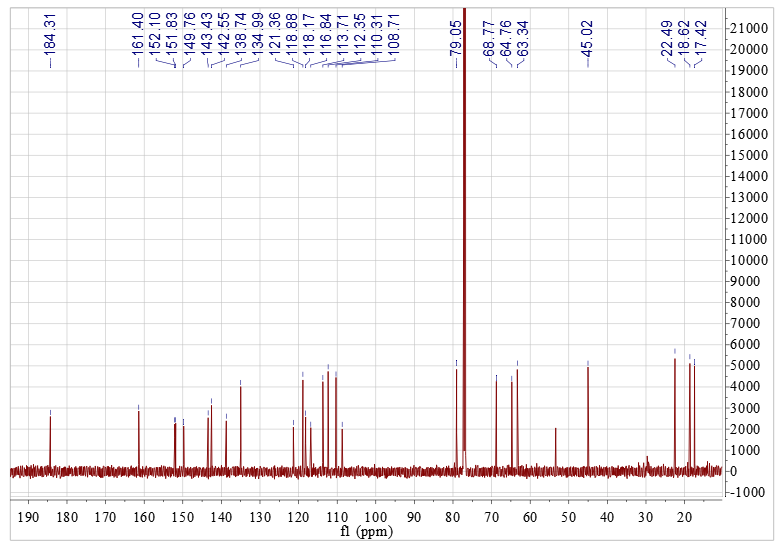


**Figure S53.** ^13^C NMR (150 MHz, CDCl_3_) spectrum of compound **8**


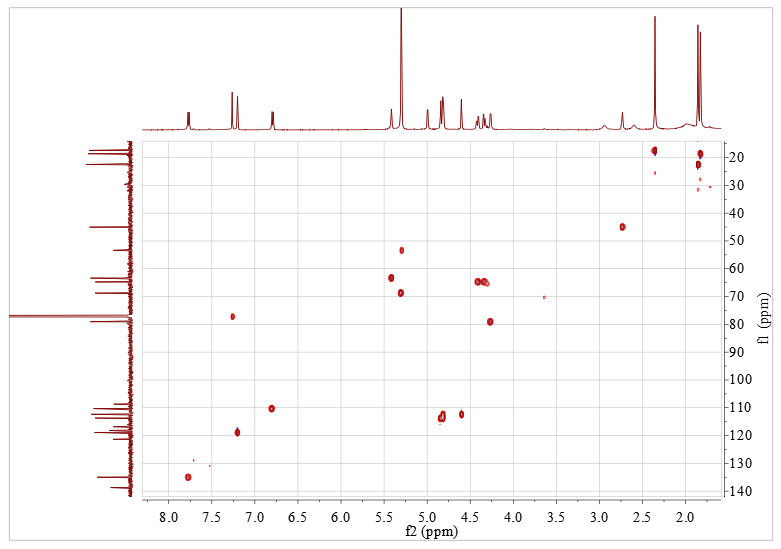


**Figure S54.** HSQC (CDCl_3_) spectrum of compound **8**


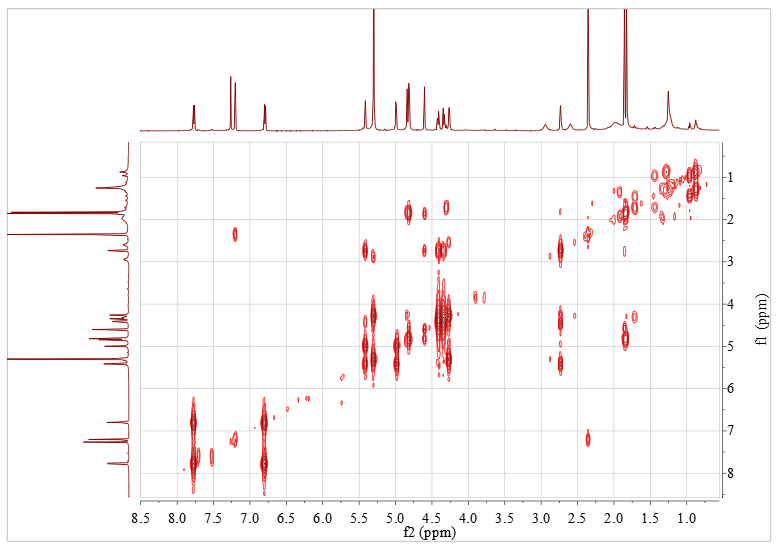


**Figure S55.** ^1^H-^1^H COSY (CDCl_3_) spectrum of compound **8**


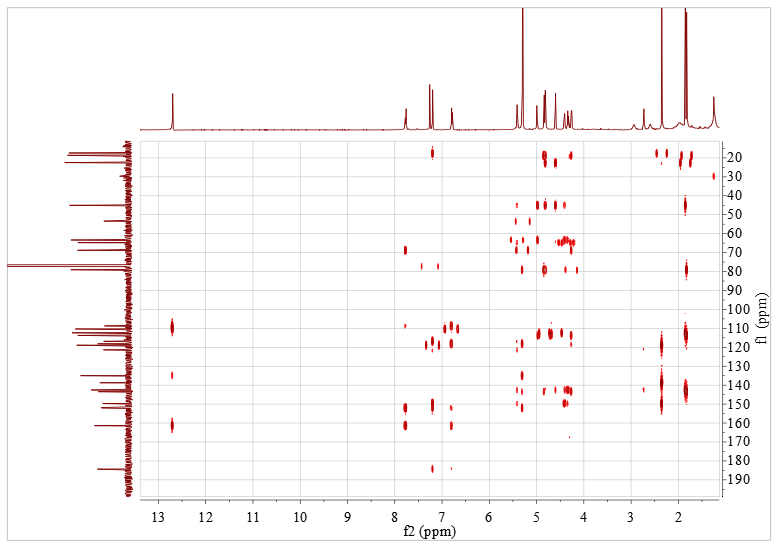


**Figure S56.** HMBC (CDCl_3_) spectrum of compound **8**


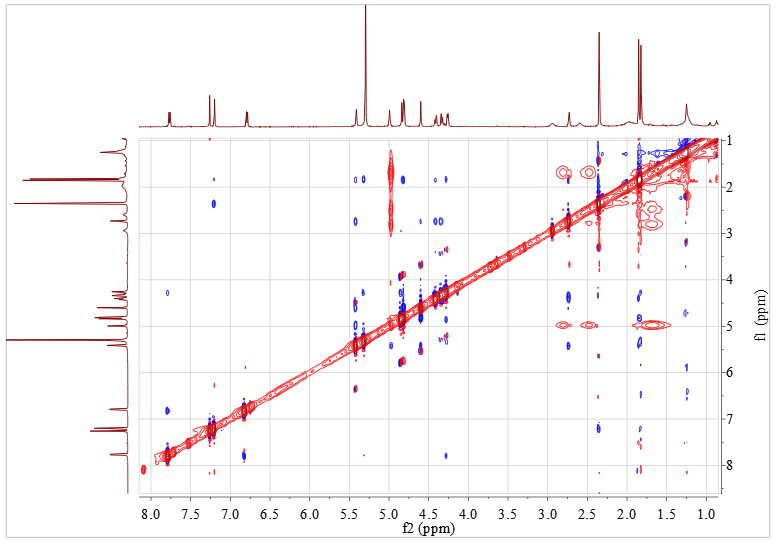


**Figure S57.** NOESY (CDCl_3_) spectrum of compound **8**

**Figure S58.** HRESIMS spectrum of compound **8**

1. Lowest energy conformers (14*R*,15*R*,20*S*,25*R*)-**1** for ECD calcation.

**C1**


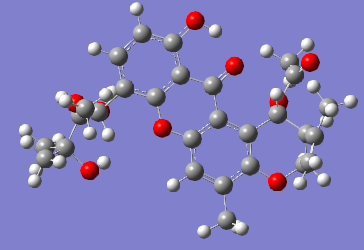


E = -1763.8132175 a.u

Standard orientation:

| Center Atomic Atomic Coordinates (Angstroms) |
| --- |
| Number Number Type X Y Z |
| 1 6 0 -2.224532 3.559154 0.578635 |
| 2 6 0 -3.224289 2.595696 0.645131 |
| 3 6 0 -2.958753 1.225047 0.565983 |
| 4 6 0 -1.618189 0.852607 0.417875 |
| 5 6 0 -0.568697 1.787403 0.344605 |
| 6 6 0 -0.893305 3.172535 0.428291 |
| 7 8 0 -1.354181 -0.478156 0.345844 |
| 8 6 0 -0.078624 -0.940136 0.188777 |
| 9 6 0 1.039972 -0.103516 0.094001 |
| 10 6 0 0.814533 1.347082 0.178467 |
| 11 6 0 0.029434 -2.331404 0.124511 |
| 12 6 0 1.259838 -2.928594 -0.044239 |
| 13 6 0 2.404883 -2.095903 -0.153138 |
| 14 6 0 2.323721 -0.708563 -0.072762 |
| 15 8 0 3.570829 -2.779671 -0.312072 |
| 16 6 0 4.747279 -2.033099 -0.655120 |
| 17 6 0 4.862461 -0.723405 0.109755 |
| 18 6 0 3.592601 0.112183 -0.166705 |
| 19 8 0 0.049398 4.120711 0.366210 |
| 20 6 0 -4.083861 0.201307 0.637346 |
| 21 6 0 -4.861481 0.065159 -0.700025 |
| 22 6 0 -5.513151 -1.327067 -0.920371 |
| 23 6 0 -6.357598 -1.793740 0.261361 |
| 24 6 0 -6.367811 -1.295054 -2.197295 |
| 25 8 0 -4.462130 -2.296629 -1.061263 |
| 26 8 0 1.736213 2.182152 0.110647 |
| 27 6 0 5.162663 -0.829274 1.600105 |
| 28 6 0 4.962374 -1.936564 2.318883 |
| 29 6 0 5.751948 0.415475 2.218858 |
| 30 8 0 -4.003570 0.240207 -1.832540 |
| 31 6 0 1.409550 -4.421525 -0.123441 |
| 32 8 0 3.637016 0.644251 -1.526783 |
| 33 8 0 4.976369 2.335314 -0.849718 |
| 34 6 0 4.311941 1.806732 -1.706289 |
| 35 6 0 4.104708 2.337076 -3.101390 |
| 36 8 0 -5.057617 0.582470 1.607391 |
| 37 6 0 -4.657877 0.358696 2.949000 |
| 38 1 0 -2.453309 4.616126 0.646558 |
| 39 1 0 -4.254096 2.908144 0.784006 |
| 40 1 0 -0.875098 -2.924504 0.201419 |
| 41 1 0 4.716343 -1.828173 -1.730435 |
| 42 1 0 5.579860 -2.704865 -0.448103 |
| 43 1 0 5.691023 -0.167364 -0.341390 |
| 44 1 0 3.547926 0.962430 0.503608 |
| 45 1 0 0.923526 3.671156 0.265615 |
| 46 1 0 -3.662804 -0.775317 0.894222 |
| 47 1 0 -5.642708 0.836445 -0.698908 |
| 48 1 0 -6.815324 -2.754301 0.013197 |
| 49 1 0 -5.752478 -1.934060 1.156968 |
| 50 1 0 -7.149008 -1.078326 0.491570 |
| 51 1 0 -6.770296 -2.291799 -2.390968 |
| 52 1 0 -5.777641 -0.984121 -3.060603 |
| 53 1 0 -7.207128 -0.600402 -2.093282 |
| 54 1 0 -3.848745 -1.954579 -1.726887 |
| 55 1 0 5.210186 -1.964450 3.375704 |
| 56 1 0 4.552438 -2.845731 1.896268 |
| 57 1 0 6.720918 0.652590 1.765459 |
| 58 1 0 5.123256 1.296123 2.059590 |
| 59 1 0 5.900748 0.292400 3.293217 |
| 60 1 0 -3.495452 1.053945 -1.728086 |
| 61 1 0 0.441035 -4.917207 -0.044021 |
| 62 1 0 1.878308 -4.720463 -1.064805 |
| 63 1 0 2.057075 -4.795589 0.674616 |
| 64 1 0 3.121185 2.812565 -3.154729 |
| 65 1 0 4.866742 3.080273 -3.329356 |
| 66 1 0 4.122520 1.533798 -3.839255 |
| 67 1 0 -5.489360 0.669226 3.581648 |
| 68 1 0 -4.447355 -0.703585 3.128393 |
| 69 1 0 -3.769336 0.944587 3.212186 |

**C2**


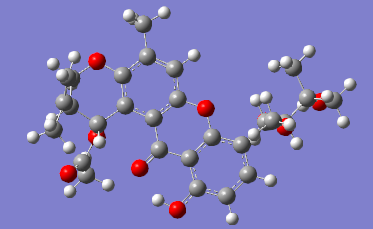


E = -1763.8118209 a.u

Standard orientation:

| Center Atomic Atomic Coordinates (Angstroms) |
| --- |
| Number Number Type X Y Z |
| 1 6 0 2.235743 -3.438425 0.668976 |
| 2 6 0 3.213085 -2.451781 0.703172 |
| 3 6 0 2.919633 -1.091002 0.568838 |
| 4 6 0 1.572740 -0.752074 0.403595 |
| 5 6 0 0.543458 -1.712401 0.362933 |
| 6 6 0 0.897291 -3.086192 0.497988 |
| 7 8 0 1.276627 0.570004 0.284392 |
| 8 6 0 -0.009870 0.995776 0.112423 |
| 9 6 0 -1.109398 0.131351 0.053876 |
| 10 6 0 -0.849537 -1.310114 0.184192 |
| 11 6 0 -0.152373 2.380334 -0.006115 |
| 12 6 0 -1.396612 2.942524 -0.192780 |
| 13 6 0 -2.522637 2.080525 -0.261110 |
| 14 6 0 -2.407857 0.699691 -0.125622 |
| 15 8 0 -3.704329 2.730442 -0.440849 |
| 16 6 0 -4.871441 1.943598 -0.723056 |
| 17 6 0 -4.939453 0.670559 0.105873 |
| 18 6 0 -3.659893 -0.150352 -0.169535 |
| 19 8 0 -0.023702 -4.056638 0.467579 |
| 20 6 0 4.031719 -0.049669 0.614552 |
| 21 6 0 4.890288 -0.091876 -0.671517 |
| 22 6 0 5.927469 1.047556 -0.881771 |
| 23 6 0 5.282620 2.438630 -0.796551 |
| 24 6 0 7.125438 0.938241 0.057217 |
| 25 8 0 6.472846 0.858048 -2.197703 |
| 26 8 0 -1.752085 -2.167433 0.143771 |
| 27 6 0 -5.201560 0.846056 1.597041 |
| 28 6 0 -5.063949 2.008813 2.238980 |
| 29 6 0 -5.682873 -0.393513 2.312296 |
| 30 8 0 4.023355 -0.020600 -1.815003 |
| 31 6 0 -1.581116 4.427190 -0.331015 |
| 32 8 0 -3.720966 -0.724792 -1.511186 |
| 33 8 0 -5.015345 -2.417680 -0.755943 |
| 34 6 0 -4.378353 -1.904058 -1.641814 |
| 35 6 0 -4.190007 -2.472270 -3.024569 |
| 36 8 0 4.908245 -0.282627 1.712918 |
| 37 6 0 4.345163 0.015122 2.979826 |
| 38 1 0 2.486635 -4.486848 0.778285 |
| 39 1 0 4.247631 -2.739399 0.860727 |
| 40 1 0 0.736858 2.998695 0.042598 |
| 41 1 0 -4.858596 1.688648 -1.787737 |
| 42 1 0 -5.714223 2.606677 -0.529973 |
| 43 1 0 -5.768487 0.076546 -0.293504 |
| 44 1 0 -3.585038 -0.978271 0.525235 |
| 45 1 0 -0.907414 -3.631084 0.348477 |
| 46 1 0 3.580258 0.942714 0.704930 |
| 47 1 0 5.443070 -1.039524 -0.688455 |
| 48 1 0 6.013222 3.186884 -1.110529 |
| 49 1 0 4.413016 2.512631 -1.453864 |
| 50 1 0 4.967170 2.684129 0.221754 |
| 51 1 0 7.879081 1.666658 -0.251785 |
| 52 1 0 7.574519 -0.055491 -0.004191 |
| 53 1 0 6.848469 1.126782 1.092937 |
| 54 1 0 5.727975 0.707907 -2.796516 |
| 55 1 0 -5.287375 2.087716 3.298612 |
| 56 1 0 -4.729746 2.915343 1.749159 |
| 57 1 0 -6.625216 -0.751216 1.883201 |
| 58 1 0 -4.978095 -1.225268 2.223591 |
| 59 1 0 -5.844410 -0.199256 3.374220 |
| 60 1 0 3.516230 -0.837875 -1.882113 |
| 61 1 0 -0.624377 4.948397 -0.273609 |
| 62 1 0 -2.057516 4.677529 -1.282651 |
| 63 1 0 -2.236158 4.817707 0.452813 |
| 64 1 0 -3.199287 -2.931758 -3.084550 |
| 65 1 0 -4.942876 -3.235225 -3.214493 |
| 66 1 0 -4.237523 -1.691813 -3.785275 |
| 67 1 0 5.121379 -0.174136 3.721076 |
| 68 1 0 4.039023 1.067737 3.040381 |
| 69 1 0 3.478328 -0.618747 3.199513 |

**C3**


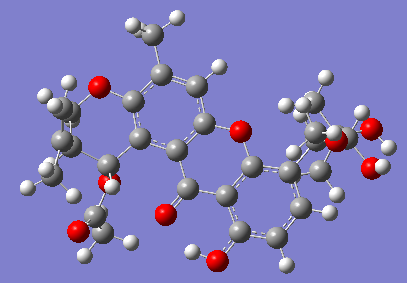


E = -1763.8114181 a.u

Standard orientation:

| Center Atomic Atomic Coordinates (Angstroms) |
| --- |
| Number Number Type X Y Z |
| 1 6 0 2.650781 -2.983823 1.003924 |
| 2 6 0 3.525242 -1.906526 1.033343 |
| 3 6 0 3.109491 -0.588161 0.813693 |
| 4 6 0 1.744780 -0.395290 0.580568 |
| 5 6 0 0.815116 -1.453000 0.542110 |
| 6 6 0 1.294709 -2.777276 0.756708 |
| 7 8 0 1.322499 0.886388 0.376092 |
| 8 6 0 0.004494 1.174659 0.156286 |
| 9 6 0 -1.000086 0.202448 0.100094 |
| 10 6 0 -0.600320 -1.200805 0.290588 |
| 11 6 0 -0.273437 2.532825 -0.015479 |
| 12 6 0 -1.561744 2.959957 -0.256644 |
| 13 6 0 -2.592427 1.986805 -0.324151 |
| 14 6 0 -2.342676 0.629991 -0.134544 |
| 15 8 0 -3.828102 2.506634 -0.558363 |
| 16 6 0 -4.902275 1.595835 -0.839112 |
| 17 6 0 -4.863781 0.350729 0.032631 |
| 18 6 0 -3.500703 -0.343962 -0.179709 |
| 19 8 0 0.475819 -3.835962 0.734717 |
| 20 6 0 4.084297 0.571866 0.884804 |
| 21 6 0 5.208304 0.641097 -0.175291 |
| 22 6 0 4.850265 0.724416 -1.678229 |
| 23 6 0 4.226213 -0.554819 -2.249668 |
| 24 6 0 3.981014 1.944671 -1.970358 |
| 25 8 0 6.093497 0.973992 -2.359760 |
| 26 8 0 -1.411420 -2.144848 0.244572 |
| 27 6 0 -5.188234 0.546481 1.508943 |
| 28 6 0 -5.192481 1.737669 2.112407 |
| 29 6 0 -5.559705 -0.712827 2.254481 |
| 30 8 0 6.116033 -0.455973 -0.028661 |
| 31 6 0 -1.888865 4.413510 -0.452210 |
| 32 8 0 -3.464637 -0.967826 -1.499702 |
| 33 8 0 -4.598644 -2.758034 -0.711842 |
| 34 6 0 -3.992599 -2.213276 -1.600866 |
| 35 6 0 -3.706558 -2.808412 -2.955079 |
| 36 8 0 4.821025 0.550267 2.121939 |
| 37 6 0 4.053600 0.850833 3.275493 |
| 38 1 0 2.998413 -3.996272 1.172178 |
| 39 1 0 4.574226 -2.086830 1.229333 |
| 40 1 0 0.545566 3.241439 0.038884 |
| 41 1 0 -4.835559 1.308697 -1.893490 |
| 42 1 0 -5.812442 2.175875 -0.689387 |
| 43 1 0 -5.615776 -0.338087 -0.367106 |
| 44 1 0 -3.363738 -1.135693 0.547177 |
| 45 1 0 -0.437702 -3.507344 0.553518 |
| 46 1 0 3.526233 1.511586 0.830922 |
| 47 1 0 5.749191 1.569433 0.048525 |
| 48 1 0 4.169823 -0.457623 -3.336175 |
| 49 1 0 4.837128 -1.428402 -2.014569 |
| 50 1 0 3.220216 -0.735504 -1.867658 |
| 51 1 0 3.892579 2.071244 -3.051477 |
| 52 1 0 4.435627 2.852887 -1.565793 |
| 53 1 0 2.977128 1.835335 -1.555917 |
| 54 1 0 6.721837 0.300931 -2.063378 |
| 55 1 0 -5.455634 1.827345 3.162047 |
| 56 1 0 -4.939136 2.657976 1.600309 |
| 57 1 0 -6.448321 -1.179127 1.815107 |
| 58 1 0 -4.771349 -1.469663 2.210897 |
| 59 1 0 -5.769372 -0.502941 3.304935 |
| 60 1 0 6.502119 -0.393252 0.854083 |
| 61 1 0 -0.991717 5.030178 -0.379843 |
| 62 1 0 -2.351182 4.585386 -1.427991 |
| 63 1 0 -2.608891 4.760023 0.294357 |
| 64 1 0 -2.672856 -3.165326 -2.971425 |
| 65 1 0 -4.371907 -3.651125 -3.134264 |
| 66 1 0 -3.810813 -2.064879 -3.746492 |
| 67 1 0 4.737865 0.825619 4.123468 |
| 68 1 0 3.608318 1.851462 3.203307 |
| 69 1 0 3.257095 0.115886 3.435571 |

**C4**


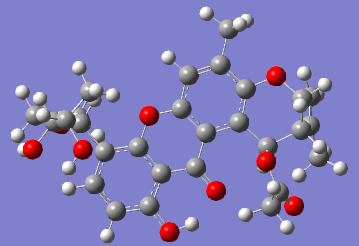


E = -1763.8112703 a.u

Standard orientation:

| Center Atomic Atomic Coordinates (Angstroms) |
| --- |
| Number Number Type X Y Z |
| 1 6 0 -2.674429 -2.963948 -1.035732 |
| 2 6 0 -3.544080 -1.882007 -1.065299 |
| 3 6 0 -3.127852 -0.566806 -0.834008 |
| 4 6 0 -1.762356 -0.380450 -0.603314 |
| 5 6 0 -0.837720 -1.441781 -0.561086 |
| 6 6 0 -1.320785 -2.764420 -0.775117 |
| 7 8 0 -1.331758 0.900687 -0.415121 |
| 8 6 0 -0.013836 1.183716 -0.194702 |
| 9 6 0 0.984213 0.206388 -0.121233 |
| 10 6 0 0.576744 -1.196063 -0.302021 |
| 11 6 0 0.273086 2.542636 -0.042688 |
| 12 6 0 1.562948 2.964780 0.198451 |
| 13 6 0 2.586150 1.985856 0.287179 |
| 14 6 0 2.328088 0.628336 0.115673 |
| 15 8 0 3.824523 2.500483 0.522719 |
| 16 6 0 4.887409 1.585777 0.830850 |
| 17 6 0 4.849181 0.327383 -0.021736 |
| 18 6 0 3.478292 -0.353343 0.185237 |
| 19 8 0 -0.506376 -3.827331 -0.746327 |
| 20 6 0 -4.105423 0.593685 -0.878464 |
| 21 6 0 -5.184049 0.659747 0.232662 |
| 22 6 0 -4.717472 0.728205 1.705472 |
| 23 6 0 -3.730724 1.870778 1.937352 |
| 24 6 0 -5.945752 0.907401 2.613451 |
| 25 8 0 -4.027393 -0.474451 2.066474 |
| 26 8 0 1.383342 -2.143779 -0.244239 |
| 27 6 0 5.192738 0.497878 -1.496822 |
| 28 6 0 5.216112 1.679896 -2.117729 |
| 29 6 0 5.559734 -0.775929 -2.219580 |
| 30 8 0 -6.059391 -0.464594 0.132159 |
| 31 6 0 1.899191 4.419001 0.373491 |
| 32 8 0 3.423066 -0.959087 1.512986 |
| 33 8 0 4.552496 -2.768733 0.763923 |
| 34 6 0 3.938102 -2.207539 1.637040 |
| 35 6 0 3.626576 -2.782719 2.994236 |
| 36 8 0 -4.900437 0.553309 -2.079717 |
| 37 6 0 -4.187405 0.825693 -3.274860 |
| 38 1 0 -3.025225 -3.973666 -1.213951 |
| 39 1 0 -4.589848 -2.063220 -1.277059 |
| 40 1 0 -0.540791 3.255910 -0.111513 |
| 41 1 0 4.805682 1.315351 1.888649 |
| 42 1 0 5.804247 2.155920 0.683351 |
| 43 1 0 5.590865 -0.361176 0.397318 |
| 44 1 0 3.342310 -1.153761 -0.532155 |
| 45 1 0 0.406097 -3.502044 -0.554577 |
| 46 1 0 -3.549355 1.535713 -0.863565 |
| 47 1 0 -5.754100 1.575490 0.023033 |
| 48 1 0 -3.477104 1.919676 2.998537 |
| 49 1 0 -2.805812 1.724571 1.381042 |
| 50 1 0 -4.168076 2.831309 1.650091 |
| 51 1 0 -5.622811 0.914585 3.656732 |
| 52 1 0 -6.659097 0.093796 2.475036 |
| 53 1 0 -6.464956 1.848709 2.407138 |
| 54 1 0 -4.583328 -1.223990 1.813908 |
| 55 1 0 5.492383 1.751225 -3.165441 |
| 56 1 0 4.965448 2.609974 -1.622211 |
| 57 1 0 6.436147 -1.246801 -1.760993 |
| 58 1 0 4.761650 -1.522594 -2.177211 |
| 59 1 0 5.786896 -0.583021 -3.269674 |
| 60 1 0 -6.392740 -0.489066 -0.774242 |
| 61 1 0 1.006783 5.040664 0.285519 |
| 62 1 0 2.356206 4.603119 1.349560 |
| 63 1 0 2.626343 4.749023 -0.373669 |
| 64 1 0 2.586416 -3.120510 3.003089 |
| 65 1 0 4.275231 -3.634601 3.190501 |
| 66 1 0 3.734957 -2.031953 3.778314 |
| 67 1 0 -4.911705 0.788342 -4.088479 |
| 68 1 0 -3.734302 1.824839 -3.243125 |
| 69 1 0 -3.403055 0.083254 -3.457010 |

**C5**


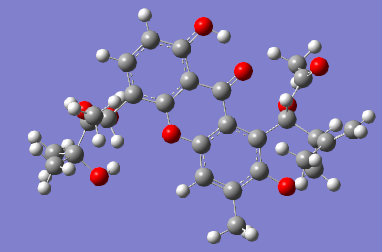


E = -1763.8112033 a.u

Standard orientation:

| Center Atomic Atomic Coordinates (Angstroms) |
| --- |
| Number Number Type X Y Z |
| 1 6 0 -2.247328 3.550939 0.635126 |
| 2 6 0 -3.240847 2.579921 0.680200 |
| 3 6 0 -2.965785 1.212584 0.577997 |
| 4 6 0 -1.622444 0.852100 0.426873 |
| 5 6 0 -0.578751 1.794684 0.377001 |
| 6 6 0 -0.912589 3.175831 0.485646 |
| 7 8 0 -1.349547 -0.475755 0.329199 |
| 8 6 0 -0.070844 -0.926270 0.166691 |
| 9 6 0 1.043001 -0.081012 0.095618 |
| 10 6 0 0.808758 1.366592 0.212885 |
| 11 6 0 0.045913 -2.315401 0.072491 |
| 12 6 0 1.280837 -2.901662 -0.102673 |
| 13 6 0 2.421356 -2.059982 -0.185865 |
| 14 6 0 2.331576 -0.674678 -0.076497 |
| 15 8 0 3.595450 -2.732821 -0.339967 |
| 16 6 0 4.753377 -1.973834 -0.715104 |
| 17 6 0 4.886427 -0.669392 0.066622 |
| 18 6 0 3.597244 0.155694 -0.143925 |
| 19 8 0 0.024124 4.130929 0.447573 |
| 20 6 0 -4.083204 0.179461 0.632567 |
| 21 6 0 -4.857551 0.054177 -0.707651 |
| 22 6 0 -5.495742 -1.341201 -0.947310 |
| 23 6 0 -6.333789 -1.833630 0.228519 |
| 24 6 0 -6.352378 -1.299258 -2.222540 |
| 25 8 0 -4.435132 -2.298041 -1.103425 |
| 26 8 0 1.726062 2.207108 0.175048 |
| 27 6 0 5.284764 -0.829439 1.529344 |
| 28 6 0 6.433154 -0.293829 1.951097 |
| 29 6 0 4.380858 -1.577660 2.479253 |
| 30 8 0 -4.000131 0.252598 -1.836646 |
| 31 6 0 1.440428 -4.391638 -0.212688 |
| 32 8 0 3.614030 0.742210 -1.482057 |
| 33 8 0 4.971407 2.407487 -0.775106 |
| 34 6 0 4.282881 1.913719 -1.632136 |
| 35 6 0 4.033706 2.498532 -2.998612 |
| 36 8 0 -5.061975 0.539914 1.605485 |
| 37 6 0 -4.663406 0.302200 2.945018 |
| 38 1 0 -2.483462 4.604964 0.721143 |
| 39 1 0 -4.273129 2.883044 0.821219 |
| 40 1 0 -0.855608 -2.915125 0.131628 |
| 41 1 0 4.683284 -1.752835 -1.785941 |
| 42 1 0 5.597634 -2.642187 -0.547784 |
| 43 1 0 5.686702 -0.101554 -0.411797 |
| 44 1 0 3.566773 0.975613 0.567084 |
| 45 1 0 0.902519 3.689386 0.348941 |
| 46 1 0 -3.654766 -0.797025 0.877686 |
| 47 1 0 -5.646060 0.817895 -0.697226 |
| 48 1 0 -6.782920 -2.794533 -0.033528 |
| 49 1 0 -5.725675 -1.982008 1.120821 |
| 50 1 0 -7.131374 -1.129269 0.471134 |
| 51 1 0 -6.744505 -2.297230 -2.430597 |
| 52 1 0 -5.766835 -0.969142 -3.081882 |
| 53 1 0 -7.198919 -0.615277 -2.106956 |
| 54 1 0 -3.825818 -1.939849 -1.764307 |
| 55 1 0 6.755531 -0.383462 2.984070 |
| 56 1 0 7.085374 0.266322 1.288716 |
| 57 1 0 3.394432 -1.108531 2.554567 |
| 58 1 0 4.213440 -2.608039 2.153830 |
| 59 1 0 4.811652 -1.604001 3.481918 |
| 60 1 0 -3.501165 1.070563 -1.721622 |
| 61 1 0 0.474768 -4.895063 -0.148703 |
| 62 1 0 1.915526 -4.667201 -1.157991 |
| 63 1 0 2.087079 -4.778758 0.579818 |
| 64 1 0 3.047293 2.970858 -3.005085 |
| 65 1 0 4.786349 3.253725 -3.218215 |
| 66 1 0 4.033712 1.725333 -3.768231 |
| 67 1 0 -5.499257 0.596691 3.579566 |
| 68 1 0 -4.443580 -0.760404 3.110934 |
| 69 1 0 -3.781027 0.892780 3.218264 |

**C6**


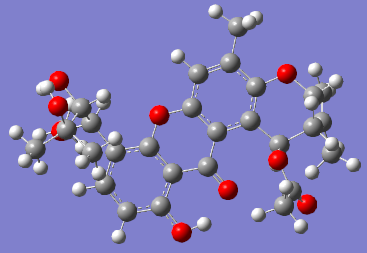


E = -1763.8109974 a.u

Standard orientation:

| Center Atomic Atomic Coordinates (Angstroms) |
| --- |
| Number Number Type X Y Z |
| 1 6 0 -2.559908 -3.005001 -1.072020 |
| 2 6 0 -3.444148 -1.931932 -1.090259 |
| 3 6 0 -3.035036 -0.616855 -0.867396 |
| 4 6 0 -1.671763 -0.414806 -0.636700 |
| 5 6 0 -0.735990 -1.463094 -0.604301 |
| 6 6 0 -1.205544 -2.790328 -0.825923 |
| 7 8 0 -1.271156 0.871169 -0.433693 |
| 8 6 0 0.042777 1.173524 -0.203492 |
| 9 6 0 1.056801 0.210462 -0.141833 |
| 10 6 0 0.676270 -1.196377 -0.345194 |
| 11 6 0 0.303746 2.533868 -0.025859 |
| 12 6 0 1.585477 2.973670 0.226792 |
| 13 6 0 2.625570 2.010734 0.302696 |
| 14 6 0 2.392267 0.651686 0.108592 |
| 15 8 0 3.853111 2.542594 0.550784 |
| 16 6 0 4.931698 1.642634 0.849031 |
| 17 6 0 4.918526 0.395793 -0.020946 |
| 18 6 0 3.558747 -0.311205 0.170199 |
| 19 8 0 -0.377126 -3.842448 -0.810135 |
| 20 6 0 -4.002778 0.554163 -0.896670 |
| 21 6 0 -4.404430 1.169594 0.468337 |
| 22 6 0 -4.999728 0.297965 1.618273 |
| 23 6 0 -6.257026 -0.484350 1.218808 |
| 24 6 0 -3.962021 -0.621239 2.259731 |
| 25 8 0 -5.343063 1.233482 2.656053 |
| 26 8 0 1.496024 -2.132492 -0.303841 |
| 27 6 0 5.264906 0.591822 -1.492285 |
| 28 6 0 5.268417 1.782106 -2.097500 |
| 29 6 0 5.658524 -0.665511 -2.229783 |
| 30 8 0 -5.303460 2.252798 0.190172 |
| 31 6 0 1.895640 4.430035 0.428029 |
| 32 8 0 3.507080 -0.935417 1.489814 |
| 33 8 0 4.672260 -2.714649 0.722735 |
| 34 6 0 4.047227 -2.174695 1.601512 |
| 35 6 0 3.747703 -2.769868 2.952876 |
| 36 8 0 -5.211347 0.233545 -1.588712 |
| 37 6 0 -5.106519 0.298054 -3.005144 |
| 38 1 0 -2.901007 -4.018296 -1.248229 |
| 39 1 0 -4.494176 -2.116070 -1.286686 |
| 40 1 0 -0.523394 3.232658 -0.083718 |
| 41 1 0 4.851964 1.356712 1.902833 |
| 42 1 0 5.838346 2.231179 0.711836 |
| 43 1 0 5.670326 -0.285356 0.392119 |
| 44 1 0 3.440294 -1.104137 -0.558439 |
| 45 1 0 0.533141 -3.507399 -0.626022 |
| 46 1 0 -3.518178 1.383774 -1.428437 |
| 47 1 0 -3.510563 1.641777 0.881239 |
| 48 1 0 -6.668515 -0.967399 2.107867 |
| 49 1 0 -7.029506 0.175840 0.815806 |
| 50 1 0 -6.047783 -1.249740 0.469636 |
| 51 1 0 -4.370380 -1.003457 3.198274 |
| 52 1 0 -3.048681 -0.070893 2.499292 |
| 53 1 0 -3.707053 -1.470449 1.626649 |
| 54 1 0 -5.780007 1.984031 2.227694 |
| 55 1 0 5.547185 1.872556 -3.143027 |
| 56 1 0 4.998981 2.700907 -1.590928 |
| 57 1 0 6.541030 -1.126574 -1.772929 |
| 58 1 0 4.873887 -1.426918 -2.201434 |
| 59 1 0 5.887422 -0.454691 -3.276026 |
| 60 1 0 -5.939489 1.926960 -0.462789 |
| 61 1 0 0.992705 5.037232 0.349273 |
| 62 1 0 2.347928 4.604573 1.407996 |
| 63 1 0 2.618296 4.785308 -0.311893 |
| 64 1 0 2.717900 -3.138258 2.955212 |
| 65 1 0 4.419652 -3.604674 3.144044 |
| 66 1 0 3.831900 -2.023305 3.743803 |
| 67 1 0 -6.090655 0.054598 -3.405204 |
| 68 1 0 -4.821985 1.305991 -3.331209 |
| 69 1 0 -4.374604 -0.420235 -3.389450 |

**C7**

14R, 15R, 20S, 25R-4 for ECD


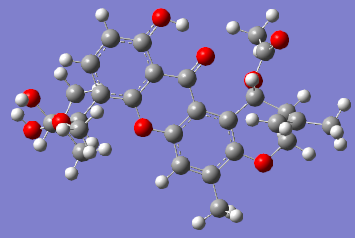


E =- 1763.8106037 a.u

Standard orientation:

| Center Atomic Atomic Coordinates (Angstroms) |
| --- |
| Number Number Type X Y Z |
| 1 6 0 -2.719921 2.937856 1.073591 |
| 2 6 0 -3.578267 1.847389 1.067466 |
| 3 6 0 -3.140387 0.540818 0.821357 |
| 4 6 0 -1.770719 0.374089 0.597263 |
| 5 6 0 -0.857067 1.446236 0.593488 |
| 6 6 0 -1.358130 2.757286 0.837195 |
| 7 8 0 -1.326625 -0.896504 0.368601 |
| 8 6 0 -0.003446 -1.158651 0.147903 |
| 9 6 0 0.984768 -0.168651 0.113774 |
| 10 6 0 0.564343 1.222186 0.347123 |
| 11 6 0 0.298025 -2.509158 -0.045841 |
| 12 6 0 1.593394 -2.909784 -0.294861 |
| 13 6 0 2.605891 -1.917385 -0.349879 |
| 14 6 0 2.334344 -0.568339 -0.131353 |
| 15 8 0 3.850886 -2.407771 -0.601924 |
| 16 6 0 4.868457 -1.461641 -0.946917 |
| 17 6 0 4.855895 -0.257152 -0.006711 |
| 18 6 0 3.482230 0.421558 -0.136029 |
| 19 8 0 -0.554745 3.827763 0.851631 |
| 20 6 0 -4.097829 -0.635296 0.857598 |
| 21 6 0 -5.213612 -0.696560 -0.211644 |
| 22 6 0 -4.843904 -0.740191 -1.713427 |
| 23 6 0 -4.235866 0.561112 -2.251308 |
| 24 6 0 -3.953505 -1.939897 -2.026249 |
| 25 8 0 -6.078175 -0.993598 -2.409724 |
| 26 8 0 1.363126 2.176859 0.342125 |
| 27 6 0 5.279956 -0.606331 1.413091 |
| 28 6 0 4.437138 -0.704774 2.442239 |
| 29 6 0 6.763915 -0.813732 1.593617 |
| 30 8 0 -6.138611 0.383104 -0.046264 |
| 31 6 0 1.948656 -4.353703 -0.510150 |
| 32 8 0 3.412631 1.124012 -1.414012 |
| 33 8 0 4.542952 2.878641 -0.543095 |
| 34 6 0 3.918648 2.383342 -1.447399 |
| 35 6 0 3.582951 3.059566 -2.751331 |
| 36 8 0 -4.843178 -0.654535 2.089725 |
| 37 6 0 -4.078739 -0.968525 3.241668 |
| 38 1 0 -3.084438 3.940541 1.263230 |
| 39 1 0 -4.631823 2.007305 1.256498 |
| 40 1 0 -0.508643 -3.232565 -0.002659 |
| 41 1 0 4.699543 -1.128555 -1.976645 |
| 42 1 0 5.801374 -2.022396 -0.910145 |
| 43 1 0 5.590287 0.456592 -0.393791 |
| 44 1 0 3.363094 1.163911 0.646344 |
| 45 1 0 0.366493 3.516722 0.678305 |
| 46 1 0 -3.525137 -1.564929 0.785381 |
| 47 1 0 -5.741931 -1.637773 -0.012981 |
| 48 1 0 -4.167584 0.488383 -3.339062 |
| 49 1 0 -4.863366 1.419355 -2.003641 |
| 50 1 0 -3.236706 0.750013 -1.855616 |
| 51 1 0 -3.853733 -2.039782 -3.109156 |
| 52 1 0 -4.397595 -2.864094 -1.647019 |
| 53 1 0 -2.955105 -1.825138 -1.600192 |
| 54 1 0 -6.718890 -0.336659 -2.103762 |
| 55 1 0 4.797351 -0.944232 3.437928 |
| 56 1 0 3.368469 -0.550668 2.349396 |
| 57 1 0 7.151550 -1.604374 0.941785 |
| 58 1 0 7.318264 0.098344 1.344888 |
| 59 1 0 7.007402 -1.086294 2.621940 |
| 60 1 0 -6.530102 0.294000 0.831837 |
| 61 1 0 1.065346 -4.990118 -0.438187 |
| 62 1 0 2.406756 -4.504070 -1.491511 |
| 63 1 0 2.682556 -4.691885 0.226587 |
| 64 1 0 2.543521 3.397705 -2.714729 |
| 65 1 0 4.228930 3.924042 -2.894140 |
| 66 1 0 3.675974 2.370871 -3.592283 |
| 67 1 0 -4.769574 -0.976378 4.084649 |
| 68 1 0 -3.614304 -1.958715 3.149072 |
| 69 1 0 -3.297363 -0.222960 3.425540 |

**C8**


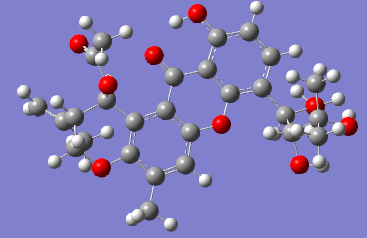


E = -1763.8110286 a.u

Standard orientation:

| Center Atomic Atomic Coordinates (Angstroms) |
| --- |
| Number Number Type X Y Z |
| 1 6 0 2.435202 3.182743 -0.950014 |
| 2 6 0 3.349175 2.134463 -0.979106 |
| 3 6 0 2.979218 0.804762 -0.777255 |
| 4 6 0 1.617725 0.565252 -0.564474 |
| 5 6 0 0.651672 1.585635 -0.522615 |
| 6 6 0 1.085701 2.928603 -0.718154 |
| 7 8 0 1.248932 -0.735941 -0.401379 |
| 8 6 0 -0.056691 -1.080554 -0.191538 |
| 9 6 0 -1.098537 -0.148382 -0.119772 |
| 10 6 0 -0.756454 1.273260 -0.292503 |
| 11 6 0 -0.277503 -2.452310 -0.047377 |
| 12 6 0 -1.548767 -2.934782 0.178137 |
| 13 6 0 -2.617892 -2.004489 0.260192 |
| 14 6 0 -2.424061 -0.634179 0.103231 |
| 15 8 0 -3.836218 -2.578941 0.462322 |
| 16 6 0 -4.922767 -1.721048 0.838293 |
| 17 6 0 -4.974241 -0.434890 0.017805 |
| 18 6 0 -3.620283 0.293765 0.170152 |
| 19 8 0 0.228058 3.957600 -0.691435 |
| 20 6 0 3.961693 -0.359328 -0.845460 |
| 21 6 0 4.245758 -1.094133 0.496244 |
| 22 6 0 5.364778 -0.511770 1.414461 |
| 23 6 0 5.254928 0.995220 1.653957 |
| 24 6 0 5.360768 -1.250478 2.754425 |
| 25 8 0 6.634806 -0.828654 0.804991 |
| 26 8 0 -1.604714 2.182876 -0.251838 |
| 27 6 0 -5.420734 -0.608325 -1.429366 |
| 28 6 0 -6.531983 0.005703 -1.843590 |
| 29 6 0 -4.603380 -1.457131 -2.373133 |
| 30 8 0 4.564909 -2.450617 0.198987 |
| 31 6 0 -1.818704 -4.404009 0.341418 |
| 32 8 0 -3.558667 0.925868 1.485874 |
| 33 8 0 -4.793718 2.669522 0.744470 |
| 34 6 0 -4.127087 2.152706 1.605187 |
| 35 6 0 -3.798682 2.765342 2.942349 |
| 36 8 0 5.206973 0.031145 -1.435989 |
| 37 6 0 5.219527 -0.006736 -2.858118 |
| 38 1 0 2.750214 4.207268 -1.109262 |
| 39 1 0 4.392426 2.353785 -1.173738 |
| 40 1 0 0.572736 -3.122465 -0.110225 |
| 41 1 0 -4.810228 -1.473769 1.899719 |
| 42 1 0 -5.819871 -2.326438 0.711073 |
| 43 1 0 -5.715530 0.208363 0.495966 |
| 44 1 0 -3.546045 1.084099 -0.570577 |
| 45 1 0 -0.675384 3.593560 -0.531387 |
| 46 1 0 3.513683 -1.139429 -1.471106 |
| 47 1 0 3.307189 -1.116969 1.054207 |
| 48 1 0 6.005959 1.298857 2.387203 |
| 49 1 0 5.430092 1.564498 0.741714 |
| 50 1 0 4.270023 1.271139 2.040543 |
| 51 1 0 6.229439 -0.944140 3.341210 |
| 52 1 0 5.402998 -2.330860 2.614218 |
| 53 1 0 4.458537 -1.012873 3.324400 |
| 54 1 0 6.652238 -0.383285 -0.055927 |
| 55 1 0 -6.885727 -0.089545 -2.865753 |
| 56 1 0 -7.120322 0.637619 -1.185982 |
| 57 1 0 -3.581515 -1.078208 -2.477307 |
| 58 1 0 -4.519429 -2.489262 -2.021757 |
| 59 1 0 -5.053870 -1.472439 -3.367305 |
| 60 1 0 5.532631 -2.500482 0.150095 |
| 61 1 0 -0.896267 -4.981697 0.267750 |
| 62 1 0 -2.284809 -4.613407 1.307981 |
| 63 1 0 -2.516031 -4.764624 -0.419977 |
| 64 1 0 -2.783478 3.170384 2.903279 |
| 65 1 0 -4.492091 3.577172 3.154979 |
| 66 1 0 -3.826642 2.021030 3.739346 |
| 67 1 0 6.206760 0.328069 -3.176694 |
| 68 1 0 5.048730 -1.026478 -3.220832 |
| 69 1 0 4.460428 0.658197 -3.284150 |

**C9**


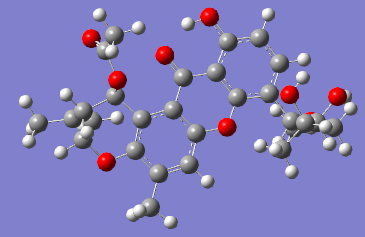


E = -1763.8104693 a.u

Standard orientation:

| Center Atomic Atomic Coordinates (Angstroms) |
| --- |
| Number Number Type X Y Z |
| 1 6 0 2.743603 2.917779 -1.106554 |
| 2 6 0 3.597354 1.822940 -1.100424 |
| 3 6 0 3.159362 0.519764 -0.842267 |
| 4 6 0 1.789099 0.359220 -0.620570 |
| 5 6 0 0.880286 1.434837 -0.612630 |
| 6 6 0 1.384386 2.744313 -0.856010 |
| 7 8 0 1.337069 -0.911167 -0.408646 |
| 8 6 0 0.014151 -1.168417 -0.187156 |
| 9 6 0 -0.967657 -0.173270 -0.135141 |
| 10 6 0 -0.539921 1.217187 -0.357911 |
| 11 6 0 -0.296044 -2.519969 -0.013682 |
| 12 6 0 -1.592839 -2.915998 0.234857 |
| 13 6 0 -2.598030 -1.917974 0.311012 |
| 14 6 0 -2.318321 -0.567741 0.111806 |
| 15 8 0 -3.845681 -2.403826 0.562460 |
| 16 6 0 -4.852541 -1.456401 0.933166 |
| 17 6 0 -4.839099 -0.235037 0.014769 |
| 18 6 0 -3.458688 0.430300 0.141581 |
| 19 8 0 0.585336 3.819034 -0.863262 |
| 20 6 0 4.120184 -0.655734 -0.850278 |
| 21 6 0 5.185696 -0.712688 0.273820 |
| 22 6 0 4.702369 -0.743861 1.742496 |
| 23 6 0 3.697044 -1.867469 1.986821 |
| 24 6 0 5.918194 -0.921451 2.667480 |
| 25 8 0 4.025606 0.475530 2.071087 |
| 26 8 0 -1.334350 2.175774 -0.339776 |
| 27 6 0 -5.280477 -0.555175 -1.406573 |
| 28 6 0 -4.447862 -0.650291 -2.444288 |
| 29 6 0 -6.768862 -0.737928 -1.576967 |
| 30 8 0 6.077355 0.397343 0.159512 |
| 31 6 0 -1.956904 -4.360874 0.428965 |
| 32 8 0 -3.370928 1.112173 1.429524 |
| 33 8 0 -4.495944 2.889922 0.599543 |
| 34 6 0 -3.864036 2.375402 1.487891 |
| 35 6 0 -3.503921 3.028599 2.797013 |
| 36 8 0 4.928452 -0.653408 -2.043331 |
| 37 6 0 4.224226 -0.941049 -3.240125 |
| 38 1 0 3.111013 3.917460 -1.306543 |
| 39 1 0 4.647659 1.983820 -1.306076 |
| 40 1 0 0.505524 -3.248027 -0.071566 |
| 41 1 0 -4.672290 -1.142829 1.967164 |
| 42 1 0 -5.790332 -2.009030 0.894392 |
| 43 1 0 -5.563419 0.477669 0.422175 |
| 44 1 0 -3.340593 1.183762 -0.630209 |
| 45 1 0 -0.334748 3.511630 -0.678300 |
| 46 1 0 3.550336 -1.589185 -0.820800 |
| 47 1 0 5.745449 -1.640218 0.089896 |
| 48 1 0 3.432322 -1.891851 3.046144 |
| 49 1 0 2.779962 -1.718761 1.418360 |
| 50 1 0 4.123139 -2.839666 1.722776 |
| 51 1 0 5.583895 -0.903585 3.707051 |
| 52 1 0 6.644061 -0.120400 2.521075 |
| 53 1 0 6.426621 -1.873601 2.485446 |
| 54 1 0 4.593258 1.211947 1.806159 |
| 55 1 0 -4.819821 -0.868974 -3.440478 |
| 56 1 0 -3.376321 -0.513484 -2.357711 |
| 57 1 0 -7.162619 -1.531019 -0.931722 |
| 58 1 0 -7.307060 0.179070 -1.311381 |
| 59 1 0 -7.025358 -0.993391 -2.606554 |
| 60 1 0 6.421579 0.397749 -0.743161 |
| 61 1 0 -1.078266 -5.001970 0.341534 |
| 62 1 0 -2.410216 -4.524177 1.410511 |
| 63 1 0 -2.697184 -4.682462 -0.308845 |
| 64 1 0 -2.458669 3.347537 2.755193 |
| 65 1 0 -4.132963 3.902417 2.957542 |
| 66 1 0 -3.601437 2.330313 3.629586 |
| 67 1 0 4.957968 -0.933895 -4.046087 |
| 68 1 0 3.754333 -1.931716 -3.191197 |
| 69 1 0 3.454341 -0.190194 -3.447857 |

**C10**


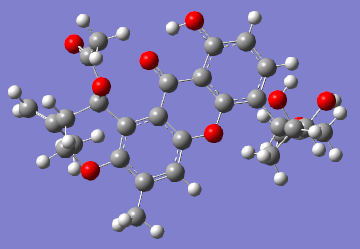


E = -1763.8091874 a.u

Standard orientation:

| Center Atomic Atomic Coordinates (Angstroms) |
| --- |
| Number Number Type X Y Z |
| 1 6 0 2.690426 2.936728 -1.116930 |
| 2 6 0 3.556088 1.851344 -1.109415 |
| 3 6 0 3.132968 0.544339 -0.845576 |
| 4 6 0 1.765378 0.370051 -0.618762 |
| 5 6 0 0.844503 1.435300 -0.613318 |
| 6 6 0 1.333670 2.749394 -0.862681 |
| 7 8 0 1.327997 -0.904117 -0.398363 |
| 8 6 0 0.008730 -1.175226 -0.173289 |
| 9 6 0 -0.985659 -0.192242 -0.129672 |
| 10 6 0 -0.573029 1.202618 -0.357101 |
| 11 6 0 -0.283587 -2.528748 0.014497 |
| 12 6 0 -1.576013 -2.939488 0.261477 |
| 13 6 0 -2.595485 -1.954574 0.319669 |
| 14 6 0 -2.332302 -0.602123 0.115714 |
| 15 8 0 -3.840665 -2.459353 0.547475 |
| 16 6 0 -4.878662 -1.534139 0.901127 |
| 17 6 0 -4.869418 -0.275285 0.038057 |
| 18 6 0 -3.479444 0.387481 0.159713 |
| 19 8 0 0.523131 3.815320 -0.872314 |
| 20 6 0 4.106184 -0.620961 -0.854447 |
| 21 6 0 5.175143 -0.665712 0.266767 |
| 22 6 0 4.696192 -0.702660 1.736693 |
| 23 6 0 3.705046 -1.838222 1.983616 |
| 24 6 0 5.916549 -0.865694 2.658324 |
| 25 8 0 4.005507 0.508393 2.067098 |
| 26 8 0 -1.377545 2.152534 -0.341704 |
| 27 6 0 -5.335340 -0.475533 -1.399337 |
| 28 6 0 -6.434959 0.155197 -1.819457 |
| 29 6 0 -4.550189 -1.370883 -2.327228 |
| 30 8 0 6.053821 0.454414 0.150220 |
| 31 6 0 -1.919202 -4.387430 0.472360 |
| 32 8 0 -3.376584 1.055101 1.454372 |
| 33 8 0 -4.517409 2.841219 0.664742 |
| 34 6 0 -3.873937 2.315521 1.537657 |
| 35 6 0 -3.501211 2.949102 2.853039 |
| 36 8 0 4.911187 -0.611250 -2.049569 |
| 37 6 0 4.207229 -0.907729 -3.244335 |
| 38 1 0 3.046347 3.939680 -1.321239 |
| 39 1 0 4.603996 2.022810 -1.318695 |
| 40 1 0 0.528118 -3.246302 -0.031777 |
| 41 1 0 -4.744864 -1.257545 1.952786 |
| 42 1 0 -5.806861 -2.095919 0.799503 |
| 43 1 0 -5.573723 0.420670 0.498092 |
| 44 1 0 -3.370255 1.149777 -0.605622 |
| 45 1 0 -0.393192 3.499060 -0.683984 |
| 46 1 0 3.546212 -1.560308 -0.822590 |
| 47 1 0 5.744885 -1.586803 0.081225 |
| 48 1 0 3.443882 -1.866293 3.043721 |
| 49 1 0 2.784599 -1.699742 1.418060 |
| 50 1 0 4.141726 -2.805184 1.717666 |
| 51 1 0 5.584962 -0.851858 3.698807 |
| 52 1 0 6.632405 -0.056095 2.509826 |
| 53 1 0 6.435791 -1.811681 2.474766 |
| 54 1 0 4.564443 1.251667 1.802868 |
| 55 1 0 -6.802919 0.039877 -2.834524 |
| 56 1 0 -6.999502 0.820545 -1.174040 |
| 57 1 0 -3.520845 -1.019828 -2.453065 |
| 58 1 0 -4.487471 -2.394395 -1.947229 |
| 59 1 0 -5.012281 -1.403121 -3.315696 |
| 60 1 0 6.396974 0.458029 -0.752820 |
| 61 1 0 -1.028905 -5.014717 0.405624 |
| 62 1 0 -2.382287 -4.543886 1.450375 |
| 63 1 0 -2.644241 -4.734288 -0.269238 |
| 64 1 0 -2.456984 3.270645 2.805923 |
| 65 1 0 -4.130138 3.819119 3.033387 |
| 66 1 0 -3.589314 2.237883 3.675647 |
| 67 1 0 4.939123 -0.894720 -4.051886 |
| 68 1 0 3.747210 -1.902901 -3.192820 |
| 69 1 0 3.429595 -0.164800 -3.451683 |

1. Lowest energy conformers (14*R*,15*S*,20*S*,25*R*)-**2** for ECD calcation.

**C1**


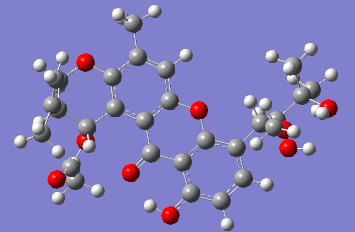


E = -1763.816384 a.u

Standard orientation:

| Center Atomic Atomic Coordinates (Angstroms) |
| --- |
| Number Number Type X Y Z |
| 1 6 0 2.229197 -3.398804 0.630152 |
| 2 6 0 3.200176 -2.406153 0.650933 |
| 3 6 0 2.890010 -1.048755 0.531604 |
| 4 6 0 1.542066 -0.723611 0.371966 |
| 5 6 0 0.518497 -1.690229 0.340820 |
| 6 6 0 0.886352 -3.060229 0.476055 |
| 7 8 0 1.236580 0.599808 0.233119 |
| 8 6 0 -0.056099 1.014792 0.075441 |
| 9 6 0 -1.148473 0.141274 0.031886 |
| 10 6 0 -0.875867 -1.299722 0.168242 |
| 11 6 0 -0.212760 2.397604 -0.046693 |
| 12 6 0 -1.463348 2.949120 -0.224785 |
| 13 6 0 -2.581480 2.077426 -0.280811 |
| 14 6 0 -2.452752 0.698052 -0.139073 |
| 15 8 0 -3.770950 2.716125 -0.454598 |
| 16 6 0 -4.932776 1.917015 -0.723707 |
| 17 6 0 -4.982408 0.648100 0.112473 |
| 18 6 0 -3.697948 -0.162841 -0.169510 |
| 19 8 0 -0.027719 -4.039539 0.458701 |
| 20 6 0 3.967350 0.019057 0.545511 |
| 21 6 0 4.659476 0.170900 -0.830586 |
| 22 6 0 5.787377 1.248487 -0.866345 |
| 23 6 0 6.208480 1.513138 -2.311962 |
| 24 6 0 5.385439 2.560755 -0.185491 |
| 25 8 0 6.950227 0.689136 -0.222355 |
| 26 8 0 -1.775173 -2.161047 0.137792 |
| 27 6 0 -5.233904 0.829458 1.604728 |
| 28 6 0 -5.108336 1.998386 2.237909 |
| 29 6 0 -5.689844 -0.412347 2.332614 |
| 30 8 0 5.178894 -1.067801 -1.284147 |
| 31 6 0 -1.661841 4.432067 -0.364678 |
| 32 8 0 -3.764039 -0.743160 -1.508130 |
| 33 8 0 -5.041649 -2.441952 -0.737918 |
| 34 6 0 -4.410689 -1.929402 -1.628767 |
| 35 6 0 -4.219025 -2.506300 -3.007338 |
| 36 8 0 4.989711 -0.255711 1.516840 |
| 37 6 0 4.559699 -0.151954 2.867880 |
| 38 1 0 2.491866 -4.445712 0.725666 |
| 39 1 0 4.240524 -2.683430 0.766596 |
| 40 1 0 0.670181 3.025694 -0.003772 |
| 41 1 0 -4.925853 1.656086 -1.787019 |
| 42 1 0 -5.780287 2.573330 -0.527941 |
| 43 1 0 -5.809284 0.044297 -0.276619 |
| 44 1 0 -3.610170 -0.987244 0.527838 |
| 45 1 0 -0.914921 -3.622595 0.343465 |
| 46 1 0 3.502118 0.978292 0.794601 |
| 47 1 0 3.875139 0.458917 -1.538449 |
| 48 1 0 7.088705 2.159573 -2.325037 |
| 49 1 0 6.456023 0.584011 -2.826125 |
| 50 1 0 5.407024 2.009124 -2.866045 |
| 51 1 0 6.178778 3.299434 -0.320261 |
| 52 1 0 5.231951 2.433767 0.888623 |
| 53 1 0 4.465933 2.970733 -0.615544 |
| 54 1 0 6.692918 0.468374 0.686741 |
| 55 1 0 -5.324130 2.080970 3.298865 |
| 56 1 0 -4.791031 2.906283 1.739488 |
| 57 1 0 -6.627721 -0.790563 1.911455 |
| 58 1 0 -4.970669 -1.232034 2.247271 |
| 59 1 0 -5.849207 -0.212039 3.393768 |
| 60 1 0 6.100279 -1.106777 -0.983714 |
| 61 1 0 -0.709199 4.961811 -0.314689 |
| 62 1 0 -2.146825 4.676848 -1.313453 |
| 63 1 0 -2.314560 4.818837 0.422917 |
| 64 1 0 -3.224760 -2.958475 -3.064035 |
| 65 1 0 -4.966091 -3.276411 -3.191279 |
| 66 1 0 -4.272642 -1.731832 -3.773759 |
| 67 1 0 5.430394 -0.349488 3.493009 |
| 68 1 0 4.180190 0.854813 3.084667 |
| 69 1 0 3.778852 -0.883190 3.097513 |

**C2**


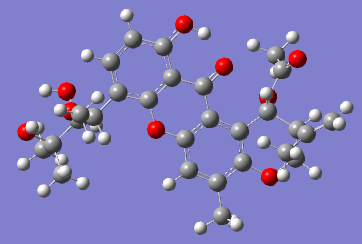


E = -1763.814321 a.u

Standard orientation:

| Center Atomic Atomic Coordinates (Angstroms) |
| --- |
| Number Number Type X Y Z |
| 1 6 0 -2.246675 3.381501 0.710111 |
| 2 6 0 -3.212959 2.384310 0.701016 |
| 3 6 0 -2.895310 1.031088 0.554731 |
| 4 6 0 -1.544728 0.716131 0.398733 |
| 5 6 0 -0.525688 1.687811 0.395936 |
| 6 6 0 -0.900832 3.052621 0.559828 |
| 7 8 0 -1.231115 -0.603334 0.237060 |
| 8 6 0 0.064051 -1.008246 0.076609 |
| 9 6 0 1.151841 -0.128218 0.050704 |
| 10 6 0 0.872102 1.308092 0.224668 |
| 11 6 0 0.228081 -2.388415 -0.066490 |
| 12 6 0 1.482109 -2.930544 -0.249281 |
| 13 6 0 2.595151 -2.051769 -0.290731 |
| 14 6 0 2.459618 -0.674717 -0.130493 |
| 15 8 0 3.791513 -2.681517 -0.458644 |
| 16 6 0 4.925610 -1.872854 -0.801660 |
| 17 6 0 5.013836 -0.594402 0.027627 |
| 18 6 0 3.699415 0.196887 -0.156964 |
| 19 8 0 0.008461 4.036165 0.572518 |
| 20 6 0 -3.967800 -0.041660 0.539406 |
| 21 6 0 -4.652307 -0.166366 -0.843346 |
| 22 6 0 -5.777200 -1.245542 -0.908624 |
| 23 6 0 -6.189685 -1.478982 -2.362179 |
| 24 6 0 -5.376289 -2.572007 -0.255340 |
| 25 8 0 -6.944861 -0.703130 -0.259047 |
| 26 8 0 1.767828 2.172357 0.229163 |
| 27 6 0 5.412218 -0.796081 1.485141 |
| 28 6 0 6.541430 -0.240693 1.932280 |
| 29 6 0 4.528580 -1.606628 2.402475 |
| 30 8 0 -5.172575 1.080391 -1.273346 |
| 31 6 0 1.689449 -4.410284 -0.409824 |
| 32 8 0 3.702629 0.837497 -1.469527 |
| 33 8 0 5.006221 2.512298 -0.688198 |
| 34 6 0 4.333324 2.035775 -1.567080 |
| 35 6 0 4.064030 2.672830 -2.905834 |
| 36 8 0 -4.996094 0.207454 1.511304 |
| 37 6 0 -4.572860 0.073990 2.861825 |
| 38 1 0 -2.515031 4.424754 0.827263 |
| 39 1 0 -4.255456 2.654079 0.814854 |
| 40 1 0 -0.651899 -3.021363 -0.035984 |
| 41 1 0 4.852087 -1.615658 -1.864183 |
| 42 1 0 5.790449 -2.519377 -0.654804 |
| 43 1 0 5.796826 0.016255 -0.425986 |
| 44 1 0 3.639677 0.985516 0.586838 |
| 45 1 0 0.899312 3.626174 0.460686 |
| 46 1 0 -3.499181 -1.003903 0.770152 |
| 47 1 0 -3.863295 -0.437049 -1.552882 |
| 48 1 0 -7.067760 -2.127619 -2.394534 |
| 49 1 0 -6.437477 -0.539386 -2.856758 |
| 50 1 0 -5.383668 -1.959880 -2.922895 |
| 51 1 0 -6.166194 -3.309693 -0.413570 |
| 52 1 0 -5.231792 -2.470189 0.822707 |
| 53 1 0 -4.452269 -2.969225 -0.687714 |
| 54 1 0 -6.692714 -0.500976 0.655793 |
| 55 1 0 6.862452 -0.358553 2.962854 |
| 56 1 0 7.177887 0.364424 1.294584 |
| 57 1 0 3.527684 -1.171395 2.489887 |
| 58 1 0 4.394668 -2.629076 2.038644 |
| 59 1 0 4.956084 -1.656307 3.405686 |
| 60 1 0 -6.096095 1.110219 -0.978422 |
| 61 1 0 0.739669 -4.945993 -0.370403 |
| 62 1 0 2.178555 -4.638258 -1.360642 |
| 63 1 0 2.342587 -4.805129 0.373405 |
| 64 1 0 3.066612 3.121152 -2.887238 |
| 65 1 0 4.797050 3.455089 -3.095081 |
| 66 1 0 4.078487 1.934128 -3.708444 |
| 67 1 0 -5.447914 0.252241 3.486631 |
| 68 1 0 -4.189374 -0.935771 3.056769 |
| 69 1 0 -3.797106 0.803569 3.113067 |

1. Lowest energy conformers (14*S*,15*R*,20*S*,25*R*)-**3** for ECD calcation.

**C1**


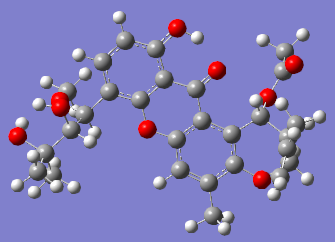


E = -1763.8164571 a.u

Standard orientation:

| Center Atomic Atomic Coordinates (Angstroms) |
| --- |
| Number Number Type X Y Z |
| 1 6 0 -2.213873 3.419623 0.626560 |
| 2 6 0 -3.196028 2.493822 0.299244 |
| 3 6 0 -2.896132 1.185446 -0.088291 |
| 4 6 0 -1.546338 0.831676 -0.116301 |
| 5 6 0 -0.511816 1.728163 0.213353 |
| 6 6 0 -0.869541 3.055339 0.589017 |
| 7 8 0 -1.250349 -0.450746 -0.479372 |
| 8 6 0 0.042543 -0.892068 -0.525367 |
| 9 6 0 1.145229 -0.087272 -0.217451 |
| 10 6 0 0.884460 1.310544 0.166405 |
| 11 6 0 0.189029 -2.227806 -0.907362 |
| 12 6 0 1.440218 -2.799582 -0.994215 |
| 13 6 0 2.569606 -1.997909 -0.686461 |
| 14 6 0 2.447827 -0.668804 -0.289189 |
| 15 8 0 3.756704 -2.656425 -0.786705 |
| 16 6 0 4.967064 -1.891921 -0.677337 |
| 17 6 0 4.892580 -0.813014 0.391957 |
| 18 6 0 3.697765 0.109269 0.063468 |
| 19 8 0 0.055540 3.969604 0.910338 |
| 20 6 0 -3.984600 0.184079 -0.425499 |
| 21 6 0 -4.576192 -0.481470 0.841026 |
| 22 6 0 -5.717215 -1.506909 0.555076 |
| 23 6 0 -5.389754 -2.464107 -0.595538 |
| 24 6 0 -6.033823 -2.298782 1.824012 |
| 25 8 0 -6.914186 -0.759866 0.257248 |
| 26 8 0 1.795300 2.114829 0.440283 |
| 27 6 0 4.863377 -1.291055 1.839072 |
| 28 6 0 4.558584 -2.540426 2.197782 |
| 29 6 0 5.249183 -0.249781 2.861991 |
| 30 8 0 -5.038834 0.490844 1.763063 |
| 31 6 0 1.628172 -4.233182 -1.403762 |
| 32 8 0 4.013419 0.933964 -1.100180 |
| 33 8 0 5.168163 2.375898 0.203292 |
| 34 6 0 4.695506 2.082677 -0.866733 |
| 35 6 0 4.764418 2.927541 -2.112437 |
| 36 8 0 -5.069907 0.787054 -1.147436 |
| 37 6 0 -4.742283 1.191635 -2.470306 |
| 38 1 0 -2.468643 4.430429 0.922352 |
| 39 1 0 -4.237592 2.787874 0.338715 |
| 40 1 0 -0.701121 -2.802943 -1.137234 |
| 41 1 0 5.165160 -1.431324 -1.650674 |
| 42 1 0 5.746753 -2.624261 -0.469266 |
| 43 1 0 5.789301 -0.194294 0.279841 |
| 44 1 0 3.506448 0.786265 0.887296 |
| 45 1 0 0.941962 3.542257 0.834152 |
| 46 1 0 -3.550387 -0.605357 -1.047632 |
| 47 1 0 -3.746476 -1.004418 1.328073 |
| 48 1 0 -6.183787 -3.208444 -0.689081 |
| 49 1 0 -5.312671 -1.942195 -1.552069 |
| 50 1 0 -4.448703 -2.994922 -0.419175 |
| 51 1 0 -6.923943 -2.910460 1.661826 |
| 52 1 0 -6.222023 -1.632857 2.666541 |
| 53 1 0 -5.203316 -2.959292 2.087127 |
| 54 1 0 -6.720043 -0.211456 -0.519153 |
| 55 1 0 4.569530 -2.837028 3.242212 |
| 56 1 0 4.290050 -3.307344 1.481569 |
| 57 1 0 4.627322 0.647521 2.796842 |
| 58 1 0 5.171903 -0.643604 3.877057 |
| 59 1 0 6.279799 0.087077 2.704187 |
| 60 1 0 -5.978838 0.629664 1.568825 |
| 61 1 0 0.670006 -4.707493 -1.621869 |
| 62 1 0 2.264548 -4.309985 -2.289459 |
| 63 1 0 2.126447 -4.806230 -0.616791 |
| 64 1 0 3.805156 3.435810 -2.245518 |
| 65 1 0 5.547046 3.676714 -2.006371 |
| 66 1 0 4.939178 2.315940 -2.998732 |
| 67 1 0 -5.651757 1.599984 -2.910730 |
| 68 1 0 -3.964068 1.960755 -2.473090 |
| 69 1 0 -4.401827 0.337992 -3.070290 |

**C2**


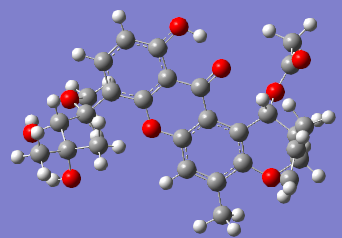


E = -1763.8149438 a.u

Standard orientation:

| Center Atomic Atomic Coordinates (Angstroms) |
| --- |
| Number Number Type X Y Z |
| 1 6 0 -2.455661 3.294976 0.736966 |
| 2 6 0 -3.402277 2.381160 0.294005 |
| 3 6 0 -3.067044 1.102284 -0.162691 |
| 4 6 0 -1.705998 0.782119 -0.177225 |
| 5 6 0 -0.705318 1.670452 0.260418 |
| 6 6 0 -1.103465 2.955741 0.728285 |
| 7 8 0 -1.365481 -0.452782 -0.636973 |
| 8 6 0 -0.062938 -0.866029 -0.667295 |
| 9 6 0 1.010918 -0.062843 -0.266654 |
| 10 6 0 0.703875 1.289419 0.222815 |
| 11 6 0 0.122268 -2.168899 -1.135452 |
| 12 6 0 1.388629 -2.705374 -1.225524 |
| 13 6 0 2.491457 -1.901144 -0.835152 |
| 14 6 0 2.328944 -0.608169 -0.345202 |
| 15 8 0 3.696532 -2.521815 -0.955888 |
| 16 6 0 4.883428 -1.734920 -0.773277 |
| 17 6 0 4.765263 -0.739468 0.370403 |
| 18 6 0 3.549629 0.172129 0.093281 |
| 19 8 0 -0.211850 3.856734 1.157641 |
| 20 6 0 -4.132485 0.164244 -0.694370 |
| 21 6 0 -5.250198 -0.194940 0.321211 |
| 22 6 0 -4.915516 -1.352042 1.304526 |
| 23 6 0 -3.611368 -1.160851 2.072347 |
| 24 6 0 -6.084267 -1.513934 2.290131 |
| 25 8 0 -4.753041 -2.555073 0.540322 |
| 26 8 0 1.582832 2.090404 0.593777 |
| 27 6 0 4.734258 -1.321462 1.778782 |
| 28 6 0 4.456404 -2.600199 2.043780 |
| 29 6 0 5.086695 -0.348504 2.878078 |
| 30 8 0 -6.404742 -0.642109 -0.391580 |
| 31 6 0 1.620783 -4.103412 -1.724843 |
| 32 8 0 3.861093 1.096501 -0.994684 |
| 33 8 0 4.950851 2.460585 0.441776 |
| 34 6 0 4.508146 2.239667 -0.658075 |
| 35 6 0 4.581412 3.179858 -1.833392 |
| 36 8 0 -4.824469 0.788016 -1.798615 |
| 37 6 0 -4.071371 0.861887 -2.998898 |
| 38 1 0 -2.741330 4.279947 1.086691 |
| 39 1 0 -4.445751 2.677857 0.283465 |
| 40 1 0 -0.750042 -2.745999 -1.421168 |
| 41 1 0 5.080422 -1.199082 -1.707617 |
| 42 1 0 5.680751 -2.459166 -0.608267 |
| 43 1 0 5.645517 -0.090130 0.315274 |
| 44 1 0 3.324617 0.775345 0.964696 |
| 45 1 0 0.686899 3.454434 1.076221 |
| 46 1 0 -3.680804 -0.765430 -1.049566 |
| 47 1 0 -5.509713 0.700301 0.901660 |
| 48 1 0 -3.512292 -1.956892 2.814095 |
| 49 1 0 -2.748667 -1.221029 1.410419 |
| 50 1 0 -3.589972 -0.202691 2.596787 |
| 51 1 0 -5.904202 -2.384357 2.924719 |
| 52 1 0 -7.029093 -1.655554 1.763329 |
| 53 1 0 -6.184204 -0.634233 2.933727 |
| 54 1 0 -5.530283 -2.622411 -0.033301 |
| 55 1 0 4.466252 -2.970550 3.064409 |
| 56 1 0 4.211963 -3.320492 1.272787 |
| 57 1 0 4.443020 0.535820 2.873640 |
| 58 1 0 5.012454 -0.817528 3.860922 |
| 59 1 0 6.109605 0.024164 2.754337 |
| 60 1 0 -6.428408 -0.146707 -1.223604 |
| 61 1 0 0.679768 -4.583290 -1.997540 |
| 62 1 0 2.279926 -4.105671 -2.597149 |
| 63 1 0 2.114014 -4.716414 -0.965297 |
| 64 1 0 3.609411 3.665727 -1.957150 |
| 65 1 0 5.334876 3.943206 -1.647465 |
| 66 1 0 4.801709 2.644004 -2.757805 |
| 67 1 0 -4.718399 1.315668 -3.749335 |
| 68 1 0 -3.178554 1.483938 -2.873785 |
| 69 1 0 -3.768487 -0.136922 -3.336217 |

**C3**


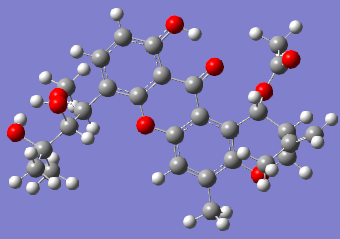


E = -1763.8143269 a.u

Standard orientation:

| Center Atomic Atomic Coordinates (Angstroms) |
| --- |
| Number Number Type X Y Z |
| 1 6 0 -2.232444 3.402795 0.694125 |
| 2 6 0 -3.208580 2.479367 0.343536 |
| 3 6 0 -2.901213 1.176864 -0.058360 |
| 4 6 0 -1.550395 0.827201 -0.076841 |
| 5 6 0 -0.521574 1.722090 0.273865 |
| 6 6 0 -0.886649 3.042495 0.665721 |
| 7 8 0 -1.247239 -0.451238 -0.449906 |
| 8 6 0 0.047640 -0.885383 -0.498535 |
| 9 6 0 1.146251 -0.077617 -0.183569 |
| 10 6 0 0.876884 1.310377 0.231495 |
| 11 6 0 0.200269 -2.217796 -0.890523 |
| 12 6 0 1.454370 -2.781829 -0.985467 |
| 13 6 0 2.580008 -1.975009 -0.677901 |
| 14 6 0 2.452916 -0.649467 -0.268821 |
| 15 8 0 3.772219 -2.626196 -0.780982 |
| 16 6 0 4.966825 -1.832065 -0.769319 |
| 17 6 0 4.946074 -0.745636 0.302759 |
| 18 6 0 3.700790 0.139561 0.073967 |
| 19 8 0 0.032177 3.954653 1.009335 |
| 20 6 0 -3.983810 0.178190 -0.421557 |
| 21 6 0 -4.590894 -0.504058 0.828475 |
| 22 6 0 -5.726787 -1.527121 0.513934 |
| 23 6 0 -5.383242 -2.467391 -0.645851 |
| 24 6 0 -6.058426 -2.337120 1.767404 |
| 25 8 0 -6.920964 -0.777579 0.211402 |
| 26 8 0 1.781425 2.109026 0.536703 |
| 27 6 0 5.092327 -1.248247 1.734494 |
| 28 6 0 6.153497 -0.867461 2.450365 |
| 29 6 0 4.041349 -2.156243 2.325974 |
| 30 8 0 -5.066721 0.455446 1.757042 |
| 31 6 0 1.649686 -4.211232 -1.406243 |
| 32 8 0 3.938985 1.019829 -1.067343 |
| 33 8 0 5.130952 2.436692 0.231462 |
| 34 6 0 4.605445 2.176575 -0.821271 |
| 35 6 0 4.583520 3.072674 -2.032762 |
| 36 8 0 -5.060586 0.788935 -1.149748 |
| 37 6 0 -4.716346 1.213155 -2.462227 |
| 38 1 0 -2.492979 4.408628 1.001601 |
| 39 1 0 -4.251259 2.770225 0.376190 |
| 40 1 0 -0.687447 -2.795612 -1.123123 |
| 41 1 0 5.078054 -1.369756 -1.756495 |
| 42 1 0 5.779204 -2.542927 -0.620013 |
| 43 1 0 5.808332 -0.104328 0.110456 |
| 44 1 0 3.537836 0.772876 0.940649 |
| 45 1 0 0.920543 3.529966 0.942203 |
| 46 1 0 -3.540960 -0.603172 -1.047868 |
| 47 1 0 -3.766921 -1.032521 1.319412 |
| 48 1 0 -6.175606 -3.210461 -0.761241 |
| 49 1 0 -5.293066 -1.931492 -1.593469 |
| 50 1 0 -4.444516 -3.000626 -0.464369 |
| 51 1 0 -6.944903 -2.948369 1.584823 |
| 52 1 0 -6.259365 -1.683262 2.616428 |
| 53 1 0 -5.230071 -2.999556 2.032520 |
| 54 1 0 -6.718178 -0.218760 -0.555288 |
| 55 1 0 6.297492 -1.201369 3.473465 |
| 56 1 0 6.907741 -0.197105 2.050734 |
| 57 1 0 3.934409 -3.081399 1.752757 |
| 58 1 0 4.295074 -2.421685 3.353972 |
| 59 1 0 3.055599 -1.680043 2.340481 |
| 60 1 0 -6.004444 0.595424 1.552840 |
| 61 1 0 0.693747 -4.689824 -1.624757 |
| 62 1 0 2.283469 -4.276986 -2.294667 |
| 63 1 0 2.154825 -4.787360 -0.625916 |
| 64 1 0 3.601276 3.548639 -2.101423 |
| 65 1 0 5.343095 3.845875 -1.931797 |
| 66 1 0 4.738949 2.503671 -2.950616 |
| 67 1 0 -5.621292 1.624215 -2.909401 |
| 68 1 0 -3.941291 1.985234 -2.443692 |
| 69 1 0 -4.364283 0.369303 -3.069322 |

1. Lowest energy conformers (14*S*,15*S*,20*S*,25*R*)-**4** for ECD calcation.

**C1**


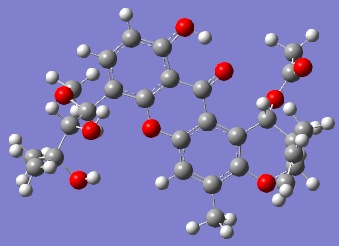


E = -1763.8133412 a.u

Standard orientation:

| Center Atomic Atomic Coordinates (Angstroms) |
| --- |
| Number Number Type X Y Z |
| 1 6 0 -2.207241 3.538963 0.738835 |
| 2 6 0 -3.216456 2.652435 0.381668 |
| 3 6 0 -2.959050 1.342422 -0.034324 |
| 4 6 0 -1.616544 0.950558 -0.081225 |
| 5 6 0 -0.557551 1.808692 0.269831 |
| 6 6 0 -0.874042 3.133837 0.687782 |
| 7 8 0 -1.359932 -0.318052 -0.494842 |
| 8 6 0 -0.082742 -0.798066 -0.556927 |
| 9 6 0 1.045446 -0.036126 -0.230480 |
| 10 6 0 0.828758 1.353214 0.198759 |
| 11 6 0 0.018108 -2.126301 -0.977635 |
| 12 6 0 1.251099 -2.732081 -1.088573 |
| 13 6 0 2.407369 -1.973717 -0.765898 |
| 14 6 0 2.329633 -0.654508 -0.328255 |
| 15 8 0 3.573109 -2.663950 -0.894361 |
| 16 6 0 4.806418 -1.938571 -0.775171 |
| 17 6 0 4.774104 -0.890068 0.326534 |
| 18 6 0 3.603700 0.075437 0.038673 |
| 19 8 0 0.078081 4.008269 1.034466 |
| 20 6 0 -4.094644 0.402634 -0.416088 |
| 21 6 0 -4.830523 -0.187360 0.816729 |
| 22 6 0 -5.498419 -1.566389 0.562376 |
| 23 6 0 -6.310878 -1.976554 1.800800 |
| 24 6 0 -6.388442 -1.589924 -0.676504 |
| 25 8 0 -4.460190 -2.529462 0.319469 |
| 26 8 0 1.760599 2.125612 0.493083 |
| 27 6 0 4.746376 -1.410613 1.758970 |
| 28 6 0 4.405942 -2.660034 2.083738 |
| 29 6 0 5.177309 -0.413735 2.807700 |
| 30 8 0 -3.932405 -0.422691 1.906091 |
| 31 6 0 1.392176 -4.158177 -1.540246 |
| 32 8 0 3.934381 0.929759 -1.099529 |
| 33 8 0 5.135128 2.296105 0.243239 |
| 34 6 0 4.650109 2.050373 -0.833296 |
| 35 6 0 4.738502 2.932126 -2.051953 |
| 36 8 0 -5.094918 1.092700 -1.163060 |
| 37 6 0 -4.739521 1.347071 -2.511440 |
| 38 1 0 -2.430166 4.551391 1.054181 |
| 39 1 0 -4.248475 2.987125 0.403294 |
| 40 1 0 -0.893048 -2.664608 -1.214146 |
| 41 1 0 5.008122 -1.455041 -1.736605 |
| 42 1 0 5.566236 -2.699196 -0.597364 |
| 43 1 0 5.687296 -0.294417 0.222723 |
| 44 1 0 3.438604 0.729962 0.886177 |
| 45 1 0 0.951966 3.557748 0.936234 |
| 46 1 0 -3.690492 -0.424867 -1.006643 |
| 47 1 0 -5.598364 0.538981 1.113736 |
| 48 1 0 -6.724346 -2.976206 1.649794 |
| 49 1 0 -5.687984 -1.989111 2.696412 |
| 50 1 0 -7.140809 -1.284763 1.975399 |
| 51 1 0 -6.855414 -2.574240 -0.759158 |
| 52 1 0 -5.814522 -1.415283 -1.586454 |
| 53 1 0 -7.173516 -0.834155 -0.616703 |
| 54 1 0 -3.820699 -2.444984 1.040735 |
| 55 1 0 4.419911 -2.988665 3.118504 |
| 56 1 0 4.104283 -3.395991 1.348510 |
| 57 1 0 6.216005 -0.104306 2.646716 |
| 58 1 0 4.583156 0.504061 2.777959 |
| 59 1 0 5.100132 -0.835958 3.811314 |
| 60 1 0 -3.422111 0.377375 2.081409 |
| 61 1 0 0.418118 -4.596965 -1.760900 |
| 62 1 0 2.017278 -4.227809 -2.434622 |
| 63 1 0 1.881301 -4.767629 -0.775213 |
| 64 1 0 4.896359 2.344508 -2.957431 |
| 65 1 0 3.792397 3.468610 -2.167260 |
| 66 1 0 5.540245 3.657308 -1.924170 |
| 67 1 0 -5.588709 1.852483 -2.971359 |
| 68 1 0 -3.856075 1.992306 -2.585143 |
| 69 1 0 -4.541493 0.412792 -3.052645 |

**C2**


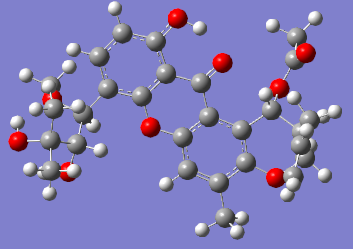


E = -1763.8131656 a.u

Standard orientation:

| Center Atomic Atomic Coordinates (Angstroms) |
| --- |
| Number Number Type X Y Z |
| 1 6 0 -2.404928 3.300133 0.484356 |
| 2 6 0 -3.339330 2.355048 0.072964 |
| 3 6 0 -2.986837 1.057979 -0.299310 |
| 4 6 0 -1.622513 0.750803 -0.262327 |
| 5 6 0 -0.636177 1.665515 0.146173 |
| 6 6 0 -1.052379 2.972624 0.531523 |
| 7 8 0 -1.272396 -0.503591 -0.659876 |
| 8 6 0 0.033879 -0.906269 -0.663776 |
| 9 6 0 1.096354 -0.080133 -0.278572 |
| 10 6 0 0.774732 1.289585 0.152928 |
| 11 6 0 0.233722 -2.222317 -1.086595 |
| 12 6 0 1.505124 -2.751305 -1.143632 |
| 13 6 0 2.596714 -1.926039 -0.765541 |
| 14 6 0 2.419223 -0.617860 -0.323196 |
| 15 8 0 3.807466 -2.542274 -0.848125 |
| 16 6 0 4.986794 -1.740764 -0.681828 |
| 17 6 0 4.849363 -0.702821 0.421202 |
| 18 6 0 3.629394 0.188049 0.098427 |
| 19 8 0 -0.174462 3.902172 0.932585 |
| 20 6 0 -3.991465 0.023067 -0.793482 |
| 21 6 0 -4.242744 -1.192521 0.145460 |
| 22 6 0 -5.322189 -1.033855 1.260466 |
| 23 6 0 -5.286406 -2.254324 2.182584 |
| 24 6 0 -5.181444 0.244676 2.088898 |
| 25 8 0 -6.616111 -1.073197 0.621155 |
| 26 8 0 1.642716 2.109347 0.507363 |
| 27 6 0 4.806452 -1.229238 1.851005 |
| 28 6 0 4.513169 -2.493318 2.164783 |
| 29 6 0 5.162276 -0.215395 2.911526 |
| 30 8 0 -4.591502 -2.311445 -0.665229 |
| 31 6 0 1.754245 -4.162056 -1.596919 |
| 32 8 0 3.944034 1.074066 -1.020158 |
| 33 8 0 5.009717 2.499290 0.374355 |
| 34 6 0 4.578269 2.234432 -0.720302 |
| 35 6 0 4.652825 3.131981 -1.928389 |
| 36 8 0 -5.249243 0.621671 -1.128305 |
| 37 6 0 -5.310908 1.160350 -2.443386 |
| 38 1 0 -2.706538 4.300965 0.770047 |
| 39 1 0 -4.385091 2.635861 0.030332 |
| 40 1 0 -0.631069 -2.812204 -1.369706 |
| 41 1 0 5.191744 -1.239900 -1.633725 |
| 42 1 0 5.786639 -2.452559 -0.479170 |
| 43 1 0 5.725559 -0.049666 0.349939 |
| 44 1 0 3.391118 0.821241 0.944791 |
| 45 1 0 0.726785 3.500963 0.896714 |
| 46 1 0 -3.578412 -0.438216 -1.697469 |
| 47 1 0 -3.286933 -1.439976 0.612244 |
| 48 1 0 -6.128791 -2.210142 2.876228 |
| 49 1 0 -5.354045 -3.183470 1.616343 |
| 50 1 0 -4.361187 -2.272836 2.764691 |
| 51 1 0 -5.905113 0.225146 2.907339 |
| 52 1 0 -5.375901 1.136243 1.493637 |
| 53 1 0 -4.181275 0.337643 2.520813 |
| 54 1 0 -6.655205 -0.314151 0.019302 |
| 55 1 0 4.511941 -2.822976 3.199343 |
| 56 1 0 4.267273 -3.240591 1.420342 |
| 57 1 0 6.196421 0.126146 2.790063 |
| 58 1 0 4.541452 0.683002 2.852042 |
| 59 1 0 5.059162 -0.635823 3.913571 |
| 60 1 0 -5.560870 -2.335749 -0.696849 |
| 61 1 0 0.820650 -4.656413 -1.869157 |
| 62 1 0 2.426198 -4.185552 -2.459049 |
| 63 1 0 2.240505 -4.748534 -0.812282 |
| 64 1 0 4.879269 2.564366 -2.832090 |
| 65 1 0 3.679147 3.608639 -2.073213 |
| 66 1 0 5.401898 3.905164 -1.766837 |
| 67 1 0 -6.303938 1.593619 -2.563700 |
| 68 1 0 -4.558134 1.942023 -2.593003 |
| 69 1 0 -5.166309 0.373260 -3.191893 |

**C3**


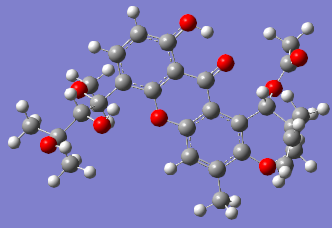


E = -1763.8119647 a.u

Standard orientation:

| Center Atomic Atomic Coordinates (Angstroms) |
| --- |
| Number Number Type X Y Z |
| 1 6 0 -2.219430 3.436735 0.695161 |
| 2 6 0 -3.206406 2.525623 0.341600 |
| 3 6 0 -2.919780 1.217495 -0.061577 |
| 4 6 0 -1.569755 0.854200 -0.102883 |
| 5 6 0 -0.530886 1.738262 0.247761 |
| 6 6 0 -0.877572 3.059313 0.654145 |
| 7 8 0 -1.279849 -0.409933 -0.512814 |
| 8 6 0 0.008444 -0.862158 -0.558573 |
| 9 6 0 1.117001 -0.073194 -0.229332 |
| 10 6 0 0.865657 1.314752 0.185673 |
| 11 6 0 0.144000 -2.191589 -0.965375 |
| 12 6 0 1.390699 -2.771710 -1.057972 |
| 13 6 0 2.526688 -1.985129 -0.732194 |
| 14 6 0 2.415482 -0.663351 -0.309572 |
| 15 8 0 3.707967 -2.651360 -0.841633 |
| 16 6 0 4.924714 -1.899074 -0.717067 |
| 17 6 0 4.858228 -0.838184 0.370863 |
| 18 6 0 3.670383 0.098547 0.059377 |
| 19 8 0 0.053016 3.957070 0.998645 |
| 20 6 0 -4.043753 0.261111 -0.442971 |
| 21 6 0 -4.834601 -0.193332 0.805655 |
| 22 6 0 -5.917401 -1.293469 0.614753 |
| 23 6 0 -7.167006 -0.777049 -0.093686 |
| 24 6 0 -5.358891 -2.539600 -0.086789 |
| 25 8 0 -6.364971 -1.651399 1.932407 |
| 26 8 0 1.778561 2.110723 0.476157 |
| 27 6 0 4.825756 -1.340439 1.809692 |
| 28 6 0 4.501867 -2.590608 2.148117 |
| 29 6 0 5.231089 -0.322561 2.848435 |
| 30 8 0 -3.909353 -0.740579 1.759953 |
| 31 6 0 1.567835 -4.199310 -1.491790 |
| 32 8 0 3.992813 0.943724 -1.087747 |
| 33 8 0 5.150965 2.354725 0.246148 |
| 34 6 0 4.682397 2.083159 -0.831374 |
| 35 6 0 4.765024 2.947996 -2.062451 |
| 36 8 0 -4.967913 0.882875 -1.330632 |
| 37 6 0 -4.464794 1.093526 -2.639437 |
| 38 1 0 -2.465351 4.446844 1.000608 |
| 39 1 0 -4.244761 2.840929 0.355794 |
| 40 1 0 -0.751310 -2.754457 -1.204432 |
| 41 1 0 5.127790 -1.423484 -1.682109 |
| 42 1 0 5.697717 -2.641647 -0.520892 |
| 43 1 0 5.759619 -0.224531 0.268831 |
| 44 1 0 3.483694 0.760576 0.896443 |
| 45 1 0 0.937467 3.526237 0.906636 |
| 46 1 0 -3.607636 -0.621365 -0.920627 |
| 47 1 0 -5.335618 0.680287 1.240776 |
| 48 1 0 -7.938348 -1.549679 -0.047694 |
| 49 1 0 -7.554403 0.112672 0.407900 |
| 50 1 0 -6.970593 -0.527533 -1.134717 |
| 51 1 0 -6.110322 -3.331061 -0.052943 |
| 52 1 0 -4.456786 -2.903432 0.411056 |
| 53 1 0 -5.120770 -2.346481 -1.136833 |
| 54 1 0 -5.575676 -1.775686 2.477973 |
| 55 1 0 4.510720 -2.905080 3.187311 |
| 56 1 0 4.219954 -3.341207 1.419885 |
| 57 1 0 6.268551 -0.004394 2.696518 |
| 58 1 0 4.627725 0.588138 2.795763 |
| 59 1 0 5.145007 -0.729723 3.857506 |
| 60 1 0 -3.376437 -0.029002 2.132823 |
| 61 1 0 0.606409 -4.661747 -1.719985 |
| 62 1 0 2.205899 -4.265599 -2.377143 |
| 63 1 0 2.059621 -4.789686 -0.713633 |
| 64 1 0 4.945526 2.350124 -2.956928 |
| 65 1 0 3.808922 3.461984 -2.196256 |
| 66 1 0 5.549495 3.692264 -1.937408 |
| 67 1 0 -5.274783 1.533980 -3.220616 |
| 68 1 0 -3.609267 1.778723 -2.641639 |
| 69 1 0 -4.161886 0.146502 -3.104975 |

**C4**


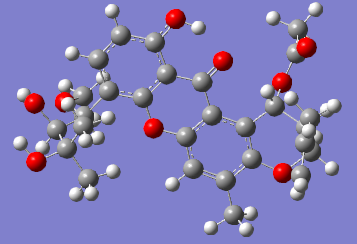


E = -1763.8114431 a.u

Standard orientation:

| Center Atomic Atomic Coordinates (Angstroms) |
| --- |
| Number Number Type X Y Z |
| 1 6 0 -2.649378 3.100056 0.549148 |
| 2 6 0 -3.537951 2.142692 0.080384 |
| 3 6 0 -3.128097 0.874186 -0.347011 |
| 4 6 0 -1.755517 0.613309 -0.298830 |
| 5 6 0 -0.811552 1.550235 0.165978 |
| 6 6 0 -1.284711 2.821738 0.600857 |
| 7 8 0 -1.339027 -0.614294 -0.725715 |
| 8 6 0 -0.014863 -0.954348 -0.730169 |
| 9 6 0 1.004009 -0.098798 -0.296850 |
| 10 6 0 0.613228 1.235114 0.186058 |
| 11 6 0 0.254832 -2.239401 -1.207070 |
| 12 6 0 1.550565 -2.705222 -1.272669 |
| 13 6 0 2.597521 -1.847401 -0.845767 |
| 14 6 0 2.351053 -0.571407 -0.345779 |
| 15 8 0 3.837727 -2.398074 -0.946372 |
| 16 6 0 4.974252 -1.550020 -0.719616 |
| 17 6 0 4.768898 -0.583901 0.436315 |
| 18 6 0 3.516379 0.267593 0.133357 |
| 19 8 0 -0.452168 3.766366 1.055114 |
| 20 6 0 -4.119420 -0.137274 -0.890379 |
| 21 6 0 -5.212483 -0.668643 0.064186 |
| 22 6 0 -4.808137 -1.422009 1.353746 |
| 23 6 0 -3.946040 -2.639795 1.031320 |
| 24 6 0 -4.148130 -0.538836 2.419847 |
| 25 8 0 -6.032295 -1.955978 1.891200 |
| 26 8 0 1.439608 2.073768 0.592480 |
| 27 6 0 4.725900 -1.196261 1.831723 |
| 28 6 0 4.566216 -2.502436 2.058658 |
| 29 6 0 4.927123 -0.220761 2.966581 |
| 30 8 0 -6.104919 0.378663 0.459300 |
| 31 6 0 1.871022 -4.081325 -1.784217 |
| 32 8 0 3.809996 1.216782 -0.937604 |
| 33 8 0 4.789675 2.619924 0.539972 |
| 34 6 0 4.388940 2.388158 -0.573499 |
| 35 6 0 4.445646 3.342488 -1.738239 |
| 36 8 0 -4.890232 0.426367 -1.968950 |
| 37 6 0 -4.158743 0.666277 -3.159303 |
| 38 1 0 -2.992587 4.073291 0.879680 |
| 39 1 0 -4.593733 2.377739 0.047417 |
| 40 1 0 -0.574892 -2.859350 -1.528050 |
| 41 1 0 5.165595 -0.987899 -1.639431 |
| 42 1 0 5.806300 -2.231570 -0.544809 |
| 43 1 0 5.614688 0.111999 0.422067 |
| 44 1 0 3.236492 0.850176 1.002598 |
| 45 1 0 0.465298 3.402565 1.020258 |
| 46 1 0 -3.572195 -1.004481 -1.272072 |
| 47 1 0 -5.777013 -1.395727 -0.533200 |
| 48 1 0 -3.821891 -3.240438 1.934868 |
| 49 1 0 -4.427074 -3.269311 0.277978 |
| 50 1 0 -2.955253 -2.352402 0.673650 |
| 51 1 0 -4.055613 -1.117963 3.341327 |
| 52 1 0 -4.754748 0.344227 2.629240 |
| 53 1 0 -3.153267 -0.200651 2.125379 |
| 54 1 0 -6.659872 -1.222604 1.953543 |
| 55 1 0 4.567874 -2.894240 3.071258 |
| 56 1 0 4.427278 -3.224908 1.263535 |
| 57 1 0 5.897406 0.280829 2.883776 |
| 58 1 0 4.176631 0.574860 2.969192 |
| 59 1 0 4.885925 -0.725984 3.933275 |
| 60 1 0 -6.527195 0.713985 -0.341545 |
| 61 1 0 0.964507 -4.608793 -2.084977 |
| 62 1 0 2.548549 -4.035352 -2.641056 |
| 63 1 0 2.380336 -4.676756 -1.021305 |
| 64 1 0 4.711416 2.827039 -2.662274 |
| 65 1 0 3.455370 3.784684 -1.879893 |
| 66 1 0 5.158459 4.137919 -1.527879 |
| 67 1 0 -4.864817 1.066245 -3.886935 |
| 68 1 0 -3.355116 1.394208 -3.002053 |
| 69 1 0 -3.727136 -0.263602 -3.551532 |

**C5**


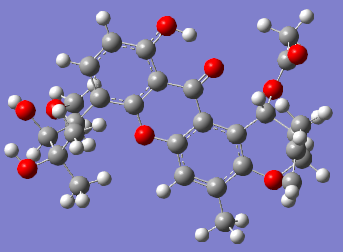


E = -1763.811443 a.u

Standard orientation:

| Center Atomic Atomic Coordinates (Angstroms) |
| --- |
| Number Number Type X Y Z |
| 1 6 0 -2.649375 3.100064 0.549157 |
| 2 6 0 -3.537951 2.142702 0.080391 |
| 3 6 0 -3.128101 0.874197 -0.347008 |
| 4 6 0 -1.755521 0.613319 -0.298834 |
| 5 6 0 -0.811552 1.550241 0.165977 |
| 6 6 0 -1.284708 2.821743 0.600866 |
| 7 8 0 -1.339033 -0.614280 -0.725730 |
| 8 6 0 -0.014871 -0.954341 -0.730178 |
| 9 6 0 1.004004 -0.098795 -0.296857 |
| 10 6 0 0.613227 1.235118 0.186054 |
| 11 6 0 0.254820 -2.239395 -1.207079 |
| 12 6 0 1.550551 -2.705223 -1.272672 |
| 13 6 0 2.597510 -1.847408 -0.845766 |
| 14 6 0 2.351047 -0.571411 -0.345780 |
| 15 8 0 3.837714 -2.398088 -0.946361 |
| 16 6 0 4.974241 -1.550033 -0.719622 |
| 17 6 0 4.768898 -0.583905 0.436304 |
| 18 6 0 3.516376 0.267587 0.133352 |
| 19 8 0 -0.452163 3.766364 1.055129 |
| 20 6 0 -4.119425 -0.137263 -0.890373 |
| 21 6 0 -5.212478 -0.668647 0.064195 |
| 22 6 0 -4.808119 -1.422018 1.353748 |
| 23 6 0 -3.946020 -2.639799 1.031308 |
| 24 6 0 -4.148112 -0.538853 2.419854 |
| 25 8 0 -6.032272 -1.955997 1.891206 |
| 26 8 0 1.439608 2.073769 0.592480 |
| 27 6 0 4.725914 -1.196252 1.831718 |
| 28 6 0 4.566211 -2.502422 2.058668 |
| 29 6 0 4.927178 -0.220745 2.966562 |
| 30 8 0 -6.104930 0.378642 0.459316 |
| 31 6 0 1.871003 -4.081326 -1.784221 |
| 32 8 0 3.809984 1.216778 -0.937608 |
| 33 8 0 4.789690 2.619907 0.539962 |
| 34 6 0 4.388941 2.388148 -0.573505 |
| 35 6 0 4.445641 3.342481 -1.738242 |
| 36 8 0 -4.890249 0.426385 -1.968933 |
| 37 6 0 -4.158769 0.666301 -3.159293 |
| 38 1 0 -2.992583 4.073298 0.879694 |
| 39 1 0 -4.593732 2.377755 0.047427 |
| 40 1 0 -0.574906 -2.859340 -1.528063 |
| 41 1 0 5.165575 -0.987919 -1.639444 |
| 42 1 0 5.806290 -2.231583 -0.544819 |
| 43 1 0 5.614688 0.111995 0.422040 |
| 44 1 0 3.236493 0.850169 1.002596 |
| 45 1 0 0.465302 3.402562 1.020271 |
| 46 1 0 -3.572201 -1.004464 -1.272079 |
| 47 1 0 -5.777000 -1.395736 -0.533194 |
| 48 1 0 -3.821860 -3.240448 1.934852 |
| 49 1 0 -4.427058 -3.269314 0.277967 |
| 50 1 0 -2.955236 -2.352403 0.673632 |
| 51 1 0 -4.055594 -1.117989 3.341329 |
| 52 1 0 -4.754729 0.344207 2.629257 |
| 53 1 0 -3.153248 -0.200666 2.125389 |
| 54 1 0 -6.659853 -1.222628 1.953549 |
| 55 1 0 4.567879 -2.894217 3.071271 |
| 56 1 0 4.427244 -3.224899 1.263554 |
| 57 1 0 5.897476 0.280813 2.883742 |
| 58 1 0 4.176711 0.574899 2.969172 |
| 59 1 0 4.885973 -0.725955 3.933263 |
| 60 1 0 -6.527195 0.713976 -0.341531 |
| 61 1 0 0.964484 -4.608796 -2.084970 |
| 62 1 0 2.548519 -4.035355 -2.641070 |
| 63 1 0 2.380328 -4.676757 -1.021315 |
| 64 1 0 4.711368 2.827032 -2.662290 |
| 65 1 0 3.455375 3.784710 -1.879865 |
| 66 1 0 5.158484 4.137891 -1.527899 |
| 67 1 0 -4.864850 1.066269 -3.886918 |
| 68 1 0 -3.355143 1.394234 -3.002045 |
| 69 1 0 -3.727163 -0.263576 -3.551527 |

**C6**


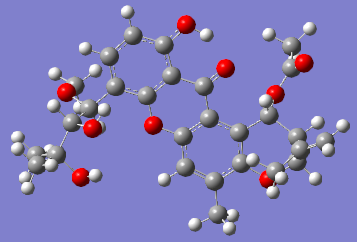


E = -1763.8112419 a.u

Standard orientation:

| Center Atomic Atomic Coordinates (Angstroms) |
| --- |
| Number Number Type X Y Z |
| 1 6 0 -2.227365 3.522993 0.802384 |
| 2 6 0 -3.230352 2.638697 0.423226 |
| 3 6 0 -2.965050 1.334667 -0.006808 |
| 4 6 0 -1.621152 0.947339 -0.044880 |
| 5 6 0 -0.568029 1.804179 0.325767 |
| 6 6 0 -0.892385 3.122379 0.759211 |
| 7 8 0 -1.357048 -0.316998 -0.468286 |
| 8 6 0 -0.077516 -0.789430 -0.533103 |
| 9 6 0 1.046435 -0.024444 -0.199122 |
| 10 6 0 0.820839 1.355143 0.258629 |
| 11 6 0 0.029987 -2.114013 -0.964406 |
| 12 6 0 1.266193 -2.711860 -1.082440 |
| 13 6 0 2.418543 -1.948578 -0.757735 |
| 14 6 0 2.334828 -0.633150 -0.308428 |
| 15 8 0 3.589499 -2.632098 -0.886973 |
| 16 6 0 4.809064 -1.877108 -0.852804 |
| 17 6 0 4.824120 -0.822578 0.251110 |
| 18 6 0 3.606670 0.106819 0.052183 |
| 19 8 0 0.053420 3.994909 1.126568 |
| 20 6 0 -4.093715 0.397350 -0.414237 |
| 21 6 0 -4.842496 -0.211807 0.801433 |
| 22 6 0 -5.501173 -1.589982 0.519901 |
| 23 6 0 -6.328875 -2.019597 1.741474 |
| 24 6 0 -6.373891 -1.600602 -0.731432 |
| 25 8 0 -4.455675 -2.545799 0.278747 |
| 26 8 0 1.746357 2.122415 0.581124 |
| 27 6 0 4.957308 -1.370523 1.667487 |
| 28 6 0 6.016878 -1.020259 2.401111 |
| 29 6 0 3.895519 -2.288008 2.223997 |
| 30 8 0 -3.957137 -0.457635 1.898954 |
| 31 6 0 1.415056 -4.133496 -1.545679 |
| 32 8 0 3.869464 1.012944 -1.063654 |
| 33 8 0 5.105518 2.354560 0.273355 |
| 34 6 0 4.572384 2.140367 -0.786014 |
| 35 6 0 4.579576 3.069866 -1.972301 |
| 36 8 0 -5.087001 1.094937 -1.163476 |
| 37 6 0 -4.715428 1.370675 -2.503301 |
| 38 1 0 -2.456347 4.530468 1.129033 |
| 39 1 0 -4.263445 2.970386 0.438654 |
| 40 1 0 -0.878659 -2.654900 -1.204766 |
| 41 1 0 4.934293 -1.389367 -1.825901 |
| 42 1 0 5.598337 -2.617829 -0.726372 |
| 43 1 0 5.705358 -0.202728 0.075212 |
| 44 1 0 3.466416 0.719352 0.937605 |
| 45 1 0 0.929621 3.547320 1.035969 |
| 46 1 0 -3.680610 -0.421109 -1.011186 |
| 47 1 0 -5.617265 0.507185 1.098272 |
| 48 1 0 -6.735312 -3.019204 1.572229 |
| 49 1 0 -5.718614 -2.040368 2.645615 |
| 50 1 0 -7.164515 -1.333953 1.912953 |
| 51 1 0 -6.837467 -2.584790 -0.832337 |
| 52 1 0 -5.787927 -1.413867 -1.631279 |
| 53 1 0 -7.161305 -0.847155 -0.673555 |
| 54 1 0 -3.827699 -2.469653 1.010945 |
| 55 1 0 6.151468 -1.385890 3.414618 |
| 56 1 0 6.779207 -0.344716 2.026394 |
| 57 1 0 2.913942 -1.803730 2.250377 |
| 58 1 0 3.782411 -3.191877 1.618992 |
| 59 1 0 4.142545 -2.590449 3.243336 |
| 60 1 0 -3.454297 0.342972 2.092448 |
| 61 1 0 0.443351 -4.576233 -1.768777 |
| 62 1 0 2.039430 -4.191773 -2.441344 |
| 63 1 0 1.909324 -4.746446 -0.786768 |
| 64 1 0 4.725215 2.522288 -2.904694 |
| 65 1 0 3.610276 3.572664 -2.032943 |
| 66 1 0 5.358386 3.819976 -1.846808 |
| 67 1 0 -5.560178 1.880767 -2.966195 |
| 68 1 0 -3.832994 2.019315 -2.555945 |
| 69 1 0 -4.507964 0.445224 -3.055998 |

**C7**


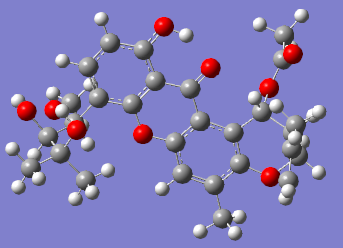


E = -1763.8111215 a.u

Standard orientation:

| Center Atomic Atomic Coordinates (Angstroms) |
| --- |
| Number Number Type X Y Z |
| 1 6 0 -2.680687 3.085285 0.498123 |
| 2 6 0 -3.560677 2.120082 0.028236 |
| 3 6 0 -3.145841 0.846285 -0.375421 |
| 4 6 0 -1.772310 0.595652 -0.319362 |
| 5 6 0 -0.836424 1.538545 0.148652 |
| 6 6 0 -1.317461 2.809525 0.574331 |
| 7 8 0 -1.344169 -0.626438 -0.750841 |
| 8 6 0 -0.018265 -0.955331 -0.755714 |
| 9 6 0 0.991977 -0.097473 -0.308249 |
| 10 6 0 0.588754 1.229720 0.183283 |
| 11 6 0 0.264072 -2.231530 -1.249425 |
| 12 6 0 1.563785 -2.685207 -1.318402 |
| 13 6 0 2.601817 -1.825198 -0.875232 |
| 14 6 0 2.342738 -0.559067 -0.356952 |
| 15 8 0 3.847585 -2.363565 -0.980716 |
| 16 6 0 4.974899 -1.509174 -0.734818 |
| 17 6 0 4.755764 -0.567659 0.438747 |
| 18 6 0 3.498773 0.280119 0.143580 |
| 19 8 0 -0.492812 3.758671 1.035606 |
| 20 6 0 -4.131916 -0.183729 -0.895656 |
| 21 6 0 -5.190731 -0.727390 0.096283 |
| 22 6 0 -4.698923 -1.405832 1.396593 |
| 23 6 0 -5.907952 -1.997559 2.140430 |
| 24 6 0 -3.673682 -2.502315 1.115005 |
| 25 8 0 -4.039822 -0.451238 2.236710 |
| 26 8 0 1.408055 2.068555 0.604396 |
| 27 6 0 4.709707 -1.209171 1.820918 |
| 28 6 0 4.578042 -2.523127 2.019401 |
| 29 6 0 4.874248 -0.253705 2.978547 |
| 30 8 0 -6.076788 0.320685 0.492650 |
| 31 6 0 1.897588 -4.050600 -1.850204 |
| 32 8 0 3.793157 1.250694 -0.907997 |
| 33 8 0 4.754304 2.633081 0.601031 |
| 34 6 0 4.359067 2.419813 -0.518199 |
| 35 6 0 4.409733 3.398695 -1.662709 |
| 36 8 0 -4.947998 0.367571 -1.948608 |
| 37 6 0 -4.258430 0.639048 -3.157469 |
| 38 1 0 -3.030551 4.062114 0.810806 |
| 39 1 0 -4.614227 2.361600 -0.026439 |
| 40 1 0 -0.559550 -2.853830 -1.581376 |
| 41 1 0 5.163581 -0.927304 -1.642896 |
| 42 1 0 5.813384 -2.185494 -0.570564 |
| 43 1 0 5.596191 0.134903 0.444013 |
| 44 1 0 3.208850 0.845297 1.020974 |
| 45 1 0 0.424759 3.393945 1.019059 |
| 46 1 0 -3.581488 -1.039439 -1.297278 |
| 47 1 0 -5.757765 -1.476299 -0.474035 |
| 48 1 0 -5.571253 -2.439267 3.080758 |
| 49 1 0 -6.647575 -1.227072 2.363027 |
| 50 1 0 -6.400414 -2.776955 1.550341 |
| 51 1 0 -3.405035 -2.994839 2.051974 |
| 52 1 0 -2.761080 -2.097936 0.678906 |
| 53 1 0 -4.082157 -3.260096 0.440005 |
| 54 1 0 -4.619678 0.317325 2.325229 |
| 55 1 0 4.577289 -2.935270 3.023893 |
| 56 1 0 4.463647 -3.232408 1.208568 |
| 57 1 0 5.827135 0.282309 2.912052 |
| 58 1 0 4.096865 0.515435 2.996310 |
| 59 1 0 4.845711 -0.782362 3.933060 |
| 60 1 0 -6.419421 0.726716 -0.314350 |
| 61 1 0 0.996196 -4.581186 -2.160981 |
| 62 1 0 2.576173 -3.985717 -2.705019 |
| 63 1 0 2.410512 -4.653345 -1.095513 |
| 64 1 0 4.681325 2.905001 -2.596872 |
| 65 1 0 3.415771 3.835038 -1.796399 |
| 66 1 0 5.115320 4.195620 -1.434369 |
| 67 1 0 -4.996115 1.027067 -3.859806 |
| 68 1 0 -3.469641 1.385649 -3.015416 |
| 69 1 0 -3.814855 -0.275521 -3.571430 |

**C8**


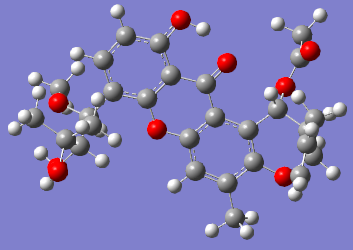


E = -1763.8110206 a.u

Standard orientation:

| Center Atomic Atomic Coordinates (Angstroms) |
| --- |
| Number Number Type X Y Z |
| 1 6 0 -2.543238 3.174750 0.440524 |
| 2 6 0 -3.446129 2.212813 0.000895 |
| 3 6 0 -3.049158 0.936189 -0.398164 |
| 4 6 0 -1.680173 0.660753 -0.346980 |
| 5 6 0 -0.725126 1.595388 0.088747 |
| 6 6 0 -1.181726 2.883682 0.492147 |
| 7 8 0 -1.293068 -0.582146 -0.747449 |
| 8 6 0 0.024131 -0.950378 -0.734046 |
| 9 6 0 1.058765 -0.100349 -0.325624 |
| 10 6 0 0.695258 1.256734 0.112476 |
| 11 6 0 0.266586 -2.256705 -1.163962 |
| 12 6 0 1.552078 -2.752227 -1.205108 |
| 13 6 0 2.615403 -1.902112 -0.802985 |
| 14 6 0 2.395790 -0.603006 -0.352578 |
| 15 8 0 3.842736 -2.485465 -0.871482 |
| 16 6 0 4.998126 -1.655671 -0.675437 |
| 17 6 0 4.813410 -0.632888 0.434664 |
| 18 6 0 3.577265 0.229863 0.097415 |
| 19 8 0 -0.334674 3.830102 0.916809 |
| 20 6 0 -4.033553 -0.105124 -0.903066 |
| 21 6 0 -4.398895 -1.259901 0.063569 |
| 22 6 0 -4.963087 -0.986546 1.492485 |
| 23 6 0 -3.908215 -0.426901 2.445402 |
| 24 6 0 -6.230961 -0.123594 1.505402 |
| 25 8 0 -5.277506 -2.285464 2.025178 |
| 26 8 0 1.535743 2.095520 0.486703 |
| 27 6 0 4.758117 -1.174416 1.858453 |
| 28 6 0 4.494791 -2.449274 2.154701 |
| 29 6 0 5.064964 -0.161571 2.935083 |
| 30 8 0 -5.306325 -2.121462 -0.639267 |
| 31 6 0 1.844931 -4.152522 -1.664412 |
| 32 8 0 3.889010 1.133020 -1.007905 |
| 33 8 0 4.891452 2.573411 0.417620 |
| 34 6 0 4.489053 2.306276 -0.687346 |
| 35 6 0 4.566958 3.213525 -1.887958 |
| 36 8 0 -5.259736 0.483130 -1.342828 |
| 37 6 0 -5.200506 1.051864 -2.644287 |
| 38 1 0 -2.875006 4.160081 0.745801 |
| 39 1 0 -4.501344 2.458155 -0.039152 |
| 40 1 0 -0.576059 -2.868878 -1.465569 |
| 41 1 0 5.207444 -1.140477 -1.618654 |
| 42 1 0 5.811982 -2.348931 -0.464689 |
| 43 1 0 5.673649 0.043153 0.385875 |
| 44 1 0 3.308251 0.849003 0.944993 |
| 45 1 0 0.578016 3.455084 0.885591 |
| 46 1 0 -3.576549 -0.615299 -1.761372 |
| 47 1 0 -3.494851 -1.855527 0.206391 |
| 48 1 0 -4.290230 -0.498547 3.466441 |
| 49 1 0 -2.988121 -1.014747 2.397125 |
| 50 1 0 -3.671212 0.616719 2.240962 |
| 51 1 0 -6.614177 -0.074565 2.527092 |
| 52 1 0 -7.016790 -0.555894 0.880258 |
| 53 1 0 -6.043892 0.891611 1.151248 |
| 54 1 0 -5.726106 -2.778599 1.322861 |
| 55 1 0 4.483269 -2.789053 3.185873 |
| 56 1 0 4.283290 -3.195845 1.399093 |
| 57 1 0 6.090093 0.212512 2.835067 |
| 58 1 0 4.417873 0.718007 2.874220 |
| 59 1 0 4.957492 -0.595614 3.930810 |
| 60 1 0 -5.960727 -1.549630 -1.065494 |
| 61 1 0 0.929119 -4.669104 -1.955375 |
| 62 1 0 2.531332 -4.152321 -2.515370 |
| 63 1 0 2.333123 -4.732070 -0.875884 |
| 64 1 0 4.831212 2.658306 -2.789137 |
| 65 1 0 3.584133 3.663718 -2.053987 |
| 66 1 0 5.290059 4.006145 -1.703882 |
| 67 1 0 -6.194948 1.440266 -2.863620 |
| 68 1 0 -4.475744 1.871303 -2.695163 |
| 69 1 0 -4.934781 0.293959 -3.391449 |

1. Lowest energy conformers (14*R*,15*R*,20*S*,25*R*)-**1** for ORD calcation.

**C1**


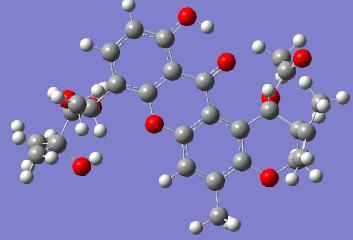


E = -1763.8132175 a.u

Standard orientation:

| Center Atomic Atomic Coordinates (Angstroms) |
| --- |
| Number Number Type X Y Z |
| 1 6 0 -2.224532 3.559154 0.578635 |
| 2 6 0 -3.224289 2.595696 0.645131 |
| 3 6 0 -2.958753 1.225047 0.565983 |
| 4 6 0 -1.618189 0.852607 0.417875 |
| 5 6 0 -0.568697 1.787403 0.344605 |
| 6 6 0 -0.893305 3.172535 0.428291 |
| 7 8 0 -1.354181 -0.478156 0.345844 |
| 8 6 0 -0.078624 -0.940136 0.188777 |
| 9 6 0 1.039972 -0.103516 0.094001 |
| 10 6 0 0.814533 1.347082 0.178467 |
| 11 6 0 0.029434 -2.331404 0.124511 |
| 12 6 0 1.259838 -2.928594 -0.044239 |
| 13 6 0 2.404883 -2.095903 -0.153138 |
| 14 6 0 2.323721 -0.708563 -0.072762 |
| 15 8 0 3.570829 -2.779671 -0.312072 |
| 16 6 0 4.747279 -2.033099 -0.655120 |
| 17 6 0 4.862461 -0.723405 0.109755 |
| 18 6 0 3.592601 0.112183 -0.166705 |
| 19 8 0 0.049398 4.120711 0.366210 |
| 20 6 0 -4.083861 0.201307 0.637346 |
| 21 6 0 -4.861481 0.065159 -0.700025 |
| 22 6 0 -5.513151 -1.327067 -0.920371 |
| 23 6 0 -6.357598 -1.793740 0.261361 |
| 24 6 0 -6.367811 -1.295054 -2.197295 |
| 25 8 0 -4.462130 -2.296629 -1.061263 |
| 26 8 0 1.736213 2.182152 0.110647 |
| 27 6 0 5.162663 -0.829274 1.600105 |
| 28 6 0 4.962374 -1.936564 2.318883 |
| 29 6 0 5.751948 0.415475 2.218858 |
| 30 8 0 -4.003570 0.240207 -1.832540 |
| 31 6 0 1.409550 -4.421525 -0.123441 |
| 32 8 0 3.637016 0.644251 -1.526783 |
| 33 8 0 4.976369 2.335314 -0.849718 |
| 34 6 0 4.311941 1.806732 -1.706289 |
| 35 6 0 4.104708 2.337076 -3.101390 |
| 36 8 0 -5.057617 0.582470 1.607391 |
| 37 6 0 -4.657877 0.358696 2.949000 |
| 38 1 0 -2.453309 4.616126 0.646558 |
| 39 1 0 -4.254096 2.908144 0.784006 |
| 40 1 0 -0.875098 -2.924504 0.201419 |
| 41 1 0 4.716343 -1.828173 -1.730435 |
| 42 1 0 5.579860 -2.704865 -0.448103 |
| 43 1 0 5.691023 -0.167364 -0.341390 |
| 44 1 0 3.547926 0.962430 0.503608 |
| 45 1 0 0.923526 3.671156 0.265615 |
| 46 1 0 -3.662804 -0.775317 0.894222 |
| 47 1 0 -5.642708 0.836445 -0.698908 |
| 48 1 0 -6.815324 -2.754301 0.013197 |
| 49 1 0 -5.752478 -1.934060 1.156968 |
| 50 1 0 -7.149008 -1.078326 0.491570 |
| 51 1 0 -6.770296 -2.291799 -2.390968 |
| 52 1 0 -5.777641 -0.984121 -3.060603 |
| 53 1 0 -7.207128 -0.600402 -2.093282 |
| 54 1 0 -3.848745 -1.954579 -1.726887 |
| 55 1 0 5.210186 -1.964450 3.375704 |
| 56 1 0 4.552438 -2.845731 1.896268 |
| 57 1 0 6.720918 0.652590 1.765459 |
| 58 1 0 5.123256 1.296123 2.059590 |
| 59 1 0 5.900748 0.292400 3.293217 |
| 60 1 0 -3.495452 1.053945 -1.728086 |
| 61 1 0 0.441035 -4.917207 -0.044021 |
| 62 1 0 1.878308 -4.720463 -1.064805 |
| 63 1 0 2.057075 -4.795589 0.674616 |
| 64 1 0 3.121185 2.812565 -3.154729 |
| 65 1 0 4.866742 3.080273 -3.329356 |
| 66 1 0 4.122520 1.533798 -3.839255 |
| 67 1 0 -5.489360 0.669226 3.581648 |
| 68 1 0 -4.447355 -0.703585 3.128393 |
| 69 1 0 -3.769336 0.944587 3.212186 |

**C2**


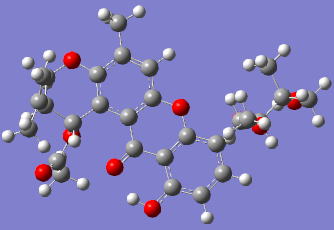


E = -1763.8118209 a.u

Standard orientation:

| Center Atomic Atomic Coordinates (Angstroms) |
| --- |
| Number Number Type X Y Z |
| 1 6 0 2.235743 -3.438425 0.668976 |
| 2 6 0 3.213085 -2.451781 0.703172 |
| 3 6 0 2.919633 -1.091002 0.568838 |
| 4 6 0 1.572740 -0.752074 0.403595 |
| 5 6 0 0.543458 -1.712401 0.362933 |
| 6 6 0 0.897291 -3.086192 0.497988 |
| 7 8 0 1.276627 0.570004 0.284392 |
| 8 6 0 -0.009870 0.995776 0.112423 |
| 9 6 0 -1.109398 0.131351 0.053876 |
| 10 6 0 -0.849537 -1.310114 0.184192 |
| 11 6 0 -0.152373 2.380334 -0.006115 |
| 12 6 0 -1.396612 2.942524 -0.192780 |
| 13 6 0 -2.522637 2.080525 -0.261110 |
| 14 6 0 -2.407857 0.699691 -0.125622 |
| 15 8 0 -3.704329 2.730442 -0.440849 |
| 16 6 0 -4.871441 1.943598 -0.723056 |
| 17 6 0 -4.939453 0.670559 0.105873 |
| 18 6 0 -3.659893 -0.150352 -0.169535 |
| 19 8 0 -0.023702 -4.056638 0.467579 |
| 20 6 0 4.031719 -0.049669 0.614552 |
| 21 6 0 4.890288 -0.091876 -0.671517 |
| 22 6 0 5.927469 1.047556 -0.881771 |
| 23 6 0 5.282620 2.438630 -0.796551 |
| 24 6 0 7.125438 0.938241 0.057217 |
| 25 8 0 6.472846 0.858048 -2.197703 |
| 26 8 0 -1.752085 -2.167433 0.143771 |
| 27 6 0 -5.201560 0.846056 1.597041 |
| 28 6 0 -5.063949 2.008813 2.238980 |
| 29 6 0 -5.682873 -0.393513 2.312296 |
| 30 8 0 4.023355 -0.020600 -1.815003 |
| 31 6 0 -1.581116 4.427190 -0.331015 |
| 32 8 0 -3.720966 -0.724792 -1.511186 |
| 33 8 0 -5.015345 -2.417680 -0.755943 |
| 34 6 0 -4.378353 -1.904058 -1.641814 |
| 35 6 0 -4.190007 -2.472270 -3.024569 |
| 36 8 0 4.908245 -0.282627 1.712918 |
| 37 6 0 4.345163 0.015122 2.979826 |
| 38 1 0 2.486635 -4.486848 0.778285 |
| 39 1 0 4.247631 -2.739399 0.860727 |
| 40 1 0 0.736858 2.998695 0.042598 |
| 41 1 0 -4.858596 1.688648 -1.787737 |
| 42 1 0 -5.714223 2.606677 -0.529973 |
| 43 1 0 -5.768487 0.076546 -0.293504 |
| 44 1 0 -3.585038 -0.978271 0.525235 |
| 45 1 0 -0.907414 -3.631084 0.348477 |
| 46 1 0 3.580258 0.942714 0.704930 |
| 47 1 0 5.443070 -1.039524 -0.688455 |
| 48 1 0 6.013222 3.186884 -1.110529 |
| 49 1 0 4.413016 2.512631 -1.453864 |
| 50 1 0 4.967170 2.684129 0.221754 |
| 51 1 0 7.879081 1.666658 -0.251785 |
| 52 1 0 7.574519 -0.055491 -0.004191 |
| 53 1 0 6.848469 1.126782 1.092937 |
| 54 1 0 5.727975 0.707907 -2.796516 |
| 55 1 0 -5.287375 2.087716 3.298612 |
| 56 1 0 -4.729746 2.915343 1.749159 |
| 57 1 0 -6.625216 -0.751216 1.883201 |
| 58 1 0 -4.978095 -1.225268 2.223591 |
| 59 1 0 -5.844410 -0.199256 3.374220 |
| 60 1 0 3.516230 -0.837875 -1.882113 |
| 61 1 0 -0.624377 4.948397 -0.273609 |
| 62 1 0 -2.057516 4.677529 -1.282651 |
| 63 1 0 -2.236158 4.817707 0.452813 |
| 64 1 0 -3.199287 -2.931758 -3.084550 |
| 65 1 0 -4.942876 -3.235225 -3.214493 |
| 66 1 0 -4.237523 -1.691813 -3.785275 |
| 67 1 0 5.121379 -0.174136 3.721076 |
| 68 1 0 4.039023 1.067737 3.040381 |
| 69 1 0 3.478328 -0.618747 3.199513 |

**C3**


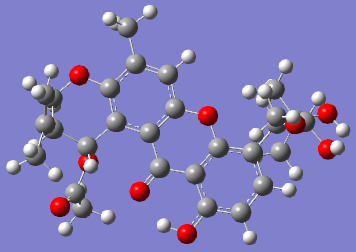


E = -1763.8114181 a.u

Standard orientation:

| Center Atomic Atomic Coordinates (Angstroms) |
| --- |
| Number Number Type X Y Z |
| 1 6 0 2.650781 -2.983823 1.003924 |
| 2 6 0 3.525242 -1.906526 1.033343 |
| 3 6 0 3.109491 -0.588161 0.813693 |
| 4 6 0 1.744780 -0.395290 0.580568 |
| 5 6 0 0.815116 -1.453000 0.542110 |
| 6 6 0 1.294709 -2.777276 0.756708 |
| 7 8 0 1.322499 0.886388 0.376092 |
| 8 6 0 0.004494 1.174659 0.156286 |
| 9 6 0 -1.000086 0.202448 0.100094 |
| 10 6 0 -0.600320 -1.200805 0.290588 |
| 11 6 0 -0.273437 2.532825 -0.015479 |
| 12 6 0 -1.561744 2.959957 -0.256644 |
| 13 6 0 -2.592427 1.986805 -0.324151 |
| 14 6 0 -2.342676 0.629991 -0.134544 |
| 15 8 0 -3.828102 2.506634 -0.558363 |
| 16 6 0 -4.902275 1.595835 -0.839112 |
| 17 6 0 -4.863781 0.350729 0.032631 |
| 18 6 0 -3.500703 -0.343962 -0.179709 |
| 19 8 0 0.475819 -3.835962 0.734717 |
| 20 6 0 4.084297 0.571866 0.884804 |
| 21 6 0 5.208304 0.641097 -0.175291 |
| 22 6 0 4.850265 0.724416 -1.678229 |
| 23 6 0 4.226213 -0.554819 -2.249668 |
| 24 6 0 3.981014 1.944671 -1.970358 |
| 25 8 0 6.093497 0.973992 -2.359760 |
| 26 8 0 -1.411420 -2.144848 0.244572 |
| 27 6 0 -5.188234 0.546481 1.508943 |
| 28 6 0 -5.192481 1.737669 2.112407 |
| 29 6 0 -5.559705 -0.712827 2.254481 |
| 30 8 0 6.116033 -0.455973 -0.028661 |
| 31 6 0 -1.888865 4.413510 -0.452210 |
| 32 8 0 -3.464637 -0.967826 -1.499702 |
| 33 8 0 -4.598644 -2.758034 -0.711842 |
| 34 6 0 -3.992599 -2.213276 -1.600866 |
| 35 6 0 -3.706558 -2.808412 -2.955079 |
| 36 8 0 4.821025 0.550267 2.121939 |
| 37 6 0 4.053600 0.850833 3.275493 |
| 38 1 0 2.998413 -3.996272 1.172178 |
| 39 1 0 4.574226 -2.086830 1.229333 |
| 40 1 0 0.545566 3.241439 0.038884 |
| 41 1 0 -4.835559 1.308697 -1.893490 |
| 42 1 0 -5.812442 2.175875 -0.689387 |
| 43 1 0 -5.615776 -0.338087 -0.367106 |
| 44 1 0 -3.363738 -1.135693 0.547177 |
| 45 1 0 -0.437702 -3.507344 0.553518 |
| 46 1 0 3.526233 1.511586 0.830922 |
| 47 1 0 5.749191 1.569433 0.048525 |
| 48 1 0 4.169823 -0.457623 -3.336175 |
| 49 1 0 4.837128 -1.428402 -2.014569 |
| 50 1 0 3.220216 -0.735504 -1.867658 |
| 51 1 0 3.892579 2.071244 -3.051477 |
| 52 1 0 4.435627 2.852887 -1.565793 |
| 53 1 0 2.977128 1.835335 -1.555917 |
| 54 1 0 6.721837 0.300931 -2.063378 |
| 55 1 0 -5.455634 1.827345 3.162047 |
| 56 1 0 -4.939136 2.657976 1.600309 |
| 57 1 0 -6.448321 -1.179127 1.815107 |
| 58 1 0 -4.771349 -1.469663 2.210897 |
| 59 1 0 -5.769372 -0.502941 3.304935 |
| 60 1 0 6.502119 -0.393252 0.854083 |
| 61 1 0 -0.991717 5.030178 -0.379843 |
| 62 1 0 -2.351182 4.585386 -1.427991 |
| 63 1 0 -2.608891 4.760023 0.294357 |
| 64 1 0 -2.672856 -3.165326 -2.971425 |
| 65 1 0 -4.371907 -3.651125 -3.134264 |
| 66 1 0 -3.810813 -2.064879 -3.746492 |
| 67 1 0 4.737865 0.825619 4.123468 |
| 68 1 0 3.608318 1.851462 3.203307 |
| 69 1 0 3.257095 0.115886 3.435571 |

**C4**


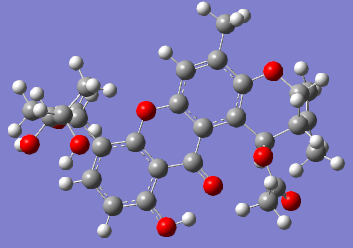


E = -1763.8112703 a.u

Standard orientation:

| Center Atomic Atomic Coordinates (Angstroms) |
| --- |
| Number Number Type X Y Z |
| 1 6 0 -2.674429 -2.963948 -1.035732 |
| 2 6 0 -3.544080 -1.882007 -1.065299 |
| 3 6 0 -3.127852 -0.566806 -0.834008 |
| 4 6 0 -1.762356 -0.380450 -0.603314 |
| 5 6 0 -0.837720 -1.441781 -0.561086 |
| 6 6 0 -1.320785 -2.764420 -0.775117 |
| 7 8 0 -1.331758 0.900687 -0.415121 |
| 8 6 0 -0.013836 1.183716 -0.194702 |
| 9 6 0 0.984213 0.206388 -0.121233 |
| 10 6 0 0.576744 -1.196063 -0.302021 |
| 11 6 0 0.273086 2.542636 -0.042688 |
| 12 6 0 1.562948 2.964780 0.198451 |
| 13 6 0 2.586150 1.985856 0.287179 |
| 14 6 0 2.328088 0.628336 0.115673 |
| 15 8 0 3.824523 2.500483 0.522719 |
| 16 6 0 4.887409 1.585777 0.830850 |
| 17 6 0 4.849181 0.327383 -0.021736 |
| 18 6 0 3.478292 -0.353343 0.185237 |
| 19 8 0 -0.506376 -3.827331 -0.746327 |
| 20 6 0 -4.105423 0.593685 -0.878464 |
| 21 6 0 -5.184049 0.659747 0.232662 |
| 22 6 0 -4.717472 0.728205 1.705472 |
| 23 6 0 -3.730724 1.870778 1.937352 |
| 24 6 0 -5.945752 0.907401 2.613451 |
| 25 8 0 -4.027393 -0.474451 2.066474 |
| 26 8 0 1.383342 -2.143779 -0.244239 |
| 27 6 0 5.192738 0.497878 -1.496822 |
| 28 6 0 5.216112 1.679896 -2.117729 |
| 29 6 0 5.559734 -0.775929 -2.219580 |
| 30 8 0 -6.059391 -0.464594 0.132159 |
| 31 6 0 1.899191 4.419001 0.373491 |
| 32 8 0 3.423066 -0.959087 1.512986 |
| 33 8 0 4.552496 -2.768733 0.763923 |
| 34 6 0 3.938102 -2.207539 1.637040 |
| 35 6 0 3.626576 -2.782719 2.994236 |
| 36 8 0 -4.900437 0.553309 -2.079717 |
| 37 6 0 -4.187405 0.825693 -3.274860 |
| 38 1 0 -3.025225 -3.973666 -1.213951 |
| 39 1 0 -4.589848 -2.063220 -1.277059 |
| 40 1 0 -0.540791 3.255910 -0.111513 |
| 41 1 0 4.805682 1.315351 1.888649 |
| 42 1 0 5.804247 2.155920 0.683351 |
| 43 1 0 5.590865 -0.361176 0.397318 |
| 44 1 0 3.342310 -1.153761 -0.532155 |
| 45 1 0 0.406097 -3.502044 -0.554577 |
| 46 1 0 -3.549355 1.535713 -0.863565 |
| 47 1 0 -5.754100 1.575490 0.023033 |
| 48 1 0 -3.477104 1.919676 2.998537 |
| 49 1 0 -2.805812 1.724571 1.381042 |
| 50 1 0 -4.168076 2.831309 1.650091 |
| 51 1 0 -5.622811 0.914585 3.656732 |
| 52 1 0 -6.659097 0.093796 2.475036 |
| 53 1 0 -6.464956 1.848709 2.407138 |
| 54 1 0 -4.583328 -1.223990 1.813908 |
| 55 1 0 5.492383 1.751225 -3.165441 |
| 56 1 0 4.965448 2.609974 -1.622211 |
| 57 1 0 6.436147 -1.246801 -1.760993 |
| 58 1 0 4.761650 -1.522594 -2.177211 |
| 59 1 0 5.786896 -0.583021 -3.269674 |
| 60 1 0 -6.392740 -0.489066 -0.774242 |
| 61 1 0 1.006783 5.040664 0.285519 |
| 62 1 0 2.356206 4.603119 1.349560 |
| 63 1 0 2.626343 4.749023 -0.373669 |
| 64 1 0 2.586416 -3.120510 3.003089 |
| 65 1 0 4.275231 -3.634601 3.190501 |
| 66 1 0 3.734957 -2.031953 3.778314 |
| 67 1 0 -4.911705 0.788342 -4.088479 |
| 68 1 0 -3.734302 1.824839 -3.243125 |
| 69 1 0 -3.403055 0.083254 -3.457010 |

**C5**


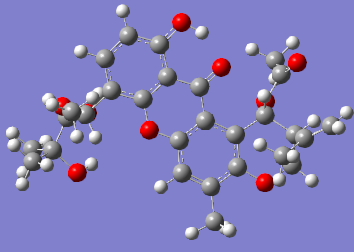


E = -1763.8112033 a.u

Standard orientation:

| Center Atomic Atomic Coordinates (Angstroms) |
| --- |
| Number Number Type X Y Z |
| 1 6 0 -2.247328 3.550939 0.635126 |
| 2 6 0 -3.240847 2.579921 0.680200 |
| 3 6 0 -2.965785 1.212584 0.577997 |
| 4 6 0 -1.622444 0.852100 0.426873 |
| 5 6 0 -0.578751 1.794684 0.377001 |
| 6 6 0 -0.912589 3.175831 0.485646 |
| 7 8 0 -1.349547 -0.475755 0.329199 |
| 8 6 0 -0.070844 -0.926270 0.166691 |
| 9 6 0 1.043001 -0.081012 0.095618 |
| 10 6 0 0.808758 1.366592 0.212885 |
| 11 6 0 0.045913 -2.315401 0.072491 |
| 12 6 0 1.280837 -2.901662 -0.102673 |
| 13 6 0 2.421356 -2.059982 -0.185865 |
| 14 6 0 2.331576 -0.674678 -0.076497 |
| 15 8 0 3.595450 -2.732821 -0.339967 |
| 16 6 0 4.753377 -1.973834 -0.715104 |
| 17 6 0 4.886427 -0.669392 0.066622 |
| 18 6 0 3.597244 0.155694 -0.143925 |
| 19 8 0 0.024124 4.130929 0.447573 |
| 20 6 0 -4.083204 0.179461 0.632567 |
| 21 6 0 -4.857551 0.054177 -0.707651 |
| 22 6 0 -5.495742 -1.341201 -0.947310 |
| 23 6 0 -6.333789 -1.833630 0.228519 |
| 24 6 0 -6.352378 -1.299258 -2.222540 |
| 25 8 0 -4.435132 -2.298041 -1.103425 |
| 26 8 0 1.726062 2.207108 0.175048 |
| 27 6 0 5.284764 -0.829439 1.529344 |
| 28 6 0 6.433154 -0.293829 1.951097 |
| 29 6 0 4.380858 -1.577660 2.479253 |
| 30 8 0 -4.000131 0.252598 -1.836646 |
| 31 6 0 1.440428 -4.391638 -0.212688 |
| 32 8 0 3.614030 0.742210 -1.482057 |
| 33 8 0 4.971407 2.407487 -0.775106 |
| 34 6 0 4.282881 1.913719 -1.632136 |
| 35 6 0 4.033706 2.498532 -2.998612 |
| 36 8 0 -5.061975 0.539914 1.605485 |
| 37 6 0 -4.663406 0.302200 2.945018 |
| 38 1 0 -2.483462 4.604964 0.721143 |
| 39 1 0 -4.273129 2.883044 0.821219 |
| 40 1 0 -0.855608 -2.915125 0.131628 |
| 41 1 0 4.683284 -1.752835 -1.785941 |
| 42 1 0 5.597634 -2.642187 -0.547784 |
| 43 1 0 5.686702 -0.101554 -0.411797 |
| 44 1 0 3.566773 0.975613 0.567084 |
| 45 1 0 0.902519 3.689386 0.348941 |
| 46 1 0 -3.654766 -0.797025 0.877686 |
| 47 1 0 -5.646060 0.817895 -0.697226 |
| 48 1 0 -6.782920 -2.794533 -0.033528 |
| 49 1 0 -5.725675 -1.982008 1.120821 |
| 50 1 0 -7.131374 -1.129269 0.471134 |
| 51 1 0 -6.744505 -2.297230 -2.430597 |
| 52 1 0 -5.766835 -0.969142 -3.081882 |
| 53 1 0 -7.198919 -0.615277 -2.106956 |
| 54 1 0 -3.825818 -1.939849 -1.764307 |
| 55 1 0 6.755531 -0.383462 2.984070 |
| 56 1 0 7.085374 0.266322 1.288716 |
| 57 1 0 3.394432 -1.108531 2.554567 |
| 58 1 0 4.213440 -2.608039 2.153830 |
| 59 1 0 4.811652 -1.604001 3.481918 |
| 60 1 0 -3.501165 1.070563 -1.721622 |
| 61 1 0 0.474768 -4.895063 -0.148703 |
| 62 1 0 1.915526 -4.667201 -1.157991 |
| 63 1 0 2.087079 -4.778758 0.579818 |
| 64 1 0 3.047293 2.970858 -3.005085 |
| 65 1 0 4.786349 3.253725 -3.218215 |
| 66 1 0 4.033712 1.725333 -3.768231 |
| 67 1 0 -5.499257 0.596691 3.579566 |
| 68 1 0 -4.443580 -0.760404 3.110934 |
| 69 1 0 -3.781027 0.892780 3.218264 |

**C6**


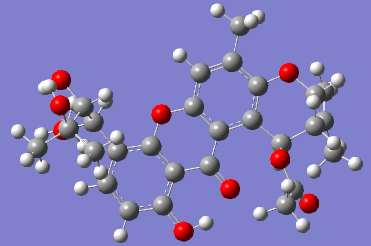


E = -1763.8109974 a.u

Standard orientation:

| Center Atomic Atomic Coordinates (Angstroms) |
| --- |
| Number Number Type X Y Z |
| 1 6 0 -2.559908 -3.005001 -1.072020 |
| 2 6 0 -3.444148 -1.931932 -1.090259 |
| 3 6 0 -3.035036 -0.616855 -0.867396 |
| 4 6 0 -1.671763 -0.414806 -0.636700 |
| 5 6 0 -0.735990 -1.463094 -0.604301 |
| 6 6 0 -1.205544 -2.790328 -0.825923 |
| 7 8 0 -1.271156 0.871169 -0.433693 |
| 8 6 0 0.042777 1.173524 -0.203492 |
| 9 6 0 1.056801 0.210462 -0.141833 |
| 10 6 0 0.676270 -1.196377 -0.345194 |
| 11 6 0 0.303746 2.533868 -0.025859 |
| 12 6 0 1.585477 2.973670 0.226792 |
| 13 6 0 2.625570 2.010734 0.302696 |
| 14 6 0 2.392267 0.651686 0.108592 |
| 15 8 0 3.853111 2.542594 0.550784 |
| 16 6 0 4.931698 1.642634 0.849031 |
| 17 6 0 4.918526 0.395793 -0.020946 |
| 18 6 0 3.558747 -0.311205 0.170199 |
| 19 8 0 -0.377126 -3.842448 -0.810135 |
| 20 6 0 -4.002778 0.554163 -0.896670 |
| 21 6 0 -4.404430 1.169594 0.468337 |
| 22 6 0 -4.999728 0.297965 1.618273 |
| 23 6 0 -6.257026 -0.484350 1.218808 |
| 24 6 0 -3.962021 -0.621239 2.259731 |
| 25 8 0 -5.343063 1.233482 2.656053 |
| 26 8 0 1.496024 -2.132492 -0.303841 |
| 27 6 0 5.264906 0.591822 -1.492285 |
| 28 6 0 5.268417 1.782106 -2.097500 |
| 29 6 0 5.658524 -0.665511 -2.229783 |
| 30 8 0 -5.303460 2.252798 0.190172 |
| 31 6 0 1.895640 4.430035 0.428029 |
| 32 8 0 3.507080 -0.935417 1.489814 |
| 33 8 0 4.672260 -2.714649 0.722735 |
| 34 6 0 4.047227 -2.174695 1.601512 |
| 35 6 0 3.747703 -2.769868 2.952876 |
| 36 8 0 -5.211347 0.233545 -1.588712 |
| 37 6 0 -5.106519 0.298054 -3.005144 |
| 38 1 0 -2.901007 -4.018296 -1.248229 |
| 39 1 0 -4.494176 -2.116070 -1.286686 |
| 40 1 0 -0.523394 3.232658 -0.083718 |
| 41 1 0 4.851964 1.356712 1.902833 |
| 42 1 0 5.838346 2.231179 0.711836 |
| 43 1 0 5.670326 -0.285356 0.392119 |
| 44 1 0 3.440294 -1.104137 -0.558439 |
| 45 1 0 0.533141 -3.507399 -0.626022 |
| 46 1 0 -3.518178 1.383774 -1.428437 |
| 47 1 0 -3.510563 1.641777 0.881239 |
| 48 1 0 -6.668515 -0.967399 2.107867 |
| 49 1 0 -7.029506 0.175840 0.815806 |
| 50 1 0 -6.047783 -1.249740 0.469636 |
| 51 1 0 -4.370380 -1.003457 3.198274 |
| 52 1 0 -3.048681 -0.070893 2.499292 |
| 53 1 0 -3.707053 -1.470449 1.626649 |
| 54 1 0 -5.780007 1.984031 2.227694 |
| 55 1 0 5.547185 1.872556 -3.143027 |
| 56 1 0 4.998981 2.700907 -1.590928 |
| 57 1 0 6.541030 -1.126574 -1.772929 |
| 58 1 0 4.873887 -1.426918 -2.201434 |
| 59 1 0 5.887422 -0.454691 -3.276026 |
| 60 1 0 -5.939489 1.926960 -0.462789 |
| 61 1 0 0.992705 5.037232 0.349273 |
| 62 1 0 2.347928 4.604573 1.407996 |
| 63 1 0 2.618296 4.785308 -0.311893 |
| 64 1 0 2.717900 -3.138258 2.955212 |
| 65 1 0 4.419652 -3.604674 3.144044 |
| 66 1 0 3.831900 -2.023305 3.743803 |
| 67 1 0 -6.090655 0.054598 -3.405204 |
| 68 1 0 -4.821985 1.305991 -3.331209 |
| 69 1 0 -4.374604 -0.420235 -3.389450 |

**C7**


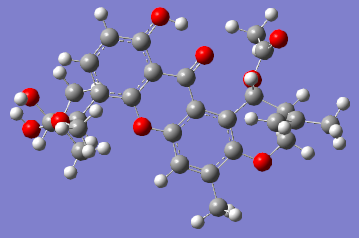


E = -1763.8106037 a.u

Standard orientation:

| Center Atomic Atomic Coordinates (Angstroms) |
| --- |
| Number Number Type X Y Z |
| 1 6 0 -2.719921 2.937856 1.073591 |
| 2 6 0 -3.578267 1.847389 1.067466 |
| 3 6 0 -3.140387 0.540818 0.821357 |
| 4 6 0 -1.770719 0.374089 0.597263 |
| 5 6 0 -0.857067 1.446236 0.593488 |
| 6 6 0 -1.358130 2.757286 0.837195 |
| 7 8 0 -1.326625 -0.896504 0.368601 |
| 8 6 0 -0.003446 -1.158651 0.147903 |
| 9 6 0 0.984768 -0.168651 0.113774 |
| 10 6 0 0.564343 1.222186 0.347123 |
| 11 6 0 0.298025 -2.509158 -0.045841 |
| 12 6 0 1.593394 -2.909784 -0.294861 |
| 13 6 0 2.605891 -1.917385 -0.349879 |
| 14 6 0 2.334344 -0.568339 -0.131353 |
| 15 8 0 3.850886 -2.407771 -0.601924 |
| 16 6 0 4.868457 -1.461641 -0.946917 |
| 17 6 0 4.855895 -0.257152 -0.006711 |
| 18 6 0 3.482230 0.421558 -0.136029 |
| 19 8 0 -0.554745 3.827763 0.851631 |
| 20 6 0 -4.097829 -0.635296 0.857598 |
| 21 6 0 -5.213612 -0.696560 -0.211644 |
| 22 6 0 -4.843904 -0.740191 -1.713427 |
| 23 6 0 -4.235866 0.561112 -2.251308 |
| 24 6 0 -3.953505 -1.939897 -2.026249 |
| 25 8 0 -6.078175 -0.993598 -2.409724 |
| 26 8 0 1.363126 2.176859 0.342125 |
| 27 6 0 5.279956 -0.606331 1.413091 |
| 28 6 0 4.437138 -0.704774 2.442239 |
| 29 6 0 6.763915 -0.813732 1.593617 |
| 30 8 0 -6.138611 0.383104 -0.046264 |
| 31 6 0 1.948656 -4.353703 -0.510150 |
| 32 8 0 3.412631 1.124012 -1.414012 |
| 33 8 0 4.542952 2.878641 -0.543095 |
| 34 6 0 3.918648 2.383342 -1.447399 |
| 35 6 0 3.582951 3.059566 -2.751331 |
| 36 8 0 -4.843178 -0.654535 2.089725 |
| 37 6 0 -4.078739 -0.968525 3.241668 |
| 38 1 0 -3.084438 3.940541 1.263230 |
| 39 1 0 -4.631823 2.007305 1.256498 |
| 40 1 0 -0.508643 -3.232565 -0.002659 |
| 41 1 0 4.699543 -1.128555 -1.976645 |
| 42 1 0 5.801374 -2.022396 -0.910145 |
| 43 1 0 5.590287 0.456592 -0.393791 |
| 44 1 0 3.363094 1.163911 0.646344 |
| 45 1 0 0.366493 3.516722 0.678305 |
| 46 1 0 -3.525137 -1.564929 0.785381 |
| 47 1 0 -5.741931 -1.637773 -0.012981 |
| 48 1 0 -4.167584 0.488383 -3.339062 |
| 49 1 0 -4.863366 1.419355 -2.003641 |
| 50 1 0 -3.236706 0.750013 -1.855616 |
| 51 1 0 -3.853733 -2.039782 -3.109156 |
| 52 1 0 -4.397595 -2.864094 -1.647019 |
| 53 1 0 -2.955105 -1.825138 -1.600192 |
| 54 1 0 -6.718890 -0.336659 -2.103762 |
| 55 1 0 4.797351 -0.944232 3.437928 |
| 56 1 0 3.368469 -0.550668 2.349396 |
| 57 1 0 7.151550 -1.604374 0.941785 |
| 58 1 0 7.318264 0.098344 1.344888 |
| 59 1 0 7.007402 -1.086294 2.621940 |
| 60 1 0 -6.530102 0.294000 0.831837 |
| 61 1 0 1.065346 -4.990118 -0.438187 |
| 62 1 0 2.406756 -4.504070 -1.491511 |
| 63 1 0 2.682556 -4.691885 0.226587 |
| 64 1 0 2.543521 3.397705 -2.714729 |
| 65 1 0 4.228930 3.924042 -2.894140 |
| 66 1 0 3.675974 2.370871 -3.592283 |
| 67 1 0 -4.769574 -0.976378 4.084649 |
| 68 1 0 -3.614304 -1.958715 3.149072 |
| 69 1 0 -3.297363 -0.222960 3.425540 |

**C8**


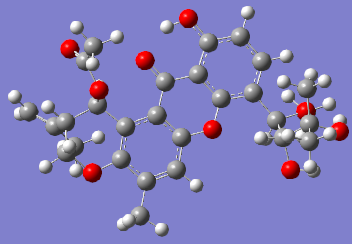


E = -1763.8110286 a.u

Standard orientation:

| Center Atomic Atomic Coordinates (Angstroms) |
| --- |
| Number Number Type X Y Z |
| 1 6 0 2.435202 3.182743 -0.950014 |
| 2 6 0 3.349175 2.134463 -0.979106 |
| 3 6 0 2.979218 0.804762 -0.777255 |
| 4 6 0 1.617725 0.565252 -0.564474 |
| 5 6 0 0.651672 1.585635 -0.522615 |
| 6 6 0 1.085701 2.928603 -0.718154 |
| 7 8 0 1.248932 -0.735941 -0.401379 |
| 8 6 0 -0.056691 -1.080554 -0.191538 |
| 9 6 0 -1.098537 -0.148382 -0.119772 |
| 10 6 0 -0.756454 1.273260 -0.292503 |
| 11 6 0 -0.277503 -2.452310 -0.047377 |
| 12 6 0 -1.548767 -2.934782 0.178137 |
| 13 6 0 -2.617892 -2.004489 0.260192 |
| 14 6 0 -2.424061 -0.634179 0.103231 |
| 15 8 0 -3.836218 -2.578941 0.462322 |
| 16 6 0 -4.922767 -1.721048 0.838293 |
| 17 6 0 -4.974241 -0.434890 0.017805 |
| 18 6 0 -3.620283 0.293765 0.170152 |
| 19 8 0 0.228058 3.957600 -0.691435 |
| 20 6 0 3.961693 -0.359328 -0.845460 |
| 21 6 0 4.245758 -1.094133 0.496244 |
| 22 6 0 5.364778 -0.511770 1.414461 |
| 23 6 0 5.254928 0.995220 1.653957 |
| 24 6 0 5.360768 -1.250478 2.754425 |
| 25 8 0 6.634806 -0.828654 0.804991 |
| 26 8 0 -1.604714 2.182876 -0.251838 |
| 27 6 0 -5.420734 -0.608325 -1.429366 |
| 28 6 0 -6.531983 0.005703 -1.843590 |
| 29 6 0 -4.603380 -1.457131 -2.373133 |
| 30 8 0 4.564909 -2.450617 0.198987 |
| 31 6 0 -1.818704 -4.404009 0.341418 |
| 32 8 0 -3.558667 0.925868 1.485874 |
| 33 8 0 -4.793718 2.669522 0.744470 |
| 34 6 0 -4.127087 2.152706 1.605187 |
| 35 6 0 -3.798682 2.765342 2.942349 |
| 36 8 0 5.206973 0.031145 -1.435989 |
| 37 6 0 5.219527 -0.006736 -2.858118 |
| 38 1 0 2.750214 4.207268 -1.109262 |
| 39 1 0 4.392426 2.353785 -1.173738 |
| 40 1 0 0.572736 -3.122465 -0.110225 |
| 41 1 0 -4.810228 -1.473769 1.899719 |
| 42 1 0 -5.819871 -2.326438 0.711073 |
| 43 1 0 -5.715530 0.208363 0.495966 |
| 44 1 0 -3.546045 1.084099 -0.570577 |
| 45 1 0 -0.675384 3.593560 -0.531387 |
| 46 1 0 3.513683 -1.139429 -1.471106 |
| 47 1 0 3.307189 -1.116969 1.054207 |
| 48 1 0 6.005959 1.298857 2.387203 |
| 49 1 0 5.430092 1.564498 0.741714 |
| 50 1 0 4.270023 1.271139 2.040543 |
| 51 1 0 6.229439 -0.944140 3.341210 |
| 52 1 0 5.402998 -2.330860 2.614218 |
| 53 1 0 4.458537 -1.012873 3.324400 |
| 54 1 0 6.652238 -0.383285 -0.055927 |
| 55 1 0 -6.885727 -0.089545 -2.865753 |
| 56 1 0 -7.120322 0.637619 -1.185982 |
| 57 1 0 -3.581515 -1.078208 -2.477307 |
| 58 1 0 -4.519429 -2.489262 -2.021757 |
| 59 1 0 -5.053870 -1.472439 -3.367305 |
| 60 1 0 5.532631 -2.500482 0.150095 |
| 61 1 0 -0.896267 -4.981697 0.267750 |
| 62 1 0 -2.284809 -4.613407 1.307981 |
| 63 1 0 -2.516031 -4.764624 -0.419977 |
| 64 1 0 -2.783478 3.170384 2.903279 |
| 65 1 0 -4.492091 3.577172 3.154979 |
| 66 1 0 -3.826642 2.021030 3.739346 |
| 67 1 0 6.206760 0.328069 -3.176694 |
| 68 1 0 5.048730 -1.026478 -3.220832 |
| 69 1 0 4.460428 0.658197 -3.284150 |

**C9**


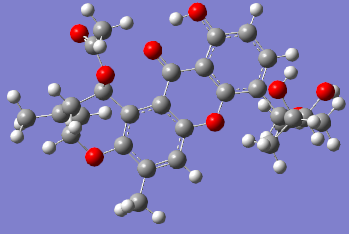


E = -1763.8104693 a.u

Standard orientation:

| Center Atomic Atomic Coordinates (Angstroms) |
| --- |
| Number Number Type X Y Z |
| 1 6 0 2.743603 2.917779 -1.106554 |
| 2 6 0 3.597354 1.822940 -1.100424 |
| 3 6 0 3.159362 0.519764 -0.842267 |
| 4 6 0 1.789099 0.359220 -0.620570 |
| 5 6 0 0.880286 1.434837 -0.612630 |
| 6 6 0 1.384386 2.744313 -0.856010 |
| 7 8 0 1.337069 -0.911167 -0.408646 |
| 8 6 0 0.014151 -1.168417 -0.187156 |
| 9 6 0 -0.967657 -0.173270 -0.135141 |
| 10 6 0 -0.539921 1.217187 -0.357911 |
| 11 6 0 -0.296044 -2.519969 -0.013682 |
| 12 6 0 -1.592839 -2.915998 0.234857 |
| 13 6 0 -2.598030 -1.917974 0.311012 |
| 14 6 0 -2.318321 -0.567741 0.111806 |
| 15 8 0 -3.845681 -2.403826 0.562460 |
| 16 6 0 -4.852541 -1.456401 0.933166 |
| 17 6 0 -4.839099 -0.235037 0.014769 |
| 18 6 0 -3.458688 0.430300 0.141581 |
| 19 8 0 0.585336 3.819034 -0.863262 |
| 20 6 0 4.120184 -0.655734 -0.850278 |
| 21 6 0 5.185696 -0.712688 0.273820 |
| 22 6 0 4.702369 -0.743861 1.742496 |
| 23 6 0 3.697044 -1.867469 1.986821 |
| 24 6 0 5.918194 -0.921451 2.667480 |
| 25 8 0 4.025606 0.475530 2.071087 |
| 26 8 0 -1.334350 2.175774 -0.339776 |
| 27 6 0 -5.280477 -0.555175 -1.406573 |
| 28 6 0 -4.447862 -0.650291 -2.444288 |
| 29 6 0 -6.768862 -0.737928 -1.576967 |
| 30 8 0 6.077355 0.397343 0.159512 |
| 31 6 0 -1.956904 -4.360874 0.428965 |
| 32 8 0 -3.370928 1.112173 1.429524 |
| 33 8 0 -4.495944 2.889922 0.599543 |
| 34 6 0 -3.864036 2.375402 1.487891 |
| 35 6 0 -3.503921 3.028599 2.797013 |
| 36 8 0 4.928452 -0.653408 -2.043331 |
| 37 6 0 4.224226 -0.941049 -3.240125 |
| 38 1 0 3.111013 3.917460 -1.306543 |
| 39 1 0 4.647659 1.983820 -1.306076 |
| 40 1 0 0.505524 -3.248027 -0.071566 |
| 41 1 0 -4.672290 -1.142829 1.967164 |
| 42 1 0 -5.790332 -2.009030 0.894392 |
| 43 1 0 -5.563419 0.477669 0.422175 |
| 44 1 0 -3.340593 1.183762 -0.630209 |
| 45 1 0 -0.334748 3.511630 -0.678300 |
| 46 1 0 3.550336 -1.589185 -0.820800 |
| 47 1 0 5.745449 -1.640218 0.089896 |
| 48 1 0 3.432322 -1.891851 3.046144 |
| 49 1 0 2.779962 -1.718761 1.418360 |
| 50 1 0 4.123139 -2.839666 1.722776 |
| 51 1 0 5.583895 -0.903585 3.707051 |
| 52 1 0 6.644061 -0.120400 2.521075 |
| 53 1 0 6.426621 -1.873601 2.485446 |
| 54 1 0 4.593258 1.211947 1.806159 |
| 55 1 0 -4.819821 -0.868974 -3.440478 |
| 56 1 0 -3.376321 -0.513484 -2.357711 |
| 57 1 0 -7.162619 -1.531019 -0.931722 |
| 58 1 0 -7.307060 0.179070 -1.311381 |
| 59 1 0 -7.025358 -0.993391 -2.606554 |
| 60 1 0 6.421579 0.397749 -0.743161 |
| 61 1 0 -1.078266 -5.001970 0.341534 |
| 62 1 0 -2.410216 -4.524177 1.410511 |
| 63 1 0 -2.697184 -4.682462 -0.308845 |
| 64 1 0 -2.458669 3.347537 2.755193 |
| 65 1 0 -4.132963 3.902417 2.957542 |
| 66 1 0 -3.601437 2.330313 3.629586 |
| 67 1 0 4.957968 -0.933895 -4.046087 |
| 68 1 0 3.754333 -1.931716 -3.191197 |
| 69 1 0 3.454341 -0.190194 -3.447857 |

**C10**


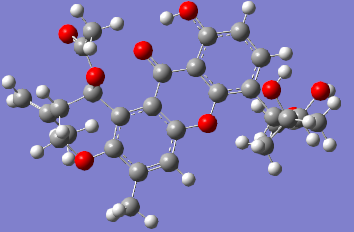


E = -1763.8091874 a.u

Standard orientation:

| Center Atomic Atomic Coordinates (Angstroms) |
| --- |
| Number Number Type X Y Z |
| 1 6 0 2.690426 2.936728 -1.116930 |
| 2 6 0 3.556088 1.851344 -1.109415 |
| 3 6 0 3.132968 0.544339 -0.845576 |
| 4 6 0 1.765378 0.370051 -0.618762 |
| 5 6 0 0.844503 1.435300 -0.613318 |
| 6 6 0 1.333670 2.749394 -0.862681 |
| 7 8 0 1.327997 -0.904117 -0.398363 |
| 8 6 0 0.008730 -1.175226 -0.173289 |
| 9 6 0 -0.985659 -0.192242 -0.129672 |
| 10 6 0 -0.573029 1.202618 -0.357101 |
| 11 6 0 -0.283587 -2.528748 0.014497 |
| 12 6 0 -1.576013 -2.939488 0.261477 |
| 13 6 0 -2.595485 -1.954574 0.319669 |
| 14 6 0 -2.332302 -0.602123 0.115714 |
| 15 8 0 -3.840665 -2.459353 0.547475 |
| 16 6 0 -4.878662 -1.534139 0.901127 |
| 17 6 0 -4.869418 -0.275285 0.038057 |
| 18 6 0 -3.479444 0.387481 0.159713 |
| 19 8 0 0.523131 3.815320 -0.872314 |
| 20 6 0 4.106184 -0.620961 -0.854447 |
| 21 6 0 5.175143 -0.665712 0.266767 |
| 22 6 0 4.696192 -0.702660 1.736693 |
| 23 6 0 3.705046 -1.838222 1.983616 |
| 24 6 0 5.916549 -0.865694 2.658324 |
| 25 8 0 4.005507 0.508393 2.067098 |
| 26 8 0 -1.377545 2.152534 -0.341704 |
| 27 6 0 -5.335340 -0.475533 -1.399337 |
| 28 6 0 -6.434959 0.155197 -1.819457 |
| 29 6 0 -4.550189 -1.370883 -2.327228 |
| 30 8 0 6.053821 0.454414 0.150220 |
| 31 6 0 -1.919202 -4.387430 0.472360 |
| 32 8 0 -3.376584 1.055101 1.454372 |
| 33 8 0 -4.517409 2.841219 0.664742 |
| 34 6 0 -3.873937 2.315521 1.537657 |
| 35 6 0 -3.501211 2.949102 2.853039 |
| 36 8 0 4.911187 -0.611250 -2.049569 |
| 37 6 0 4.207229 -0.907729 -3.244335 |
| 38 1 0 3.046347 3.939680 -1.321239 |
| 39 1 0 4.603996 2.022810 -1.318695 |
| 40 1 0 0.528118 -3.246302 -0.031777 |
| 41 1 0 -4.744864 -1.257545 1.952786 |
| 42 1 0 -5.806861 -2.095919 0.799503 |
| 43 1 0 -5.573723 0.420670 0.498092 |
| 44 1 0 -3.370255 1.149777 -0.605622 |
| 45 1 0 -0.393192 3.499060 -0.683984 |
| 46 1 0 3.546212 -1.560308 -0.822590 |
| 47 1 0 5.744885 -1.586803 0.081225 |
| 48 1 0 3.443882 -1.866293 3.043721 |
| 49 1 0 2.784599 -1.699742 1.418060 |
| 50 1 0 4.141726 -2.805184 1.717666 |
| 51 1 0 5.584962 -0.851858 3.698807 |
| 52 1 0 6.632405 -0.056095 2.509826 |
| 53 1 0 6.435791 -1.811681 2.474766 |
| 54 1 0 4.564443 1.251667 1.802868 |
| 55 1 0 -6.802919 0.039877 -2.834524 |
| 56 1 0 -6.999502 0.820545 -1.174040 |
| 57 1 0 -3.520845 -1.019828 -2.453065 |
| 58 1 0 -4.487471 -2.394395 -1.947229 |
| 59 1 0 -5.012281 -1.403121 -3.315696 |
| 60 1 0 6.396974 0.458029 -0.752820 |
| 61 1 0 -1.028905 -5.014717 0.405624 |
| 62 1 0 -2.382287 -4.543886 1.450375 |
| 63 1 0 -2.644241 -4.734288 -0.269238 |
| 64 1 0 -2.456984 3.270645 2.805923 |
| 65 1 0 -4.130138 3.819119 3.033387 |
| 66 1 0 -3.589314 2.237883 3.675647 |
| 67 1 0 4.939123 -0.894720 -4.051886 |
| 68 1 0 3.747210 -1.902901 -3.192820 |
| 69 1 0 3.429595 -0.164800 -3.451683 |

1. Lowest energy conformers (14*R*,15*S*,20*S*,25*R*)-**2** for ORD calcation.

**C1**


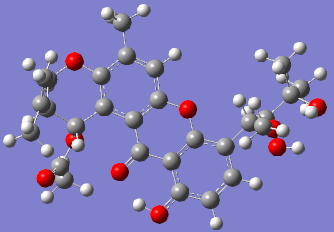


E = -1763.816384 a.u

Standard orientation:

| Center Atomic Atomic Coordinates (Angstroms) |
| --- |
| Number Number Type X Y Z |
| 1 6 0 2.229197 -3.398804 0.630152 |
| 2 6 0 3.200176 -2.406153 0.650933 |
| 3 6 0 2.890010 -1.048755 0.531604 |
| 4 6 0 1.542066 -0.723611 0.371966 |
| 5 6 0 0.518497 -1.690229 0.340820 |
| 6 6 0 0.886352 -3.060229 0.476055 |
| 7 8 0 1.236580 0.599808 0.233119 |
| 8 6 0 -0.056099 1.014792 0.075441 |
| 9 6 0 -1.148473 0.141274 0.031886 |
| 10 6 0 -0.875867 -1.299722 0.168242 |
| 11 6 0 -0.212760 2.397604 -0.046693 |
| 12 6 0 -1.463348 2.949120 -0.224785 |
| 13 6 0 -2.581480 2.077426 -0.280811 |
| 14 6 0 -2.452752 0.698052 -0.139073 |
| 15 8 0 -3.770950 2.716125 -0.454598 |
| 16 6 0 -4.932776 1.917015 -0.723707 |
| 17 6 0 -4.982408 0.648100 0.112473 |
| 18 6 0 -3.697948 -0.162841 -0.169510 |
| 19 8 0 -0.027719 -4.039539 0.458701 |
| 20 6 0 3.967350 0.019057 0.545511 |
| 21 6 0 4.659476 0.170900 -0.830586 |
| 22 6 0 5.787377 1.248487 -0.866345 |
| 23 6 0 6.208480 1.513138 -2.311962 |
| 24 6 0 5.385439 2.560755 -0.185491 |
| 25 8 0 6.950227 0.689136 -0.222355 |
| 26 8 0 -1.775173 -2.161047 0.137792 |
| 27 6 0 -5.233904 0.829458 1.604728 |
| 28 6 0 -5.108336 1.998386 2.237909 |
| 29 6 0 -5.689844 -0.412347 2.332614 |
| 30 8 0 5.178894 -1.067801 -1.284147 |
| 31 6 0 -1.661841 4.432067 -0.364678 |
| 32 8 0 -3.764039 -0.743160 -1.508130 |
| 33 8 0 -5.041649 -2.441952 -0.737918 |
| 34 6 0 -4.410689 -1.929402 -1.628767 |
| 35 6 0 -4.219025 -2.506300 -3.007338 |
| 36 8 0 4.989711 -0.255711 1.516840 |
| 37 6 0 4.559699 -0.151954 2.867880 |
| 38 1 0 2.491866 -4.445712 0.725666 |
| 39 1 0 4.240524 -2.683430 0.766596 |
| 40 1 0 0.670181 3.025694 -0.003772 |
| 41 1 0 -4.925853 1.656086 -1.787019 |
| 42 1 0 -5.780287 2.573330 -0.527941 |
| 43 1 0 -5.809284 0.044297 -0.276619 |
| 44 1 0 -3.610170 -0.987244 0.527838 |
| 45 1 0 -0.914921 -3.622595 0.343465 |
| 46 1 0 3.502118 0.978292 0.794601 |
| 47 1 0 3.875139 0.458917 -1.538449 |
| 48 1 0 7.088705 2.159573 -2.325037 |
| 49 1 0 6.456023 0.584011 -2.826125 |
| 50 1 0 5.407024 2.009124 -2.866045 |
| 51 1 0 6.178778 3.299434 -0.320261 |
| 52 1 0 5.231951 2.433767 0.888623 |
| 53 1 0 4.465933 2.970733 -0.615544 |
| 54 1 0 6.692918 0.468374 0.686741 |
| 55 1 0 -5.324130 2.080970 3.298865 |
| 56 1 0 -4.791031 2.906283 1.739488 |
| 57 1 0 -6.627721 -0.790563 1.911455 |
| 58 1 0 -4.970669 -1.232034 2.247271 |
| 59 1 0 -5.849207 -0.212039 3.393768 |
| 60 1 0 6.100279 -1.106777 -0.983714 |
| 61 1 0 -0.709199 4.961811 -0.314689 |
| 62 1 0 -2.146825 4.676848 -1.313453 |
| 63 1 0 -2.314560 4.818837 0.422917 |
| 64 1 0 -3.224760 -2.958475 -3.064035 |
| 65 1 0 -4.966091 -3.276411 -3.191279 |
| 66 1 0 -4.272642 -1.731832 -3.773759 |
| 67 1 0 5.430394 -0.349488 3.493009 |
| 68 1 0 4.180190 0.854813 3.084667 |
| 69 1 0 3.778852 -0.883190 3.097513 |

**C2**


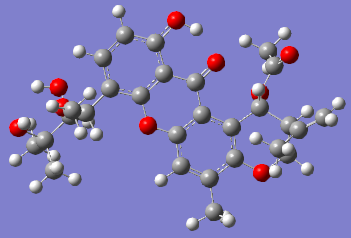


E = -1763.814321 a.u

Standard orientation:

| Center Atomic Atomic Coordinates (Angstroms) |
| --- |
| Number Number Type X Y Z |
| 1 6 0 -2.246675 3.381501 0.710111 |
| 2 6 0 -3.212959 2.384310 0.701016 |
| 3 6 0 -2.895310 1.031088 0.554731 |
| 4 6 0 -1.544728 0.716131 0.398733 |
| 5 6 0 -0.525688 1.687811 0.395936 |
| 6 6 0 -0.900832 3.052621 0.559828 |
| 7 8 0 -1.231115 -0.603334 0.237060 |
| 8 6 0 0.064051 -1.008246 0.076609 |
| 9 6 0 1.151841 -0.128218 0.050704 |
| 10 6 0 0.872102 1.308092 0.224668 |
| 11 6 0 0.228081 -2.388415 -0.066490 |
| 12 6 0 1.482109 -2.930544 -0.249281 |
| 13 6 0 2.595151 -2.051769 -0.290731 |
| 14 6 0 2.459618 -0.674717 -0.130493 |
| 15 8 0 3.791513 -2.681517 -0.458644 |
| 16 6 0 4.925610 -1.872854 -0.801660 |
| 17 6 0 5.013836 -0.594402 0.027627 |
| 18 6 0 3.699415 0.196887 -0.156964 |
| 19 8 0 0.008461 4.036165 0.572518 |
| 20 6 0 -3.967800 -0.041660 0.539406 |
| 21 6 0 -4.652307 -0.166366 -0.843346 |
| 22 6 0 -5.777200 -1.245542 -0.908624 |
| 23 6 0 -6.189685 -1.478982 -2.362179 |
| 24 6 0 -5.376289 -2.572007 -0.255340 |
| 25 8 0 -6.944861 -0.703130 -0.259047 |
| 26 8 0 1.767828 2.172357 0.229163 |
| 27 6 0 5.412218 -0.796081 1.485141 |
| 28 6 0 6.541430 -0.240693 1.932280 |
| 29 6 0 4.528580 -1.606628 2.402475 |
| 30 8 0 -5.172575 1.080391 -1.273346 |
| 31 6 0 1.689449 -4.410284 -0.409824 |
| 32 8 0 3.702629 0.837497 -1.469527 |
| 33 8 0 5.006221 2.512298 -0.688198 |
| 34 6 0 4.333324 2.035775 -1.567080 |
| 35 6 0 4.064030 2.672830 -2.905834 |
| 36 8 0 -4.996094 0.207454 1.511304 |
| 37 6 0 -4.572860 0.073990 2.861825 |
| 38 1 0 -2.515031 4.424754 0.827263 |
| 39 1 0 -4.255456 2.654079 0.814854 |
| 40 1 0 -0.651899 -3.021363 -0.035984 |
| 41 1 0 4.852087 -1.615658 -1.864183 |
| 42 1 0 5.790449 -2.519377 -0.654804 |
| 43 1 0 5.796826 0.016255 -0.425986 |
| 44 1 0 3.639677 0.985516 0.586838 |
| 45 1 0 0.899312 3.626174 0.460686 |
| 46 1 0 -3.499181 -1.003903 0.770152 |
| 47 1 0 -3.863295 -0.437049 -1.552882 |
| 48 1 0 -7.067760 -2.127619 -2.394534 |
| 49 1 0 -6.437477 -0.539386 -2.856758 |
| 50 1 0 -5.383668 -1.959880 -2.922895 |
| 51 1 0 -6.166194 -3.309693 -0.413570 |
| 52 1 0 -5.231792 -2.470189 0.822707 |
| 53 1 0 -4.452269 -2.969225 -0.687714 |
| 54 1 0 -6.692714 -0.500976 0.655793 |
| 55 1 0 6.862452 -0.358553 2.962854 |
| 56 1 0 7.177887 0.364424 1.294584 |
| 57 1 0 3.527684 -1.171395 2.489887 |
| 58 1 0 4.394668 -2.629076 2.038644 |
| 59 1 0 4.956084 -1.656307 3.405686 |
| 60 1 0 -6.096095 1.110219 -0.978422 |
| 61 1 0 0.739669 -4.945993 -0.370403 |
| 62 1 0 2.178555 -4.638258 -1.360642 |
| 63 1 0 2.342587 -4.805129 0.373405 |
| 64 1 0 3.066612 3.121152 -2.887238 |
| 65 1 0 4.797050 3.455089 -3.095081 |
| 66 1 0 4.078487 1.934128 -3.708444 |
| 67 1 0 -5.447914 0.252241 3.486631 |
| 68 1 0 -4.189374 -0.935771 3.056769 |
| 69 1 0 -3.797106 0.803569 3.113067 |

1. Lowest energy conformers (14*S*,15*R*,20*S*,25*R*)-**3** for ORD calcation.

**C1**


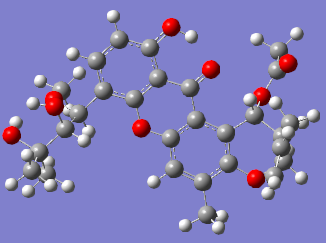


E = -1763.8164571 a.u

Standard orientation:

| Center Atomic Atomic Coordinates (Angstroms) |
| --- |
| Number Number Type X Y Z |
| 1 6 0 -2.213873 3.419623 0.626560 |
| 2 6 0 -3.196028 2.493822 0.299244 |
| 3 6 0 -2.896132 1.185446 -0.088291 |
| 4 6 0 -1.546338 0.831676 -0.116301 |
| 5 6 0 -0.511816 1.728163 0.213353 |
| 6 6 0 -0.869541 3.055339 0.589017 |
| 7 8 0 -1.250349 -0.450746 -0.479372 |
| 8 6 0 0.042543 -0.892068 -0.525367 |
| 9 6 0 1.145229 -0.087272 -0.217451 |
| 10 6 0 0.884460 1.310544 0.166405 |
| 11 6 0 0.189029 -2.227806 -0.907362 |
| 12 6 0 1.440218 -2.799582 -0.994215 |
| 13 6 0 2.569606 -1.997909 -0.686461 |
| 14 6 0 2.447827 -0.668804 -0.289189 |
| 15 8 0 3.756704 -2.656425 -0.786705 |
| 16 6 0 4.967064 -1.891921 -0.677337 |
| 17 6 0 4.892580 -0.813014 0.391957 |
| 18 6 0 3.697765 0.109269 0.063468 |
| 19 8 0 0.055540 3.969604 0.910338 |
| 20 6 0 -3.984600 0.184079 -0.425499 |
| 21 6 0 -4.576192 -0.481470 0.841026 |
| 22 6 0 -5.717215 -1.506909 0.555076 |
| 23 6 0 -5.389754 -2.464107 -0.595538 |
| 24 6 0 -6.033823 -2.298782 1.824012 |
| 25 8 0 -6.914186 -0.759866 0.257248 |
| 26 8 0 1.795300 2.114829 0.440283 |
| 27 6 0 4.863377 -1.291055 1.839072 |
| 28 6 0 4.558584 -2.540426 2.197782 |
| 29 6 0 5.249183 -0.249781 2.861991 |
| 30 8 0 -5.038834 0.490844 1.763063 |
| 31 6 0 1.628172 -4.233182 -1.403762 |
| 32 8 0 4.013419 0.933964 -1.100180 |
| 33 8 0 5.168163 2.375898 0.203292 |
| 34 6 0 4.695506 2.082677 -0.866733 |
| 35 6 0 4.764418 2.927541 -2.112437 |
| 36 8 0 -5.069907 0.787054 -1.147436 |
| 37 6 0 -4.742283 1.191635 -2.470306 |
| 38 1 0 -2.468643 4.430429 0.922352 |
| 39 1 0 -4.237592 2.787874 0.338715 |
| 40 1 0 -0.701121 -2.802943 -1.137234 |
| 41 1 0 5.165160 -1.431324 -1.650674 |
| 42 1 0 5.746753 -2.624261 -0.469266 |
| 43 1 0 5.789301 -0.194294 0.279841 |
| 44 1 0 3.506448 0.786265 0.887296 |
| 45 1 0 0.941962 3.542257 0.834152 |
| 46 1 0 -3.550387 -0.605357 -1.047632 |
| 47 1 0 -3.746476 -1.004418 1.328073 |
| 48 1 0 -6.183787 -3.208444 -0.689081 |
| 49 1 0 -5.312671 -1.942195 -1.552069 |
| 50 1 0 -4.448703 -2.994922 -0.419175 |
| 51 1 0 -6.923943 -2.910460 1.661826 |
| 52 1 0 -6.222023 -1.632857 2.666541 |
| 53 1 0 -5.203316 -2.959292 2.087127 |
| 54 1 0 -6.720043 -0.211456 -0.519153 |
| 55 1 0 4.569530 -2.837028 3.242212 |
| 56 1 0 4.290050 -3.307344 1.481569 |
| 57 1 0 4.627322 0.647521 2.796842 |
| 58 1 0 5.171903 -0.643604 3.877057 |
| 59 1 0 6.279799 0.087077 2.704187 |
| 60 1 0 -5.978838 0.629664 1.568825 |
| 61 1 0 0.670006 -4.707493 -1.621869 |
| 62 1 0 2.264548 -4.309985 -2.289459 |
| 63 1 0 2.126447 -4.806230 -0.616791 |
| 64 1 0 3.805156 3.435810 -2.245518 |
| 65 1 0 5.547046 3.676714 -2.006371 |
| 66 1 0 4.939178 2.315940 -2.998732 |
| 67 1 0 -5.651757 1.599984 -2.910730 |
| 68 1 0 -3.964068 1.960755 -2.473090 |
| 69 1 0 -4.401827 0.337992 -3.070290 |

**C2**


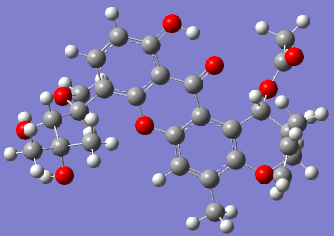


E = -1763.8149438 a.u

Standard orientation:

| Center Atomic Atomic Coordinates (Angstroms) |
| --- |
| Number Number Type X Y Z |
|  |
| 1 6 0 -2.455661 3.294976 0.736966 |
| 2 6 0 -3.402277 2.381160 0.294005 |
| 3 6 0 -3.067044 1.102284 -0.162691 |
| 4 6 0 -1.705998 0.782119 -0.177225 |
| 5 6 0 -0.705318 1.670452 0.260418 |
| 6 6 0 -1.103465 2.955741 0.728285 |
| 7 8 0 -1.365481 -0.452782 -0.636973 |
| 8 6 0 -0.062938 -0.866029 -0.667295 |
| 9 6 0 1.010918 -0.062843 -0.266654 |
| 10 6 0 0.703875 1.289419 0.222815 |
| 11 6 0 0.122268 -2.168899 -1.135452 |
| 12 6 0 1.388629 -2.705374 -1.225524 |
| 13 6 0 2.491457 -1.901144 -0.835152 |
| 14 6 0 2.328944 -0.608169 -0.345202 |
| 15 8 0 3.696532 -2.521815 -0.955888 |
| 16 6 0 4.883428 -1.734920 -0.773277 |
| 17 6 0 4.765263 -0.739468 0.370403 |
| 18 6 0 3.549629 0.172129 0.093281 |
| 19 8 0 -0.211850 3.856734 1.157641 |
| 20 6 0 -4.132485 0.164244 -0.694370 |
| 21 6 0 -5.250198 -0.194940 0.321211 |
| 22 6 0 -4.915516 -1.352042 1.304526 |
| 23 6 0 -3.611368 -1.160851 2.072347 |
| 24 6 0 -6.084267 -1.513934 2.290131 |
| 25 8 0 -4.753041 -2.555073 0.540322 |
| 26 8 0 1.582832 2.090404 0.593777 |
| 27 6 0 4.734258 -1.321462 1.778782 |
| 28 6 0 4.456404 -2.600199 2.043780 |
| 29 6 0 5.086695 -0.348504 2.878078 |
| 30 8 0 -6.404742 -0.642109 -0.391580 |
| 31 6 0 1.620783 -4.103412 -1.724843 |
| 32 8 0 3.861093 1.096501 -0.994684 |
| 33 8 0 4.950851 2.460585 0.441776 |
| 34 6 0 4.508146 2.239667 -0.658075 |
| 35 6 0 4.581412 3.179858 -1.833392 |
| 36 8 0 -4.824469 0.788016 -1.798615 |
| 37 6 0 -4.071371 0.861887 -2.998898 |
| 38 1 0 -2.741330 4.279947 1.086691 |
| 39 1 0 -4.445751 2.677857 0.283465 |
| 40 1 0 -0.750042 -2.745999 -1.421168 |
| 41 1 0 5.080422 -1.199082 -1.707617 |
| 42 1 0 5.680751 -2.459166 -0.608267 |
| 43 1 0 5.645517 -0.090130 0.315274 |
| 44 1 0 3.324617 0.775345 0.964696 |
| 45 1 0 0.686899 3.454434 1.076221 |
| 46 1 0 -3.680804 -0.765430 -1.049566 |
| 47 1 0 -5.509713 0.700301 0.901660 |
| 48 1 0 -3.512292 -1.956892 2.814095 |
| 49 1 0 -2.748667 -1.221029 1.410419 |
| 50 1 0 -3.589972 -0.202691 2.596787 |
| 51 1 0 -5.904202 -2.384357 2.924719 |
| 52 1 0 -7.029093 -1.655554 1.763329 |
| 53 1 0 -6.184204 -0.634233 2.933727 |
| 54 1 0 -5.530283 -2.622411 -0.033301 |
| 55 1 0 4.466252 -2.970550 3.064409 |
| 56 1 0 4.211963 -3.320492 1.272787 |
| 57 1 0 4.443020 0.535820 2.873640 |
| 58 1 0 5.012454 -0.817528 3.860922 |
| 59 1 0 6.109605 0.024164 2.754337 |
| 60 1 0 -6.428408 -0.146707 -1.223604 |
| 61 1 0 0.679768 -4.583290 -1.997540 |
| 62 1 0 2.279926 -4.105671 -2.597149 |
| 63 1 0 2.114014 -4.716414 -0.965297 |
| 64 1 0 3.609411 3.665727 -1.957150 |
| 65 1 0 5.334876 3.943206 -1.647465 |
| 66 1 0 4.801709 2.644004 -2.757805 |
| 67 1 0 -4.718399 1.315668 -3.749335 |
| 68 1 0 -3.178554 1.483938 -2.873785 |
| 69 1 0 -3.768487 -0.136922 -3.336217 |

**C3**


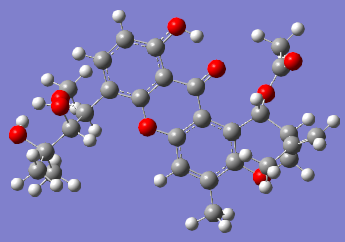


E = -1763.8143269 a.u

Standard orientation:

| Center Atomic Atomic Coordinates (Angstroms) |
| --- |
| Number Number Type X Y Z |
| 1 6 0 -2.232444 3.402795 0.694125 |
| 2 6 0 -3.208580 2.479367 0.343536 |
| 3 6 0 -2.901213 1.176864 -0.058360 |
| 4 6 0 -1.550395 0.827201 -0.076841 |
| 5 6 0 -0.521574 1.722090 0.273865 |
| 6 6 0 -0.886649 3.042495 0.665721 |
| 7 8 0 -1.247239 -0.451238 -0.449906 |
| 8 6 0 0.047640 -0.885383 -0.498535 |
| 9 6 0 1.146251 -0.077617 -0.183569 |
| 10 6 0 0.876884 1.310377 0.231495 |
| 11 6 0 0.200269 -2.217796 -0.890523 |
| 12 6 0 1.454370 -2.781829 -0.985467 |
| 13 6 0 2.580008 -1.975009 -0.677901 |
| 14 6 0 2.452916 -0.649467 -0.268821 |
| 15 8 0 3.772219 -2.626196 -0.780982 |
| 16 6 0 4.966825 -1.832065 -0.769319 |
| 17 6 0 4.946074 -0.745636 0.302759 |
| 18 6 0 3.700790 0.139561 0.073967 |
| 19 8 0 0.032177 3.954653 1.009335 |
| 20 6 0 -3.983810 0.178190 -0.421557 |
| 21 6 0 -4.590894 -0.504058 0.828475 |
| 22 6 0 -5.726787 -1.527121 0.513934 |
| 23 6 0 -5.383242 -2.467391 -0.645851 |
| 24 6 0 -6.058426 -2.337120 1.767404 |
| 25 8 0 -6.920964 -0.777579 0.211402 |
| 26 8 0 1.781425 2.109026 0.536703 |
| 27 6 0 5.092327 -1.248247 1.734494 |
| 28 6 0 6.153497 -0.867461 2.450365 |
| 29 6 0 4.041349 -2.156243 2.325974 |
| 30 8 0 -5.066721 0.455446 1.757042 |
| 31 6 0 1.649686 -4.211232 -1.406243 |
| 32 8 0 3.938985 1.019829 -1.067343 |
| 33 8 0 5.130952 2.436692 0.231462 |
| 34 6 0 4.605445 2.176575 -0.821271 |
| 35 6 0 4.583520 3.072674 -2.032762 |
| 36 8 0 -5.060586 0.788935 -1.149748 |
| 37 6 0 -4.716346 1.213155 -2.462227 |
| 38 1 0 -2.492979 4.408628 1.001601 |
| 39 1 0 -4.251259 2.770225 0.376190 |
| 40 1 0 -0.687447 -2.795612 -1.123123 |
| 41 1 0 5.078054 -1.369756 -1.756495 |
| 42 1 0 5.779204 -2.542927 -0.620013 |
| 43 1 0 5.808332 -0.104328 0.110456 |
| 44 1 0 3.537836 0.772876 0.940649 |
| 45 1 0 0.920543 3.529966 0.942203 |
| 46 1 0 -3.540960 -0.603172 -1.047868 |
| 47 1 0 -3.766921 -1.032521 1.319412 |
| 48 1 0 -6.175606 -3.210461 -0.761241 |
| 49 1 0 -5.293066 -1.931492 -1.593469 |
| 50 1 0 -4.444516 -3.000626 -0.464369 |
| 51 1 0 -6.944903 -2.948369 1.584823 |
| 52 1 0 -6.259365 -1.683262 2.616428 |
| 53 1 0 -5.230071 -2.999556 2.032520 |
| 54 1 0 -6.718178 -0.218760 -0.555288 |
| 55 1 0 6.297492 -1.201369 3.473465 |
| 56 1 0 6.907741 -0.197105 2.050734 |
| 57 1 0 3.934409 -3.081399 1.752757 |
| 58 1 0 4.295074 -2.421685 3.353972 |
| 59 1 0 3.055599 -1.680043 2.340481 |
| 60 1 0 -6.004444 0.595424 1.552840 |
| 61 1 0 0.693747 -4.689824 -1.624757 |
| 62 1 0 2.283469 -4.276986 -2.294667 |
| 63 1 0 2.154825 -4.787360 -0.625916 |
| 64 1 0 3.601276 3.548639 -2.101423 |
| 65 1 0 5.343095 3.845875 -1.931797 |
| 66 1 0 4.738949 2.503671 -2.950616 |
| 67 1 0 -5.621292 1.624215 -2.909401 |
| 68 1 0 -3.941291 1.985234 -2.443692 |
| 69 1 0 -4.364283 0.369303 -3.069322 |

1. Lowest energy conformers (14*S*,15*S*,20*S*,25*R*)-**4** for ORD calcation.

**C1**


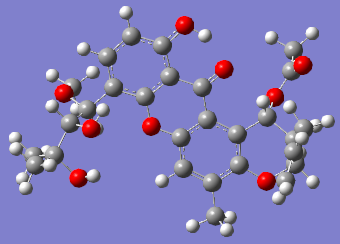


E = -1763.8133412 a.u

Standard orientation:

| Center Atomic Atomic Coordinates (Angstroms) |
| --- |
| Number Number Type X Y Z |
| 1 6 0 -2.207241 3.538963 0.738835 |
| 2 6 0 -3.216456 2.652435 0.381668 |
| 3 6 0 -2.959050 1.342422 -0.034324 |
| 4 6 0 -1.616544 0.950558 -0.081225 |
| 5 6 0 -0.557551 1.808692 0.269831 |
| 6 6 0 -0.874042 3.133837 0.687782 |
| 7 8 0 -1.359932 -0.318052 -0.494842 |
| 8 6 0 -0.082742 -0.798066 -0.556927 |
| 9 6 0 1.045446 -0.036126 -0.230480 |
| 10 6 0 0.828758 1.353214 0.198759 |
| 11 6 0 0.018108 -2.126301 -0.977635 |
| 12 6 0 1.251099 -2.732081 -1.088573 |
| 13 6 0 2.407369 -1.973717 -0.765898 |
| 14 6 0 2.329633 -0.654508 -0.328255 |
| 15 8 0 3.573109 -2.663950 -0.894361 |
| 16 6 0 4.806418 -1.938571 -0.775171 |
| 17 6 0 4.774104 -0.890068 0.326534 |
| 18 6 0 3.603700 0.075437 0.038673 |
| 19 8 0 0.078081 4.008269 1.034466 |
| 20 6 0 -4.094644 0.402634 -0.416088 |
| 21 6 0 -4.830523 -0.187360 0.816729 |
| 22 6 0 -5.498419 -1.566389 0.562376 |
| 23 6 0 -6.310878 -1.976554 1.800800 |
| 24 6 0 -6.388442 -1.589924 -0.676504 |
| 25 8 0 -4.460190 -2.529462 0.319469 |
| 26 8 0 1.760599 2.125612 0.493083 |
| 27 6 0 4.746376 -1.410613 1.758970 |
| 28 6 0 4.405942 -2.660034 2.083738 |
| 29 6 0 5.177309 -0.413735 2.807700 |
| 30 8 0 -3.932405 -0.422691 1.906091 |
| 31 6 0 1.392176 -4.158177 -1.540246 |
| 32 8 0 3.934381 0.929759 -1.099529 |
| 33 8 0 5.135128 2.296105 0.243239 |
| 34 6 0 4.650109 2.050373 -0.833296 |
| 35 6 0 4.738502 2.932126 -2.051953 |
| 36 8 0 -5.094918 1.092700 -1.163060 |
| 37 6 0 -4.739521 1.347071 -2.511440 |
| 38 1 0 -2.430166 4.551391 1.054181 |
| 39 1 0 -4.248475 2.987125 0.403294 |
| 40 1 0 -0.893048 -2.664608 -1.214146 |
| 41 1 0 5.008122 -1.455041 -1.736605 |
| 42 1 0 5.566236 -2.699196 -0.597364 |
| 43 1 0 5.687296 -0.294417 0.222723 |
| 44 1 0 3.438604 0.729962 0.886177 |
| 45 1 0 0.951966 3.557748 0.936234 |
| 46 1 0 -3.690492 -0.424867 -1.006643 |
| 47 1 0 -5.598364 0.538981 1.113736 |
| 48 1 0 -6.724346 -2.976206 1.649794 |
| 49 1 0 -5.687984 -1.989111 2.696412 |
| 50 1 0 -7.140809 -1.284763 1.975399 |
| 51 1 0 -6.855414 -2.574240 -0.759158 |
| 52 1 0 -5.814522 -1.415283 -1.586454 |
| 53 1 0 -7.173516 -0.834155 -0.616703 |
| 54 1 0 -3.820699 -2.444984 1.040735 |
| 55 1 0 4.419911 -2.988665 3.118504 |
| 56 1 0 4.104283 -3.395991 1.348510 |
| 57 1 0 6.216005 -0.104306 2.646716 |
| 58 1 0 4.583156 0.504061 2.777959 |
| 59 1 0 5.100132 -0.835958 3.811314 |
| 60 1 0 -3.422111 0.377375 2.081409 |
| 61 1 0 0.418118 -4.596965 -1.760900 |
| 62 1 0 2.017278 -4.227809 -2.434622 |
| 63 1 0 1.881301 -4.767629 -0.775213 |
| 64 1 0 4.896359 2.344508 -2.957431 |
| 65 1 0 3.792397 3.468610 -2.167260 |
| 66 1 0 5.540245 3.657308 -1.924170 |
| 67 1 0 -5.588709 1.852483 -2.971359 |
| 68 1 0 -3.856075 1.992306 -2.585143 |
| 69 1 0 -4.541493 0.412792 -3.052645 |

**C2**


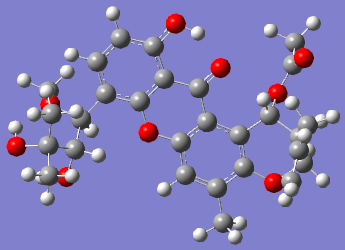


E = -1763.8131656 a.u

Standard orientation:

| Center Atomic Atomic Coordinates (Angstroms) |
| --- |
| Number Number Type X Y Z |
| 1 6 0 -2.404928 3.300133 0.484356 |
| 2 6 0 -3.339330 2.355048 0.072964 |
| 3 6 0 -2.986837 1.057979 -0.299310 |
| 4 6 0 -1.622513 0.750803 -0.262327 |
| 5 6 0 -0.636177 1.665515 0.146173 |
| 6 6 0 -1.052379 2.972624 0.531523 |
| 7 8 0 -1.272396 -0.503591 -0.659876 |
| 8 6 0 0.033879 -0.906269 -0.663776 |
| 9 6 0 1.096354 -0.080133 -0.278572 |
| 10 6 0 0.774732 1.289585 0.152928 |
| 11 6 0 0.233722 -2.222317 -1.086595 |
| 12 6 0 1.505124 -2.751305 -1.143632 |
| 13 6 0 2.596714 -1.926039 -0.765541 |
| 14 6 0 2.419223 -0.617860 -0.323196 |
| 15 8 0 3.807466 -2.542274 -0.848125 |
| 16 6 0 4.986794 -1.740764 -0.681828 |
| 17 6 0 4.849363 -0.702821 0.421202 |
| 18 6 0 3.629394 0.188049 0.098427 |
| 19 8 0 -0.174462 3.902172 0.932585 |
| 20 6 0 -3.991465 0.023067 -0.793482 |
| 21 6 0 -4.242744 -1.192521 0.145460 |
| 22 6 0 -5.322189 -1.033855 1.260466 |
| 23 6 0 -5.286406 -2.254324 2.182584 |
| 24 6 0 -5.181444 0.244676 2.088898 |
| 25 8 0 -6.616111 -1.073197 0.621155 |
| 26 8 0 1.642716 2.109347 0.507363 |
| 27 6 0 4.806452 -1.229238 1.851005 |
| 28 6 0 4.513169 -2.493318 2.164783 |
| 29 6 0 5.162276 -0.215395 2.911526 |
| 30 8 0 -4.591502 -2.311445 -0.665229 |
| 31 6 0 1.754245 -4.162056 -1.596919 |
| 32 8 0 3.944034 1.074066 -1.020158 |
| 33 8 0 5.009717 2.499290 0.374355 |
| 34 6 0 4.578269 2.234432 -0.720302 |
| 35 6 0 4.652825 3.131981 -1.928389 |
| 36 8 0 -5.249243 0.621671 -1.128305 |
| 37 6 0 -5.310908 1.160350 -2.443386 |
| 38 1 0 -2.706538 4.300965 0.770047 |
| 39 1 0 -4.385091 2.635861 0.030332 |
| 40 1 0 -0.631069 -2.812204 -1.369706 |
| 41 1 0 5.191744 -1.239900 -1.633725 |
| 42 1 0 5.786639 -2.452559 -0.479170 |
| 43 1 0 5.725559 -0.049666 0.349939 |
| 44 1 0 3.391118 0.821241 0.944791 |
| 45 1 0 0.726785 3.500963 0.896714 |
| 46 1 0 -3.578412 -0.438216 -1.697469 |
| 47 1 0 -3.286933 -1.439976 0.612244 |
| 48 1 0 -6.128791 -2.210142 2.876228 |
| 49 1 0 -5.354045 -3.183470 1.616343 |
| 50 1 0 -4.361187 -2.272836 2.764691 |
| 51 1 0 -5.905113 0.225146 2.907339 |
| 52 1 0 -5.375901 1.136243 1.493637 |
| 53 1 0 -4.181275 0.337643 2.520813 |
| 54 1 0 -6.655205 -0.314151 0.019302 |
| 55 1 0 4.511941 -2.822976 3.199343 |
| 56 1 0 4.267273 -3.240591 1.420342 |
| 57 1 0 6.196421 0.126146 2.790063 |
| 58 1 0 4.541452 0.683002 2.852042 |
| 59 1 0 5.059162 -0.635823 3.913571 |
| 60 1 0 -5.560870 -2.335749 -0.696849 |
| 61 1 0 0.820650 -4.656413 -1.869157 |
| 62 1 0 2.426198 -4.185552 -2.459049 |
| 63 1 0 2.240505 -4.748534 -0.812282 |
| 64 1 0 4.879269 2.564366 -2.832090 |
| 65 1 0 3.679147 3.608639 -2.073213 |
| 66 1 0 5.401898 3.905164 -1.766837 |
| 67 1 0 -6.303938 1.593619 -2.563700 |
| 68 1 0 -4.558134 1.942023 -2.593003 |
| 69 1 0 -5.166309 0.373260 -3.191893 |

**C3**


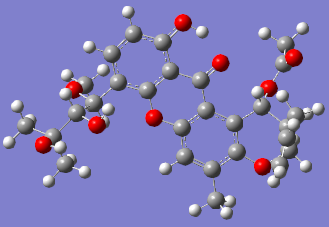


E = -1763.8119647 a.u

Standard orientation:

| Center Atomic Atomic Coordinates (Angstroms) |
| --- |
| Number Number Type X Y Z |
| 1 6 0 -2.219430 3.436735 0.695161 |
| 2 6 0 -3.206406 2.525623 0.341600 |
| 3 6 0 -2.919780 1.217495 -0.061577 |
| 4 6 0 -1.569755 0.854200 -0.102883 |
| 5 6 0 -0.530886 1.738262 0.247761 |
| 6 6 0 -0.877572 3.059313 0.654145 |
| 7 8 0 -1.279849 -0.409933 -0.512814 |
| 8 6 0 0.008444 -0.862158 -0.558573 |
| 9 6 0 1.117001 -0.073194 -0.229332 |
| 10 6 0 0.865657 1.314752 0.185673 |
| 11 6 0 0.144000 -2.191589 -0.965375 |
| 12 6 0 1.390699 -2.771710 -1.057972 |
| 13 6 0 2.526688 -1.985129 -0.732194 |
| 14 6 0 2.415482 -0.663351 -0.309572 |
| 15 8 0 3.707967 -2.651360 -0.841633 |
| 16 6 0 4.924714 -1.899074 -0.717067 |
| 17 6 0 4.858228 -0.838184 0.370863 |
| 18 6 0 3.670383 0.098547 0.059377 |
| 19 8 0 0.053016 3.957070 0.998645 |
| 20 6 0 -4.043753 0.261111 -0.442971 |
| 21 6 0 -4.834601 -0.193332 0.805655 |
| 22 6 0 -5.917401 -1.293469 0.614753 |
| 23 6 0 -7.167006 -0.777049 -0.093686 |
| 24 6 0 -5.358891 -2.539600 -0.086789 |
| 25 8 0 -6.364971 -1.651399 1.932407 |
| 26 8 0 1.778561 2.110723 0.476157 |
| 27 6 0 4.825756 -1.340439 1.809692 |
| 28 6 0 4.501867 -2.590608 2.148117 |
| 29 6 0 5.231089 -0.322561 2.848435 |
| 30 8 0 -3.909353 -0.740579 1.759953 |
| 31 6 0 1.567835 -4.199310 -1.491790 |
| 32 8 0 3.992813 0.943724 -1.087747 |
| 33 8 0 5.150965 2.354725 0.246148 |
| 34 6 0 4.682397 2.083159 -0.831374 |
| 35 6 0 4.765024 2.947996 -2.062451 |
| 36 8 0 -4.967913 0.882875 -1.330632 |
| 37 6 0 -4.464794 1.093526 -2.639437 |
| 38 1 0 -2.465351 4.446844 1.000608 |
| 39 1 0 -4.244761 2.840929 0.355794 |
| 40 1 0 -0.751310 -2.754457 -1.204432 |
| 41 1 0 5.127790 -1.423484 -1.682109 |
| 42 1 0 5.697717 -2.641647 -0.520892 |
| 43 1 0 5.759619 -0.224531 0.268831 |
| 44 1 0 3.483694 0.760576 0.896443 |
| 45 1 0 0.937467 3.526237 0.906636 |
| 46 1 0 -3.607636 -0.621365 -0.920627 |
| 47 1 0 -5.335618 0.680287 1.240776 |
| 48 1 0 -7.938348 -1.549679 -0.047694 |
| 49 1 0 -7.554403 0.112672 0.407900 |
| 50 1 0 -6.970593 -0.527533 -1.134717 |
| 51 1 0 -6.110322 -3.331061 -0.052943 |
| 52 1 0 -4.456786 -2.903432 0.411056 |
| 53 1 0 -5.120770 -2.346481 -1.136833 |
| 54 1 0 -5.575676 -1.775686 2.477973 |
| 55 1 0 4.510720 -2.905080 3.187311 |
| 56 1 0 4.219954 -3.341207 1.419885 |
| 57 1 0 6.268551 -0.004394 2.696518 |
| 58 1 0 4.627725 0.588138 2.795763 |
| 59 1 0 5.145007 -0.729723 3.857506 |
| 60 1 0 -3.376437 -0.029002 2.132823 |
| 61 1 0 0.606409 -4.661747 -1.719985 |
| 62 1 0 2.205899 -4.265599 -2.377143 |
| 63 1 0 2.059621 -4.789686 -0.713633 |
| 64 1 0 4.945526 2.350124 -2.956928 |
| 65 1 0 3.808922 3.461984 -2.196256 |
| 66 1 0 5.549495 3.692264 -1.937408 |
| 67 1 0 -5.274783 1.533980 -3.220616 |
| 68 1 0 -3.609267 1.778723 -2.641639 |
| 69 1 0 -4.161886 0.146502 -3.104975 |

**C4**


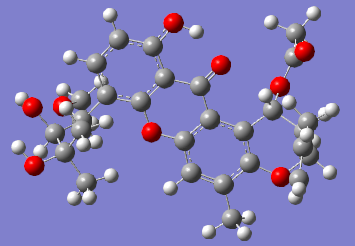


E =-1763.8114431 a.u

Standard orientation:

| Center Atomic Atomic Coordinates (Angstroms) |
| --- |
| Number Number Type X Y Z |
| 1 6 0 -2.649378 3.100056 0.549148 |
| 2 6 0 -3.537951 2.142692 0.080384 |
| 3 6 0 -3.128097 0.874186 -0.347011 |
| 4 6 0 -1.755517 0.613309 -0.298830 |
| 5 6 0 -0.811552 1.550235 0.165978 |
| 6 6 0 -1.284711 2.821738 0.600857 |
| 7 8 0 -1.339027 -0.614294 -0.725715 |
| 8 6 0 -0.014863 -0.954348 -0.730169 |
| 9 6 0 1.004009 -0.098798 -0.296850 |
| 10 6 0 0.613228 1.235114 0.186058 |
| 11 6 0 0.254832 -2.239401 -1.207070 |
| 12 6 0 1.550565 -2.705222 -1.272669 |
| 13 6 0 2.597521 -1.847401 -0.845767 |
| 14 6 0 2.351053 -0.571407 -0.345779 |
| 15 8 0 3.837727 -2.398074 -0.946372 |
| 16 6 0 4.974252 -1.550020 -0.719616 |
| 17 6 0 4.768898 -0.583901 0.436315 |
| 18 6 0 3.516379 0.267593 0.133357 |
| 19 8 0 -0.452168 3.766366 1.055114 |
| 20 6 0 -4.119420 -0.137274 -0.890379 |
| 21 6 0 -5.212483 -0.668643 0.064186 |
| 22 6 0 -4.808137 -1.422009 1.353746 |
| 23 6 0 -3.946040 -2.639795 1.031320 |
| 24 6 0 -4.148130 -0.538836 2.419847 |
| 25 8 0 -6.032295 -1.955978 1.891200 |
| 26 8 0 1.439608 2.073768 0.592480 |
| 27 6 0 4.725900 -1.196261 1.831723 |
| 28 6 0 4.566216 -2.502436 2.058658 |
| 29 6 0 4.927123 -0.220761 2.966581 |
| 30 8 0 -6.104919 0.378663 0.459300 |
| 31 6 0 1.871022 -4.081325 -1.784217 |
| 32 8 0 3.809996 1.216782 -0.937604 |
| 33 8 0 4.789675 2.619924 0.539972 |
| 34 6 0 4.388940 2.388158 -0.573499 |
| 35 6 0 4.445646 3.342488 -1.738239 |
| 36 8 0 -4.890232 0.426367 -1.968950 |
| 37 6 0 -4.158743 0.666277 -3.159303 |
| 38 1 0 -2.992587 4.073291 0.879680 |
| 39 1 0 -4.593733 2.377739 0.047417 |
| 40 1 0 -0.574892 -2.859350 -1.528050 |
| 41 1 0 5.165595 -0.987899 -1.639431 |
| 42 1 0 5.806300 -2.231570 -0.544809 |
| 43 1 0 5.614688 0.111999 0.422067 |
| 44 1 0 3.236492 0.850176 1.002598 |
| 45 1 0 0.465298 3.402565 1.020258 |
| 46 1 0 -3.572195 -1.004481 -1.272072 |
| 47 1 0 -5.777013 -1.395727 -0.533200 |
| 48 1 0 -3.821891 -3.240438 1.934868 |
| 49 1 0 -4.427074 -3.269311 0.277978 |
| 50 1 0 -2.955253 -2.352402 0.673650 |
| 51 1 0 -4.055613 -1.117963 3.341327 |
| 52 1 0 -4.754748 0.344227 2.629240 |
| 53 1 0 -3.153267 -0.200651 2.125379 |
| 54 1 0 -6.659872 -1.222604 1.953543 |
| 55 1 0 4.567874 -2.894240 3.071258 |
| 56 1 0 4.427278 -3.224908 1.263535 |
| 57 1 0 5.897406 0.280829 2.883776 |
| 58 1 0 4.176631 0.574860 2.969192 |
| 59 1 0 4.885925 -0.725984 3.933275 |
| 60 1 0 -6.527195 0.713985 -0.341545 |
| 61 1 0 0.964507 -4.608793 -2.084977 |
| 62 1 0 2.548549 -4.035352 -2.641056 |
| 63 1 0 2.380336 -4.676756 -1.021305 |
| 64 1 0 4.711416 2.827039 -2.662274 |
| 65 1 0 3.455370 3.784684 -1.879893 |
| 66 1 0 5.158459 4.137919 -1.527879 |
| 67 1 0 -4.864817 1.066245 -3.886935 |
| 68 1 0 -3.355116 1.394208 -3.002053 |
| 69 1 0 -3.727136 -0.263602 -3.551532 |

**C5**


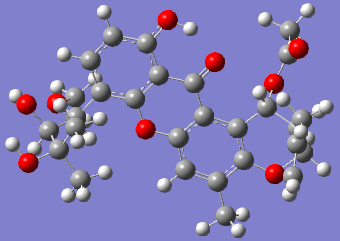


E = -1763.811443 a.u

Standard orientation:

| Center Atomic Atomic Coordinates (Angstroms) |
| --- |
| Number Number Type X Y Z |
| 1 6 0 -2.649375 3.100064 0.549157 |
| 2 6 0 -3.537951 2.142702 0.080391 |
| 3 6 0 -3.128101 0.874197 -0.347008 |
| 4 6 0 -1.755521 0.613319 -0.298834 |
| 5 6 0 -0.811552 1.550241 0.165977 |
| 6 6 0 -1.284708 2.821743 0.600866 |
| 7 8 0 -1.339033 -0.614280 -0.725730 |
| 8 6 0 -0.014871 -0.954341 -0.730178 |
| 9 6 0 1.004004 -0.098795 -0.296857 |
| 10 6 0 0.613227 1.235118 0.186054 |
| 11 6 0 0.254820 -2.239395 -1.207079 |
| 12 6 0 1.550551 -2.705223 -1.272672 |
| 13 6 0 2.597510 -1.847408 -0.845766 |
| 14 6 0 2.351047 -0.571411 -0.345780 |
| 15 8 0 3.837714 -2.398088 -0.946361 |
| 16 6 0 4.974241 -1.550033 -0.719622 |
| 17 6 0 4.768898 -0.583905 0.436304 |
| 18 6 0 3.516376 0.267587 0.133352 |
| 19 8 0 -0.452163 3.766364 1.055129 |
| 20 6 0 -4.119425 -0.137263 -0.890373 |
| 21 6 0 -5.212478 -0.668647 0.064195 |
| 22 6 0 -4.808119 -1.422018 1.353748 |
| 23 6 0 -3.946020 -2.639799 1.031308 |
| 24 6 0 -4.148112 -0.538853 2.419854 |
| 25 8 0 -6.032272 -1.955997 1.891206 |
| 26 8 0 1.439608 2.073769 0.592480 |
| 27 6 0 4.725914 -1.196252 1.831718 |
| 28 6 0 4.566211 -2.502422 2.058668 |
| 29 6 0 4.927178 -0.220745 2.966562 |
| 30 8 0 -6.104930 0.378642 0.459316 |
| 31 6 0 1.871003 -4.081326 -1.784221 |
| 32 8 0 3.809984 1.216778 -0.937608 |
| 33 8 0 4.789690 2.619907 0.539962 |
| 34 6 0 4.388941 2.388148 -0.573505 |
| 35 6 0 4.445641 3.342481 -1.738242 |
| 36 8 0 -4.890249 0.426385 -1.968933 |
| 37 6 0 -4.158769 0.666301 -3.159293 |
| 38 1 0 -2.992583 4.073298 0.879694 |
| 39 1 0 -4.593732 2.377755 0.047427 |
| 40 1 0 -0.574906 -2.859340 -1.528063 |
| 41 1 0 5.165575 -0.987919 -1.639444 |
| 42 1 0 5.806290 -2.231583 -0.544819 |
| 43 1 0 5.614688 0.111995 0.422040 |
| 44 1 0 3.236493 0.850169 1.002596 |
| 45 1 0 0.465302 3.402562 1.020271 |
| 46 1 0 -3.572201 -1.004464 -1.272079 |
| 47 1 0 -5.777000 -1.395736 -0.533194 |
| 48 1 0 -3.821860 -3.240448 1.934852 |
| 49 1 0 -4.427058 -3.269314 0.277967 |
| 50 1 0 -2.955236 -2.352403 0.673632 |
| 51 1 0 -4.055594 -1.117989 3.341329 |
| 52 1 0 -4.754729 0.344207 2.629257 |
| 53 1 0 -3.153248 -0.200666 2.125389 |
| 54 1 0 -6.659853 -1.222628 1.953549 |
| 55 1 0 4.567879 -2.894217 3.071271 |
| 56 1 0 4.427244 -3.224899 1.263554 |
| 57 1 0 5.897476 0.280813 2.883742 |
| 58 1 0 4.176711 0.574899 2.969172 |
| 59 1 0 4.885973 -0.725955 3.933263 |
| 60 1 0 -6.527195 0.713976 -0.341531 |
| 61 1 0 0.964484 -4.608796 -2.084970 |
| 62 1 0 2.548519 -4.035355 -2.641070 |
| 63 1 0 2.380328 -4.676757 -1.021315 |
| 64 1 0 4.711368 2.827032 -2.662290 |
| 65 1 0 3.455375 3.784710 -1.879865 |
| 66 1 0 5.158484 4.137891 -1.527899 |
| 67 1 0 -4.864850 1.066269 -3.886918 |
| 68 1 0 -3.355143 1.394234 -3.002045 |
| 69 1 0 -3.727163 -0.263576 -3.551527 |

**C6**


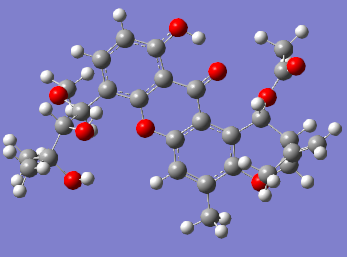


E = -1763.8112419 a.u

Standard orientation:

| Center Atomic Atomic Coordinates (Angstroms) |
| --- |
| Number Number Type X Y Z |
| 1 6 0 -2.227365 3.522993 0.802384 |
| 2 6 0 -3.230352 2.638697 0.423226 |
| 3 6 0 -2.965050 1.334667 -0.006808 |
| 4 6 0 -1.621152 0.947339 -0.044880 |
| 5 6 0 -0.568029 1.804179 0.325767 |
| 6 6 0 -0.892385 3.122379 0.759211 |
| 7 8 0 -1.357048 -0.316998 -0.468286 |
| 8 6 0 -0.077516 -0.789430 -0.533103 |
| 9 6 0 1.046435 -0.024444 -0.199122 |
| 10 6 0 0.820839 1.355143 0.258629 |
| 11 6 0 0.029987 -2.114013 -0.964406 |
| 12 6 0 1.266193 -2.711860 -1.082440 |
| 13 6 0 2.418543 -1.948578 -0.757735 |
| 14 6 0 2.334828 -0.633150 -0.308428 |
| 15 8 0 3.589499 -2.632098 -0.886973 |
| 16 6 0 4.809064 -1.877108 -0.852804 |
| 17 6 0 4.824120 -0.822578 0.251110 |
| 18 6 0 3.606670 0.106819 0.052183 |
| 19 8 0 0.053420 3.994909 1.126568 |
| 20 6 0 -4.093715 0.397350 -0.414237 |
| 21 6 0 -4.842496 -0.211807 0.801433 |
| 22 6 0 -5.501173 -1.589982 0.519901 |
| 23 6 0 -6.328875 -2.019597 1.741474 |
| 24 6 0 -6.373891 -1.600602 -0.731432 |
| 25 8 0 -4.455675 -2.545799 0.278747 |
| 26 8 0 1.746357 2.122415 0.581124 |
| 27 6 0 4.957308 -1.370523 1.667487 |
| 28 6 0 6.016878 -1.020259 2.401111 |
| 29 6 0 3.895519 -2.288008 2.223997 |
| 30 8 0 -3.957137 -0.457635 1.898954 |
| 31 6 0 1.415056 -4.133496 -1.545679 |
| 32 8 0 3.869464 1.012944 -1.063654 |
| 33 8 0 5.105518 2.354560 0.273355 |
| 34 6 0 4.572384 2.140367 -0.786014 |
| 35 6 0 4.579576 3.069866 -1.972301 |
| 36 8 0 -5.087001 1.094937 -1.163476 |
| 37 6 0 -4.715428 1.370675 -2.503301 |
| 38 1 0 -2.456347 4.530468 1.129033 |
| 39 1 0 -4.263445 2.970386 0.438654 |
| 40 1 0 -0.878659 -2.654900 -1.204766 |
| 41 1 0 4.934293 -1.389367 -1.825901 |
| 42 1 0 5.598337 -2.617829 -0.726372 |
| 43 1 0 5.705358 -0.202728 0.075212 |
| 44 1 0 3.466416 0.719352 0.937605 |
| 45 1 0 0.929621 3.547320 1.035969 |
| 46 1 0 -3.680610 -0.421109 -1.011186 |
| 47 1 0 -5.617265 0.507185 1.098272 |
| 48 1 0 -6.735312 -3.019204 1.572229 |
| 49 1 0 -5.718614 -2.040368 2.645615 |
| 50 1 0 -7.164515 -1.333953 1.912953 |
| 51 1 0 -6.837467 -2.584790 -0.832337 |
| 52 1 0 -5.787927 -1.413867 -1.631279 |
| 53 1 0 -7.161305 -0.847155 -0.673555 |
| 54 1 0 -3.827699 -2.469653 1.010945 |
| 55 1 0 6.151468 -1.385890 3.414618 |
| 56 1 0 6.779207 -0.344716 2.026394 |
| 57 1 0 2.913942 -1.803730 2.250377 |
| 58 1 0 3.782411 -3.191877 1.618992 |
| 59 1 0 4.142545 -2.590449 3.243336 |
| 60 1 0 -3.454297 0.342972 2.092448 |
| 61 1 0 0.443351 -4.576233 -1.768777 |
| 62 1 0 2.039430 -4.191773 -2.441344 |
| 63 1 0 1.909324 -4.746446 -0.786768 |
| 64 1 0 4.725215 2.522288 -2.904694 |
| 65 1 0 3.610276 3.572664 -2.032943 |
| 66 1 0 5.358386 3.819976 -1.846808 |
| 67 1 0 -5.560178 1.880767 -2.966195 |
| 68 1 0 -3.832994 2.019315 -2.555945 |
| 69 1 0 -4.507964 0.445224 -3.055998 |

**C7**


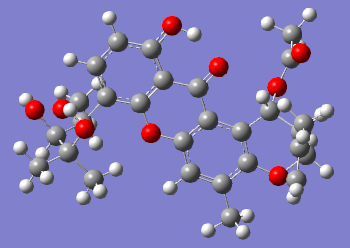


E = -1763.8111215 a.u

Standard orientation:

| Center Atomic Atomic Coordinates (Angstroms) |
| --- |
| Number Number Type X Y Z |
| 1 6 0 -2.680687 3.085285 0.498123 |
| 2 6 0 -3.560677 2.120082 0.028236 |
| 3 6 0 -3.145841 0.846285 -0.375421 |
| 4 6 0 -1.772310 0.595652 -0.319362 |
| 5 6 0 -0.836424 1.538545 0.148652 |
| 6 6 0 -1.317461 2.809525 0.574331 |
| 7 8 0 -1.344169 -0.626438 -0.750841 |
| 8 6 0 -0.018265 -0.955331 -0.755714 |
| 9 6 0 0.991977 -0.097473 -0.308249 |
| 10 6 0 0.588754 1.229720 0.183283 |
| 11 6 0 0.264072 -2.231530 -1.249425 |
| 12 6 0 1.563785 -2.685207 -1.318402 |
| 13 6 0 2.601817 -1.825198 -0.875232 |
| 14 6 0 2.342738 -0.559067 -0.356952 |
| 15 8 0 3.847585 -2.363565 -0.980716 |
| 16 6 0 4.974899 -1.509174 -0.734818 |
| 17 6 0 4.755764 -0.567659 0.438747 |
| 18 6 0 3.498773 0.280119 0.143580 |
| 19 8 0 -0.492812 3.758671 1.035606 |
| 20 6 0 -4.131916 -0.183729 -0.895656 |
| 21 6 0 -5.190731 -0.727390 0.096283 |
| 22 6 0 -4.698923 -1.405832 1.396593 |
| 23 6 0 -5.907952 -1.997559 2.140430 |
| 24 6 0 -3.673682 -2.502315 1.115005 |
| 25 8 0 -4.039822 -0.451238 2.236710 |
| 26 8 0 1.408055 2.068555 0.604396 |
| 27 6 0 4.709707 -1.209171 1.820918 |
| 28 6 0 4.578042 -2.523127 2.019401 |
| 29 6 0 4.874248 -0.253705 2.978547 |
| 30 8 0 -6.076788 0.320685 0.492650 |
| 31 6 0 1.897588 -4.050600 -1.850204 |
| 32 8 0 3.793157 1.250694 -0.907997 |
| 33 8 0 4.754304 2.633081 0.601031 |
| 34 6 0 4.359067 2.419813 -0.518199 |
| 35 6 0 4.409733 3.398695 -1.662709 |
| 36 8 0 -4.947998 0.367571 -1.948608 |
| 37 6 0 -4.258430 0.639048 -3.157469 |
| 38 1 0 -3.030551 4.062114 0.810806 |
| 39 1 0 -4.614227 2.361600 -0.026439 |
| 40 1 0 -0.559550 -2.853830 -1.581376 |
| 41 1 0 5.163581 -0.927304 -1.642896 |
| 42 1 0 5.813384 -2.185494 -0.570564 |
| 43 1 0 5.596191 0.134903 0.444013 |
| 44 1 0 3.208850 0.845297 1.020974 |
| 45 1 0 0.424759 3.393945 1.019059 |
| 46 1 0 -3.581488 -1.039439 -1.297278 |
| 47 1 0 -5.757765 -1.476299 -0.474035 |
| 48 1 0 -5.571253 -2.439267 3.080758 |
| 49 1 0 -6.647575 -1.227072 2.363027 |
| 50 1 0 -6.400414 -2.776955 1.550341 |
| 51 1 0 -3.405035 -2.994839 2.051974 |
| 52 1 0 -2.761080 -2.097936 0.678906 |
| 53 1 0 -4.082157 -3.260096 0.440005 |
| 54 1 0 -4.619678 0.317325 2.325229 |
| 55 1 0 4.577289 -2.935270 3.023893 |
| 56 1 0 4.463647 -3.232408 1.208568 |
| 57 1 0 5.827135 0.282309 2.912052 |
| 58 1 0 4.096865 0.515435 2.996310 |
| 59 1 0 4.845711 -0.782362 3.933060 |
| 60 1 0 -6.419421 0.726716 -0.314350 |
| 61 1 0 0.996196 -4.581186 -2.160981 |
| 62 1 0 2.576173 -3.985717 -2.705019 |
| 63 1 0 2.410512 -4.653345 -1.095513 |
| 64 1 0 4.681325 2.905001 -2.596872 |
| 65 1 0 3.415771 3.835038 -1.796399 |
| 66 1 0 5.115320 4.195620 -1.434369 |
| 67 1 0 -4.996115 1.027067 -3.859806 |
| 68 1 0 -3.469641 1.385649 -3.015416 |
| 69 1 0 -3.814855 -0.275521 -3.571430 |

**C8**


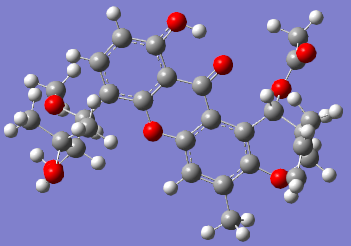


E = -1763.8110206 a.u

Standard orientation:

| Center Atomic Atomic Coordinates (Angstroms) |
| --- |
| Number Number Type X Y Z |
| 1 6 0 -2.543238 3.174750 0.440524 |
| 2 6 0 -3.446129 2.212813 0.000895 |
| 3 6 0 -3.049158 0.936189 -0.398164 |
| 4 6 0 -1.680173 0.660753 -0.346980 |
| 5 6 0 -0.725126 1.595388 0.088747 |
| 6 6 0 -1.181726 2.883682 0.492147 |
| 7 8 0 -1.293068 -0.582146 -0.747449 |
| 8 6 0 0.024131 -0.950378 -0.734046 |
| 9 6 0 1.058765 -0.100349 -0.325624 |
| 10 6 0 0.695258 1.256734 0.112476 |
| 11 6 0 0.266586 -2.256705 -1.163962 |
| 12 6 0 1.552078 -2.752227 -1.205108 |
| 13 6 0 2.615403 -1.902112 -0.802985 |
| 14 6 0 2.395790 -0.603006 -0.352578 |
| 15 8 0 3.842736 -2.485465 -0.871482 |
| 16 6 0 4.998126 -1.655671 -0.675437 |
| 17 6 0 4.813410 -0.632888 0.434664 |
| 18 6 0 3.577265 0.229863 0.097415 |
| 19 8 0 -0.334674 3.830102 0.916809 |
| 20 6 0 -4.033553 -0.105124 -0.903066 |
| 21 6 0 -4.398895 -1.259901 0.063569 |
| 22 6 0 -4.963087 -0.986546 1.492485 |
| 23 6 0 -3.908215 -0.426901 2.445402 |
| 24 6 0 -6.230961 -0.123594 1.505402 |
| 25 8 0 -5.277506 -2.285464 2.025178 |
| 26 8 0 1.535743 2.095520 0.486703 |
| 27 6 0 4.758117 -1.174416 1.858453 |
| 28 6 0 4.494791 -2.449274 2.154701 |
| 29 6 0 5.064964 -0.161571 2.935083 |
| 30 8 0 -5.306325 -2.121462 -0.639267 |
| 31 6 0 1.844931 -4.152522 -1.664412 |
| 32 8 0 3.889010 1.133020 -1.007905 |
| 33 8 0 4.891452 2.573411 0.417620 |
| 34 6 0 4.489053 2.306276 -0.687346 |
| 35 6 0 4.566958 3.213525 -1.887958 |
| 36 8 0 -5.259736 0.483130 -1.342828 |
| 37 6 0 -5.200506 1.051864 -2.644287 |
| 38 1 0 -2.875006 4.160081 0.745801 |
| 39 1 0 -4.501344 2.458155 -0.039152 |
| 40 1 0 -0.576059 -2.868878 -1.465569 |
| 41 1 0 5.207444 -1.140477 -1.618654 |
| 42 1 0 5.811982 -2.348931 -0.464689 |
| 43 1 0 5.673649 0.043153 0.385875 |
| 44 1 0 3.308251 0.849003 0.944993 |
| 45 1 0 0.578016 3.455084 0.885591 |
| 46 1 0 -3.576549 -0.615299 -1.761372 |
| 47 1 0 -3.494851 -1.855527 0.206391 |
| 48 1 0 -4.290230 -0.498547 3.466441 |
| 49 1 0 -2.988121 -1.014747 2.397125 |
| 50 1 0 -3.671212 0.616719 2.240962 |
| 51 1 0 -6.614177 -0.074565 2.527092 |
| 52 1 0 -7.016790 -0.555894 0.880258 |
| 53 1 0 -6.043892 0.891611 1.151248 |
| 54 1 0 -5.726106 -2.778599 1.322861 |
| 55 1 0 4.483269 -2.789053 3.185873 |
| 56 1 0 4.283290 -3.195845 1.399093 |
| 57 1 0 6.090093 0.212512 2.835067 |
| 58 1 0 4.417873 0.718007 2.874220 |
| 59 1 0 4.957492 -0.595614 3.930810 |
| 60 1 0 -5.960727 -1.549630 -1.065494 |
| 61 1 0 0.929119 -4.669104 -1.955375 |
| 62 1 0 2.531332 -4.152321 -2.515370 |
| 63 1 0 2.333123 -4.732070 -0.875884 |
| 64 1 0 4.831212 2.658306 -2.789137 |
| 65 1 0 3.584133 3.663718 -2.053987 |
| 66 1 0 5.290059 4.006145 -1.703882 |
| 67 1 0 -6.194948 1.440266 -2.863620 |
| 68 1 0 -4.475744 1.871303 -2.695163 |
| 69 1 0 -4.934781 0.293959 -3.391449 |

1. Lowest energy conformers (14*R*,15*R*,20*S*,25*R*)-**1** for VCD calcation.

**C1**


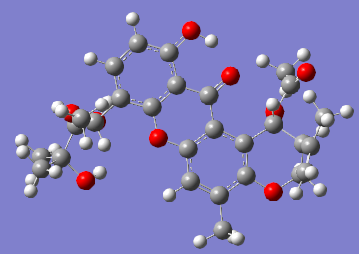


E = -1763.7224079 a.u

Standard orientation:

| Center Atomic Atomic Coordinates (Angstroms) |
| --- |
| Number Number Type X Y Z |
| 1 6 0 -2.224532 3.559154 0.578635 |
| 2 6 0 -3.224289 2.595696 0.645131 |
| 3 6 0 -2.958753 1.225047 0.565983 |
| 4 6 0 -1.618189 0.852607 0.417875 |
| 5 6 0 -0.568697 1.787403 0.344605 |
| 6 6 0 -0.893305 3.172535 0.428291 |
| 7 8 0 -1.354181 -0.478156 0.345844 |
| 8 6 0 -0.078624 -0.940136 0.188777 |
| 9 6 0 1.039972 -0.103516 0.094001 |
| 10 6 0 0.814533 1.347082 0.178467 |
| 11 6 0 0.029434 -2.331404 0.124511 |
| 12 6 0 1.259838 -2.928594 -0.044239 |
| 13 6 0 2.404883 -2.095903 -0.153138 |
| 14 6 0 2.323721 -0.708563 -0.072762 |
| 15 8 0 3.570829 -2.779671 -0.312072 |
| 16 6 0 4.747279 -2.033099 -0.655120 |
| 17 6 0 4.862461 -0.723405 0.109755 |
| 18 6 0 3.592601 0.112183 -0.166705 |
| 19 8 0 0.049398 4.120711 0.366210 |
| 20 6 0 -4.083861 0.201307 0.637346 |
| 21 6 0 -4.861481 0.065159 -0.700025 |
| 22 6 0 -5.513151 -1.327067 -0.920371 |
| 23 6 0 -6.357598 -1.793740 0.261361 |
| 24 6 0 -6.367811 -1.295054 -2.197295 |
| 25 8 0 -4.462130 -2.296629 -1.061263 |
| 26 8 0 1.736213 2.182152 0.110647 |
| 27 6 0 5.162663 -0.829274 1.600105 |
| 28 6 0 4.962374 -1.936564 2.318883 |
| 29 6 0 5.751948 0.415475 2.218858 |
| 30 8 0 -4.003570 0.240207 -1.832540 |
| 31 6 0 1.409550 -4.421525 -0.123441 |
| 32 8 0 3.637016 0.644251 -1.526783 |
| 33 8 0 4.976369 2.335314 -0.849718 |
| 34 6 0 4.311941 1.806732 -1.706289 |
| 35 6 0 4.104708 2.337076 -3.101390 |
| 36 8 0 -5.057617 0.582470 1.607391 |
| 37 6 0 -4.657877 0.358696 2.949000 |
| 38 1 0 -2.453309 4.616126 0.646558 |
| 39 1 0 -4.254096 2.908144 0.784006 |
| 40 1 0 -0.875098 -2.924504 0.201419 |
| 41 1 0 4.716343 -1.828173 -1.730435 |
| 42 1 0 5.579860 -2.704865 -0.448103 |
| 43 1 0 5.691023 -0.167364 -0.341390 |
| 44 1 0 3.547926 0.962430 0.503608 |
| 45 1 0 0.923526 3.671156 0.265615 |
| 46 1 0 -3.662804 -0.775317 0.894222 |
| 47 1 0 -5.642708 0.836445 -0.698908 |
| 48 1 0 -6.815324 -2.754301 0.013197 |
| 49 1 0 -5.752478 -1.934060 1.156968 |
| 50 1 0 -7.149008 -1.078326 0.491570 |
| 51 1 0 -6.770296 -2.291799 -2.390968 |
| 52 1 0 -5.777641 -0.984121 -3.060603 |
| 53 1 0 -7.207128 -0.600402 -2.093282 |
| 54 1 0 -3.848745 -1.954579 -1.726887 |
| 55 1 0 5.210186 -1.964450 3.375704 |
| 56 1 0 4.552438 -2.845731 1.896268 |
| 57 1 0 6.720918 0.652590 1.765459 |
| 58 1 0 5.123256 1.296123 2.059590 |
| 59 1 0 5.900748 0.292400 3.293217 |
| 60 1 0 -3.495452 1.053945 -1.728086 |
| 61 1 0 0.441035 -4.917207 -0.044021 |
| 62 1 0 1.878308 -4.720463 -1.064805 |
| 63 1 0 2.057075 -4.795589 0.674616 |
| 64 1 0 3.121185 2.812565 -3.154729 |
| 65 1 0 4.866742 3.080273 -3.329356 |
| 66 1 0 4.122520 1.533798 -3.839255 |
| 67 1 0 -5.489360 0.669226 3.581648 |
| 68 1 0 -4.447355 -0.703585 3.128393 |
| 69 1 0 -3.769336 0.944587 3.212186 |

**C2**


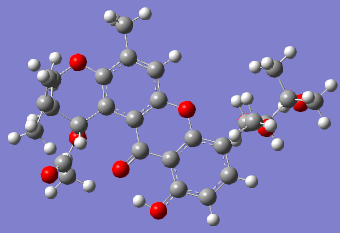


E = -1763.7219687 a.u

Standard orientation:

| Center Atomic Atomic Coordinates (Angstroms) |
| --- |
| Number Number Type X Y Z |
| 1 6 0 2.235743 -3.438425 0.668976 |
| 2 6 0 3.213085 -2.451781 0.703172 |
| 3 6 0 2.919633 -1.091002 0.568838 |
| 4 6 0 1.572740 -0.752074 0.403595 |
| 5 6 0 0.543458 -1.712401 0.362933 |
| 6 6 0 0.897291 -3.086192 0.497988 |
| 7 8 0 1.276627 0.570004 0.284392 |
| 8 6 0 -0.009870 0.995776 0.112423 |
| 9 6 0 -1.109398 0.131351 0.053876 |
| 10 6 0 -0.849537 -1.310114 0.184192 |
| 11 6 0 -0.152373 2.380334 -0.006115 |
| 12 6 0 -1.396612 2.942524 -0.192780 |
| 13 6 0 -2.522637 2.080525 -0.261110 |
| 14 6 0 -2.407857 0.699691 -0.125622 |
| 15 8 0 -3.704329 2.730442 -0.440849 |
| 16 6 0 -4.871441 1.943598 -0.723056 |
| 17 6 0 -4.939453 0.670559 0.105873 |
| 18 6 0 -3.659893 -0.150352 -0.169535 |
| 19 8 0 -0.023702 -4.056638 0.467579 |
| 20 6 0 4.031719 -0.049669 0.614552 |
| 21 6 0 4.890288 -0.091876 -0.671517 |
| 22 6 0 5.927469 1.047556 -0.881771 |
| 23 6 0 5.282620 2.438630 -0.796551 |
| 24 6 0 7.125438 0.938241 0.057217 |
| 25 8 0 6.472846 0.858048 -2.197703 |
| 26 8 0 -1.752085 -2.167433 0.143771 |
| 27 6 0 -5.201560 0.846056 1.597041 |
| 28 6 0 -5.063949 2.008813 2.238980 |
| 29 6 0 -5.682873 -0.393513 2.312296 |
| 30 8 0 4.023355 -0.020600 -1.815003 |
| 31 6 0 -1.581116 4.427190 -0.331015 |
| 32 8 0 -3.720966 -0.724792 -1.511186 |
| 33 8 0 -5.015345 -2.417680 -0.755943 |
| 34 6 0 -4.378353 -1.904058 -1.641814 |
| 35 6 0 -4.190007 -2.472270 -3.024569 |
| 36 8 0 4.908245 -0.282627 1.712918 |
| 37 6 0 4.345163 0.015122 2.979826 |
| 38 1 0 2.486635 -4.486848 0.778285 |
| 39 1 0 4.247631 -2.739399 0.860727 |
| 40 1 0 0.736858 2.998695 0.042598 |
| 41 1 0 -4.858596 1.688648 -1.787737 |
| 42 1 0 -5.714223 2.606677 -0.529973 |
| 43 1 0 -5.768487 0.076546 -0.293504 |
| 44 1 0 -3.585038 -0.978271 0.525235 |
| 45 1 0 -0.907414 -3.631084 0.348477 |
| 46 1 0 3.580258 0.942714 0.704930 |
| 47 1 0 5.443070 -1.039524 -0.688455 |
| 48 1 0 6.013222 3.186884 -1.110529 |
| 49 1 0 4.413016 2.512631 -1.453864 |
| 50 1 0 4.967170 2.684129 0.221754 |
| 51 1 0 7.879081 1.666658 -0.251785 |
| 52 1 0 7.574519 -0.055491 -0.004191 |
| 53 1 0 6.848469 1.126782 1.092937 |
| 54 1 0 5.727975 0.707907 -2.796516 |
| 55 1 0 -5.287375 2.087716 3.298612 |
| 56 1 0 -4.729746 2.915343 1.749159 |
| 57 1 0 -6.625216 -0.751216 1.883201 |
| 58 1 0 -4.978095 -1.225268 2.223591 |
| 59 1 0 -5.844410 -0.199256 3.374220 |
| 60 1 0 3.516230 -0.837875 -1.882113 |
| 61 1 0 -0.624377 4.948397 -0.273609 |
| 62 1 0 -2.057516 4.677529 -1.282651 |
| 63 1 0 -2.236158 4.817707 0.452813 |
| 64 1 0 -3.199287 -2.931758 -3.084550 |
| 65 1 0 -4.942876 -3.235225 -3.214493 |
| 66 1 0 -4.237523 -1.691813 -3.785275 |
| 67 1 0 5.121379 -0.174136 3.721076 |
| 68 1 0 4.039023 1.067737 3.040381 |
| 69 1 0 3.478328 -0.618747 3.199513 |

**C3**


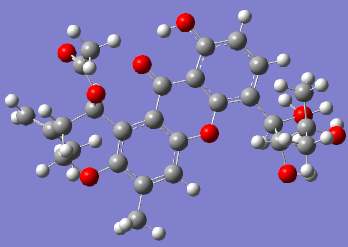


E = -1763.721509 a.u

Standard orientation:

| Center Atomic Atomic Coordinates (Angstroms) |
| --- |
| Number Number Type X Y Z |
| 1 6 0 2.435202 3.182743 -0.950014 |
| 2 6 0 3.349175 2.134463 -0.979106 |
| 3 6 0 2.979218 0.804762 -0.777255 |
| 4 6 0 1.617725 0.565252 -0.564474 |
| 5 6 0 0.651672 1.585635 -0.522615 |
| 6 6 0 1.085701 2.928603 -0.718154 |
| 7 8 0 1.248932 -0.735941 -0.401379 |
| 8 6 0 -0.056691 -1.080554 -0.191538 |
| 9 6 0 -1.098537 -0.148382 -0.119772 |
| 10 6 0 -0.756454 1.273260 -0.292503 |
| 11 6 0 -0.277503 -2.452310 -0.047377 |
| 12 6 0 -1.548767 -2.934782 0.178137 |
| 13 6 0 -2.617892 -2.004489 0.260192 |
| 14 6 0 -2.424061 -0.634179 0.103231 |
| 15 8 0 -3.836218 -2.578941 0.462322 |
| 16 6 0 -4.922767 -1.721048 0.838293 |
| 17 6 0 -4.974241 -0.434890 0.017805 |
| 18 6 0 -3.620283 0.293765 0.170152 |
| 19 8 0 0.228058 3.957600 -0.691435 |
| 20 6 0 3.961693 -0.359328 -0.845460 |
| 21 6 0 4.245758 -1.094133 0.496244 |
| 22 6 0 5.364778 -0.511770 1.414461 |
| 23 6 0 5.254928 0.995220 1.653957 |
| 24 6 0 5.360768 -1.250478 2.754425 |
| 25 8 0 6.634806 -0.828654 0.804991 |
| 26 8 0 -1.604714 2.182876 -0.251838 |
| 27 6 0 -5.420734 -0.608325 -1.429366 |
| 28 6 0 -6.531983 0.005703 -1.843590 |
| 29 6 0 -4.603380 -1.457131 -2.373133 |
| 30 8 0 4.564909 -2.450617 0.198987 |
| 31 6 0 -1.818704 -4.404009 0.341418 |
| 32 8 0 -3.558667 0.925868 1.485874 |
| 33 8 0 -4.793718 2.669522 0.744470 |
| 34 6 0 -4.127087 2.152706 1.605187 |
| 35 6 0 -3.798682 2.765342 2.942349 |
| 36 8 0 5.206973 0.031145 -1.435989 |
| 37 6 0 5.219527 -0.006736 -2.858118 |
| 38 1 0 2.750214 4.207268 -1.109262 |
| 39 1 0 4.392426 2.353785 -1.173738 |
| 40 1 0 0.572736 -3.122465 -0.110225 |
| 41 1 0 -4.810228 -1.473769 1.899719 |
| 42 1 0 -5.819871 -2.326438 0.711073 |
| 43 1 0 -5.715530 0.208363 0.495966 |
| 44 1 0 -3.546045 1.084099 -0.570577 |
| 45 1 0 -0.675384 3.593560 -0.531387 |
| 46 1 0 3.513683 -1.139429 -1.471106 |
| 47 1 0 3.307189 -1.116969 1.054207 |
| 48 1 0 6.005959 1.298857 2.387203 |
| 49 1 0 5.430092 1.564498 0.741714 |
| 50 1 0 4.270023 1.271139 2.040543 |
| 51 1 0 6.229439 -0.944140 3.341210 |
| 52 1 0 5.402998 -2.330860 2.614218 |
| 53 1 0 4.458537 -1.012873 3.324400 |
| 54 1 0 6.652238 -0.383285 -0.055927 |
| 55 1 0 -6.885727 -0.089545 -2.865753 |
| 56 1 0 -7.120322 0.637619 -1.185982 |
| 57 1 0 -3.581515 -1.078208 -2.477307 |
| 58 1 0 -4.519429 -2.489262 -2.021757 |
| 59 1 0 -5.053870 -1.472439 -3.367305 |
| 60 1 0 5.532631 -2.500482 0.150095 |
| 61 1 0 -0.896267 -4.981697 0.267750 |
| 62 1 0 -2.284809 -4.613407 1.307981 |
| 63 1 0 -2.516031 -4.764624 -0.419977 |
| 64 1 0 -2.783478 3.170384 2.903279 |
| 65 1 0 -4.492091 3.577172 3.154979 |
| 66 1 0 -3.826642 2.021030 3.739346 |
| 67 1 0 6.206760 0.328069 -3.176694 |
| 68 1 0 5.048730 -1.026478 -3.220832 |
| 69 1 0 4.460428 0.658197 -3.284150 |

**C4**


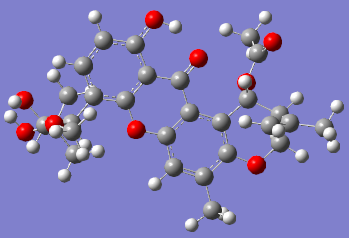


E = -1763.7214779 a.u

Standard orientation:

| Center Atomic Atomic Coordinates (Angstroms) |
| --- |
| Number Number Type X Y Z |
| 1 6 0 -2.719921 2.937856 1.073591 |
| 2 6 0 -3.578267 1.847389 1.067466 |
| 3 6 0 -3.140387 0.540818 0.821357 |
| 4 6 0 -1.770719 0.374089 0.597263 |
| 5 6 0 -0.857067 1.446236 0.593488 |
| 6 6 0 -1.358130 2.757286 0.837195 |
| 7 8 0 -1.326625 -0.896504 0.368601 |
| 8 6 0 -0.003446 -1.158651 0.147903 |
| 9 6 0 0.984768 -0.168651 0.113774 |
| 10 6 0 0.564343 1.222186 0.347123 |
| 11 6 0 0.298025 -2.509158 -0.045841 |
| 12 6 0 1.593394 -2.909784 -0.294861 |
| 13 6 0 2.605891 -1.917385 -0.349879 |
| 14 6 0 2.334344 -0.568339 -0.131353 |
| 15 8 0 3.850886 -2.407771 -0.601924 |
| 16 6 0 4.868457 -1.461641 -0.946917 |
| 17 6 0 4.855895 -0.257152 -0.006711 |
| 18 6 0 3.482230 0.421558 -0.136029 |
| 19 8 0 -0.554745 3.827763 0.851631 |
| 20 6 0 -4.097829 -0.635296 0.857598 |
| 21 6 0 -5.213612 -0.696560 -0.211644 |
| 22 6 0 -4.843904 -0.740191 -1.713427 |
| 23 6 0 -4.235866 0.561112 -2.251308 |
| 24 6 0 -3.953505 -1.939897 -2.026249 |
| 25 8 0 -6.078175 -0.993598 -2.409724 |
| 26 8 0 1.363126 2.176859 0.342125 |
| 27 6 0 5.279956 -0.606331 1.413091 |
| 28 6 0 4.437138 -0.704774 2.442239 |
| 29 6 0 6.763915 -0.813732 1.593617 |
| 30 8 0 -6.138611 0.383104 -0.046264 |
| 31 6 0 1.948656 -4.353703 -0.510150 |
| 32 8 0 3.412631 1.124012 -1.414012 |
| 33 8 0 4.542952 2.878641 -0.543095 |
| 34 6 0 3.918648 2.383342 -1.447399 |
| 35 6 0 3.582951 3.059566 -2.751331 |
| 36 8 0 -4.843178 -0.654535 2.089725 |
| 37 6 0 -4.078739 -0.968525 3.241668 |
| 38 1 0 -3.084438 3.940541 1.263230 |
| 39 1 0 -4.631823 2.007305 1.256498 |
| 40 1 0 -0.508643 -3.232565 -0.002659 |
| 41 1 0 4.699543 -1.128555 -1.976645 |
| 42 1 0 5.801374 -2.022396 -0.910145 |
| 43 1 0 5.590287 0.456592 -0.393791 |
| 44 1 0 3.363094 1.163911 0.646344 |
| 45 1 0 0.366493 3.516722 0.678305 |
| 46 1 0 -3.525137 -1.564929 0.785381 |
| 47 1 0 -5.741931 -1.637773 -0.012981 |
| 48 1 0 -4.167584 0.488383 -3.339062 |
| 49 1 0 -4.863366 1.419355 -2.003641 |
| 50 1 0 -3.236706 0.750013 -1.855616 |
| 51 1 0 -3.853733 -2.039782 -3.109156 |
| 52 1 0 -4.397595 -2.864094 -1.647019 |
| 53 1 0 -2.955105 -1.825138 -1.600192 |
| 54 1 0 -6.718890 -0.336659 -2.103762 |
| 55 1 0 4.797351 -0.944232 3.437928 |
| 56 1 0 3.368469 -0.550668 2.349396 |
| 57 1 0 7.151550 -1.604374 0.941785 |
| 58 1 0 7.318264 0.098344 1.344888 |
| 59 1 0 7.007402 -1.086294 2.621940 |
| 60 1 0 -6.530102 0.294000 0.831837 |
| 61 1 0 1.065346 -4.990118 -0.438187 |
| 62 1 0 2.406756 -4.504070 -1.491511 |
| 63 1 0 2.682556 -4.691885 0.226587 |
| 64 1 0 2.543521 3.397705 -2.714729 |
| 65 1 0 4.228930 3.924042 -2.894140 |
| 66 1 0 3.675974 2.370871 -3.592283 |
| 67 1 0 -4.769574 -0.976378 4.084649 |
| 68 1 0 -3.614304 -1.958715 3.149072 |
| 69 1 0 -3.297363 -0.222960 3.425540 |

**C5**

E = -1763.7214011 a.u

Standard orientation:

| Center Atomic Atomic Coordinates (Angstroms) |
| --- |
| Number Number Type X Y Z |
| 1 6 0 2.650781 -2.983823 1.003924 |
| 2 6 0 3.525242 -1.906526 1.033343 |
| 3 6 0 3.109491 -0.588161 0.813693 |
| 4 6 0 1.744780 -0.395290 0.580568 |
| 5 6 0 0.815116 -1.453000 0.542110 |
| 6 6 0 1.294709 -2.777276 0.756708 |
| 7 8 0 1.322499 0.886388 0.376092 |
| 8 6 0 0.004494 1.174659 0.156286 |
| 9 6 0 -1.000086 0.202448 0.100094 |
| 10 6 0 -0.600320 -1.200805 0.290588 |
| 11 6 0 -0.273437 2.532825 -0.015479 |
| 12 6 0 -1.561744 2.959957 -0.256644 |
| 13 6 0 -2.592427 1.986805 -0.324151 |
| 14 6 0 -2.342676 0.629991 -0.134544 |
| 15 8 0 -3.828102 2.506634 -0.558363 |
| 16 6 0 -4.902275 1.595835 -0.839112 |
| 17 6 0 -4.863781 0.350729 0.032631 |
| 18 6 0 -3.500703 -0.343962 -0.179709 |
| 19 8 0 0.475819 -3.835962 0.734717 |
| 20 6 0 4.084297 0.571866 0.884804 |
| 21 6 0 5.208304 0.641097 -0.175291 |
| 22 6 0 4.850265 0.724416 -1.678229 |
| 23 6 0 4.226213 -0.554819 -2.249668 |
| 24 6 0 3.981014 1.944671 -1.970358 |
| 25 8 0 6.093497 0.973992 -2.359760 |
| 26 8 0 -1.411420 -2.144848 0.244572 |
| 27 6 0 -5.188234 0.546481 1.508943 |
| 28 6 0 -5.192481 1.737669 2.112407 |
| 29 6 0 -5.559705 -0.712827 2.254481 |
| 30 8 0 6.116033 -0.455973 -0.028661 |
| 31 6 0 -1.888865 4.413510 -0.452210 |
| 32 8 0 -3.464637 -0.967826 -1.499702 |
| 33 8 0 -4.598644 -2.758034 -0.711842 |
| 34 6 0 -3.992599 -2.213276 -1.600866 |
| 35 6 0 -3.706558 -2.808412 -2.955079 |
| 36 8 0 4.821025 0.550267 2.121939 |
| 37 6 0 4.053600 0.850833 3.275493 |
| 38 1 0 2.998413 -3.996272 1.172178 |
| 39 1 0 4.574226 -2.086830 1.229333 |
| 40 1 0 0.545566 3.241439 0.038884 |
| 41 1 0 -4.835559 1.308697 -1.893490 |
| 42 1 0 -5.812442 2.175875 -0.689387 |
| 43 1 0 -5.615776 -0.338087 -0.367106 |
| 44 1 0 -3.363738 -1.135693 0.547177 |
| 45 1 0 -0.437702 -3.507344 0.553518 |
| 46 1 0 3.526233 1.511586 0.830922 |
| 47 1 0 5.749191 1.569433 0.048525 |
| 48 1 0 4.169823 -0.457623 -3.336175 |
| 49 1 0 4.837128 -1.428402 -2.014569 |
| 50 1 0 3.220216 -0.735504 -1.867658 |
| 51 1 0 3.892579 2.071244 -3.051477 |
| 52 1 0 4.435627 2.852887 -1.565793 |
| 53 1 0 2.977128 1.835335 -1.555917 |
| 54 1 0 6.721837 0.300931 -2.063378 |
| 55 1 0 -5.455634 1.827345 3.162047 |
| 56 1 0 -4.939136 2.657976 1.600309 |
| 57 1 0 -6.448321 -1.179127 1.815107 |
| 58 1 0 -4.771349 -1.469663 2.210897 |
| 59 1 0 -5.769372 -0.502941 3.304935 |
| 60 1 0 6.502119 -0.393252 0.854083 |
| 61 1 0 -0.991717 5.030178 -0.379843 |
| 62 1 0 -2.351182 4.585386 -1.427991 |
| 63 1 0 -2.608891 4.760023 0.294357 |
| 64 1 0 -2.672856 -3.165326 -2.971425 |
| 65 1 0 -4.371907 -3.651125 -3.134264 |
| 66 1 0 -3.810813 -2.064879 -3.746492 |
| 67 1 0 4.737865 0.825619 4.123468 |
| 68 1 0 3.608318 1.851462 3.203307 |
| 69 1 0 3.257095 0.115886 3.435571 |

**C6**

E = -1763.7212116 a.u

Standard orientation:

| Center Atomic Atomic Coordinates (Angstroms) |
| --- |
| Number Number Type X Y Z |
| 1 6 0 -2.559908 -3.005001 -1.072020 |
| 2 6 0 -3.444148 -1.931932 -1.090259 |
| 3 6 0 -3.035036 -0.616855 -0.867396 |
| 4 6 0 -1.671763 -0.414806 -0.636700 |
| 5 6 0 -0.735990 -1.463094 -0.604301 |
| 6 6 0 -1.205544 -2.790328 -0.825923 |
| 7 8 0 -1.271156 0.871169 -0.433693 |
| 8 6 0 0.042777 1.173524 -0.203492 |
| 9 6 0 1.056801 0.210462 -0.141833 |
| 10 6 0 0.676270 -1.196377 -0.345194 |
| 11 6 0 0.303746 2.533868 -0.025859 |
| 12 6 0 1.585477 2.973670 0.226792 |
| 13 6 0 2.625570 2.010734 0.302696 |
| 14 6 0 2.392267 0.651686 0.108592 |
| 15 8 0 3.853111 2.542594 0.550784 |
| 16 6 0 4.931698 1.642634 0.849031 |
| 17 6 0 4.918526 0.395793 -0.020946 |
| 18 6 0 3.558747 -0.311205 0.170199 |
| 19 8 0 -0.377126 -3.842448 -0.810135 |
| 20 6 0 -4.002778 0.554163 -0.896670 |
| 21 6 0 -4.404430 1.169594 0.468337 |
| 22 6 0 -4.999728 0.297965 1.618273 |
| 23 6 0 -6.257026 -0.484350 1.218808 |
| 24 6 0 -3.962021 -0.621239 2.259731 |
| 25 8 0 -5.343063 1.233482 2.656053 |
| 26 8 0 1.496024 -2.132492 -0.303841 |
| 27 6 0 5.264906 0.591822 -1.492285 |
| 28 6 0 5.268417 1.782106 -2.097500 |
| 29 6 0 5.658524 -0.665511 -2.229783 |
| 30 8 0 -5.303460 2.252798 0.190172 |
| 31 6 0 1.895640 4.430035 0.428029 |
| 32 8 0 3.507080 -0.935417 1.489814 |
| 33 8 0 4.672260 -2.714649 0.722735 |
| 34 6 0 4.047227 -2.174695 1.601512 |
| 35 6 0 3.747703 -2.769868 2.952876 |
| 36 8 0 -5.211347 0.233545 -1.588712 |
| 37 6 0 -5.106519 0.298054 -3.005144 |
| 38 1 0 -2.901007 -4.018296 -1.248229 |
| 39 1 0 -4.494176 -2.116070 -1.286686 |
| 40 1 0 -0.523394 3.232658 -0.083718 |
| 41 1 0 4.851964 1.356712 1.902833 |
| 42 1 0 5.838346 2.231179 0.711836 |
| 43 1 0 5.670326 -0.285356 0.392119 |
| 44 1 0 3.440294 -1.104137 -0.558439 |
| 45 1 0 0.533141 -3.507399 -0.626022 |
| 46 1 0 -3.518178 1.383774 -1.428437 |
| 47 1 0 -3.510563 1.641777 0.881239 |
| 48 1 0 -6.668515 -0.967399 2.107867 |
| 49 1 0 -7.029506 0.175840 0.815806 |
| 50 1 0 -6.047783 -1.249740 0.469636 |
| 51 1 0 -4.370380 -1.003457 3.198274 |
| 52 1 0 -3.048681 -0.070893 2.499292 |
| 53 1 0 -3.707053 -1.470449 1.626649 |
| 54 1 0 -5.780007 1.984031 2.227694 |
| 55 1 0 5.547185 1.872556 -3.143027 |
| 56 1 0 4.998981 2.700907 -1.590928 |
| 57 1 0 6.541030 -1.126574 -1.772929 |
| 58 1 0 4.873887 -1.426918 -2.201434 |
| 59 1 0 5.887422 -0.454691 -3.276026 |
| 60 1 0 -5.939489 1.926960 -0.462789 |
| 61 1 0 0.992705 5.037232 0.349273 |
| 62 1 0 2.347928 4.604573 1.407996 |
| 63 1 0 2.618296 4.785308 -0.311893 |
| 64 1 0 2.717900 -3.138258 2.955212 |
| 65 1 0 4.419652 -3.604674 3.144044 |
| 66 1 0 3.831900 -2.023305 3.743803 |
| 67 1 0 -6.090655 0.054598 -3.405204 |
| 68 1 0 -4.821985 1.305991 -3.331209 |
| 69 1 0 -4.374604 -0.420235 -3.389450 |

**C7**

E = -1763.7211606 a.u

Standard orientation:

| Center Atomic Atomic Coordinates (Angstroms) |
| --- |
| Number Number Type X Y Z |
| 1 6 0 2.743603 2.917779 -1.106554 |
| 2 6 0 3.597354 1.822940 -1.100424 |
| 3 6 0 3.159362 0.519764 -0.842267 |
| 4 6 0 1.789099 0.359220 -0.620570 |
| 5 6 0 0.880286 1.434837 -0.612630 |
| 6 6 0 1.384386 2.744313 -0.856010 |
| 7 8 0 1.337069 -0.911167 -0.408646 |
| 8 6 0 0.014151 -1.168417 -0.187156 |
| 9 6 0 -0.967657 -0.173270 -0.135141 |
| 10 6 0 -0.539921 1.217187 -0.357911 |
| 11 6 0 -0.296044 -2.519969 -0.013682 |
| 12 6 0 -1.592839 -2.915998 0.234857 |
| 13 6 0 -2.598030 -1.917974 0.311012 |
| 14 6 0 -2.318321 -0.567741 0.111806 |
| 15 8 0 -3.845681 -2.403826 0.562460 |
| 16 6 0 -4.852541 -1.456401 0.933166 |
| 17 6 0 -4.839099 -0.235037 0.014769 |
| 18 6 0 -3.458688 0.430300 0.141581 |
| 19 8 0 0.585336 3.819034 -0.863262 |
| 20 6 0 4.120184 -0.655734 -0.850278 |
| 21 6 0 5.185696 -0.712688 0.273820 |
| 22 6 0 4.702369 -0.743861 1.742496 |
| 23 6 0 3.697044 -1.867469 1.986821 |
| 24 6 0 5.918194 -0.921451 2.667480 |
| 25 8 0 4.025606 0.475530 2.071087 |
| 26 8 0 -1.334350 2.175774 -0.339776 |
| 27 6 0 -5.280477 -0.555175 -1.406573 |
| 28 6 0 -4.447862 -0.650291 -2.444288 |
| 29 6 0 -6.768862 -0.737928 -1.576967 |
| 30 8 0 6.077355 0.397343 0.159512 |
| 31 6 0 -1.956904 -4.360874 0.428965 |
| 32 8 0 -3.370928 1.112173 1.429524 |
| 33 8 0 -4.495944 2.889922 0.599543 |
| 34 6 0 -3.864036 2.375402 1.487891 |
| 35 6 0 -3.503921 3.028599 2.797013 |
| 36 8 0 4.928452 -0.653408 -2.043331 |
| 37 6 0 4.224226 -0.941049 -3.240125 |
| 38 1 0 3.111013 3.917460 -1.306543 |
| 39 1 0 4.647659 1.983820 -1.306076 |
| 40 1 0 0.505524 -3.248027 -0.071566 |
| 41 1 0 -4.672290 -1.142829 1.967164 |
| 42 1 0 -5.790332 -2.009030 0.894392 |
| 43 1 0 -5.563419 0.477669 0.422175 |
| 44 1 0 -3.340593 1.183762 -0.630209 |
| 45 1 0 -0.334748 3.511630 -0.678300 |
| 46 1 0 3.550336 -1.589185 -0.820800 |
| 47 1 0 5.745449 -1.640218 0.089896 |
| 48 1 0 3.432322 -1.891851 3.046144 |
| 49 1 0 2.779962 -1.718761 1.418360 |
| 50 1 0 4.123139 -2.839666 1.722776 |
| 51 1 0 5.583895 -0.903585 3.707051 |
| 52 1 0 6.644061 -0.120400 2.521075 |
| 53 1 0 6.426621 -1.873601 2.485446 |
| 54 1 0 4.593258 1.211947 1.806159 |
| 55 1 0 -4.819821 -0.868974 -3.440478 |
| 56 1 0 -3.376321 -0.513484 -2.357711 |
| 57 1 0 -7.162619 -1.531019 -0.931722 |
| 58 1 0 -7.307060 0.179070 -1.311381 |
| 59 1 0 -7.025358 -0.993391 -2.606554 |
| 60 1 0 6.421579 0.397749 -0.743161 |
| 61 1 0 -1.078266 -5.001970 0.341534 |
| 62 1 0 -2.410216 -4.524177 1.410511 |
| 63 1 0 -2.697184 -4.682462 -0.308845 |
| 64 1 0 -2.458669 3.347537 2.755193 |
| 65 1 0 -4.132963 3.902417 2.957542 |
| 66 1 0 -3.601437 2.330313 3.629586 |
| 67 1 0 4.957968 -0.933895 -4.046087 |
| 68 1 0 3.754333 -1.931716 -3.191197 |
| 69 1 0 3.454341 -0.190194 -3.447857 |

**C8**

E = -1763.7210655 a.u

Standard orientation:

| Center Atomic Atomic Coordinates (Angstroms) |
| --- |
| Number Number Type X Y Z |
| 1 6 0 -2.674429 -2.963948 -1.035732 |
| 2 6 0 -3.544080 -1.882007 -1.065299 |
| 3 6 0 -3.127852 -0.566806 -0.834008 |
| 4 6 0 -1.762356 -0.380450 -0.603314 |
| 5 6 0 -0.837720 -1.441781 -0.561086 |
| 6 6 0 -1.320785 -2.764420 -0.775117 |
| 7 8 0 -1.331758 0.900687 -0.415121 |
| 8 6 0 -0.013836 1.183716 -0.194702 |
| 9 6 0 0.984213 0.206388 -0.121233 |
| 10 6 0 0.576744 -1.196063 -0.302021 |
| 11 6 0 0.273086 2.542636 -0.042688 |
| 12 6 0 1.562948 2.964780 0.198451 |
| 13 6 0 2.586150 1.985856 0.287179 |
| 14 6 0 2.328088 0.628336 0.115673 |
| 15 8 0 3.824523 2.500483 0.522719 |
| 16 6 0 4.887409 1.585777 0.830850 |
| 17 6 0 4.849181 0.327383 -0.021736 |
| 18 6 0 3.478292 -0.353343 0.185237 |
| 19 8 0 -0.506376 -3.827331 -0.746327 |
| 20 6 0 -4.105423 0.593685 -0.878464 |
| 21 6 0 -5.184049 0.659747 0.232662 |
| 22 6 0 -4.717472 0.728205 1.705472 |
| 23 6 0 -3.730724 1.870778 1.937352 |
| 24 6 0 -5.945752 0.907401 2.613451 |
| 25 8 0 -4.027393 -0.474451 2.066474 |
| 26 8 0 1.383342 -2.143779 -0.244239 |
| 27 6 0 5.192738 0.497878 -1.496822 |
| 28 6 0 5.216112 1.679896 -2.117729 |
| 29 6 0 5.559734 -0.775929 -2.219580 |
| 30 8 0 -6.059391 -0.464594 0.132159 |
| 31 6 0 1.899191 4.419001 0.373491 |
| 32 8 0 3.423066 -0.959087 1.512986 |
| 33 8 0 4.552496 -2.768733 0.763923 |
| 34 6 0 3.938102 -2.207539 1.637040 |
| 35 6 0 3.626576 -2.782719 2.994236 |
| 36 8 0 -4.900437 0.553309 -2.079717 |
| 37 6 0 -4.187405 0.825693 -3.274860 |
| 38 1 0 -3.025225 -3.973666 -1.213951 |
| 39 1 0 -4.589848 -2.063220 -1.277059 |
| 40 1 0 -0.540791 3.255910 -0.111513 |
| 41 1 0 4.805682 1.315351 1.888649 |
| 42 1 0 5.804247 2.155920 0.683351 |
| 43 1 0 5.590865 -0.361176 0.397318 |
| 44 1 0 3.342310 -1.153761 -0.532155 |
| 45 1 0 0.406097 -3.502044 -0.554577 |
| 46 1 0 -3.549355 1.535713 -0.863565 |
| 47 1 0 -5.754100 1.575490 0.023033 |
| 48 1 0 -3.477104 1.919676 2.998537 |
| 49 1 0 -2.805812 1.724571 1.381042 |
| 50 1 0 -4.168076 2.831309 1.650091 |
| 51 1 0 -5.622811 0.914585 3.656732 |
| 52 1 0 -6.659097 0.093796 2.475036 |
| 53 1 0 -6.464956 1.848709 2.407138 |
| 54 1 0 -4.583328 -1.223990 1.813908 |
| 55 1 0 5.492383 1.751225 -3.165441 |
| 56 1 0 4.965448 2.609974 -1.622211 |
| 57 1 0 6.436147 -1.246801 -1.760993 |
| 58 1 0 4.761650 -1.522594 -2.177211 |
| 59 1 0 5.786896 -0.583021 -3.269674 |
| 60 1 0 -6.392740 -0.489066 -0.774242 |
| 61 1 0 1.006783 5.040664 0.285519 |
| 62 1 0 2.356206 4.603119 1.349560 |
| 63 1 0 2.626343 4.749023 -0.373669 |
| 64 1 0 2.586416 -3.120510 3.003089 |
| 65 1 0 4.275231 -3.634601 3.190501 |
| 66 1 0 3.734957 -2.031953 3.778314 |
| 67 1 0 -4.911705 0.788342 -4.088479 |
| 68 1 0 -3.734302 1.824839 -3.243125 |
| 69 1 0 -3.403055 0.083254 -3.457010 |

**C9**

E = -1763.7210329 a.u

Standard orientation:

| Center Atomic Atomic Coordinates (Angstroms) |
| --- |
| Number Number Type X Y Z |
| 1 6 0 -2.247328 3.550939 0.635126 |
| 2 6 0 -3.240847 2.579921 0.680200 |
| 3 6 0 -2.965785 1.212584 0.577997 |
| 4 6 0 -1.622444 0.852100 0.426873 |
| 5 6 0 -0.578751 1.794684 0.377001 |
| 6 6 0 -0.912589 3.175831 0.485646 |
| 7 8 0 -1.349547 -0.475755 0.329199 |
| 8 6 0 -0.070844 -0.926270 0.166691 |
| 9 6 0 1.043001 -0.081012 0.095618 |
| 10 6 0 0.808758 1.366592 0.212885 |
| 11 6 0 0.045913 -2.315401 0.072491 |
| 12 6 0 1.280837 -2.901662 -0.102673 |
| 13 6 0 2.421356 -2.059982 -0.185865 |
| 14 6 0 2.331576 -0.674678 -0.076497 |
| 15 8 0 3.595450 -2.732821 -0.339967 |
| 16 6 0 4.753377 -1.973834 -0.715104 |
| 17 6 0 4.886427 -0.669392 0.066622 |
| 18 6 0 3.597244 0.155694 -0.143925 |
| 19 8 0 0.024124 4.130929 0.447573 |
| 20 6 0 -4.083204 0.179461 0.632567 |
| 21 6 0 -4.857551 0.054177 -0.707651 |
| 22 6 0 -5.495742 -1.341201 -0.947310 |
| 23 6 0 -6.333789 -1.833630 0.228519 |
| 24 6 0 -6.352378 -1.299258 -2.222540 |
| 25 8 0 -4.435132 -2.298041 -1.103425 |
| 26 8 0 1.726062 2.207108 0.175048 |
| 27 6 0 5.284764 -0.829439 1.529344 |
| 28 6 0 6.433154 -0.293829 1.951097 |
| 29 6 0 4.380858 -1.577660 2.479253 |
| 30 8 0 -4.000131 0.252598 -1.836646 |
| 31 6 0 1.440428 -4.391638 -0.212688 |
| 32 8 0 3.614030 0.742210 -1.482057 |
| 33 8 0 4.971407 2.407487 -0.775106 |
| 34 6 0 4.282881 1.913719 -1.632136 |
| 35 6 0 4.033706 2.498532 -2.998612 |
| 36 8 0 -5.061975 0.539914 1.605485 |
| 37 6 0 -4.663406 0.302200 2.945018 |
| 38 1 0 -2.483462 4.604964 0.721143 |
| 39 1 0 -4.273129 2.883044 0.821219 |
| 40 1 0 -0.855608 -2.915125 0.131628 |
| 41 1 0 4.683284 -1.752835 -1.785941 |
| 42 1 0 5.597634 -2.642187 -0.547784 |
| 43 1 0 5.686702 -0.101554 -0.411797 |
| 44 1 0 3.566773 0.975613 0.567084 |
| 45 1 0 0.902519 3.689386 0.348941 |
| 46 1 0 -3.654766 -0.797025 0.877686 |
| 47 1 0 -5.646060 0.817895 -0.697226 |
| 48 1 0 -6.782920 -2.794533 -0.033528 |
| 49 1 0 -5.725675 -1.982008 1.120821 |
| 50 1 0 -7.131374 -1.129269 0.471134 |
| 51 1 0 -6.744505 -2.297230 -2.430597 |
| 52 1 0 -5.766835 -0.969142 -3.081882 |
| 53 1 0 -7.198919 -0.615277 -2.106956 |
| 54 1 0 -3.825818 -1.939849 -1.764307 |
| 55 1 0 6.755531 -0.383462 2.984070 |
| 56 1 0 7.085374 0.266322 1.288716 |
| 57 1 0 3.394432 -1.108531 2.554567 |
| 58 1 0 4.213440 -2.608039 2.153830 |
| 59 1 0 4.811652 -1.604001 3.481918 |
| 60 1 0 -3.501165 1.070563 -1.721622 |
| 61 1 0 0.474768 -4.895063 -0.148703 |
| 62 1 0 1.915526 -4.667201 -1.157991 |
| 63 1 0 2.087079 -4.778758 0.579818 |
| 64 1 0 3.047293 2.970858 -3.005085 |
| 65 1 0 4.786349 3.253725 -3.218215 |
| 66 1 0 4.033712 1.725333 -3.768231 |
| 67 1 0 -5.499257 0.596691 3.579566 |
| 68 1 0 -4.443580 -0.760404 3.110934 |
| 69 1 0 -3.781027 0.892780 3.218264 |

**C10**

E = -1763.7196528 a.u

Standard orientation:

| Center Atomic Atomic Coordinates (Angstroms) |
| --- |
| Number Number Type X Y Z |
| 1 6 0 2.690426 2.936728 -1.116930 |
| 2 6 0 3.556088 1.851344 -1.109415 |
| 3 6 0 3.132968 0.544339 -0.845576 |
| 4 6 0 1.765378 0.370051 -0.618762 |
| 5 6 0 0.844503 1.435300 -0.613318 |
| 6 6 0 1.333670 2.749394 -0.862681 |
| 7 8 0 1.327997 -0.904117 -0.398363 |
| 8 6 0 0.008730 -1.175226 -0.173289 |
| 9 6 0 -0.985659 -0.192242 -0.129672 |
| 10 6 0 -0.573029 1.202618 -0.357101 |
| 11 6 0 -0.283587 -2.528748 0.014497 |
| 12 6 0 -1.576013 -2.939488 0.261477 |
| 13 6 0 -2.595485 -1.954574 0.319669 |
| 14 6 0 -2.332302 -0.602123 0.115714 |
| 15 8 0 -3.840665 -2.459353 0.547475 |
| 16 6 0 -4.878662 -1.534139 0.901127 |
| 17 6 0 -4.869418 -0.275285 0.038057 |
| 18 6 0 -3.479444 0.387481 0.159713 |
| 19 8 0 0.523131 3.815320 -0.872314 |
| 20 6 0 4.106184 -0.620961 -0.854447 |
| 21 6 0 5.175143 -0.665712 0.266767 |
| 22 6 0 4.696192 -0.702660 1.736693 |
| 23 6 0 3.705046 -1.838222 1.983616 |
| 24 6 0 5.916549 -0.865694 2.658324 |
| 25 8 0 4.005507 0.508393 2.067098 |
| 26 8 0 -1.377545 2.152534 -0.341704 |
| 27 6 0 -5.335340 -0.475533 -1.399337 |
| 28 6 0 -6.434959 0.155197 -1.819457 |
| 29 6 0 -4.550189 -1.370883 -2.327228 |
| 30 8 0 6.053821 0.454414 0.150220 |
| 31 6 0 -1.919202 -4.387430 0.472360 |
| 32 8 0 -3.376584 1.055101 1.454372 |
| 33 8 0 -4.517409 2.841219 0.664742 |
| 34 6 0 -3.873937 2.315521 1.537657 |
| 35 6 0 -3.501211 2.949102 2.853039 |
| 36 8 0 4.911187 -0.611250 -2.049569 |
| 37 6 0 4.207229 -0.907729 -3.244335 |
| 38 1 0 3.046347 3.939680 -1.321239 |
| 39 1 0 4.603996 2.022810 -1.318695 |
| 40 1 0 0.528118 -3.246302 -0.031777 |
| 41 1 0 -4.744864 -1.257545 1.952786 |
| 42 1 0 -5.806861 -2.095919 0.799503 |
| 43 1 0 -5.573723 0.420670 0.498092 |
| 44 1 0 -3.370255 1.149777 -0.605622 |
| 45 1 0 -0.393192 3.499060 -0.683984 |
| 46 1 0 3.546212 -1.560308 -0.822590 |
| 47 1 0 5.744885 -1.586803 0.081225 |
| 48 1 0 3.443882 -1.866293 3.043721 |
| 49 1 0 2.784599 -1.699742 1.418060 |
| 50 1 0 4.141726 -2.805184 1.717666 |
| 51 1 0 5.584962 -0.851858 3.698807 |
| 52 1 0 6.632405 -0.056095 2.509826 |
| 53 1 0 6.435791 -1.811681 2.474766 |
| 54 1 0 4.564443 1.251667 1.802868 |
| 55 1 0 -6.802919 0.039877 -2.834524 |
| 56 1 0 -6.999502 0.820545 -1.174040 |
| 57 1 0 -3.520845 -1.019828 -2.453065 |
| 58 1 0 -4.487471 -2.394395 -1.947229 |
| 59 1 0 -5.012281 -1.403121 -3.315696 |
| 60 1 0 6.396974 0.458029 -0.752820 |
| 61 1 0 -1.028905 -5.014717 0.405624 |
| 62 1 0 -2.382287 -4.543886 1.450375 |
| 63 1 0 -2.644241 -4.734288 -0.269238 |
| 64 1 0 -2.456984 3.270645 2.805923 |
| 65 1 0 -4.130138 3.819119 3.033387 |
| 66 1 0 -3.589314 2.237883 3.675647 |
| 67 1 0 4.939123 -0.894720 -4.051886 |
| 68 1 0 3.747210 -1.902901 -3.192820 |
| 69 1 0 3.429595 -0.164800 -3.451683 |

1. Lowest energy conformers (14*R*,15*S*,20*S*,25*R*)-**2** for VCD calcation.

**C1**

E = -1763.7264629 a.u

Standard orientation:

| Center Atomic Atomic Coordinates (Angstroms) |
| --- |
| Number Number Type X Y Z |
| 1 6 0 2.229197 -3.398804 0.630152 |
| 2 6 0 3.200176 -2.406153 0.650933 |
| 3 6 0 2.890010 -1.048755 0.531604 |
| 4 6 0 1.542066 -0.723611 0.371966 |
| 5 6 0 0.518497 -1.690229 0.340820 |
| 6 6 0 0.886352 -3.060229 0.476055 |
| 7 8 0 1.236580 0.599808 0.233119 |
| 8 6 0 -0.056099 1.014792 0.075441 |
| 9 6 0 -1.148473 0.141274 0.031886 |
| 10 6 0 -0.875867 -1.299722 0.168242 |
| 11 6 0 -0.212760 2.397604 -0.046693 |
| 12 6 0 -1.463348 2.949120 -0.224785 |
| 13 6 0 -2.581480 2.077426 -0.280811 |
| 14 6 0 -2.452752 0.698052 -0.139073 |
| 15 8 0 -3.770950 2.716125 -0.454598 |
| 16 6 0 -4.932776 1.917015 -0.723707 |
| 17 6 0 -4.982408 0.648100 0.112473 |
| 18 6 0 -3.697948 -0.162841 -0.169510 |
| 19 8 0 -0.027719 -4.039539 0.458701 |
| 20 6 0 3.967350 0.019057 0.545511 |
| 21 6 0 4.659476 0.170900 -0.830586 |
| 22 6 0 5.787377 1.248487 -0.866345 |
| 23 6 0 6.208480 1.513138 -2.311962 |
| 24 6 0 5.385439 2.560755 -0.185491 |
| 25 8 0 6.950227 0.689136 -0.222355 |
| 26 8 0 -1.775173 -2.161047 0.137792 |
| 27 6 0 -5.233904 0.829458 1.604728 |
| 28 6 0 -5.108336 1.998386 2.237909 |
| 29 6 0 -5.689844 -0.412347 2.332614 |
| 30 8 0 5.178894 -1.067801 -1.284147 |
| 31 6 0 -1.661841 4.432067 -0.364678 |
| 32 8 0 -3.764039 -0.743160 -1.508130 |
| 33 8 0 -5.041649 -2.441952 -0.737918 |
| 34 6 0 -4.410689 -1.929402 -1.628767 |
| 35 6 0 -4.219025 -2.506300 -3.007338 |
| 36 8 0 4.989711 -0.255711 1.516840 |
| 37 6 0 4.559699 -0.151954 2.867880 |
| 38 1 0 2.491866 -4.445712 0.725666 |
| 39 1 0 4.240524 -2.683430 0.766596 |
| 40 1 0 0.670181 3.025694 -0.003772 |
| 41 1 0 -4.925853 1.656086 -1.787019 |
| 42 1 0 -5.780287 2.573330 -0.527941 |
| 43 1 0 -5.809284 0.044297 -0.276619 |
| 44 1 0 -3.610170 -0.987244 0.527838 |
| 45 1 0 -0.914921 -3.622595 0.343465 |
| 46 1 0 3.502118 0.978292 0.794601 |
| 47 1 0 3.875139 0.458917 -1.538449 |
| 48 1 0 7.088705 2.159573 -2.325037 |
| 49 1 0 6.456023 0.584011 -2.826125 |
| 50 1 0 5.407024 2.009124 -2.866045 |
| 51 1 0 6.178778 3.299434 -0.320261 |
| 52 1 0 5.231951 2.433767 0.888623 |
| 53 1 0 4.465933 2.970733 -0.615544 |
| 54 1 0 6.692918 0.468374 0.686741 |
| 55 1 0 -5.324130 2.080970 3.298865 |
| 56 1 0 -4.791031 2.906283 1.739488 |
| 57 1 0 -6.627721 -0.790563 1.911455 |
| 58 1 0 -4.970669 -1.232034 2.247271 |
| 59 1 0 -5.849207 -0.212039 3.393768 |
| 60 1 0 6.100279 -1.106777 -0.983714 |
| 61 1 0 -0.709199 4.961811 -0.314689 |
| 62 1 0 -2.146825 4.676848 -1.313453 |
| 63 1 0 -2.314560 4.818837 0.422917 |
| 64 1 0 -3.224760 -2.958475 -3.064035 |
| 65 1 0 -4.966091 -3.276411 -3.191279 |
| 66 1 0 -4.272642 -1.731832 -3.773759 |
| 67 1 0 5.430394 -0.349488 3.493009 |
| 68 1 0 4.180190 0.854813 3.084667 |
| 69 1 0 3.778852 -0.883190 3.097513 |

**C2**

E = -1763.7250504 a.u

Standard orientation:

| Center Atomic Atomic Coordinates (Angstroms) |
| --- |
| Number Number Type X Y Z |
| 1 6 0 -2.246675 3.381501 0.710111 |
| 2 6 0 -3.212959 2.384310 0.701016 |
| 3 6 0 -2.895310 1.031088 0.554731 |
| 4 6 0 -1.544728 0.716131 0.398733 |
| 5 6 0 -0.525688 1.687811 0.395936 |
| 6 6 0 -0.900832 3.052621 0.559828 |
| 7 8 0 -1.231115 -0.603334 0.237060 |
| 8 6 0 0.064051 -1.008246 0.076609 |
| 9 6 0 1.151841 -0.128218 0.050704 |
| 10 6 0 0.872102 1.308092 0.224668 |
| 11 6 0 0.228081 -2.388415 -0.066490 |
| 12 6 0 1.482109 -2.930544 -0.249281 |
| 13 6 0 2.595151 -2.051769 -0.290731 |
| 14 6 0 2.459618 -0.674717 -0.130493 |
| 15 8 0 3.791513 -2.681517 -0.458644 |
| 16 6 0 4.925610 -1.872854 -0.801660 |
| 17 6 0 5.013836 -0.594402 0.027627 |
| 18 6 0 3.699415 0.196887 -0.156964 |
| 19 8 0 0.008461 4.036165 0.572518 |
| 20 6 0 -3.967800 -0.041660 0.539406 |
| 21 6 0 -4.652307 -0.166366 -0.843346 |
| 22 6 0 -5.777200 -1.245542 -0.908624 |
| 23 6 0 -6.189685 -1.478982 -2.362179 |
| 24 6 0 -5.376289 -2.572007 -0.255340 |
| 25 8 0 -6.944861 -0.703130 -0.259047 |
| 26 8 0 1.767828 2.172357 0.229163 |
| 27 6 0 5.412218 -0.796081 1.485141 |
| 28 6 0 6.541430 -0.240693 1.932280 |
| 29 6 0 4.528580 -1.606628 2.402475 |
| 30 8 0 -5.172575 1.080391 -1.273346 |
| 31 6 0 1.689449 -4.410284 -0.409824 |
| 32 8 0 3.702629 0.837497 -1.469527 |
| 33 8 0 5.006221 2.512298 -0.688198 |
| 34 6 0 4.333324 2.035775 -1.567080 |
| 35 6 0 4.064030 2.672830 -2.905834 |
| 36 8 0 -4.996094 0.207454 1.511304 |
| 37 6 0 -4.572860 0.073990 2.861825 |
| 38 1 0 -2.515031 4.424754 0.827263 |
| 39 1 0 -4.255456 2.654079 0.814854 |
| 40 1 0 -0.651899 -3.021363 -0.035984 |
| 41 1 0 4.852087 -1.615658 -1.864183 |
| 42 1 0 5.790449 -2.519377 -0.654804 |
| 43 1 0 5.796826 0.016255 -0.425986 |
| 44 1 0 3.639677 0.985516 0.586838 |
| 45 1 0 0.899312 3.626174 0.460686 |
| 46 1 0 -3.499181 -1.003903 0.770152 |
| 47 1 0 -3.863295 -0.437049 -1.552882 |
| 48 1 0 -7.067760 -2.127619 -2.394534 |
| 49 1 0 -6.437477 -0.539386 -2.856758 |
| 50 1 0 -5.383668 -1.959880 -2.922895 |
| 51 1 0 -6.166194 -3.309693 -0.413570 |
| 52 1 0 -5.231792 -2.470189 0.822707 |
| 53 1 0 -4.452269 -2.969225 -0.687714 |
| 54 1 0 -6.692714 -0.500976 0.655793 |
| 55 1 0 6.862452 -0.358553 2.962854 |
| 56 1 0 7.177887 0.364424 1.294584 |
| 57 1 0 3.527684 -1.171395 2.489887 |
| 58 1 0 4.394668 -2.629076 2.038644 |
| 59 1 0 4.956084 -1.656307 3.405686 |
| 60 1 0 -6.096095 1.110219 -0.978422 |
| 61 1 0 0.739669 -4.945993 -0.370403 |
| 62 1 0 2.178555 -4.638258 -1.360642 |
| 63 1 0 2.342587 -4.805129 0.373405 |
| 64 1 0 3.066612 3.121152 -2.887238 |
| 65 1 0 4.797050 3.455089 -3.095081 |
| 66 1 0 4.078487 1.934128 -3.708444 |
| 67 1 0 -5.447914 0.252241 3.486631 |
| 68 1 0 -4.189374 -0.935771 3.056769 |
| 69 1 0 -3.797106 0.803569 3.113067 |
